# Supplementary material for: Important Food Sources of Fructose-Containing Sugars and Non-Alcoholic Fatty Liver Disease: A Systematic Review and Meta-Analysis of Controlled Trials
Source: Nutrients. 2022 Jul 12;14(14):2846. doi: 10.3390/nu14142846 (PMC9325155; doi:10.3390/nu14142846)
Supplement: Supplementary file 1 [file nutrients-14-02846-s001.zip › nutrients-1779950-supplementary.pdf]

Online supplementary File to "Important Food Sources of Fructose-Containing Sugars and Non-Alcoholic Fatty Liver Disease: A Systematic Review and Meta-Analysis of Controlled Trials"

All Supplementary references are found within the Supplementary bibliography.

Table of Contents

Supplementary Tables 7

Supplementary Table S1: PRISMA Checklist 7

Supplementary Table S2: Search strategy for controlled trials assessing the effect of important food sources of fructose-containing sugars and NAFLD outcomes 10

Supplementary Table S3: PICO framework of the search strategy 12

Supplementary Table S4: Food source of fructose-containing sugars definitions 13

Supplementary Table S5: Trial characteristics 14

Supplementary Table S6: Sensitivity analyses of the use of correlation coefficients of 0.25 and 0.75 for crossover trials in the primary analysis of the effect of important food sources of fructose-containing sugars and NAFLD outcomes 22

Supplementary Table S7: GRADE certainty of evidence assessment for the effect of fructose-containing sugars and NAFLD outcomes by levels of energy control 24

Supplementary Table S8: GRADE certainty of evidence assessment for the effect of fructose-containing sugars and NAFLD outcomes by important food source of fructose-containing sugars 27

Supplementary Table S9: Potential mechanisms to explain the effect of food sources of fructose-containing sugars and non-alcoholic fatty liver disease 31

Supplementary Figures 33

Supplementary Figure S1: Risk of bias proportion graph for the effect of important food sources of fructose-containing sugars and IHCL (SMD) in substitution trials 33

Supplementary Figure S2: Risk of bias proportion graph for the effect of important food sources of fructose-containing sugars and IHCL (SMD) in addition trials 33

Supplementary Figure S3: Risk of bias proportion graph for the effect of important food sources of fructose-containing sugars and IHCL (SMD) in subtraction trials 34

Supplementary Figure S4: Risk of bias proportion graph for the effect of important food sources of fructose-containing sugars and ALT (U/L) in substitution trials 34

Supplementary Figure S5: Risk of bias proportion graph for the effect of important food sources of fructose-containing sugars and ALT (U/L) in addition trials 35

Supplementary Figure S6: Risk of bias proportion graph for the effect of important food sources of fructose-containing sugars and ALT (U/L) in subtraction trials 35

Supplementary Figure S7: Risk of bias proportion graph for the effect of important food sources of fructose-containing sugars and ALT (U/L) in *ad libitum* trials 36

Supplementary Figure S8: Risk of bias proportion graph for the effect of important food sources of fructose-containing sugars and AST (U/L) in substitution trials 36

Supplementary Figure S9: Risk of bias proportion graph for the effect of important food sources of fructose-containing sugars and AST (U/L) in addition trials 37

Supplementary Figure S10: Risk of bias proportion graph for the effect of important food sources of fructose-containing sugars and AST (U/L) in subtraction trials 37

Supplementary Figure S11: Risk of bias proportion graph for the effect of important food sources of fructose-containing sugars and AST (U/L) in *ad libitum* trials 38

Supplementary Figure S12: Forest plot of controlled trials of the effect of important food sources of fructose-containing sugars and IHCL (SMD) in substitution trials 39

Supplementary Figure S13: Forest plot of controlled trials of the effect of important food sources of fructose-containing sugars and IHCL (SMD) in addition trials 41

Supplementary Figure S14: Forest plot of controlled trials of the effect of important food sources of fructose-containing sugars and IHCL (SMD) in subtraction trials 42

Supplementary Figure S15: Forest plot of controlled trials of the effect of important food sources of fructose-containing sugars and ALT (U/L) in substitution trials 43

Supplementary Figure S16: Forest plot of controlled trials of the effect of important food sources of fructose-containing sugars and ALT (U/L) in addition trials 45

Supplementary Figure S17: Forest plot of controlled trials of the effect of important food sources of fructose-containing sugars and ALT (U/L) in subtraction trials 47

Supplementary Figure S18: Forest plot of controlled trials of the effect of important food sources of fructose-containing sugars and ALT (U/L) in *ad libitum* trials 48

Supplementary Figure S19: Forest plot of controlled trials of the effect of important food sources of fructose-containing sugars and AST (U/L) in substitution trials 49

Supplementary Figure S20: Forest plot of controlled trials of the effect of important food sources of fructose-containing sugars and AST (U/L) in addition trials 51

Supplementary Figure S21: Forest plot of controlled trials of the effect of important food sources of fructose-containing sugars and AST (U/L) in subtraction trials 53

Supplementary Figure S22: Forest plot of controlled trials of the effect of important food sources of fructose-containing sugars and AST (U/L) in *ad libitum* trials 54

Supplementary Figure S23: Sensitivity analysis of the systematic removal of each trial for the effect of important food sources of fructose-containing sugars and IHCL (SMD) in substitution trials 55

Supplementary Figure S24: Sensitivity analysis of the systematic removal of each trial for the effect of important food sources of fructose-containing sugars and IHCL (SMD) in addition trials 56

Supplementary Figure S25: Sensitivity analysis of the systematic removal of each trial in the primary analysis of the effect of important food sources of fructose-containing sugars and IHCL (SMD) in subtraction trials 56

Supplementary Figure S26: Sensitivity analysis of the systematic removal of each trial for the effect of important food sources of fructose-containing sugars and ALT (U/L) in substitution trials 57

Supplementary Figure S27: Sensitivity analysis of the systematic removal of each trial for the effect of important food sources of fructose-containing sugars and ALT (U/L) in addition trials 58

Supplementary Figure S28: Sensitivity analysis of the systematic removal of each trial for the effect of important food sources of fructose-containing sugars and ALT (U/L) in subtraction trials 59

Supplementary Figure S29: Sensitivity analysis of the systematic removal of each trial for the effect of important food sources of fructose-containing sugars and ALT (U/L) in *ad libitum* trials 59

Supplementary Figure S30: Sensitivity analysis of the systematic removal of each trial for the effect of important food sources of fructose-containing sugars and AST (U/L) in substitution trials 60

Supplementary Figure S31: Sensitivity analysis of the systematic removal of each trial for the effect of important food sources of fructose-containing sugars and AST (U/L) in addition trials 61

Supplementary Figure S32: Sensitivity analysis of the systematic removal of each trial in the primary analysis of the effect of important food sources of fructose-containing sugars and AST (U/L) in subtraction trials 62

Supplementary Figure S33: Sensitivity analysis of the systematic removal of each trial in the primary analysis of the effect of important food sources of fructose-containing sugars and AST (U/L) in *ad libitum* trials 62

Supplementary Figure S34: Sensitivity analysis of the systematic removal of each trial for the effect of SSBs on ALT (U/L) in addition trials 63

Supplementary Figure S35: Sensitivity analysis of the systematic removal of each trial for the effect of 100% fruit juice on ALT (U/L) in addition trials....64

Supplementary Figure S36: Sensitivity analysis of the systematic removal of each trial for the effect of fruit on ALT (U/L) in addition trials....64

Supplementary Figure S37: Sensitivity analysis of the systematic removal of each trial for the effect of dried fruit on ALT (U/L) in addition trials....65

Supplementary Figure S38: Sensitivity analysis of the systematic removal of each trial for the effect of honey on ALT (U/L) in addition trials....65

Supplementary Figure S39: Sensitivity analysis of the systematic removal of each trial for the effect of important food sources of fructose-containing sugars and ALT (U/L) in subtraction SSB trials....66

Supplementary Figure S40: Sensitivity analysis of the systematic removal of each trial for the effect of important food sources of fructose-containing sugars and ALT (U/L) in subtraction mixed sources (with SSBs) trials....66

Supplementary Figure S41: Sensitivity analysis of the systematic removal of each trial for the effect of important food sources of fructose-containing sugars and AST (U/L) in addition SSB trials....67

Supplementary Figure S42: Sensitivity analysis of the systematic removal of each trial for the effect of important food sources of fructose-containing sugars and AST (U/L) in addition 100% fruit juice trials....67

Supplementary Figure S43: Sensitivity analysis of the systematic removal of each trial for the effect of important food sources of fructose-containing sugars and AST (U/L) in addition fruit trials....68

Supplementary Figure S44: Sensitivity analysis of the systematic removal of each trial for the effect of important food sources of fructose-containing sugars and AST (U/L) in addition dried fruit trials....68

Supplementary Figure S45: Sensitivity analysis of the systematic removal of each trial for the effect of important food sources of fructose-containing sugars and AST (U/L) in addition honey trials....69

Supplementary Figure S46: Sensitivity analysis of the systematic removal of each trial in the primary analysis of the effect of important food sources of fructose-containing sugars and AST (U/L) in subtraction SSB trials....69

Supplementary Figure S47: Sensitivity analysis of the systematic removal of each trial in the primary analysis of the effect of important food sources of fructose-containing sugars and AST (U/L) in subtraction mixed sources (with SSBs) trials....70

Supplementary Figure S48 (part 1 of 3): Subgroup analyses for the effect of important food sources of fructose-containing sugars and IHCL (SMD) in substitution trials....71

Supplementary Figure S48 (part 2 of 3): Subgroup analyses for the effect of important food sources of fructose-containing sugars and IHCL (SMD) in substitution trials....72

Supplementary Figure S48 (part 3 of 3): Subgroup analyses for the effect of important food sources of fructose-containing sugars and IHCL (SMD) in substitution trials....73

Supplementary Figure S49: Risk of bias (using The Cochrane Collaboration Tool) subgroup analysis for the effect of important food sources of fructose-containing sugars and IHCL (SMD) in substitution trials....74

Supplementary Figure S50 (part 1 of 3): Subgroup analyses for the effect of important food sources of fructose-containing sugars and IHCL (SMD) in addition trials....75

Supplementary Figure S50 (part 2 of 3): A priori subgroup analyses for the effect of important food sources of fructose-containing sugars and IHCL (SMD) in addition trials....75

Supplementary Figure S50 (part 3 of 3): A priori subgroup analyses for the effect of important food sources of fructose-containing sugars and IHCL (SMD) in addition trials....77

Supplementary Figure S51: Risk of bias (using The Cochrane Collaboration Tool) subgroup analysis for the effect of important food sources of fructose-containing sugars and IHCL (SMD) in addition trials....78

Supplementary Figure S52 (part 1 of 3): Subgroup analyses for the effect of important food sources of fructose-containing sugars and ALT (U/L) in substitution trials....79

Supplementary Figure S52 (part 2 of 3): Subgroup analyses for the effect of important food sources of fructose-containing sugars and ALT (U/L) in substitution trials 80
Supplementary Figure S52 (part 3 of 3): A priori subgroup analyses for the effect of important food sources of fructose-containing sugars and ALT (U/L) in substitution trials 81
Supplementary Figure S53: Risk of bias (using The Cochrane Collaboration Tool) subgroup analysis for the effect of important food sources of fructose-containing sugars and ALT (U/L) in substitution trials 82
Supplementary Figure S54 (part 1 of 3): A priori subgroup analyses for the effect of important food sources of fructose-containing sugars and ALT (U/L) in addition trials........83
Supplementary Figure S54 (part 2 of 3): A priori subgroup analyses for the effect of important food sources of fructose-containing sugars and ALT (U/L) in addition trials........85
Supplementary Figure S54 (part 3 of 3): A priori subgroup analyses for the effect of important food sources of fructose-containing sugars and ALT (U/L) in addition trials........86
Supplementary Figure S55: Risk of bias (using The Cochrane Collaboration Tool) subgroup analysis for the effect of important food sources of fructose-containing sugars and ALT (U/L) in addition trials 87
Supplementary Figure S56 (part 1 of 3): A priori subgroup analyses for the effect of important food sources of fructose-containing sugars and ALT (U/L) in addition SSB trials 89
Supplementary Figure S56 (part 2 of 3): A priori subgroup analyses for the effect of important food sources of fructose-containing sugars and ALT (U/L) in addition SSB trials 90
Supplementary Figure S56 (part 3 of 3): A priori subgroup analyses for the effect of important food sources of fructose-containing sugars and ALT (U/L) in addition SSB trials 91
Supplementary Figure S57: Risk of bias (using The Cochrane Collaboration Tool) subgroup analysis for the effect of important food sources of fructose-containing sugars and ALT (U/L) in addition SSB trials 92
Supplementary Figure S58 (part 1 of 3): A priori subgroup analyses for the effect of important food sources of fructose-containing sugars and AST (U/L) in substitution trials 93
Supplementary Figure S58 (part 2 of 3): A priori subgroup analyses for the effect of important food sources of fructose-containing sugars and AST (U/L) in substitution trials 94
Supplementary Figure S58 (part 3 of 3): A priori subgroup analyses for the effect of important food sources of fructose-containing sugars and AST (U/L) in substitution trials 95
Supplementary Figure S59: Risk of bias (using The Cochrane Collaboration Tool) subgroup analysis for the effect of important food sources of fructose-containing sugars and AST (U/L) in substitution trials 96
Supplementary Figure S60 (part 1 of 3): A priori subgroup analyses for the effect of important food sources of fructose-containing sugars and AST (U/L) in addition trials........97
Supplementary Figure S60 (part 2 of 3): A priori subgroup analyses for the effect of important food sources of fructose-containing sugars and AST (U/L) in addition trials........98
Supplementary Figure S60 (part 3 of 3): A priori subgroup analyses for the effect of important food sources of fructose-containing sugars and AST (U/L) in addition trials........99
Supplementary Figure S61: Risk of bias (using The Cochrane Collaboration Tool) subgroup analysis for the effect of important food sources of fructose-containing sugars and AST (U/L) in addition trials........100
Supplementary Figure S62: Continuous meta-regression analysis for the effect of important food sources of fructose-containing sugars and IHCL (SMD) in substitution trials 101
Supplementary Figure S63: Continuous meta-regression analysis for the effect of important food sources of fructose-containing sugars and IHCL (SMD) in addition trials........101
Supplementary Figure S64: Continuous meta-regression analysis for the effect of important food sources of fructose-containing sugars and ALT (U/L) in substitution trials 102
Supplementary Figure S65: Continuous meta-regression analysis for the effect of important food sources of fructose-containing sugars and ALT (U/L) in addition trials........102

Supplementary Figure S66: Continuous meta-regression analysis for the effect of important food sources of fructose-containing sugars and ALT (U/L) in addition SSB trials 103

Supplementary Figure S67: Continuous meta-regression analysis for the effect of important food sources of fructose-containing sugars and AST (U/L) in substitution trials 104

Supplementary Figure S68: Continuous meta-regression analysis for the effect of important food sources of fructose-containing sugars and AST (U/L) in addition trials....................104

Supplementary Figure S69: Linear and non-linear meta-regression analyses for the effect of important food sources of fructose-containing sugars dose and IHCL (SMD) in substitution trials....................105

Supplementary Figure S70: Linear and non-linear meta-regression analyses for the effect of important food sources of SSB sugars dose and IHCL (SMD) in substitution trials .... **Error! Bookmark not defined.**

Supplementary Figure S71: Linear and non-linear meta-regression analyses for the effect of important food sources of fructose-containing sugars dose and IHCL (SMD) in addition trials 106

Supplementary Figure S72: Linear and non-linear meta-regression analyses for the effect of important food sources of fructose-containing sugars dose and ALT (U/L) in substitution trials 107

Supplementary Figure S73: Linear and non-linear meta-regression analyses for the effect of important food sources of fructose-containing sugars dose and ALT (U/L) in addition trials....................108

Supplementary Figure S74: Linear and non-linear meta-regression analyses for the effect of SSB sugars dose and ALT (U/L) in addition trials 109

Supplementary Figure S75: Linear and non-linear meta-regression analyses for the effect of important food sources of fructose-containing sugars dose and ALT (U/L) in subtraction trials 111

Supplementary Figure S76: Linear and non-linear meta-regression analyses for the effect of important food sources of fructose-containing sugars dose and AST (U/L) in substitution trials....................112

Supplementary Figure S77: Linear and non-linear meta-regression analyses for the effect of important food sources of fructose-containing sugars dose and AST (U/L) in addition trials 113

Supplementary Figure S77: Linear and non-linear meta-regression analyses for the effect of important food sources of fructose-containing sugars dose and AST (U/L) in addition SSB trials 114

Supplementary Figure S78: Linear and non-linear meta-regression analyses for the effect of important food sources of fructose-containing sugars dose and AST (U/L) in subtraction trials....................115

Supplementary Figure S79: Non-linear dose-response analysis using public thresholds of 5%, 10%, and 25% of energy for the effect of important food sources of fructose-containing sugars and IHCL (SMD) in substitution trials 116

Supplementary Figure S80: Non-linear dose-response analysis using public threshold of 25% energy for the effect of important food sources of fructose-containing sugars and IHCL (SMD) in addition trials....................118

Supplementary Figure S81: Non-linear dose-response analysis using public thresholds of 5%, 10%, and 25% of energy for the effect of important food sources of fructose-containing sugars and ALT (U/L) in substitution trials 119

Supplementary Figure S82: Non-linear dose-response analysis using public thresholds of 5%, 10%, and 25% of energy for the effect of important food sources of fructose-containing sugars and ALT (U/L) in addition trials 121

Supplementary Figure S83: Non-linear dose-response analysis using public thresholds of 10% and 25% of energy for the effect of SSBs on ALT (U/L) in addition trials....................123

Supplementary Figure S84: Non-linear dose-response analysis using public thresholds of 5%, 10%, and 25% of energy for the effect of important food sources of fructose-containing sugars and AST (U/L) in substitution trials 125

Supplementary Figure S85: Non-linear dose-response analysis using public thresholds of 5%, 10%, and 25% of energy for the effect of important food sources of fructose-containing sugars and AST (U/L) in addition trials 127

Supplementary Figure S86: Non-linear dose-response analysis using public thresholds of 5%, 10%, and 25% of energy for the effect of important food sources of fructose-containing sugars and AST (U/L) in addition SSB trials 129

Supplementary Figure S87: Publication bias funnel plots for the effect of important food sources of fructose-containing sugars and IHCL (SMD) in substitution trials 130

Supplementary Figure S88: Publication bias funnel plots for the effect of important food sources of fructose-containing sugars and IHCL (SMD) in addition trials* 131

Supplementary Figure S89: Publication bias funnel plots for the effect of important food sources of fructose-containing sugars and ALT (U/L) in substitution trials 132

Supplementary Figure S90: Publication bias funnel plots for the effect of important food sources of fructose-containing sugars and ALT (U/L) in addition trials 133

Supplementary Figure S91: Publication bias funnel plots for the effect of important food sources of fructose-containing sugars and ALT (U/L) in addition SSB trials 134

Supplementary Figure S92: Publication bias funnel plots for the effect of important food sources of fructose-containing sugars and AST (U/L) in substitution trials 135

Supplementary Figure S93: Publication bias funnel plots for the effect of important food sources of fructose-containing sugars and AST (U/L) in addition trials 136

Supplementary Figure S94: Trim and Fill funnel plot for the effect of important food sources of fructose-containing sugars IHCL (SMD) in substitution trials 137

Supplementary Figure S95: Trim and Fill funnel plot for the effect of important food sources of fructose-containing sugars IHCL (SMD) in addition trials 138

Supplementary Figure S96: Trim and Fill funnel plot for the effect of important food sources of fructose-containing sugars ALT (U/L) in substitution trials 139

Supplementary Figure S97: Trim and Fill funnel plot for the effect of important food sources of fructose-containing sugars AST (U/L) in substitution trials 140

Supplementary References 141

Supplementary Tables

Supplementary Table S1: PRISMA Checklist

Section and Topic	Item #	Checklist item	Location where item is reported
**TITLE**			
Title	1	Identify the report as a systematic review.	Page 1
**ABSTRACT**			
Abstract	2	See the PRISMA 2020 for Abstracts checklist.	Pages 1,2
**INTRODUCTION**			
Rationale	3	Describe the rationale for the review in the context of existing knowledge.	Page 2
Objectives	4	Provide an explicit statement of the objective(s) or question(s) the review addresses.	Pages 2
**METHODS**			
Eligibility criteria	5	Specify the inclusion and exclusion criteria for the review and how studies were grouped for the syntheses.	Page 3, Table S3
Information sources	6	Specify all databases, registers, websites, organisations, reference lists and other sources searched or consulted to identify studies. Specify the date when each source was last searched or consulted.	Page 3
Search strategy	7	Present the full search strategies for all databases, registers and websites, including any filters and limits used.	Page 3, Table S1
Selection process	8	Specify the methods used to decide whether a study met the inclusion criteria of the review, including how many reviewers screened each record and each report retrieved, whether they worked independently, and if applicable, details of automation tools used in the process.	Pages 3-5
Data collection process	9	Specify the methods used to collect data from reports, including how many reviewers collected data from each report, whether they worked independently, any processes for obtaining or confirming data from study investigators, and if applicable, details of automation tools used in the process.	Pages 3,4
Data items	10a	List and define all outcomes for which data were sought. Specify whether all results that were compatible with each outcome domain in each study were sought (e.g. for all measures, time points, analyses), and if not, the methods used to decide which results to collect.	Page 4
	10b	List and define all other variables for which data were sought (e.g. participant and intervention characteristics, funding sources). Describe any assumptions made about any missing or unclear information.	Page 4
Study risk of bias assessment	11	Specify the methods used to assess risk of bias in the included studies, including details of the tool(s) used, how many reviewers assessed each study and whether they worked independently, and if applicable, details of automation tools used in the process.	Page 4
Effect measures	12	Specify for each outcome the effect measure(s) (e.g. risk ratio, mean difference) used in the synthesis or presentation of results.	Pages 4,5
Synthesis methods	13a	Describe the processes used to decide which studies were eligible for each synthesis (e.g. tabulating the study intervention characteristics and comparing against the planned groups for each synthesis (item #5)).	Page 4, Table S3
	13b	Describe any methods required to prepare the data for presentation or synthesis, such as handling of missing summary statistics, or data conversions.	Pages 4,5
	13c	Describe any methods used to tabulate or visually display results of individual studies and syntheses.	Pages 4,5
	13d	Describe any methods used to synthesize results and provide a rationale for the choice(s). If meta-analysis was performed, describe the model(s), method(s) to identify the presence and extent of statistical heterogeneity, and software package(s) used.	Pages 4,5
	13e	Describe any methods used to explore possible causes of heterogeneity among study results (e.g. subgroup analysis, meta-regression).	Pages 4,5

	13f	Describe any sensitivity analyses conducted to assess robustness of the synthesized results.	Pages 4,5
Reporting bias assessment	14	Describe any methods used to assess risk of bias due to missing results in a synthesis (arising from reporting biases).	Page 5
Certainty assessment	15	Describe any methods used to assess certainty (or confidence) in the body of evidence for an outcome.	Page 5
**RESULTS**			
Study selection	16a	Describe the results of the search and selection process, from the number of records identified in the search to the number of studies included in the review, ideally using a flow diagram.	Page 6, Figure 1
	16b	Cite studies that might appear to meet the inclusion criteria, but which were excluded, and explain why they were excluded.	Figure 1
Study characteristics	17	Cite each included study and present its characteristics.	Page 6, Table 1, S5
Risk of bias in studies	18	Present assessments of risk of bias for each included study.	Page 9, Figures S1-11
Results of individual studies	19	For all outcomes, present, for each study: (a) summary statistics for each group (where appropriate) and (b) an effect estimate and its precision (e.g. confidence/credible interval), ideally using structured tables or plots.	Pages 9-18, Figures 2-4 and S12-22
Results of syntheses	20a	For each synthesis, briefly summarise the characteristics and risk of bias among contributing studies.	Figures S1-11
	20b	Present results of all statistical syntheses conducted. If meta-analysis was done, present for each the summary estimate and its precision (e.g. confidence/credible interval) and measures of statistical heterogeneity. If comparing groups, describe the direction of the effect.	Pages 9-19, Figures 2-4 and S12-97
	20c	Present results of all investigations of possible causes of heterogeneity among study results.	Page 18, Figures S23-47
	20d	Present results of all sensitivity analyses conducted to assess the robustness of the synthesized results.	Pages 18, Figures S23-47
Reporting biases	21	Present assessments of risk of bias due to missing results (arising from reporting biases) for each synthesis assessed.	Page 19
Certainty of evidence	22	Present assessments of certainty (or confidence) in the body of evidence for each outcome assessed.	Page 19, Figures 2-4, Tables S7-8
**DISCUSSION**			
Discussion	23a	Provide a general interpretation of the results in the context of other evidence.	Page 20
	23b	Discuss any limitations of the evidence included in the review.	Pages 20,21
	23c	Discuss any limitations of the review processes used.	Pages 20,21
	23d	Discuss implications of the results for practice, policy, and future research.	Page 21
**OTHER INFORMATION**			
Registration and protocol	24a	Provide registration information for the review, including register name and registration number, or state that the review was not registered.	Page 3
	24b	Indicate where the review protocol can be accessed, or state that a protocol was not prepared.	Page 3
	24c	Describe and explain any amendments to information provided at registration or in the protocol.	N/A
Support	25	Describe sources of financial or non-financial support for the review, and the role of the funders or sponsors in the review.	Pages 22-25
Competing interests	26	Declare any competing interests of review authors.	Pages 22-25
Availability of data, code and other materials	27	Report which of the following are publicly available and where they can be found: template data collection forms; data extracted from included studies; data used for all analyses; analytic code; any other materials used in the review.	Tables S2-5, Figures 2-4 and S12-22

N/A=not applicable.
Table obtained from Page et al. 2021 (1).

Supplementary Table S2: Search strategy for controlled trials assessing the effect of important food sources of fructose-containing sugars and NAFLD outcomes

Database and search terms		
**MEDLINE**	**EMBASE**	**The Cochrane Library of Controlled Studies**
1. exp fructose/	1. exp fructose/	1. exp fructose/
2. fructose.mp.	2. fructose.mp.	2. fructose.mp.
3. exp dietary sucrose/	3. exp sucrose/	3. exp sucrose/
4. sucrose.mp.	4. sucrose.mp.	4. sucrose.mp.
5. sweetened*.mp.	5. sugar*.mp.	5. sugar*.mp.
6. sugar*.mp.	6. SSB.mp.	6. cola.mp.
7. SSB.mp.	7. exp honey/	7. SSB.mp.
8. soft drink*.mp.	8. honey.mp.	8. soft drink.mp.
9. cola*.mp.	9. fruit.mp.	9. exp honey/
10. exp honey/	10. exp fruit/	10. honey.mp.
11. honey.mp.	11. exp soft drink/	11. fruit.mp.
12. fruit.mp.	12. soft drink*.mp.	12. sweetened.mp.
13. exp fruit/	13. exp carbonated beverage/	13. exp carbonated beverages/
14. exp sucrose/	14. carbonated beverage*.mp.	14. carbonated beverage*.mp.
15. exp soft drink/	15. exp energy drink/	15. exp energy drinks/
16. exp carbonated beverage/	16. energy drink*.mp.	16. energy drink*.mp.
17. carbonated beverage*.mp.	17. sweetened.mp.	17. exp fruit/
18. exp energy drink/	18. cola.mp.	18. HFCS.mp.
19. energy drink*.mp.	19. HFCS.mp.	19. exp energy drink*.mp.
20. HFCS.mp.	20. sugar*sweetened*beverage*.mp.	20. sugar*sweetened*beverage*.mp.
21. sugar*sweetened beverage*.mp.	21. exp fatty liver/	21. exp fatty liver/
22. exp fatty liver/	22. fatty liver.mp.	22. fatty liver.mp.
23. fatty liver.mp.	23. NAFLD.mp.	23. NAFLD.mp.
24. exp NAFLD/	24. NAFL.mp.	24. NAFL.mp.
25. NAFLD.mp.	25. NASH.mp.	25. NASH.mp.
26. NAFL.mp.	26. liver stenosis.mp.	26. liver cirrhosis.mp.
27. NASH.mp.	27. liver cirrhosis.mp.	27. HCC.mp.
28. liver stenosis.mp.	28. HCC.mp.	28. exp carcinoma, hepatocellular/
29. liver cirrhosis.mp.	29. exp hepatocellular carcinoma/	29. hepatocellular carcinoma.mp.
30. HCC.mp.	30. hepatocellular carcinoma.mp.	30. liver biopsy.mp.
31. exp hepatocellular carcinoma/	31. liver biopsy.mp.	31. liver histology.mp.
32. hepatocellular carcinoma.mp.	32. liver histology.mp.	32. liver inflammation.mp.
33. liver biopsy.mp.	33. liver inflammation.mp.	33. exp liver cirrhosis/
34. liver histology.mp.	34. exp liver fibrosis/	34. liver fibrosis.mp.
35. liver inflammation.mp.	35. liver fibrosis.mp.	35. exp magnetic resonance imaging/
36. exp liver fibrosis/	36. exp magnetic resonance imaging/	36. magnetic resonance imaging.mp.
37. liver fibrosis.mp.	37. magnetic resonance imaging.mp.	37. MRI.mp.
38. exp magnetic resonance imaging/	38. MRI.mp.	38. MRS.mp.
39. magnetic resonance imaging.mp.	39. MRS.mp.	39. NMRI.mp.
40. MRI.mp.	40. NMRI.mp.	40. intrahepatocellular lipid.mp.
41. MRS.mp.	41. intrahepatocellular lipid.mp.	41. alanine aminotransferase*.mp.
42. NMRI.mp.	42. alanine aminotransferase*.mp.	42. ALT.mp.
43. intrahepatocellular lipid.mp.	43. ALT.mp.	43. AST.mp.
44. alanine aminotransferase*.mp.	44. AST.mp.	44. aspartate aminotransferase.mp.
45. ALT.mp.	45. aspartate aminotransferase.mp.	45. exp gamma-glutamyltransferase/
46. AST.mp.	46. alkaline phosphatase.mp.	46. GGT.mp.
47. aspartate aminotransferase.mp.	47. ALP.mp.	47. alkaline phosphatase.mp.
48. exp GGT/	48. adipocytes.mp.	48. ALP.mp.
49. GGT.mp.	49. liver enzyme*.mp.	49. adipocytes.mp.
50. alkaline phosphatase.mp.	50. exp transaminases/	50. liver enzyme*.mp.
51. ALP.mp.	51. transaminases.mp.	51. exp transaminases/
52. adipocytes.mp.	52. proton imaging.mp.	52. transaminases.mp.
53. liver enzyme*.mp.	53. random:.tw.	53. proton imaging.mp.
54. exp transaminases/	54. clinical trial:.mp.	54. or/1-19
55. transaminases.mp.	55. exp health care quality/	55. or/20-53
56. proton imaging.mp.	56. or/1-20	56. 54 and 55
57. clinical trial.mp.	57. or/21-52	
58. clinical trial.pt.	58. or/53-55	
59. random:.mp.	59. 56 and 57	
60. tu:.xs.	60. limit 59 to animals	
61. or/1-21	61. 59 not 60	
62. or/22-56	62. limit 61 to animal studies	
63. or/57-60	63. 61 not 62	

64. 61 and 62<br>65. limit 64 to animals<br>66. 64 not 65<br>67. 63 and 66	64. 58 and 63	

ALT=alanine aminotransferase; ALP=alkaline phosphatase; AST=aspartate aminotransferase; GGT=gamma-glutamyl transferase; HCC=hepatocellular carcinoma; HFCS=high-fructose corn syrup; MRI=magnetic resonance imaging; MRS=magnetic resonance spectroscopy; NAFL=non-alcoholic fatty liver; NAFLD=non-alcoholic fatty liver disease; NASH=non-alcoholic steatohepatitis; NMRI=nuclear magnetic resonance imaging; SSB=sugar sweetened beverage.

Supplementary Table S3: PICO framework of the search strategy

PICO framework[1] defined in the present systematic review and meta-analysis					
**Participants**	**Interventions**	**Comparators**	**Outcomes**	**Time**	**Study design**
Individuals of all ages and health backgrounds	Food sources of fructose-containing sugars	Diets and foods free or lower (minimum 5g sugar difference) in fructose-containing sugars.	IHCL, ALT, and AST, mean difference and 95% confidence intervals	≥7 days	Controlled trials done in humans

ALT=alanine aminotransferase; AST=aspartate aminotransferase; IHCL=intrahepatocellular lipid; PICOTS=participants, interventions, comparators, outcomes, time and study design.

Supplementary Table S4: Food source of fructose-containing sugars definitions

Food source of fructose-containing sugars	Definition
SSB	Carbonated or non-carbonated beverages where all or the majority of sugars are added sugars. This also includes interventions where sugars were provided to participants as crystalline packages and where they are instructed to add or incorporate into beverages.
Sweetened dairy	Animal dairy products sweetened with added sugars and where the control includes non-dairy products. These would contain both added and naturally occurring sugars.
Sweetened dairy alternatives (soy)	Soy-based dairy products sweetened with added sugars and where the control includes non-soy-based dairy products.
Sweetened dairy alternative (other)	Other plant-based dairy products sweetened with added sugars and where the control includes non-plant-based dairy products.
Fruit drink	Fruit drinks which are derived from fruit juices or fruit flavouring with added sugars. These must contain added and may also contain naturally occurring sugars.
100% Fruit juice	Fruit juice which is derived 100% from fruits with no added sugar. The one exception was cranberry juice, in which a small amount of added sugars was added for palatability.
Fruit	Includes whole fruit, freeze-dried powdered fruit, smoothies in which the only difference between intervention groups is the fruit present. The dose of the sugars under investigation is naturally occurring coming from fruit.
Dried fruit	Includes unsweetened and sweetened dried fruit. Sugars can be naturally occurring, or both naturally occurring and added.
Mixed fruit forms	Interventions include two or more of the food sources of fruit sugars (i.e., fruit, dried fruit, 100% fruit juice). Sugars are naturally occurring coming from fruit.
Sweetened cereal grains and bars	Includes sweetened dried cereal, nut bars and fruit and nut bars. Sugars are added.
Sweets and desserts	Includes cookies, cakes, muffins, confectionaries, fondant, etc. Sugars are added.
Added nutritive (caloric) sweetener	Sugars provided to participants as crystalline packages or syrup or honey, where they are instructed to add or incorporate it to various foods. Sugars are added regulatory designations.
Mixed sources (with SSBs)	Interventions where fructose-containing sugars were consumed in the form of SSBs in addition to other food sources. Examples include whole dietary interventions. Sugars can be added, or both naturally occurring and added.
Mixed sources (without SSBs)	Interventions include two or more of the above food sources of fructose-containing sugars with the exception of SSBs. Sugars can be added, or both naturally occurring and added.

SSB=sugar-sweetened beverage.

Supplementary Table S5: Trial characteristics

Study, year	Participants (M, F)	Setting	Age, years*	BW, kg* (Baseline)	BMI, kg/m²* (Baseline)	IHCL (units)* (Baseline)	ALT (U/L)* (Baseline)	AST (U/L)* (Baseline)	Design	Feeding Control[a]	Randomization	Dose, g/d (% E)[b]	Interventions	Food matrix	Diet[c]	Energy Balance[d]	Follow-up	Funding[e]
**Substitution Trials (Isocaloric Comparison)**																		
**SSB**																		
**Aeberli et al. 2011**	29 NW (29M, 0F)	OP, Switzerland	26.3 (6.6)	73.7 (8.8)	22.4 (1.9)	-	23.0 (7.0)	26.0 (6.0)	C	Supp	Yes					Positive	3 wk	A, I
Intervention												40 (~7)	Fructose	600mL/d fructose SSB	~51:35:14			
Intervention												80 (~13)	Fructose	600mL/d fructose SSB	~55:32:14			
Intervention												80 (~13)	Sucrose	600mL/d sucrose SSB	~55:32:13			
Control													Glucose	600mL/d glucose SSB (40g)	~53:32:13			
Control													Glucose	600mL/d glucose SSB (80g)	~57:31:13			
**Alemán et al. 2021**	10 OB (6M, 4F)	IP, USA	57.6 (6.2)	101.8 (14.8)	35.9 (3.3)				C	Met	Yes				56:32:14	Neutral	2 wk	A
Intervention						-	21 (10)	19 (4)				75 (20.1)	Fructose	Fructose drink (75g/d)				
Control						-	25 (14)	20 (7)					Glucose	Glucose drink (75g/d)				
**Chiu et al. 2020**	30 OW/OB (30M, 0F)	OP, USA	15.3 (1.5)	86.8 (15.7)	1.8 (0.5)[f]	-	-	-	C	Supp	Yes				49:36:16	Neutral	3 wk	A, I
Intervention												80 (22)	HFCS	24oz/d SSB				
Control													Lactose	22oz/d energy-equivalent amount of 2% milk				
**Cox et al. 2012**	31 OW/OB (16M, 15F)	IP/OP, USA	52.5 (9.3)	29.3 (14.48)	29.3 (14.5)				P	Met/Supp	No				~55:30:15	Positive	10 wk	A
Intervention	16 OW (9M, 8F)					-	14.5 (1.6)	17.3 (1.6)				182 (25)	Fructose	Fructose beverage				
Control	15 OW (7M, 7F)					-	20.6 (3.2)	22.3 (2.3)					Glucose	Glucose beverage				
**Jin et al. 2014**	21 OW (11M, 10F)	OP, USA	13.5 (2.5)						P	Supp	Yes				NR	Neutral	4 wk	A
Intervention	9 OW (3M, 6F)		13.0 (2.6)	82.3 (16.9)	2.3 (0.6)[f]	14.5 (1.79)%	33.0 (6.7)	32.4 (3.1)				99 (~20)	Fructose	Fructose SSB				
Control	12 OW (8M, 4F)		14.2 (2.5)	82.0 (14.8)	2.2 (0.3)[f]	14.0 (1.77)%	32.7 (5.2)	33.8 (2.1)					Glucose	Glucose SSB				
**Johnston et al. 2013 (T1)**	32 OW (32M, 0F)	OP, UK	34 (9.9)	95.3 (5.7)		7.6 (5.3)%	28.9 (12.6)		P	Met	Yes				~55:30:15	Neutral	2 wk	A
Intervention	15 OW (15M, 0F)		35 (11)	96.8 (7.4)	30.0 (1.4)	7.2 (5.6)%	31.0 (15.0)	24.0 (8.0)				~221 (25)	Fructose	2000mL/d fructose dissolved in water				
Control	17 OW (17M, 0F)		33 (9)	93.9 (8.7)	28.9 (1.7)	8.0 (5.2)%	27.0 (10.0)	24.0 (5.0)					Glucose	2000mL/d glucose dissolved in water				
**Johnston et al. 2013 (T2)**	32 OW (32M, 0F)	OP, UK	34 (9.9)	95.26 (5.7)		7.6 (5.3)%	28.9 (12.6)		P	Supp	Yes				~55:30:15	Positive	2 wk	A
Intervention	15 OW (15M, 0F)		35 (11)	96.8 (7.4)	30.0 (1.4)	7.2 (5.6)%	31.0 (15.0)	24.0 (8.0)				~221 (25)	Fructose	2000mL/d SSB fructose dissolved in water				
Control	17 OW (17M, 0F)		33 (9)	93.9 (8.7)	28.9 (1.7)	8.0 (5.2)%	27.0 (10.0)	24.0 (5.0)					Glucose	2000mL/d SSB glucose dissolved in water				
**Maersk et al. 2012**	22 OW/OB (9M, 13F)	OP, Denmark	38 (8)	96.2 (13.8)	31.6 (2.8)				P	Supp	Yes				NR	Neutral	24 wk	A, I
Intervention	10 OW (6M, 4F)		39 (6)	97.8 (12.5)	31.3 (2.9)	0.037 (0.04)AU	-	-				~106 (~21)	Sucrose	Sucrose cola				
Control	12 OW (3M, 9F)		38 (9)	94.7 (15.3)	31.9 (2.8)	0.1 (0.1)AU	-	-					Lactose	Semi-skim milk				
**Ngo Sock et al. 2010**	11 NW (11M, 0F)	OP, Switzerland	24.6 (2.0)	71.9 (5.3)	(19-25)	-	-	-	C	Met	Yes				55:30:15	Positive	1 wk	A
Intervention												~214 (35)	Fructose	20% fructose solution				
Control													Glucose	20% glucose solution				
**Schwarz et al. 2015**	7 MW (7M, 0F)	IP, USA	42 (8.5)	-	24.4 (4.5)	-	-	-	C	Met	No				50:35:15	Neutral	9 d	A
Intervention												~112.5 (22.5)	Fructose	Fructose SSB				
Control													Starch	Isocaloric exchange of starch-containing foods				
**Silbernagel et al. 2011**	20 MW (12M, 8F)	OP, Germany	30.5 (8.9)	80.5 (4.2)	25.9 (2.3)	1.45 (0.85)%			P	Supp	Yes				50:35:15	Positive	4 wk	A
Intervention	10 MW (7M, 3F)		32.8 (9.3)	80.3 (9.1)	25.5 (2.2)	1.32 (0.29)%	-	-				150 (~22.1)	Fructose	High fructose diet				
Control	10 MW (5M, 5F)		28.2 (8.4)	80.7 (7.5)	26.2 (2.4)	1.59 (0.26)%	-	-					Glucose	High glucose diet				
**Sweetened dairy alternative (soy)**																		
**Eslami et al. 2019**	64 OW/OB (19M, 45F)	OP, Iran	45.7 (10.1)						P	Supp	Yes				55:30:15	Negative	8 wk	A, I
Intervention	32 OW/OB (10M, 22F)		46.3 (10.5)	83.8 (9.8)	30.9 (3.6)	-	41.1 (18.6)	30.8 (11.1)				5 (1)	Sucrose	240mL/d sweetened soy beverage				
Control	32 OW/OB (9M, 23F)		45.2 (9.9)	84.5 (14.0)	31.4 (3.7)	-	42.5 (17.5)	32.3 (14.9)					Mixed comparator	One serving of grains/starches and fats/oils food groups				

Study, year	Participants (M, F)	Setting	Age, years*	BW, kg*	BMI, kg/m^2*	IHCL (units)*	ALT (U/L)*	AST (U/L)*	Design	Feeding Control[a]	Randomization	Dose, g/d (% E)[b]	Interventions	Food matrix	Diet[c]	Energy Balance[d]	Follow-up	Funding[e]
				Baseline														
**100% Fruit juice**																		
**Ponce et al. 2019**	72 MetS (23 M, 49 F)	OP, Brazil	48 (9)	95 (16)	34.6 (4.1)				P	Supp	Yes					Neutral	12 wk	A, I
Intervention	36 MetS (12 M, 24 F)		49 (9)	96 (16)	34.0 (4.2)	-	34 (12)	22 (7)				44 (~12.2)	Fruit	500mL/d 100% orange juice	49:27:24			
Control	36 MetS (11 M, 25 F)		46 (9)	95 (15)	35.1 (4.1)	-	34 (12)	22 (9)					Fat	Energy equivalent nuts	48:28:24			
**Ribeiro et al. 2017**	78 OB (24M, 54F)	OP, Brazil	36 (1.0)	97.5 (12.0)	33.0 (3.0)				P	Supp	Yes				~60:35:15	Negative	12 wk	A, I
Intervention	39 OB		27 (1.0)	97.0 (12.0)	33.0 (3.0)	-	22 (8)	21 (9)				44 (~8.8)	Fruit juice	500mL/d orange juice				
Control	39 OB		33 (1.0)	98.0 (12.0)	35.0 (4.0)	-	20 (9)	21 (7)					Mixed comparator	Energy equivalent food item				
**Fruit**																		
**Agebratt et al. 2016**	30 MW (18M, 12F)	OP, Sweden	23.5 (3.7)		22.3 (1.9)				P	DA	Yes				NR	Positive	8 wk	A
Intervention	15 MW (7M, 8F)			66.5 (8.7)	22.2 (1.6)	2.11 (0.75)%	-	-				96 (~14.6)	Fruit	7kcal/kg BW/d (9.58g fruit)				
Control	15 MW (11M, 4F)			73.6 (9.0)	22.5 (2.3)	2.09 (0.68)%	-	-					Fat	7kcal/kg BW/d walnuts				
**Lehtonen et al. 2011 (BB)**	80 OW/OB (0M, 80F)	OP, Finland	44.2 (6.2)	81.6 (8.5)	29.6 (2.1)	-	21.0 (9.1)	-	C	Supp	Yes				NR	Neutral	33-35 d	A, I
Intervention												8.4 (1.7)[g]	Fruit	100g/d bilberries				
Control													Mixed comparator	Regular diet				
**Dried fruit**																		
**Kaliora et al. 2016**	44 NAFLD	OP, Greece							P	Supp	Yes				50:30:20	Negative	24 wk	A, I
Intervention	23 NAFLD		50.7 (10.9)[h]	85.7 (14.3)	29.5 (4.3)	-	30.0 (14.0)	22.8 (5.8)				24.2 (~5.0)[i]	Dried fruit	36g/d Corinthian currants				
Control	21 NAFLD		51.6 (9.4)[h]	82.0 (3.0)	28.2 (3.7)	-	29.5 (13.7)	23.1 (6.4)					Mixed comparator	Snacks (low fat yogurt, mini crackers, or bread with low fat cheese)				
**Kanellos et al. 2017**	33 NW	OP, Greece							P	Supp	Yes					Neutral	4 wk	A, I
Intervention	20 NW		30.8 (7.5)	77.5 (13.8)	24.7 (2.7)	-	19.2 (7.6)	21.1 (5.4)				~60 (~12)[i]	Dried fruit	90g/d raisins	41:27:13			
Control	13 NW		29.8 (5.0)	78.1 (14.1)	23.9 (3.2)	-	25.7 (15.5)	27.5 (14.1)					Mixed comparator	Snacks (low fat yogurt, mini crackers, or bread with low fat cheese)	43:33:17			
**Lehtonen et al. 2011 (SB)**	80 OW/OB (0M, 80F)	OP, Finland	44.2 (6.2)	81.6 (8.5)	29.6 (2.1)	-	21.0 (9.1)	-	C	Supp	Yes				NR	Neutral	33-35 d	A, I
Intervention												10.3 (2)[g]	Dried fruit	100g/d dried sea buckthorn berries				
Control													Mixed comparator	Regular diet				
**Added nutritive (caloric) sweetener**																		
**Simons et al. 2020**	37 OW/OB & fatty liver (M, F)	OP, Netherlands							P	Supp	Yes					Neutral	6 wk	A
Intervention	21 OW/OB & fatty liver (6M, 15F)		52 (38-62)	-	32.4 (4.3)	5.9 (6.5)%	-	-				~46.9 (9.4)	Fructose	3 sachets/d fructose mixed in water or food	~37:38:21			
Control	16 OW/OB & fatty liver (6M, 10F)		55 (35-62)	-	33.3 (6.9)	3.7 (5.4)%	-	-					Glucose	3 sachets/d glucose mixed in water or food	~39:38:20			
**Sweets and desserts**																		
**Claesson et al. 2009**	25 MW (11M, 14F)	OP, Sweden	23.4 (2.7)	68.0 (6.7)	22.2 (1.7)				P	Supp	Yes					Positive	2 wk	A
Intervention	12 MW (5M, 7F)		23.2 (3.5)	67.3 (7.6)	22.2 (1.4)	-	23.6 (6.6)	24.4 (3.5)				278 (~36.6)	Sucrose	20kcal/kg BW/d candy	~66:22:11			
Control	13 MW (6M, 7F)		23.6 (1.8)	68.7 (6.1)	22.2 (2.0)	-	20.3 (6.4)	25.3 (4.9)					Fat	20kcal/kg BW/d peanuts	~32:48:18			
**Dikariyanto et al. 2020**	IHL: 45 pre-CVD, ALT: 102 pre-CVD	OP, UK							P	Supp	Yes					Neutral	6 wk	A, I
Intervention	IHL: 22 pre-CVD, ALT: 49 pre-CVD		56.0 (10.7)[h]	-	26.7 (4.5)[h]	2.9 (4.0)%	22.0 (9.8)	-				15.1 (3.0)	Sucrose	20%E from muffins	~45:34:16			
Control	IHL: 23 pre-CVD, ALT: 53 pre-CVD		56.3 (10.3)[h]	-	27.3 (4.4)[h]	1.7 (2.3)%	22.1 (10.1)	-					Fat	20%E from almonds	~34:45:17			
**Kelsay et al. 1974**	8 MW (0M, 8F)	OP, USA	(18-23)	(43.6-65.3)	-	-			C	Met	Yes				50:38:12	Neutral	4 wk	NR
Intervention							5.0 (1.4)	8.6 (2.8)				212.5 (~42)	Sucrose	Sucrose patty				
Control							5.5 (1.7)	8.0 (0.8)					Glucose	Glucose patty				

Study, year	Participants (M, F)	Setting	Age, years*	Baseline					Design	Feeding Control[a]	Randomization	Dose, g/d (% E)[b]	Interventions	Food matrix	Diet[c]	Energy Balance[d]	Follow-up	Funding[e]
				BW, kg*	BMI, kg/m^2*	IHCL (units)*	ALT (U/L)*	AST (U/L)*										
**Mixed sources (with SSBs)**																		
**Lehtonen et al. 2010**	50 OW (0M, 50F)	OP, Finland	42.9 (35-52)	81.8	29.4				P	Supp	Yes					Neutral	20 wk	A, I
Intervention	28 OW (0M, 28F)			81.9	29.3 (2.2)	-	20.3 (5.5)	-				~14.7 (~3.3)[g]	Fruit	163g/d berry-containing products (fresh, dried, juice, bread, powder, oil); no SSBs	~50:32:17			
Control	22 OW (0M, 22F)			81.7	29.5 (1.8)	-	18.8 (7.6)	-					Mixed comparator	Snacks	~46:35:19			
**Luukkonen et al. 2018 (SAT)**	26 OW/OB (12M, 14F)	OP, Finland							P	Met	Yes					Positive	3 wk	A
Intervention	12 OW/OB (6M, 6F)		45 (10)	-	33 (6)	4.3 (4.7)%	24 (11)	26 (6)				~249.4 (~33.8)	Sugar	2.8dL orange juice, 4.3dL SSBs, and 200g candy	64:24:12			
Control	14 OW/OB (6M, 8F)		48 (8)	-	30 (6)	4.9 (6.6)%	28 (15)	26 (5)					Saturated fat	30g coconut oil, 40g butter, 100g of 40% fat containing blue cheese	26:59:15			
**Luukkonen et al. 2018 (UNSAT)**	24 OW/OB (11M, 13F)	OP, Finland							P	Met	Yes					Positive	3 wk	A
Intervention	12 OW/OB (6M, 6F)		45 (10)	-	33 (6)	4.3 (4.7)%	24 (11)	26 (6)				~249.4 (~33.8)	Sugar	2.8dL orange juice, 4.3dL SSBs, and 200g candy	64:24:12			
Control	12 OW/OB (5M, 7F)		52 (10)	-	31 (6)	4.8 (4.9)%	26 (9)	27 (7)					Unsaturated fat	36g olive oil, 26g pesto, 54g pecan nuts, and 20g butter	60:23:13			
**Nier et al. 2018**	13 NAFLD (7M, 5F)	OP, Germany	7.7 (0.8)	-	-				P	DA	No				~49:37:13	Neutral	52 wk	A
Intervention	6 NAFLD (3M, 3F)		7.5 (0.4)			-	26.0 (13.7)	38.0 (12.3)				69 (13)	Sucrose	Sucrose and fructose diet; includes SSBs				
Control	7 NAFLD (4M, 2F)		8.0 (0.3)			-	20.0 (2.7)	32.0 (7.9)				54 (10)	Starch	Reduce fructose intake to ~50% and replace with foods containing less fructose of the same food category				
**Parry et al. 2020**	16 OW/OB (16M, 0F)	OP, UK	47.9 (4.4)		27.7 (1.6)				C	Supp	Yes					Neutral	4 wk	A
Intervention				89.8 (10)		4.6 (3.6)%	10 (4)	-				~100 (~20)	Sucrose	High free-sugar diet; includes SSBs	65:20:15			
Control				89.3 (10.4)		4.4 (4)%	11 (4)	-					Fat	High saturated fat diet	40:45:15			
**Purkins et al. 2004**	12 MW (12M, 0F)	IP, UK	(20-41)			-	-	-	C	Met	Yes					Positive	8 d	NR
Intervention				76.6 (10.0)	-							352 (32)	Sucrose	High carbohydrate, high calorie diet	59:30:11			
Control				76.4 (10.2)	-							45 (4)	Fat	High fat, high calorie diet	29:58:13			
**Schwimmer et al. 2019**	40 NAFLD (40M, 0F)	OP, USA	13 (1.9)						P		Yes					Neutral	8 wk	A
Intervention	20 NAFLD (20M, 0F)		13.4 (1.9)	88.7 (26.3)	32.2 (6.3)	21 (8)%	72.5 (57.0-113.5)	39.0 (34.5-63.5)		DA		~88.2 (22)	Habitual diet	High sugar diet, ≥3 servings/wk or ≥8oz/wk juice or SSBs	49:33:19			
Control	20 NAFLD (20M, 0F)		12.8 (1.8)	88.1 (21.5)	33.7 (5.6)	25 (11)%	82.0 (57.0-144.0)	44.0 (32.0-79.0)		Met		~62.4 (15)	Mixed comparator	Free sugar intake restriction and substituted for low- to no-added-sugar food items	43:36:22			
**Umpleby et al. 2017 (H)**	14 OW/OB (14M, 0F)	OP, UK	54 (41-65)	89.7 (9.0)	28.4 (1.9)	2.5 (1.12)%	-	-	C	Supp	Yes					Neutral	12 wk	A
Intervention												126 (20.5)	Sucrose	High sugar diet with intake above 2.5th percentile of non-milk extrinsic sugars intake in the UK population	54:26:15			
Control												53 (6)	Starch	Low sugar diet with intake below 2.5th percentile of non-milk extrinsic sugars intake in the UK population	44:34:16			
**Umpleby et al. 2017 (NAFLD)**	11 NAFLD (11M, 0F)	OP, UK	59 (49-64)	90.0 (7.3)	28.9 (1.0)	17.2 (8.95)%	-	-	C	Supp	Yes					Neutral	12 wk	A
Intervention												126 (20.5)	Sucrose	High sugar diet with intake above 2.5th percentile of non-milk extrinsic sugars intake in the UK population	50:28:15			
Control												58 (6)	Starch	Low sugar diet with intake below 2.5th percentile of non-milk extrinsic sugars intake in the UK population	42:33:17			
**Vos et al. 2009**	10 NAFLD	OP, USA							P	DA	Yes					Neutral	24 wk	A
Intervention	4 NAFLD		12.5 (2)	-	-	-	103.3 (111.2)	65.3 (51.2)				31 (7)	Sucrose	Low-fat diet, based on the American Heart Association recommendations	51:31:19			
Control	6 NAFLD		13.3 (1.6)	-	-	-	125.5 (22.0)	71.2 (29.9)				23 (5.8)	Mixed comparator	Low-fructose diet; elimination of sugar-containing beverages, fruit juice, and food items containing high-fructose corn syrup	49:37:16			

Study, year	Participants (M, F)	Setting	Age, years*	Baseline BW, kg*	Baseline BMI, kg/m²*	Baseline IHCL (units)*	Baseline ALT (U/L)*	Baseline AST (U/L)*	Design	Feeding Control[a]	Randomization	Dose, g/d (% E)[b]	Interventions	Food matrix	Diet[c]	Energy Balance[d]	Follow-up	Funding[e]
**Addition Trials (Hypercaloric Comparison)**																		
**SSB**																		
**Aeberli et al. 2011**	29 NW (29M, 0F)	OP, Switzerland	26.3 (6.6)	73.7 (8.8)	22.4 (1.9)	-	23.0 (7.0)	26.0 (6.0)	C	Supp	Yes					Positive	3 wk	A, I
Intervention												40 (~7)	Fructose	600mL/d fructose SSB	~51:35:14			
Intervention												80 (~13)	Fructose	600mL/d fructose SSB	~55:32:14			
Intervention												80 (~13)	Sucrose	600mL/d sucrose SSB	~55:32:13			
Control													Diet alone	Reduce free fructose intake	~46:38:16			
**Debray et al. 2021 (Ctrl)**	6 OW/OB (2M, 4F)	OP, Belgium	40.93 (5.41)	72.78 (21.43)	24.93 (6.00)	-	-	-	C	Supp	Yes					Positive	1 wk	A
Intervention												103.8 (23.58)	Fructose	High fructose diet (1.4g/kg body weight/d in the form of SSBs)	50:31:18			
Control													Diet alone	Low fructose diet (<10g/d)	33:45:22			
**Debray et al. 2021 (hHFI)**	6 OW/OB (2M, 4F)	OP, Belgium	40.68 (6.12)	71.52 (17.69)	25.02 (5.34)	-	-	-	C	Supp	Yes					Positive	1 wk	A
Intervention												101.58 (~20.18)	Fructose	High fructose diet (1.4g/kg body weight/d in the form of SSBs)	50:35:15			
Control													Diet alone	Low fructose diet (<10g/d)	36:43:22			
**Johnston et al. 2013**	32 OW (32M, 0F)	OP, UK	34 (9.9)			7.6 (5.3)%	28.9 (12.6)	.	P	Met/Supp	No				~55:30:15	Positive	2 wk	A
Intervention	15 OW (15M, 0F)		35 (11)	96.8 (7.4)	30.0 (1.4)	7.2 (5.6)%						~221 (25)	Fructose	Fructose dissolved in water				
Control	17 OW (17M, 0F)		33 (9)	93.9 (8.7)	28.9 (1.7)	8.0 (5.2)%							Glucose	Glucose dissolved in water				
**Koopman et al. 2014 (HS-F)**	13 NW (13M, 0F)	OP, Netherlands							P	Supp	Yes					Positive	6 wk	A
Intervention	8 NW (8M, 0F)		21.9 (2.8)	81.0 (8.8)	22.6 (1.8)	0.8 (0.5)%	-	-				~237 (27)	Sucrose	3 servings/d sucrose-sweetened SSB	~56:29:12			
Control	5 NW (5M, 0F)		23.0 (3.1)	76.6 (7.7)	22.6 (2.3)	1.3 (0.5) %							Diet alone	No beverage	NR			
**Koopman et al. 2014 (HS-S)**	12 NW (12M, 0F)	OP, Netherlands							P	Supp	Yes					Positive	6 wk	A
Intervention	7 NW (7M, 0F)		22 (2.5)	77.4 (7.9)	21.7 (1.1)	1.5 (1.0)%	-	-				~237 (27)	Sucrose	3 servings/d sucrose-sweetened SSB	~58:27:12			
Control	5 NW (5M, 0F)		23.0 (3.1)	76.6 (7.7)	22.6 (2.3)	1.3 (0.5)%							Diet alone	No beverage	NR			
**Lê et al. 2006**	7 NW (7M, 0F)	OP, Switzerland	24.7 (3.4)	69.3 (6.9)	(19-25)	6.21 (2.09) mmol/kg	-	-	C	Supp	No				55:30:15	Positive		A
Intervention												104 (~18)	Fructose	20% fructose solution			4 wk	
Control													Diet alone	No beverage			2 wk	
**Lê et al. 2009 (H)**	8 NW (8M, 0F)	OP, Switzerland	24 (2.8)	-	-	-	16.9 (1.2)	-	C	Met	Yes				55:30:15	Positive	1 wk	A, I
Intervention												~220 (35)	Fructose	20% fructose solution				
Control													Diet alone	No beverage				
**Lê et al. 2009 (ODM2)**	16 ODM2 (16M, 0F)	OP, Switzerland	24.7 (5.2)	-	-	-	16.4 (4)	-	C	Met	Yes				55:30:15	Positive	1 wk	A, I
Intervention												~220 (35)	Fructose	20% fructose solution				
Control													Diet alone	No beverage				
**Maersk et al. 2012**	35 OW/OB (14M, 21F)	OP, Denmark							P	Supp	Yes				NR	Positive	24 wk	A, I
Intervention	10 OW/OB (6M, 4F)		39 (6)	97.8 (39.5)	31.3 (2.9)	0.04 (0.04)AU	-	-				106 (~21.2)	Sucrose	Sucrose cola				
Control	12 OW/OB (3M, 9F)		39 (8)	92.2 (10.9)	32.8 (3.8)	0.2 (0.2)AU	-	-					NNS	Diet cola (no sucrose)				
Control	13 OW/OB (5M, 8F)		39 (8)	101.7 (22.4)	32.2 (4.6)	0.1 (0.1)AU	-	-					Water	Water				
**Ngo Sock et al. 2010**	11 NW (11M, 0F)	OP, Switzerland	24.6 (2.0)	-	-	2.4 (0.8)log mmol/kg	26.0 (13.3)	.	C	Met	Yes				55:30:15	Positive	1 wk	A
Intervention												~214 (35)	Fructose	SSB				
Control													Diet alone	No beverage				
**Sigala et al. 2021**	75 MW (38M, 37F)	IP/OP, USA							P	Met + Supp	No				55:30:15	Positive	2 wk	A
Intervention	28 MW (15M, 13F)		26.8 (6.6)	72.9 (14.5)	24.9 (4.0)	2.3 (0.8)%	-	-				(25)	HFCS	HFCS as 25%E in Kool-Aid				
Intervention	24 MW (12M, 12F)		25.9 (6.3)	71.9 (12.1)	25.3 (3.4)	1.6 (0.8)%	-	-				(25)	Sucrose	Sucrose as 25%E in Kool-Aid				
Control	23 MW (11M, 12F)		25.4 (6.2)	71.8 (10.6)	24.8 (3.3)	1.9 (0.4)%	-	-					NNS	Aspartame in Market Pantry drink mix				

Study, year	Participants (M, F)	Setting	Age, years*	Baseline<br>BW, kg*	BMI, kg/m^2*	IHCL (units)*	ALT (U/L)*	AST (U/L)*	Design	Feeding Control[a]	Randomization	Dose, g/d (% E)[b]	Interventions	Food matrix	Diet[c]	Energy Balance[d]	Follow-up	Funding[e]
**SSB**																		
**Silbernagel et al. 2011**	10 MW (7M, 3F)	OP, Germany	32.8 (9.3)	80.3 (9.1)	25.5 (2.2)	1.5 (0.9)%	-	-	C	Supp	No				50:35:15	Positive		A
Intervention												150 (~22)	Fructose	Fructose dissolved in water			4 wk	
Control													Diet alone	No beverage			2 wk	
**Sobrecases et al. 2010**	12 NW (12M, 0F)	OP, Switzerland	23.9 (2.2)	-	22.6 (0.2)	-	-	-	C	Supp	No				55:30:15	Positive	1 wk	A
Intervention												~214 (35)	Fructose	SSB				
Control													Diet alone	No beverage				
**Zafrilla et al. 2021**	136 OW (80M, 56F)	OP, Spain							P	Supp	Yes			Citrus juice and maqui extract with different sweeteners added	NR	Positive	60 d	A
Intervention	45 OW (26 M, 19 F)		42 (7)	85 (12)	29 (3)	-	25 (7)	26 (7)				24.75 (5)	Sucrose	7.5g/100mL and consumed 330 mL/day				
Control	46 OW (27 M, 19 F)		44 (7)	83 (11)	28 (3)	-	22 (7)	26 (6)					Stevia	4mg/100mL and consumed 330 mL/day				
Control	45 OW (27 M, 18 F)		42 (8)	82 (11)	28 (2)	-	20 (7)	24 (6)					Sucralose	4mg/100mL and consumed 330 mL/day				
**100% Fruit juice**																		
**Amagase et al. 2009**	60 MW	OP, China	58.9 (55-72)		-				P	Supp	Yes				NR	Positive	30 d	I
Intervention	30 MW		58.7 (5.3)	61.8 (9.7)		-	23.6 (15.2)	25.9 (6.4)				~12 (2.4)	Fruit juice	120mL/d GoChi goji berry fruit juice, equivalent to 150g fresh fruit				
Control	30 MW		59.1 (5.6)	60.9 (9.5)		-	23.6 (10.5)	26.5 (7.6)					NNS	Sucralose and artificially flavored beverage				
**Banini et al. 2006**	23 OW/OB (11M, 12F)	OP, USA							P	Supp	Yes				~50:31:19	Positive	28 d	A, I
Intervention	8 OW/OB (3M, 5F)		50 (13)	-	29.3 (4.0)	-	28 (3.7)	15.5 (1.7)				22 (4)[j]	Fruit juice	150mL/d muscadine grape juice				
Control	15 OW/OB (8M, 7F)		56 (7.5)	-	27.5 (5.4)	-	27.4 (4.4)	19.5 (2.9)					Diet alone	No juice				
**Kojadinovic et al. 2017**	23 Dyslipidemia (0M, 23F)	OP, Serbia	(40-60)						P	Supp	Yes				NR	Positive	6 wk	A
Intervention	12 Dyslipidemia (0 M, 12 F)			-	31.98 (3.57)	-	32.8 (15.6)	23.4 (5.12)				37 (7.8)	Fruit	300 mL/d Polyphenol-rich pomegranate juice				
Control	11 Dyslipidemia (0 M, 11 F)			-	27.83 (7.84)	-	26.7 (12.4)	24.9 (7.5)					Water	300 mL/d water				
**Ravn-Haren et al. 2013**	23 MW (9M, 14F)	OP, Denmark	36.2 (17.9)	-	22.3 (2.6)	-	19.8 (9.0)	-	C	Supp	Yes				NR	Positive	4 wk	A
Intervention												63 (~12.6)[k]	Fruit	500mL/d polyphenolic and pectin restricted diet with cloudy apple juice				
Intervention												51 (~11.8)[k]	Fruit	500mL/d polyphenolic and pectin restricted diet with clear apple juice				
Control													Diet alone	Polyphenolic and pectin restricted diet				
Control													Diet alone	Polyphenolic and pectin restricted diet with apple pomace				
**Fruit**																		
**Ravn-Haren et al. 2013**	23 MW (9M, 14F)	OP, Denmark	36.2 (17.9)	-	22.3 (2.6)	-	19.8 (9.0)	-	C	Supp	Yes	~51 (~10.2)[k]			NR	Positive	4 wk	A
Intervention													Fruit	Polyphenolic and pectin restricted diet with whole apples equivalent to ~550g/d				
Control													Diet alone	Polyphenolic and pectin restricted diet				
Control													Diet alone	Polyphenolic and pectin restricted diet with apple pomace				
**Schell et al. 2019**	22 DM2 (5M, 20F)	OP, USA	54 (21)	104 (55)	35.3 (10)	-	45.3 (20.2)	33.2 (10.3)	C	Supp	Yes					Positive	4 wk	A, I
Intervention												11.1 (2.2)[i]	Fruit	250g/d frozen raspberries	48:34:17			
Control													No fruit	No raspberries	45:37:16			
**Tutino et al. 2021**	40 OW/OB (11M, 29F)	OP, Italy							P	Supp	Yes				NR	Positive	3wk	A
Intervention	21 OW/OB (6 M, 15 F)		47.4 (9.5)	67.5 (13.0)	25.3 (4.0)	-	18.6 (7.3)	18.7 (3.5)				~57.1 (~11.4)	Fruit	5g/kg BW/d, ~350g/d black seedless grapes (Autumn Royal)				
Control	19 OW/OB (5 M, 14 F)		44.1 (10.1)	78.0 (18.0)	27.9 (4.4)	-	22.5 (10.6)	18.9 (4.3)					Diet alone	No grapes				

Study, year	Participants (M, F)	Setting	Age, years*	Baseline BW, kg*	Baseline BMI, kg/m²*	Baseline IHCL (units)*	Baseline ALT (U/L)*	Baseline AST (U/L)*	Design	Feeding Control[a]	Randomization	Dose, g/d (% E)[b]	Interventions	Food matrix	Diet[c]	Energy Balance[d]	Follow-up	Funding[e]
**Dried fruit**																		
**Ahmed et al. 2010**	70 MW	OP, Pakistan	43 (10)	-	-				P	Supp	Yes				NR	Positive	8 wk	A
Intervention	35 MW					-	21.8 (1.7)	23.7 (1.3)				~8.8 (1.8)[i]	Dried fruit	6 dried prunes (22.86g/d), soaked in water overnight and eaten along with water mixture in morning				
Control	35 MW					-	23.2 (1.7)	27.7 (2.5)					Water	Water				
**Irranejad et al. 2020**	72 DM2 (21M, 51F)	OP, Iran							P	Supp	Yes					Positive	12 wk	A
Intervention	36 DM2 (8M, 28F)		53.2 (7.8)	76.1 (13.3)	29.1 (4.1)	-	30.0 (18.2)	23.5 (12.4)				~8 (2.1)[j]	Dried fruit	30g/d dried *Ziziphus vulgaris*	71:20:16			
Control	36 DM2 (13M, 23F)		56.6 (6.0)	74.6 (11.0)	28.1 (4.1)	-	25.9 (13.5)	20.6 (7.1)					Diet alone	No *Ziziphus vulgaris*	69:18:15			
**Added nutritive (caloric) sweetener**																		
**Bahrami et al. 2009**	48 DM2 (13M, 35F)	OP, Iran	57.2 (8.4)	70.8 (10.6)					P	Supp	Yes					Positive	8 wk	A
Intervention	25 DM2			71.3 (12.7)	-	-	23.2 (6.4)	22.7 (9.5)				~125 (~33)	Honey	Natural unprocessed honey	64:23:15			
Control	23 DM2			70.3 (8.1)	-	-	24.9 (8.3)	24.0 (9.4)					Diet alone	No honey	60:22:15			
**Tang et al. 2020**	95 HIV (81M, 14F)	IP, Malaysia	(21-58)						P	Met	Yes				NR	Positive	6 mo	A
Intervention	26 HIV			56.4 (7.8)	21.5 (2.6)	-	33.9 (24.9)	29.4 (9.9)				16.4 (3.2)[i]	Honey	20g/d Tualang honey				
Intervention	24 HIV			58.8 (8.3)	22.2 (3.1)	-	37.9 (50.2)	33.8 (30.2)				32.8 (6.2)[i]	Honey	40g/d Tualang honey				
Intervention	22 HIV			54.9 (6.8)	20.8 (2.4)	-	28.0 (24.1)	32.1 (23.5)				49.3 (9.0)[i]	Honey	60g/d Tualang honey				
Control	23 HIV			58.3 (7.1)	22.7 (2.7)	-	39.6 (48.2)	40.0 (43.8)					Diet alone	No honey				
**Zakaria et al. 2018**	72 Breast cancer (0M, 72F)	OP, Malaysia							P	Supp	Yes				NR	Positive	12 wk	A
Intervention	36 Breast cancer (0M, 36F)		56.5 (1.0)	-	-	-	29.4 (2.5)	-				16.4 (3.3)[i]	Added sweetener	20g/d Tualang honey				
Control	36 Breast cancer (0M, 36F)		59.6 (1.3)	-	-	-	25.3 (2.0)	-					Diet alone	No honey				
**Sweets and desserts**																		
**Alavinejad et al. 2015**	42 NAFLD (33M, 9F)	OP, Iran							P	Supp	Yes				NR	Positive	12 wk	A, I
Intervention	21 NAFLD (15M, 6F)		38.0 (10.3)	88.6 (13.2)	30.3 (3.6)	-	54.2 (41.3)	39.0 (18.7)				~6.1 (1.2)[i]	Dark chocolate	30g/d dark chocolate (83% cocoa)				
Control	21 NAFLD (18M, 3F)		38.2 (11.0)	84.9 (20.6)	29.7 (5.8)	-	39.6 (19.3)	31.0 (11.2)					Fat	30g/d sugar-free white chocolate				
**Subtraction Trials (Hypocaloric Comparisons)**																		
**SSB**																		
**Campos et al. 2015 (G1)**	15 OW/OB (11M, 4F)	OP, Switzerland	29.1 (6.9)						P	Supp	Yes					Negative	12 wk	A
Intervention	8 OW/OB			102.2 (11.4)	32.5 (4.5)	89.7 (31.1)mmol/L	38.6 (26.7)	28.4 (8.8)					NNS	Replace SSB with NSB	~46:38:16			
Control	7 OW/OB			100.0 (12.4)	33.8 (5.6)	189.7 (110.3)mmol/L	41 (21.2)	30.1 (5.8)				86.8 (~15)	Sucrose	Habitual SSB consumption (>2 SSB/d)	~51:34:15			
**Campos et al. 2015 (G2)**	12 OW/OB (3M, 9F)	OP, Switzerland	28.3 (6.5)						P	Supp	Yes					Negative	12 wk	A
Intervention	6 OW/OB			85.6 (11.3)	30.1 (4.9)	17.5 (12.7)mmol/L	17.3 (4.7)	23.3 (13.7)					NNS	Replace SSB with NSB	~46:38:16			
Control	6 OW/OB			78.5 (7.1)	26.9 (1.2)	13.4 (7.1)mmol/L	17.3 (8.1)	19.8 (4.7)				86.8 (~15)	Sucrose	Habitual SSB consumption (>2 SSB/d)	~51:34:15			

				Baseline														
Study, year	Participants (M, F)	Setting	Age, years*	BW, kg*	BMI, kg/m^2*	IHCL (units)*	ALT (U/L)*	AST (U/L)*	Design	Feeding Control[a]	Randomization	Dose, g/d (% E)[b]	Interventions	Food matrix	Diet[c]	Energy Balance[d]	Follow-up	Funding[e]
**Mixed sources (with SSBs)**																		
**Porikos et al. 1983 (NonOB)**	11 MW (11M, 0F)	IP, USA	(24-45)	-	-	-	-	-	C	Met	No					Negative (*ad libitum*)	12 d	A, I
Intervention													NNS	Aspartame-sweetened foods	~43:41:16			
Control												~194 (23.5)[k]	Sucrose	High sucrose diet (includes SSBs)	~56:32:12			
**Porikos et al. 1983 (OB)**	5 OW/OB (5M, 0F)	IP, USA	(24-45)	-	-	-	-	-	C	Met	No					Negative (*ad libitum*)	12 d	A, I
Intervention													NNS	Aspartame-sweetened foods	~46:39:15			
Control												~183 (18.5)[k]	Sucrose	High sucrose diet (includes SSBs)	~56:32:12			
**Ad Libitum Trials (Free-Feeding Comparisons)**																		
**Mixed sources (with SSBs)**																		
**Mäkinen et al. 1976 (F)**	92 MW (30M, 62F)	OP, Finland	27.7 (7.2)						P	Supp	Partial[l]				NR	Neutral	22 mo	NR
Intervention	38 MW (12M, 26F)		27.4 (4.4)	65.0 (15.4)	21.4 (6.1)	-	-	-				70 (14)	Fructose	Ad libitum fructose-containg foods				
Control	54 MW (18, 36F)		30.6 (9.9)	66.8 (14.3)	21.5 (7.1)	-	-	-					Sweetener	Ad libitum xylitol-containing foods, with avoidance to sweet fruits (dried figs, raisins and dates)				
**Mäkinen et al. 1976 (S)**	89 MW (31M, 58F)	OP, Finland	27.7 (7.2)						P	Supp	Partial[l]				NR	Neutral	22 mo	NR
Intervention	35 MW (13M, 22F)		27.7 (5.8)	67.3 (13.2)	23.0 (3.8)	-	-	-				73 (14.7)	Sucrose	Ad libitum sucrrose-containg foods				
Control	54 MW (18M, 36F)		30.6 (9.9)	66.8 (14.3)	21.5 (7.1)	-	-	-					Sweetener	Ad libitum xylitol-containing foods, with avoidance to sweet fruits (dried figs, raisins and dates)				

*All values presented as mean ± SD, unless otherwise indicated.

[a] Metabolic feeding control included provision of all study foods, supplement feeding control included provision of study supplements only, and dietary advice included dietary counseling without the provision of any dietary foods or supplements.

[b] Doses preceded by "~" represent approximate amounts calculated on the basis of average body weight or energy intake reported by participants. In the absence of this data, an average of 70 kg body weight or 2000 kcal/d was assumed.

[c] Total energy intake in the form of carbohydrate:fat:protein.

[d] Positive energy balance included interventions designed to consume excess calories on top of a baseline diet. Negative energy balance included interventions designed to create a caloric deficit compared to the baseline diet. Neutral energy balance included interventions designed to continue habitual caloric intake.

[e] Agency funding included government, not-for profit health agencies or University sources.

[f] Z-scores reported for pediatric studies.

[g] Fructose-containing sugar dose estimated based on data from the Finland National Food Composition Database.

[h] Data based on baseline participants, including dropouts.

[i] Fructose-containing sugar dose estimated based on data from the Canadian Nutrient File.

[j] Fructose-containing sugar dose estimated based on data from United States Department of Agriculture (USDA) nutrient database.

[k] Fructose-containing sugar dose estimated from total daily caloric intake and sugar intake from study products.

[l] Half of the participants were assigned to groups according to personal preference, while the other half of the participants were randomly allocated.

%C=percent carbohydrate; %E=percentage of total energy intake; %F=percent fat; %P=percent protein; A=agency; AI=agency-industry; ALT=alanine aminotransferase; AST=aspartate aminotransferase; BB=bilberries; BW=body weight; C=crossover; CHO=carbohydrate; Cld=cloudy apple juice; Clr=clear apple juice; Ctrl=control; d=day; DA=dietary advice; DM2=type-2 diabetes mellitus; e=energy; F=female; g=grams; G1=group one; G2=group two; H=healthy;

HF=high fructose; hHFI=heterozygote high fructose intolerance; HS=high sucrose; HS-F=high sucrose frequency; HS-S=high sucrose size; I=industry; IHL=intrahepatic lipid; IHCL=intrahepatocellular lipid; kg=kilogram; LF=low fructose; M=men; Met=metabolically controlled; MF=medium fructose; NAFLD=non-alcoholic fatty liver disease; NNS=non-nutritive sweetener; NonOB=non-obese; NSB=non-nutritively-sweetened beverage; NR=not reported; OB=obese; ODM2=offspring of type-2 diabetes mellitus patients; OW=overweight; P=parallel; Pom=apple pomace; SAT=saturated fat; SB=sea buckthorn berries; SD=standard deviation; SSB=sugar-sweetened beverage; Supp=supplemented; T1=treatment group 1; T2=treatment group 2; UNSAT=unsaturated fat; W=women; wk=week.

Supplementary Table S6: Sensitivity analyses of the use of correlation coefficients of 0.25 and 0.75 for crossover trials in the primary analysis of the effect of important food sources of fructose-containing sugars and NAFLD outcomes*

NAFLD Outcome	MD [95% CI], P_{MD}<br>I^2, P_Q		
	Correlation Coefficient used in the Primary Analysis	Correlation Coefficient used in Sensitivity Analyses	
**Energy Design and Food Source (N crossover trials/total)**	**0.5**	**0.25**	**0.75**
**IHCL (SMD)**			
**Substitution (5/16)***	**0.36 [-0.07, 0.79], p_{MD}=0.098<br>I^2=6.70%, p_Q=0.377**	**0.35 [-0.07, 0.76], p_{MD}=0.101<br>I^2=1.37%, p_Q=0.437**	**0.39 [-0.07, 0.86], p_{MD}=0.097<br>I^2=15.87%, p_Q=0.272**
**Addition (6/13)****	**1.72 [1.08, 2.36], p_{MD}<0.001<br>I^2=0.00%, p_Q=0.943**	**1.66 [1.04, 2.29], p_{MD}<0.001<br>I^2=0.00%, p_Q=0.960**	**1.79 [1.12, 2.45], p_{MD}<0.001<br>I^2=0.00%, p_Q=0.893**
SSB (6/13)**	1.72 [1.08, 2.36], p_{MD}<0.001<br>I^2=0.00%, p_Q=0.943	1.66 [1.04, 2.29], p_{MD}<0.001<br>I^2=0.00%, p_Q=0.960	1.79 [1.12, 2.45], p_{MD}<0.001<br>I^2=0.00%, p_Q=0.893
**Subtraction (0/2)**	**-0.52 [-1.60, 0.56], p_{MD}=0.345<br>I^2=0.00%, p_Q=0.470**	**NA**	**NA**
SSB (0/2)	-0.52 [-1.60, 0.56], p_{MD}=0.345<br>I^2=0.00%, p_Q=0.470	NA	NA
**ALT (U/L)**			
**Substitution (11/28)**	**-0.37 [-1.71, 0.97], p_{MD}=0.589<br>I^2=52.64%, p_Q=0.001**	**-0.34 [-1.73, 1.05], p_{MD}=0.634<br>I^2=51.34%, p_Q=0.001**	**-0.51 [-1.77, 0.75], p_{MD}=0.430<br>I^2=54.51%, p_Q<0.001**
**Addition (16/31)**	**0.91 [-0.39, 2.12], p_{MD}=0.169<br>I^2=31.44%, p_Q=0.050**	**0.96 [-0.38, 2.31], p_{MD}=0.161<br>I^2=22.88%, p_Q=0.128**	**0.83 [-0.38, 2.04], p_{MD}=0.177<br>I^2=44.19%, p_Q=0.005**
SSB (9/12)	3.09 [0.49, 5.68], p_{MD}=0.020<br>I^2=58.18%, p_Q=0.006	3.22 [0.58, 5.87], p_{MD}=0.017<br>I^2=50.97%, p_Q=0.021	2.91 [0.39, 5.42], p_{MD}=0.023<br>I^2=68.05%, p_Q<0.001
100% Fruit juice (4/7)	-0.80 [-2.43, 0.84], p_{MD}=0.340<br>I^2=0.00%, p_Q=0.949	-0.84 [-2.78, 1.11], p_{MD}=0.399<br>I^2=0.00%, p_Q=0.960	-0.75 [-1.96, 0.46], p_{MD}=0.224<br>I^2=0.00%, p_Q=0.908
Fruit (3/4)	0.44 [-2.22, 3.10], p_{MD}=0.746<br>I^2=0.00%, p_Q=0.935	0.43 [-2.66, 3.53], p_{MD}=0.784<br>I^2=0.00%, p_Q=0.962	0.43 [-1.60, 2.46], p_{MD}=0.678<br>I^2=0.00%, p_Q=0.849
Dried fruit (0/2)	-2.58 [-17.46, 12.31], p_{MD}=0.735<br>I^2=0.00%, p_Q=0.950	NA	NA
Honey (0/5)	-0.87 [-5.33, 5.60], p_{MD}=0.703<br>I^2=15.28%, p_Q=0.317	NA	NA
**Subtraction (2/4)**	**-4.86 [-15.91, 6.19], p_{MD}=0.388<br>I^2=38.84%, p_Q=0.179**	**-3.76 [-14.37, 6.85], p_{MD}=0.487<br>I^2=30.20%, p_Q=0.231**	**-6.01 [-17.24, 5.22], p_{MD}=0.294<br>I^2=49.18%, p_Q=0.116**
SSB (0/2)	1.33 [-7.55, 10.22], p_{MD}=0.769<br>I^2=15.31%, p_Q=0.277	NA	NA
Mixed sources (with SSBs) (2/2)	-15.68 [-32.90, 1.54], p_{MD}=0.074<br>I^2=0.00%, p_Q=0.645	-16.05 [-35.51, 3.40], p_{MD}=0.106<br>I^2=0.00%, p_Q=0.688	-39.15 [-58.89, -19.41], p_{MD}<0.001<br>I^2=60.39%, p_Q=0.112
***Ad libitum* (0/2)**	**1.02 [-0.87, 2.92], p_{MD}=0.290<br>I^2=0.00%, p_Q=0.509**	**NA**	**NA**
Mixed sources (0/2)	1.02 [-0.87, 2.92], p_{MD}=0.290<br>I^2=0.00%, p_Q=0.509	NA	NA
**AST (U/L)**			
**Substitution (8/23)**	**0.39 [-0.87, 1.65], p_{MD}=0.546<br>I^2=46.64%, p_Q=0.008**	**0.40 [-0.89, 1.68], p_{MD}=0.546<br>I^2=43.98%, p_Q=0.013**	**0.36 [-0.86, 1.57], p_{MD}=0.564<br>I^2=49.87%, p_Q=0.004**
**Addition (7/21)**	**-0.03 [-0.82, 0.76], p_{MD}=0.945<br>I^2=7.84%, p_Q=0.357**	**-0.03 [-0.68, 0.62], p_{MD}=0.925<br>I^2=0.07%, p_Q=0.457**	**-0.19 [-1.03, 0.66], p_{MD}=0.664<br>I^2=24.76%, p_Q=0.147**
SSB (6/9)	0.29 [-1.07, 1.64], p_{MD}=0.677<br>I^2=31.62%, p_Q=0.165	0.51 [-0.87, 1.88], p_{MD}=0.473<br>I^2=18.79%, p_Q=0.276	0.03 [-1.27, 1.34], p_{MD}=0.963<br>I^2=51.46%, p_Q=0.036
100% Fruit juice (0/3)	0.02 [-2.66, 2.71], p_{MD}=0.986<br>I^2=0.00%, p_Q=0.491	NA	NA
Fruit (1/2)	-1.60 [-4.92, 1.72], p_{MD}=0.346	-1.48 [-4.91, 1.95], p_{MD}=0.397	-1.85 [-4.91, 1.21], p_{MD}=0.236

	I^2=0.00%, p_Q=0.624	I^2=0.00%, p_Q=0.677	I^2=0.00%, p_Q=0.531
Dried fruit (0/2)	-5.24 [-14.40, 3.93], p_{MD}=0.263 I^2=0.00%, p_Q=0.606	NA	NA
Sweets and desserts (/01)	-6.08 [-14.50, 2.34], p_{MD}=0.157 I^2=., p_Q=.	NA	NA
Added nutritive (caloric) sweetener (0/4)	1.05 [-4.04, 6.15], p_{MD}=0.685 I^2=5.63%, p_Q=0.365	NA	NA
**Subtraction (2/4)**	**-5.18 [-8.60, -1.76], p_{MD}=0.003 I^2=15.10%, p_Q=0.316**	**-4.71 [-8.57, -0.85], p_{MD}=0.017 I^2=0.00%, p_Q=0.425**	**-5.88 [-8.62, -3.14], p_{MD}<0.001 I^2=43.46%, p_Q=0.151**
SSB (0/2)	-1.33 [-7.61, 4.96], p_{MD}=0.679 I^2=0.00%, p_Q=0.867	NA	NA
Mixed sources (with SSBs) (2/2)	-7.33 [-12.78, -1.87], p_{MD}=0.009 I^2=31.00%, p_Q=0.229	-6.76 [-11.65, -1.87], p_{MD}=0.007 I^2=0.00%, p_Q=0.322	-6.95 [-10.00, -3.90], p_{MD}<0.001 I^2=64.10%, p_Q=0.095
***Ad libitum* (0/2)**	**-0.45 [-1.26, 0.36], p_{MD}=0.278 I^2=0.00%, p_Q=0.716**	**NA**	**NA**
Mixed sources (with SSBs) (0/2)	-0.45 [-1.26, 0.36], p_{MD}=0.278 I^2=0.00%, p_Q=0.716	NA	NA

*Where there was a significant interaction by food source in addition trials and SSBs and mixed sources were the sole food sources in subtraction and ad libitum trials, sensitivity analysis was conducted for each food source. ALT=alanine aminotransferase; AST=aspartate aminotransferase; IHCL=intrahepatocellular lipid; CI=confidence interval; MD=mean difference; NA=not available; no.=number; SMD=standardized mean difference.

Supplementary Table S7: GRADE certainty of evidence assessment for the effect of fructose-containing sugars and NAFLD outcomes by levels of energy control

Outcome and trial (N)	Design	GRADE assessment						Effect (MD or SMD [95%CI], P_{MD})		Certainty of Evidence[a]	Interpretation of magnitude of effect[b]
		Downgrades					Upgrades				
		Risk of bias	Inconsistency	Indirectness	Imprecision	Publication bias	Dose response				
**IHCL (SMD)***											
Substitution (16)	Randomized and non-randomized trials	Not serious	Not serious	Not serious[1]	Serious[2]	None[3]	None	↔	SMD 0.36 [-0.07 to 0.79], p=0.098	⊕⊕⊕◯ Moderate	No effect
Addition (13)	Randomized and non-randomized trials	Not serious	Not serious	Very serious[4]	Not serious	None[5]	None	↑	SMD 1.72 [1.08 to 2.36], p<0.001	⊕⊕◯◯ Low	Large
Subtraction (2)	Randomized trials	Not serious	Not serious	Very serious[6]	Serious[7]	None[8]	N/A[9]	↔	SMD -0.52 [-1.60 to 0.56], p=0.345	⊕◯◯◯ Very low	No effect
**ALT (U/L)**											
Substitution (28)	Randomized and non-randomized trials	Not serious	Not serious[10]	Not serious	Not serious	None[11]	None	↔	MD -0.37U/L [-1.71 to 0.97], p=0.589	⊕⊕⊕⊕ High	No effect
Addition (31)	Randomized and non-randomized trials	Not serious	Not serious	Very serious[12]	Not serious	None	Linear DR, no upgrade[13]	↔	MD 0.91U/L [-0.39 to 2.21], p=0.169	⊕⊕◯◯ Low	No effect
Subtraction (4)	Randomized trials	Not serious	Not serious	Very serious[14]	Serious[15]	None[8]	N/A[9]	↔	MD -4.86U/L [-15.91 to 6.19], p=0.388	⊕◯◯◯ Very low	No effect
Ad libitum (2)	Non-randomized trials	Serious[16]	Not serious	Very serious[17]	Serious[18]	None[8]	N/A[9]	↔	MD 1.02U/L [-0.87 to 2.92], p=0.290	⊕◯◯◯ Very low	No effect
**AST (U/L)**											
Substitution (23)	Randomized and non-randomized trials	Not serious	Not serious	Not serious	Not serious	None[19]	None	↔	MD 0.39U/L [-0.87 to 1.65], p=0.546	⊕⊕⊕⊕ High	No effect
Addition (21)	Randomized and non-randomized trials	Not serious	Not serious	Veryserious[20]	Not serious	None	None	↔	MD -0.03U/L [-0.82 to 0.76], p=0.945	⊕⊕◯◯ Low	No effect
Subtraction (4)	Randomized trials	Not serious	Not serious	Very serious[21]	Serious[22]	None[8]	N/A[9]	↓	MD -5.18U/L [-8.60 to -1.76], p=0.003	⊕◯◯◯ Very low	Moderate
Ad libitum (2)	Non-randomized trials	Serious[23]	Not serious	Very serious[24]	Not serious	None[8]	N/A[9]	↔	MD -0.45U/L [-1.26 to 0.36], p=0.278	⊕◯◯◯ Very low	No effect

[a] Since all included trials were randomized or non-randomized controlled trials, the certainty of the evidence was graded as high for all outcomes by default and then downgraded or upgraded based on pre-specified criteria. Criteria for downgrades included risk of bias (downgraded if the majority of trials were considered to be at high risk of bias); inconsistency (downgraded if there was substantial unexplained heterogeneity [$I^2 \geq 50\%$, $p < 0.10$]; indirectness (downgraded if there were factors absent or present relating to the participants, interventions, or outcomes that limited the generalizability of the results); imprecision (downgraded if the 95% confidence interval crossed the minimally important difference [MID] for harm or benefit set at 0.26 for IHCL, 2.85U/L for ALT(2), and 2.55U/L for AST(2)); and publication bias (downgraded if there is evidence of publication bias based on funnel plot asymmetry and/or significant Egger's or Begg's tests (P<0.10) with confirmation by adjustment by Duval and Tweedie trim-and-fill analysis). Criteria for upgrades included a significant dose-response gradient.
[b] For the interpretation of the magnitude, we used the MIDs (see above) to assess the importance of magnitude of our pooled estimates using the effect size categories according to new GRADE guidance. We then used the MIDs to assess the importance of the magnitude of our point estimates using the effect size categories according to GRADE guidance (3-5) as follows: large effect (≥5x MID); moderate effect (≥2x MID); small important effect (≥1x MID); and trivial/unimportant effect (< 1 MID).
*To convert SMD to %liver fat, multiply the SMD by the baseline pooled standard deviation, 0.71%.
[1] Although the effect seen in substitution trials for total fructose-containing sugars was mainly driven from SSBs, which contributed 52.2% of the weight in the overall analysis, we did not double downgrade for very serious indirectness since there was no evidence for interaction by food source (p=0.665) and the removal of SSBs did not lead to a change in the overall estimate of effect (SMD= 0.63 [95% CI, -0.11 to 1.38], p_{SMD}=0.10).
[2] Downgrade for serious imprecision as the 95% confidence interval (-0.07 to 0.79) overlaps the MID of clinically important harm for IHCL (0.26).
[3] Although a significant publication bias was detected at p=0.001 and p=0.001 in Begg's and Egger's tests, respectfully, we did not downgrade for publication bias as the imputation of 6 trials from trim-and-fill analyses did not change the significance on the overall effect of IHCL (SMD= -0.00 [95% CI, -0.49 to 0.48]).
[4] Double downgrade for very serious indirectness as the only food source available for analyses was SSBs, thus limiting the ability to assess differences in food sources.
[5] Although a significant publication bias was detected at p<0.001 and p=0.021 in Begg's and Egger's tests, respectfully, we did not downgrade for publication bias as the imputation of 4 trials from trim-and-fill analyses did not change the significance on the overall effect of IHCL (SMD= 1.49 [95% CI, 0.90 to 2.09]).
[6] Double downgrade for very serious indirectness as the only food source available for analyses was SSBs, thus limiting the ability to assess differences in food sources.
[7] Downgrade for serious imprecision as the 95% confidence interval (-1.60 to 0.56) overlaps the MID of clinically important benefit and harm for IHCL (0.26).
[8] No downgrade for publication bias, as publication bias could not be assessed due to lack of power for assessing funnel plot asymmetry and small study effects (<10 trial comparisons included in the meta-analysis).
[9] No dose-response assessment was made as <6 trials were available for analyses.
[10] Although there was substantial heterogeneity in the analysis, we did not downgrade for serious inconsistency, since it was explained when studies by Lehtonen et al. 2010, Purkins et al. 2004, and Schwimmer et al. 2019 were individually removed as part of *a priori* sensitivity analyses (Original: I^2=53%, P_Q=0.001; after the removal of Lehtonen et al. 2010: I^2=45%, P_Q=0.007; after the removal of Purkins et al. 2004: I^2=37%, P_Q=0.028; and after removal of after the removal of Schwimmer et al. 2019: I^2=41%, P_Q=0.016).
[11] Although a significant publication bias was detected at p=0.066 and p=0.008 in Begg's and Egger's test, respectfully, we did not downgrade for publication bias as the imputation of 3 trials from trim-and-fill analyses did not change the significance on the overall effect of ALT (MD= -0.71U/L [95% CI, -2.31 to 0.89]).
[12] Double downgrade for very serious indirectness. Although there was no evidence for interaction by food source (p=0.159), there was one food source (SSBs) which contributed the majority (42.2%) of the weight in the overall analysis, thus limiting the ability to assess differences in food sources.
[13] Although a significant dose response was detected ($coef_{linear}$=0.153 [95% CI, 0.035 to 0.271], p_{linear}=0.011), we did not upgrade for dose response as we assessed that there was an influence by food source.

[14] Double downgrade for very serious indirectness as there was a significant interaction by food source (p=0.061) indicating that there is biological plausibility of differences in behaviour of foods due to the food matrices and since SSBs and mixed sources were the only food sources available for analyses, thus limiting the ability to assess differences in food sources.
[15] Downgrade for serious imprecision as the 95% confidence interval (-15.91 to 6.19) overlaps the MIDs of clinically important harm, benefit, and the no effect line for ALT (2.85U/L).
[16] Downgrade for serious risk of bias as we detected "High" risk of bias ratings for all trials under the domains of sequence generation and allocation concealment, due to the fact that they were not randomized.
[17] Double downgrade for very serious indirectness as SSBs was the only one food source available for analyses, thus limiting the ability to assess differences in food sources and only two trial comparisons were available and were conducted in Finland with healthy young adult participants, which leads to poor applicability of results to the general population.
[18] Downgrade for serious imprecision as the 95% confidence interval (-0.87 to 2.92) overlaps the MIDs of clinically important harm and the no effect line for ALT (2.85U/L).
[19] Although a significant publication bias was detected at p=0.027 and p=0.019 in Begg's and Egger's tests, respectfully, we did not downgrade for publication bias as the imputation of 1 trial from trim-and-fill analyses did not change the significance on the overall effect of AST (MD= 0.22U/L [95% CI, -1.72 to 1.29], p_{MD}=0.778).
[20] Double downgrade for very serious indirectness as there was a significant interaction by food source (p=0.007) indicating that there is biological plausibility of differences in behaviour of foods due to the food matrices and since SSBs and mixed sources were the only food sources available for analyses, thus limiting the ability to assess differences in food sources.
[21] Double downgrade for very serious indirectness as only one food source was available for analyses, thus limiting the ability to assess differences in food sources. Further, only two trial comparisons were available and were conducted in Switzerland, with overweight or obese adult participants with a small sample size (n=27), which leads to poor applicability of results to the general population.
[22] Downgrade for serious imprecision as the 95% confidence interval (-8.60 to -1.76) overlaps the MIDs of clinically important harm, benefit, and the no effect line for ALT (2.85U/L).
[22] Downgrade for serious ROB since the overall pooled estimate was driven by high ROB trials. All trials under the domains of sequence generation and allocation concealment were rated as high risk of bias since they were not randomized.
[24] Double downgrade for very serious indirectness as SSBs was the only one food source available for analyses, thus limiting the ability to assess differences in food sources. Further, only two trial comparisons were available and were conducted in Finland with healthy young adult participants, which leads to poor applicability of results to the general population.
ALT=alanine aminotransferase; AST=aspartate aminotransferase; CI=confidence interval; DR=dose response; IHCL=intrahepatocellular lipid; MD=mean difference; MID=minimally important difference; SSB=sugar-sweetened beverage.

Supplementary Table S8: GRADE certainty of evidence assessment for the effect of fructose-containing sugars and NAFLD outcomes by important food source of fructose-containing sugars

Outcome and trial (N)	Design	GRADE assessment: Downgrades: Risk of bias	Inconsistency	Indirectness	Imprecision	Publication bias	Upgrades: Dose response		Effect (MD or SMD [95%CI], P_{MD})	Certainty of Evidence[a]	Interpretation of magnitude of effect[b]
**IHCL in addition trials (SMD)***											
SSB (13)	Randomized and non-randomized trials	Not serious	Not serious	Not serious	Not serious	None[1]	None	↑	SMD 1.72 [1.08 to 2.36], p<0.001	⊕⊕⊕⊕ High	Large
**IHCL in subtraction trials (SMD)***											
SSB (2)	Randomized trials	Not serious	Not serious	Serious[2]	Serious[3]	None[4]	N/A[5]	↔	SMD -0.52 [-1.60 to 0.56], p=0.345	⊕⊕◯◯ Low	No effect
**ALT in addition trials (U/L)**											
SSB (12)	Randomized and non-randomized trials	Not serious	Not serious	Not serious	Serious[6]	None[4]	None	↔	MD 3.09U/L [0.49 to 5.68], p=0.020	⊕⊕⊕◯ Moderate	Small important
100% Fruit juice (7)	Randomized trials	Not serious	Not serious	Serious[7]	Not serious	None[4]	None	↔	MD -0.80U/L [-2.43 to 0.84], p=0.340	⊕⊕⊕◯ Moderate	No effect
Fruit (4)	Randomized trials	Not serious	Not serious	Serious[8]	Serious[9]	None[4]	N/A[5]	↔	MD 0.44U/L [-2.22 to 3.10], p=0.746	⊕⊕◯◯ Low	No effect
Dried fruit (2)	Randomized trials	Not serious	Not serious	Serious[10]	Serious[11]	None[4]	N/A[5]	↔	MD -2.58U/L [-17.46 to 12.31], p=0.735	⊕⊕◯◯ Low	No effect
Sweets and desserts (1)	Randomized trials	Not serious	Not serious[12]	Serious[13]	Serious[14]	None[4]	N/A[5]	↔	MD -2.15U/L [-20.93 to 16.63], p=0.822	⊕⊕◯◯ Low	No effect
Honey (5)	Randomized trials	Not serious	Not serious	Serious[15]	Not serious	None[4]	N/A[5]	↔	MD -0.87U/L [-5.33 to 3.60], p=0.703	⊕⊕⊕◯ Moderate	No effect
**ALT subtraction trials (U/L)**											
SSB (2)	Randomized trials	Not serious	Not serious	Not serious	Serious[16]	None[4]	N/A[5]	↔	MD 1.33U/L [-7.55 to 10.22], p=0.769	⊕⊕⊕◯ Moderate	No effect
Mixed sources (2)	Non-randomized trials	Serious[17]	Not serious	Serious[18]	Serious[19]	None[4]	N/A[5]	↔	MD -15.68U/L [-32.90 to 1.54], p=0.074	⊕◯◯◯ Very low	No effect
**ALT in *ad libitum* trials (U/L)**											

Mixed sources (2)	Non-randomized trials	Serious[20]	Not serious	Serious[21]	Serious[22]	None[4]	N/A[5]	↔	MD 1.02U/L [-0.87 to 2.92], p=0.290	⊕○○○ Very low	No effect
**AST in addition trials (U/L)**											
SSB (9)	Randomized and non-randomized trials	Not serious	Not serious	Not serious	Not serious	None[4]	None	↔	MD 0.29U/L [-1.07 to 1.64], p=0.677	⊕⊕⊕⊕ High	No effect
100% Fruit juice (3)	Randomized trials	Not serious	Not serious	Not serious	Serious[23]	None[4]	N/A[5]	↔	MD 0.02U/L [-2.66 to 2.71], p=0.986	⊕⊕⊕○ Moderate	No effect
Fruit (2)	Randomized trials	Not serious	Not serious	Not serious	Serious[24]	None[4]	N/A[5]	↔	MD -1.60U/L [-4.92 to 1.72], p=0.346	⊕⊕⊕○ Moderate	No effect
Dried fruit (2)	Randomized trials	Not serious	Not serious	Not serious	Serious[25]	None[4]	N/A[5]	↔	MD -5.24U/L [-14.40 to 3.93], p=0.263	⊕⊕⊕○ Moderate	No effect
Sweets and desserts (1)	Randomized trials	Not serious	Not serious[26]	Serious[27]	Serious[28]	None[4]	N/A[5]	↔	MD -6.08U/L [-14.50 to 2.34], p=0.157	⊕⊕○○ Low	No effect
Honey (4)	Randomized trials	Not serious	Not serious	Not serious	Serious[29]	None[4]	N/A[5]	↔	MD 1.05U/L [-4.04 to 6.15], p=0.685	⊕⊕⊕○ Moderate	No effect
**AST in subtraction trials (U/L)**											
SSB (2)	Randomized trials	Not serious	Not serious	Serious[30]	Serious[31]	None[4]	N/A[5]	↔	MD -1.33U/L [-7.61 to 4.96], p=0.679	⊕⊕○○ Low	No effect
Mixed sources (2)	Non-randomized trials	Serious[32]	Not serious	Serious[33]	Not serious	None[4]	N/A[5]	↓	MD -7.33U/L [-12.78 to -1.87], p=0.009	⊕⊕○○ Low	Moderate
**AST in *ad libitum* trials (U/L)**											
Mixed sources (2)	Non-randomized trials	Serious[34]	Not serious	Serious[35]	Not serious	None[4]	N/A[5]	↔	MD -0.45U/L [-1.11 to 0.21], p=0.183	⊕⊕○○ Low	No effect

[a] Since all included trials were randomized or non-randomized controlled trials, the certainty of the evidence was graded as high for all outcomes by default and then downgraded or upgraded based on pre-specified criteria. Criteria for downgrades included risk of bias (downgraded if the majority of trials were considered to be at high risk of bias); inconsistency (downgraded if there was substantial unexplained heterogeneity [$I^2 \geq 50\%$, $p < 0.10$]; indirectness (downgraded if there were factors absent or present relating to the participants, interventions, or outcomes that limited the generalizability of the results); imprecision (downgraded if the 95% confidence interval crossed the minimally important difference [MID] for harm or benefit set at 0.26 for IHCL, 2.85U/L for ALT(2), and 2.55U/L for AST(2)); and publication bias (downgraded if there is evidence of publication bias based on funnel plot asymmetry and/or significant Egger's or Begg's tests (P<0.10) with confirmation by adjustment by Duval and Tweedie trim-and-fill analysis). Criteria for upgrades included a significant dose-response gradient.
[b] For the interpretation of the magnitude, we used the MIDs (see above) to assess the importance of magnitude of our pooled estimates using the effect size categories according to new GRADE guidance. We then used the MIDs to assess the importance of the magnitude of our point estimates using the effect size categories according to GRADE guidance (3-5) as follows: large effect (≥5x MID); moderate effect (≥2x MID); small important effect (≥1x MID); and trivial/unimportant effect (< 1 MID).
*To convert SMD to %liver fat, multiply the SMD by the baseline pooled standard deviation, 0.71%.

[1] Although a significant publication bias was detected at p<0.001 and p=0.021 in Begg's and Egger's tests, respectfully, we did not downgrade for publication bias as the imputation of 4 trials from trim-and-fill analyses did not change the significance on the overall effect of IHCL (SMD=1.49 [0.90 to 2.09]).
[2] Downgrade for serious indirectness as only two trial comparisons were available and were conducted in Switzerland, with overweight or obese adult participants with a small sample size (n=27), which leads to poor applicability of results to the general population.
[3] Downgrade for serious imprecision as the 95% confidence interval (-1.60 to 0.56) overlaps the MIDs of clinically important harm, benefit, and the no effect line for IHCL (0.26).
[4] No downgrade for publication bias, as publication bias could not be assessed due to lack of power for assessing funnel plot asymmetry and small study effects (<10 trial comparisons included in the meta-analysis).
[5] No dose-response assessment was made as <6 trials were available for analyses.
[6] Downgrade for serious imprecision as the 95% confidence interval (2.63 to 8.00) overlaps the MIDs of clinically important harm for ALT (2.85U/L).
[7] Downgrade for serious indirectness as all trial comparisons included healthy mixed weight adult participants which leads to poor applicability of the results to the general population.
[8] Downgrade for serious indirectness as three trial comparisons had a small sample size and included participants who were healthy (n=23) or had diabetes (n=22) which leads to poor applicability of the results to the general population.
[9] Downgrade for serious imprecision as the 95% confidence interval (-2.43 to 3.40) overlaps the MID of clinically important harm and the no effect line for ALT (2.85U/L).
[10] Downgrade for serious indirectness as trial comparisons had a small sample size and included adult participants who were healthy (n=70) or had diabetes (n=72) which leads to poor applicability of the results to the general population.
[11] Downgrade for serious imprecision as the 95% confidence interval (-7.06 to 3.35) overlaps the MIDs of clinically important harm and the no effect line for ALT (2.85U/L).
[12] We did not downgrade for serious inconsistency as ≤2 trials were included in the meta-analysis and we were thus unable to test for asymmetry.
[13] Downgrade for serious indirectness as only one trial comparison was included and had a small sample size (n=42) and included participants with NAFLD which leads to poor applicability of the results to the general population.
[14] Downgrade for serious imprecision as the 95% confidence interval (-20.93 to 16.63) overlaps the MIDs of clinically important harm and the no effect line for ALT (2.85U/L).
[15] Downgrade for serious indirectness as only five trial comparisons were included and included older adult participants with HIV (n=164), breast cancer (n=72), or diabetes (n=48), which leads to poor applicability of the results to the general population.
[16] Downgrade for serious imprecision as the 95% confidence interval (-7.55 to 10.22) overlaps the MIDs of clinically important harm, benefit, and the no effect line for ALT (2.85U/L).
[17] Downgrade for serious risk of bias as we detected "High" risk of bias ratings for all trials under the domains of sequence generation, allocation concealment, and other risk of bias, since they were not randomized and the crossover trials did not include a washout period.
[18] Downgrade for serious indirectness as trial comparisons had a small sample size and included adult participants who were mixed weight (n=11) or obese (n=11) which leads to poor applicability of the results to the general population.
[19] Downgrade for serious imprecision as the 95% confidence interval -32.90 to 1.54) overlaps the MIDs of clinically important benefit and the no effect line for ALT (2.85U/L).
[20] Downgrade for serious risk of bias as we detected "High" risk of bias ratings for all trials under the domains of sequence generation and allocation concealment, due to the fact that they were not randomized.
[21] Downgrade for serious indirectness as only two trial comparisons were available and were conducted in Finland with healthy young adult participants (n=100), which leads to poor applicability of results to the general population.

[22] Downgrade for serious imprecision as the 95% confidence interval (-0.87 to 2.92) overlaps the MIDs of clinically important harm and the no effect line for ALT (2.85U/L).
[23] Downgrade for serious imprecision as the 95% confidence interval (-2.66 to 2.71) overlaps the MIDs of clinically important harm, benefit, and the no effect line for AST (2.55U/L).
[24] Downgrade for serious imprecision as the 95% confidence interval (-4.92 to 1.72) overlaps the MIDs of clinically important harm, benefit, and the no effect line for AST (2.55U/L).
[25] Downgrade for serious imprecision as the 95% confidence interval (-14.40 to 3.93) overlaps the MIDs of clinically important harm, benefit, and the no effect line for AST (2.55U/L).
[26] We did not downgrade for serious inconsistency as ≤2 trials were included in the meta-analysis and we were thus unable to test for asymmetry.
[27] Downgrade for serious indirectness as only one trial comparison was available and was conducted in Sweden, which included a small sample size (N=42) of adult participants with NAFLD, which leads to poor applicability of results to the general population.
[28] Downgrade for serious imprecision as the 95% confidence interval (-14.50 to 2.34) overlaps the MIDs of clinically important harm, benefit, and the no effect line for AST (2.55U/L).
[29] Downgrade for serious imprecision as the 95% confidence interval (-4.04 to 6.15]) overlaps the MIDs of clinically important harm, benefit, and the no effect line for AST (2.55U/L).
[30] Downgrade for serious indirectness as only two trial comparisons were available and were conducted in Switzerland, with overweight or obese adult participants with a small sample size (n=27), which leads to poor applicability of results to the general population.
[31] Downgrade for serious imprecision as the 95% confidence interval (-7.61 to 4.96) overlaps the MIDs of clinically important harm and the no effect line for AST (2.55U/L).
[32] Downgrade for serious risk of bias as we detected "High" risk of bias ratings for all trials under the domains of sequence generation, allocation concealment, and other risk of bias, since they were not randomized and the crossover trials did not include a washout period.
[33] Downgrade for serious indirectness as trial comparisons had a small sample size and included adult participants who were mixed weight (n=11) or obese (n=11) which leads to poor applicability of the results to the general population.
[34] Downgrade for serious risk of bias as we detected "High" risk of bias ratings for all trials under the domains of sequence generation and allocation concealment, due to the fact that they were not randomized.
[35] Downgrade for serious indirectness as only two trial comparisons were available and were conducted in Finland with healthy young adult participants (n=100), which leads to poor applicability of results to the general population.
ALT=alanine aminotransferase; AST=aspartate aminotransferase; CI=confidence interval; DR=dose response; IHCL=intrahepatocellular lipid; MD=mean difference; MID=minimally important difference; NAFLD=non-alcoholic fatty liver disease; SSB=sugar-sweetened beverage.

Supplementary Table S9: Potential mechanisms to explain the effect of food sources of fructose-containing sugars and non-alcoholic fatty liver disease

Potential mechanism	Description	References
SSBs consumed as excess calories stimulate disruptions in hepatic fatty acid metabolism	An increase in NAFLD measurements as a result of the consumption of fructose-containing sugars coming from SSBs could be explained when fructose is consumed as excess energy, which can stimulate liver fat accumulation by influencing hepatic de novo lipogenesis and inhibiting beta-oxidation.[1] Additionally, metabolizing high amounts of fructose depletes ATP levels, resulting in an accumulation of uric acid, which can disrupt fatty acid metabolism and contribute to NAFLD progression.[1]	1. Jensen T, Abdelmalek MF, Sullivan S, et al. Fructose and sugar: A major mediator of non-alcoholic fatty liver disease. J Hepatol 2018; 68(5): 1063-75.
The different food matrices/forms of food sources of fructose-containing sugars and NAFLD outcomes	The various effects of different food sources of fructose-containing sugars on NAFLD markers could be explained by its food matrix/form. SSBs contain few nutrients and are the leading contributors to the consumption of added sugars in the diet.[2] Furthermore, SSBs may not induce compensatory eating behaviours to reduce energy intake in subsequent meals.[3] Thus, the consumption of SSBs may result in excessive energy intake and contribute to NAFLD development.[4] By contrast, other food sources such as fruits are dense in nutrients which may offset the effect of fructose-containing sugars.[4] Nutrients in fruit including fibre has been shown to aid in maintaining blood glucose, insulin, and free fatty acid levels in NAFLD patients, while phytochemicals and antioxidants may be protective of hepatic steatosis.[1,5] One randomized controlled trial conducted in Thailand[6] of 10 female participants with type 1 diabetes followed for four weeks has shown that a low glycemic index (GI) diet reduced levels of ALT compared to a high GI diet, and another quasi-randomized controlled trial in Israel[7] of 128 middle-aged adults with obesity and diabetes followed for 12 months showed that a low glycemic load (GL) Mediterranean diet had ~28% lower levels of ALT compared to the standard of care American Diabetes Association (ADA) diet (14.4 ±1.7 U/L in the low GL diet and 19.8 ±1.4 in the ADA diet, $p<0.001$). The harmful effect found in mixed sources for AST may be explained by SSBs being included in the dietary intervention, which would thus contribute to increasing AST levels.	2. Ludwig DS, Peterson KE, Gortmaker SL. Relation between consumption of sugar-sweetened drinks and childhood obesity: a prospective, observational analysis. Lancet (London, England) 2001; 357(9255): 505-8.<br>3. Choo VL, Viguiliouk E, Blanco Mejia S, et al. Food sources of fructose-containing sugars and glycaemic control: systematic review and meta-analysis of controlled intervention studies. BMJ 2018; 363: k4644.<br>4. Mirmiran P, Amirhamidi Z, Ejtahed HS, Bahadoran Z, Azizi F. Relationship between Diet and Non-alcoholic Fatty Liver Disease: A Review Article. Iran J Public Health 2017; 46(8): 1007-17<br>5. George ES, Forsyth A, Itsiopoulos C, et al. Practical Dietary Recommendations for the Prevention and Management of Nonalcoholic Fatty Liver Disease in Adults. Adv Nutr (Bethesda) 2018; 9(1): 30-40.<br>6. Komindr S, Ingsriswang S, Lerdvuthisopon N, Boontawee A. Effect of long-term intake of Asian food with different glycemic indices on diabetic control and protein conservation in type 2 diabetic patients. Journal of the Medical Association of Thailand = Chotmaihet thangphaet 2001; 84: 85-97.<br>7. Fraser A, Abel R, Lawlor DA, Fraser D, Elhayany A. A modified Mediterranean diet is associated with the greatest reduction in alanine aminotransferase levels in obese type 2 diabetes patients: results of a quasi-randomised controlled trial. Diabetologia 2008; 51(9): 1616-22.
Subtraction trials resulted in no effect on NAFLD outcomes due to energy compensation	Although a beneficial effect on AST was seen from the subtraction trials in which energy from fructose-containing sugars was lowered relative to a control diet, no benefit was seen for IHCL and ALT. This was unexpected as a previous study where the restriction of high fructose-containing foods and subsequent decrease in caloric intake over six weeks led to a decrease in hepatic steatosis in children.[8] However, it is worth noting that all subtraction trials in our analyses were conducted with adults, and thus may not directly be comparable to previous findings in children. Our results might also be explained by an elicited energy compensation such that the sugars are replaced by other macronutrients.[3] It is also possible that energy expenditure and metabolism are altered to match the decreased levels of energy, thereby maintaining weight and NAFLD status.[3] No effect was also observed for ALT and AST from ad libitum trials, where mixed sources of fructose-containing sugars were freely exchanged in the diet. In the two *ad libitum* trials[9] included in our meta analyses, various food sources of fructose-containing sugars were provided, including	8. Ibarra-Reynoso LDR, López-Lemus HL, Garay-Sevilla ME, Malacara JM. Effect of Restriction of Foods with High Fructose Corn Syrup Content on Metabolic Indices and Fatty Liver in Obese Children. Obesity facts 2017; 10(4): 332-40.<br>9. Mäkinen KK, Scheinin A. Turku sugar studies XIII. Effect of the diet on certain clinico-chemical values of serum. Acta Odontol Scand 1976; 34(6): 371-80.

	pastries, condiments, and fruit and vegetable products, which may have opposing effects on ALT and AST measures.	

ALT=alanine aminotransferase; AST=aspartate aminotransferase; ATP=adenosine triphosphate; IHCL=intrahepatocellular lipid; NAFLD=non-alcoholic fatty liver disease; SSB=sugar-sweetened beverage.

Supplementary Figures

Supplementary Figure S1: Risk of bias proportion graph for the effect of important food sources of fructose-containing sugars and IHCL (SMD) in substitution trials

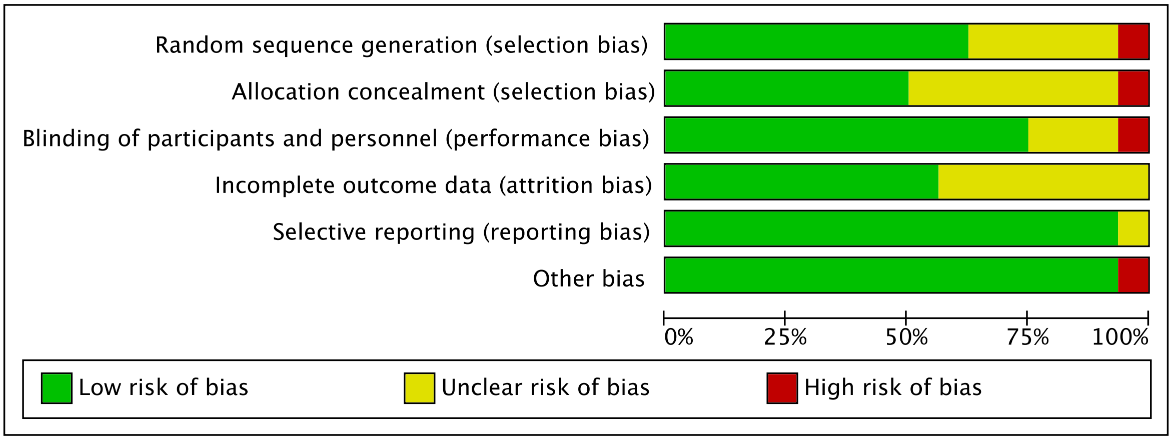

Colored bars represent the proportion of trials assessed as low (green), unclear (yellow) or high (red) risk of bias for the six domains of bias above according to criteria set by the Cochrane Risk of Bias tool in the 14 included controlled trial comparisons.
High other risk of bias (carry-over effect) was given to crossover trials which had no washout between interventions. Trials which did not have this characteristic were rated as Low.
IHCL=intrahepatocellular lipid.

Supplementary Figure S2: Risk of bias proportion graph for the effect of important food sources of fructose-containing sugars and IHCL (SMD) in addition trials

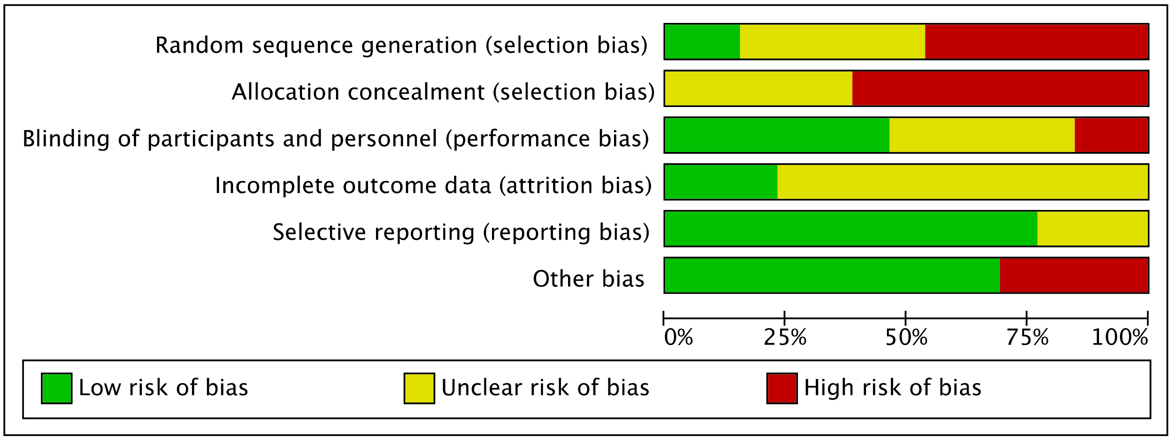

Colored bars represent the proportion of trials assessed as low (green), unclear (yellow) or high (red) risk of bias for the six domains of bias above according to criteria set by the Cochrane Risk of Bias tool in the 11 included controlled trial comparisons.
High other risk of bias (carry-over effect) was given to crossover trials which had no washout between interventions. Trials which did not have this characteristic were rated as Low.
IHCL=intrahepatocellular lipid.

Supplementary Figure S3: Risk of bias proportion graph for the effect of important food sources of fructose-containing sugars and IHCL (SMD) in subtraction trials

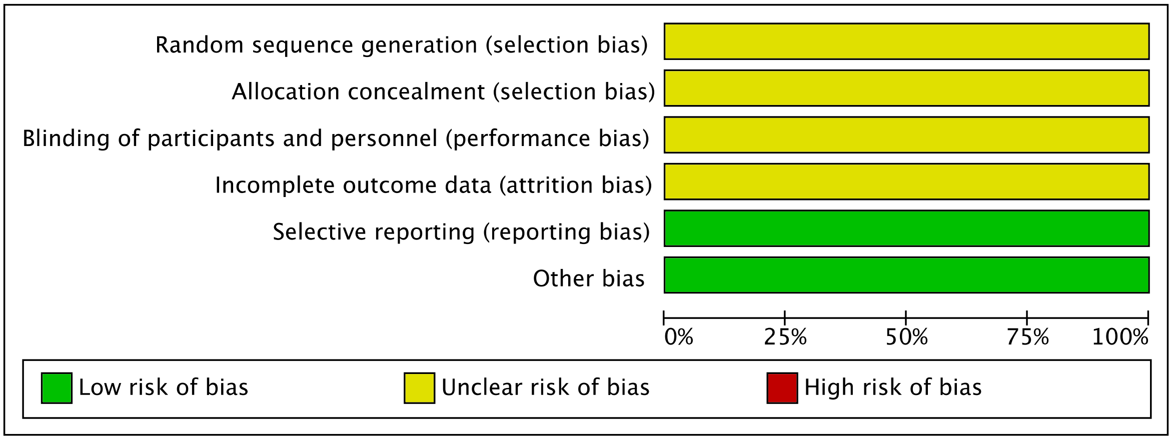

Colored bars represent the proportion of trials assessed as low (green), unclear (yellow) or high (red) risk of bias for the six domains of bias above according to criteria set by the Cochrane Risk of Bias tool in the 2 included controlled trial comparisons, both from the same study.
High other risk of bias (carry-over effect) was given to crossover trials which had no washout between interventions. Trials which did not have this characteristic were rated as Low.
IHCL=intrahepatocellular lipid.

Supplementary Figure S4: Risk of bias proportion graph for the effect of important food sources of fructose-containing sugars and ALT (U/L) in substitution trials

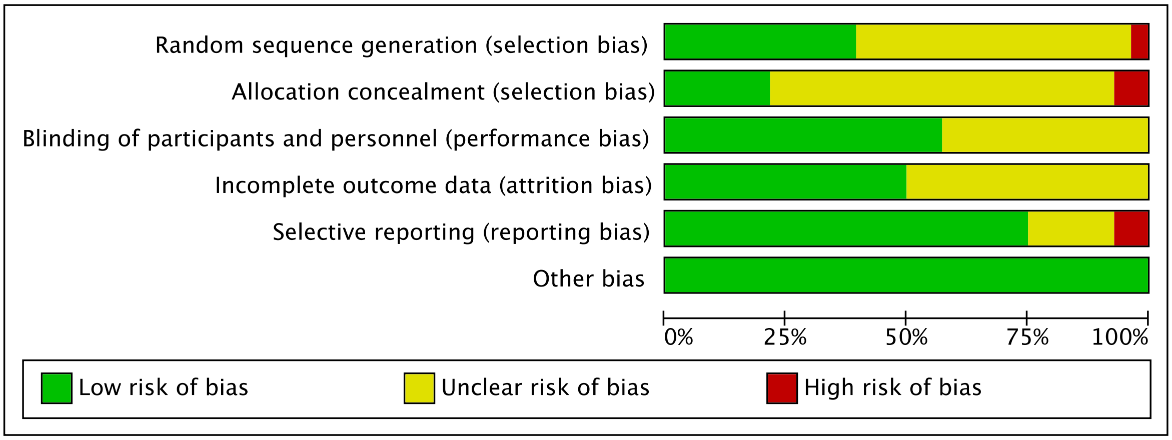

Colored bars represent the proportion of trials assessed as low (green), unclear (yellow) or high (red) risk of bias for the six domains of bias above according to criteria set by the Cochrane Risk of Bias tool in the 25 included controlled trial comparisons.
High other risk of bias (carry-over effect) was given to crossover trials which had no washout between interventions. Trials which did not have this characteristic were rated as Low.
ALT=alanine aminotransferase.

Supplementary Figure S5: Risk of bias proportion graph for the effect of important food sources of fructose-containing sugars and ALT (U/L) in addition trials

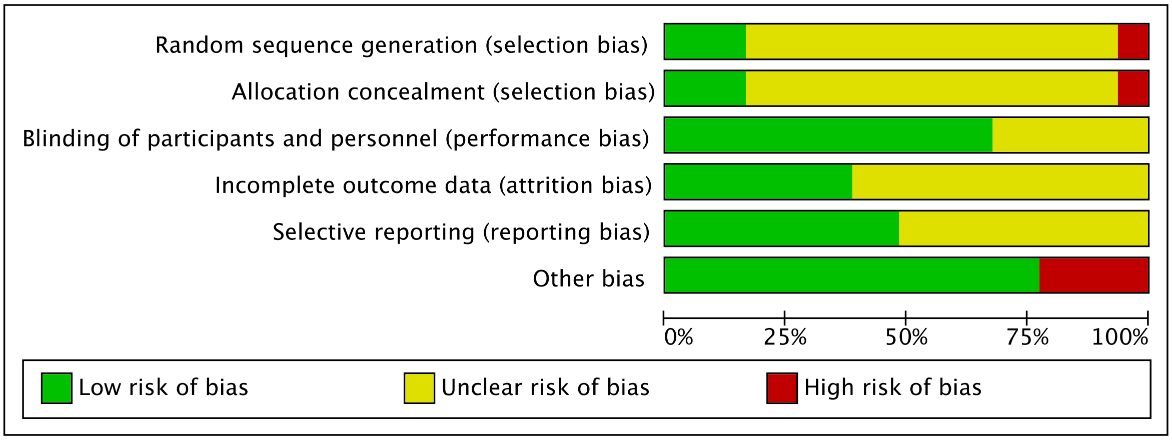

Colored bars represent the proportion of trials assessed as low (green), unclear (yellow) or high (red) risk of bias for the six domains of bias above according to criteria set by the Cochrane Risk of Bias tool in the 23 included controlled trial comparisons.
High other risk of bias (carry-over effect) was given to crossover trials which had no washout between interventions. Trials which did not have this characteristic were rated as Low.
ALT=alanine aminotransferase.

Supplementary Figure S6: Risk of bias proportion graph for the effect of important food sources of fructose-containing sugars and ALT (U/L) in subtraction trials

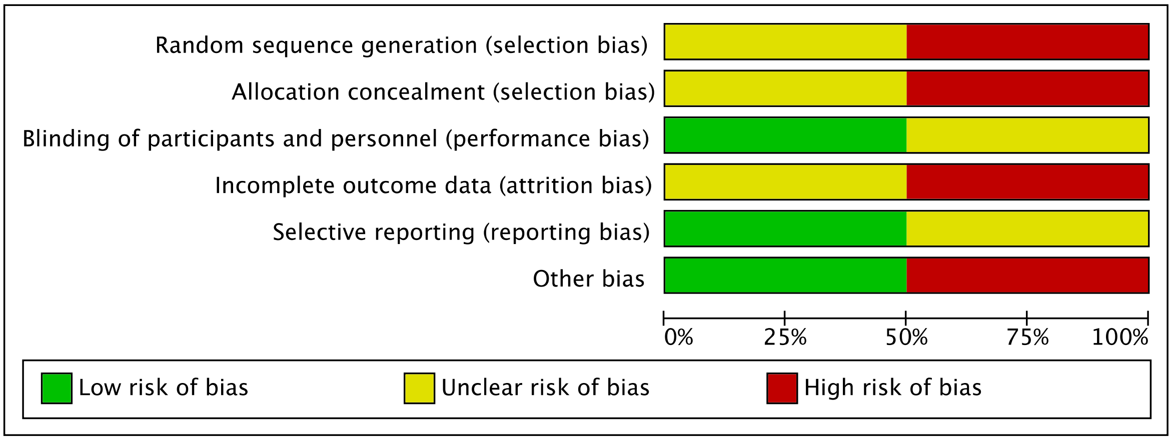

Colored bars represent the proportion of trials assessed as low (green), unclear (yellow) or high (red) risk of bias for the six domains of bias above according to criteria set by the Cochrane Risk of Bias tool in the 2 included controlled trial comparisons, both from the same study.
High other risk of bias (carry-over effect) was given to crossover trials which had no washout between interventions. Trials which did not have this characteristic were rated as Low.
ALT=alanine aminotransferase.

Supplementary Figure S7: Risk of bias proportion graph for the effect of important food sources of fructose-containing sugars and ALT (U/L) in *ad libitum* trials

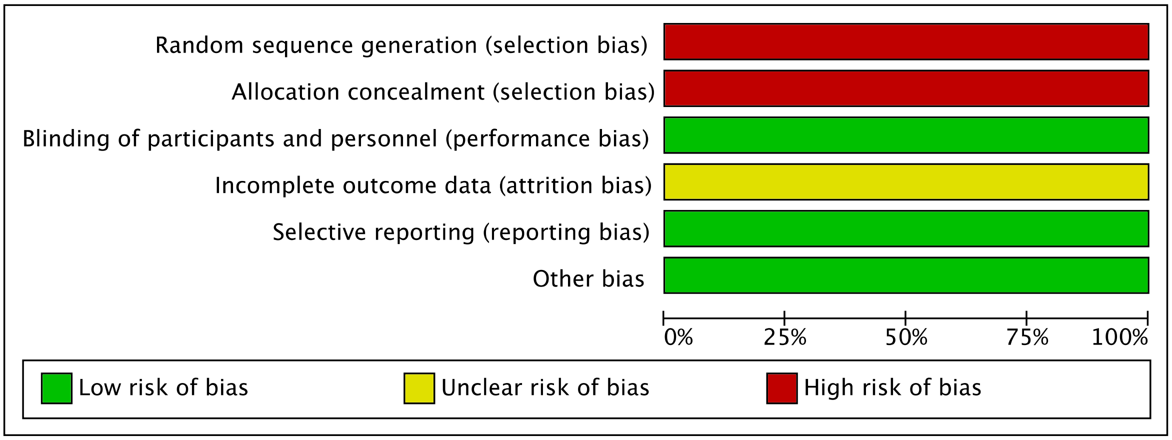

Colored bars represent the proportion of trials assessed as low (green), unclear (yellow) or high (red) risk of bias for the six domains of bias above according to criteria set by the Cochrane Risk of Bias tool in the 2 included controlled trial comparisons, both from the same study.
High other risk of bias (carry-over effect) was given to crossover trials which had no washout between interventions. Trials which did not have this characteristic were rated as Low.
ALT=alanine aminotransferase.

Supplementary Figure S8: Risk of bias proportion graph for the effect of important food sources of fructose-containing sugars and AST (U/L) in substitution trials

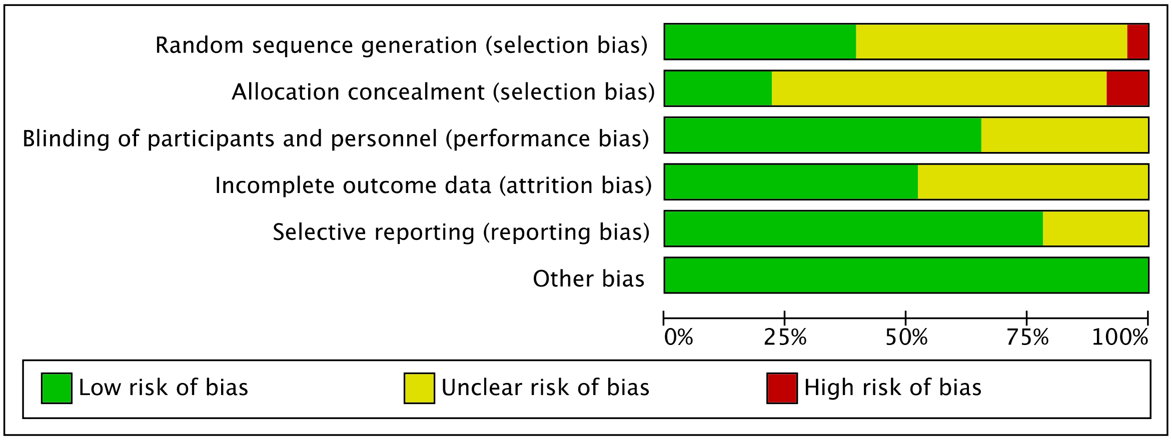

Colored bars represent the proportion of trials assessed as low (green), unclear (yellow) or high (red) risk of bias for the six domains of bias above according to criteria set by the Cochrane Risk of Bias tool in the 21 included controlled trial comparisons.
High other risk of bias (carry-over effect) was given to crossover trials which had no washout between interventions. Trials which did not have this characteristic were rated as Low.
AST=aspartate aminotransferase.

Supplementary Figure S9: Risk of bias proportion graph for the effect of important food sources of fructose-containing sugars and AST (U/L) in addition trials

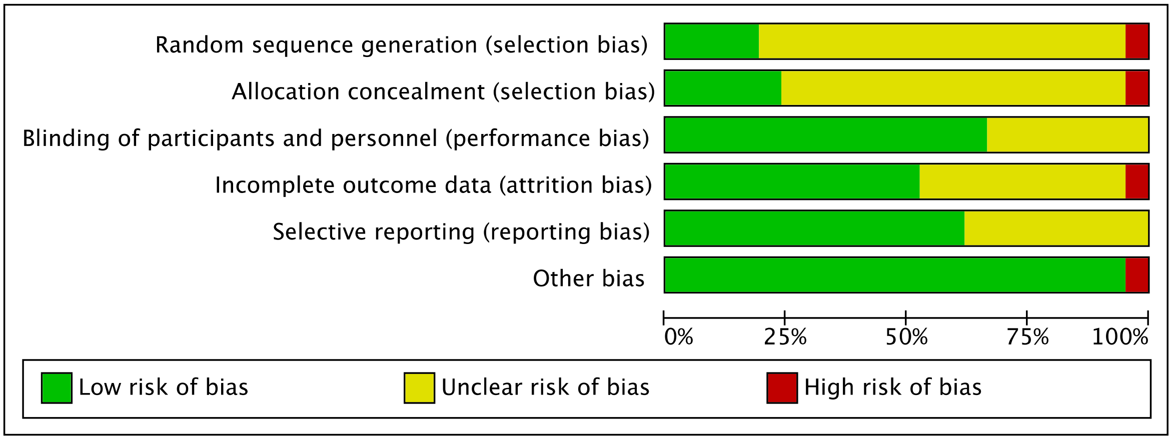

Colored bars represent the proportion of trials assessed as low (green), unclear (yellow) or high (red) risk of bias for the six domains of bias above according to criteria set by the Cochrane Risk of Bias tool in the 13 included controlled trial comparisons.
High other risk of bias (carry-over effect) was given to crossover trials which had no washout between interventions. Trials which did not have this characteristic were rated as Low.
AST=aspartate aminotransferase.

Supplementary Figure S10: Risk of bias proportion graph for the effect of important food sources of fructose-containing sugars and AST (U/L) in subtraction trials

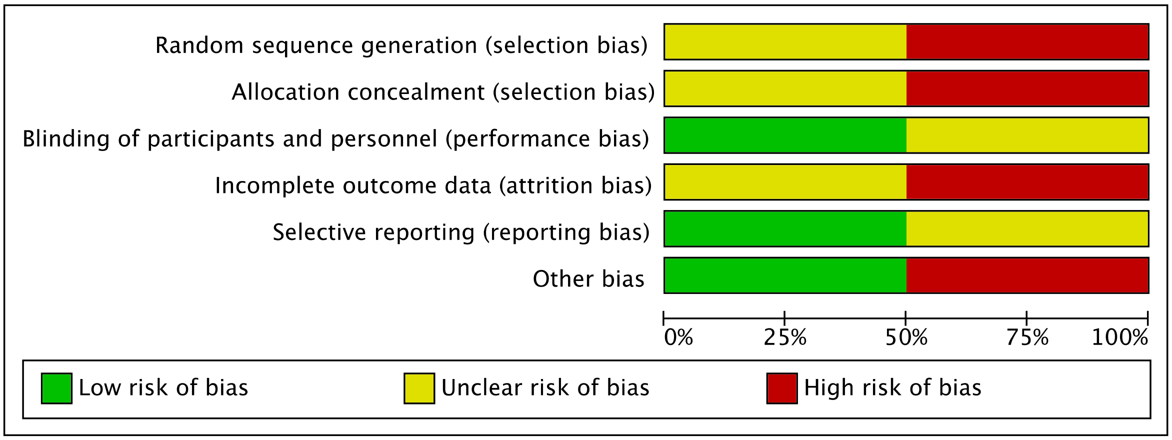

Colored bars represent the proportion of trials assessed as low (green), unclear (yellow) or high (red) risk of bias for the six domains of bias above according to criteria set by the Cochrane Risk of Bias tool in the 13 included controlled trial comparisons.
High other risk of bias (carry-over effect) was given to crossover trials which had no washout between interventions. Trials which did not have this characteristic were rated as Low.
AST=aspartate aminotransferase.

Supplementary Figure S11: Risk of bias proportion graph for the effect of important food sources of fructose-containing sugars and AST (U/L) in *ad libitum* trials

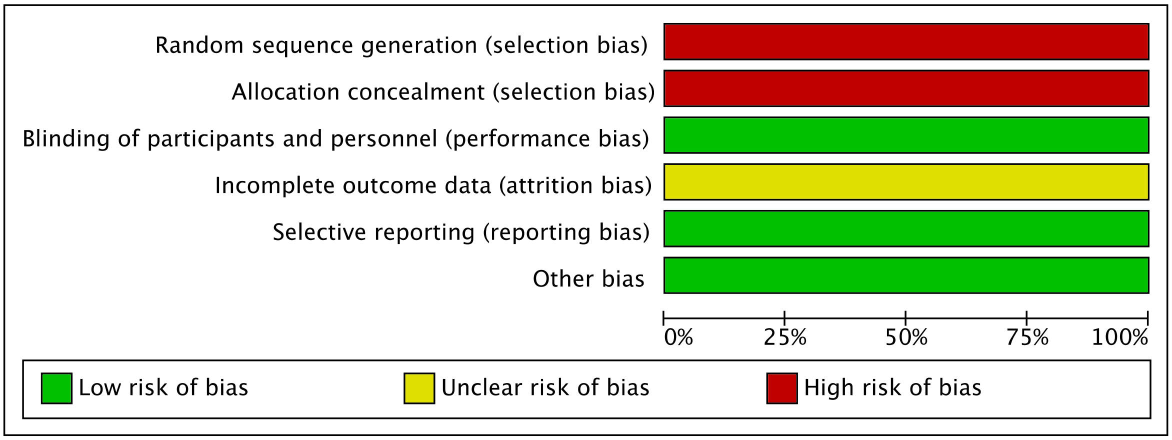

Colored bars represent the proportion of trials assessed as low (green), unclear (yellow) or high (red) risk of bias for the six domains of bias above according to criteria set by the Cochrane Risk of Bias tool in the 13 included controlled trial comparisons.

High other risk of bias (carry-over effect) was given to crossover trials which had no washout between interventions. Trials which did not have this characteristic were rated as Low.

AST=aspartate aminotransferase.

Supplementary Figure S12: Forest plot of controlled trials of the effect of important food sources of fructose-containing sugars and IHCL (SMD) in substitution trials

IHCL (SMD)

Substitution trials

Trial or subgroup	N Control	N Intervention	Mean difference with 95% CI	Weight (%)	A	B	C	D	E	F
**01. SSB**										
Jin et al. 2014	12	9	-0.27 [-1.68 to 1.14]	8.41	U	U	L	L	L	L
Johnston et al. 2013 (T1)	17	15	0.32 [-1.10 to 1.74]	8.31	L	L	L	L	L	L
Johnston et al. 2013 (T2)	17	15	-0.32 [-1.74 to 1.10]	8.31	L	L	L	L	L	L
Maersk et al. 2012 (M)	12	10	1.47 [-0.53 to 3.47]	4.38	U	U	H	U	L	L
Ngo Sock et al. 2010	11	11	0.00 [-1.39 to 1.39]	8.69	U	U	L	U	U	L
Schwarz et al. 2015	7	7	1.15 [-0.64 to 2.93]	5.45	H	H	L	L	L	H
Silbernagel et al. 2011	10	10	-0.02 [-1.41 to 1.37]	8.69	L	L	L	U	U	L
Heterogeneity: τ^2 = 0.00, I^2 = 0.00%, H^2 = 1.00			0.19 [-0.38 to 0.76]							
Test of $\theta_i = \theta_j$: Q(6) = 3.79, p = 0.71										
**06. Fruit**										
Agebratt et al. 2016	15	15	0.36 [-1.07 to 1.79]	8.20	L	U	L	L	L	L
Heterogeneity: τ^2 = 0.00, I^2 = .%, H^2 = .			0.36 [-1.07 to 1.79]							
Test of $\theta_i = \theta_j$: Q(0) = 0.00, p = .										
**10. Sweets and desserts**										
Dikariyanto et al. 2020	51	54	1.63 [-0.48 to 3.75]	3.94	L	U	L	U	L	L
Heterogeneity: τ^2 = 0.00, I^2 = .%, H^2 = .			1.63 [-0.48 to 3.75]							
Test of $\theta_i = \theta_j$: Q(0) = 0.00, p = .										
**12. Added nutritive (caloric) sweetener**										
Simons et al. 2020	16	21	1.39 [-0.55 to 3.34]	4.63	L	L	L	L	L	L
Heterogeneity: τ^2 = 0.00, I^2 = .%, H^2 = .			1.39 [-0.55 to 3.34]							
Test of $\theta_i = \theta_j$: Q(0) = 0.00, p = .										
**13. Mixed sources (with SSBs)**										
Luukkonen et al. 2018 (SAT)	14	12	-0.44 [-1.89 to 1.01]	8.00	U	U	U	L	L	L
Luukkonen et al. 2018 (UNSAT)	12	12	0.28 [-1.13 to 1.69]	8.40	U	U	U	L	L	L
Parry et al. 2020	16	16	-1.00 [-2.69 to 0.70]	5.99	L	L	U	U	L	L
Schwimmer et al. 2019	20	20	3.60 [-0.19 to 7.39]	1.26	L	L	L	L	L	L
Umpleby et al. 2017 (H)	14	14	1.78 [-0.45 to 4.01]	3.57	L	L	L	U	L	L
Umpleby et al. 2017 (NAFLD)	11	11	1.70 [-0.47 to 3.86]	3.77	L	L	L	U	L	L
Heterogeneity: τ^2 = 0.81, I^2 = 47.08%, H^2 = 1.89			0.52 [-0.56 to 1.60]							
Test of $\theta_i = \theta_j$: Q(5) = 9.45, p = 0.09										
**Overall**			0.36 [-0.07 to 0.79]							
Heterogeneity: τ^2 = 0.05, I^2 = 6.70%, H^2 = 1.07										
Test of $\theta_i = \theta_j$: Q(15) = 16.08, p = 0.38										
Test of group differences: $Q_b(4)$ = 2.89, p = 0.58										

Risk of bias: A–F

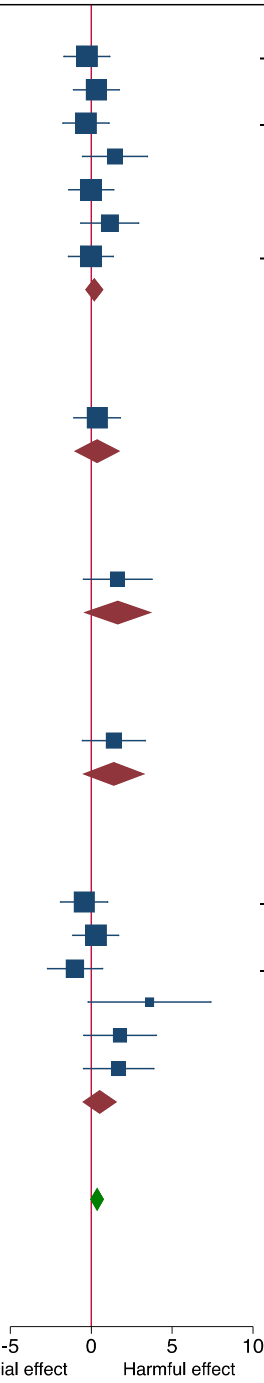

Test of θ = 0: z = 1.656, p = 0.098

Pooled effect estimates for each subgroup and overall effect are represented by the diamonds. Data are expressed as weighted mean differences with 95% confidence intervals using the generic inverse-variance method and random effects DerSimonian-Laird model. Paired analyses were applied to all crossover trials. Inter-study heterogeneity was

assessed using the Cochrane Q statistic and quantified using the I^2 statistic, with significance set at p<0.100 and $I^2 \geq 50\%$ considered to be evidence of substantial heterogeneity.
Risk of Bias Legend: (H) High Risk; (L) Low Risk; (U) Unclear. The letters represent the following risk of bias domains: A, random sequence generation (selection bias); B, allocation concealment (selection bias); C, blinding of participants and personnel and outcome assessors (performance bias); D, incomplete outcome data (attrition bias); E, selective reporting (reporting bias); and F, other bias. High other risk of bias (carry-over effect) was given to crossover trials which had no washout between interventions. Trials which did not have this characteristic were rated as Low.
Pooled effect summary calculated with the χ^2 test. Test for group differences calculated with meta-regression, which uses the Wald test.
To convert SMD to %liver fat, multiply the SMD by the baseline pooled standard deviation, 0.71%.
CI=confidence interval; H=healthy; IHCL=intrahepatocellular lipid; M=milk; NAFLD=non-alcoholic fatty liver disease; SMD=standardized mean difference; SSB=sugar-sweetened beverage; SAT=saturated fat; T1=test group 1; T2=test group 2; UNSAT=unsaturated fat.

Supplementary Figure S13: Forest plot of controlled trials of the effect of important food sources of fructose-containing sugars and IHCL (SMD) in addition trials

IHCL (SMD)

Addition trials

Trial or subgroup	N Control	N Intervention	Mean difference with 95% CI	Weight (%)	A	B	C	D	E	F
**01. SSB**										
Johnston et al. 2013	17	15	2.08 [-0.39 to 4.54]	6.75	H	H	L	L	L	H
Koopman et al. 2014 (HS-F)	5	8	1.42 [-0.54 to 3.38]	10.60	L	H	U	L	L	L
Koopman et al. 2014 (HS-S)	5	7	0.71 [-0.84 to 2.26]	17.01	L	H	U	L	L	L
Lê et al. 2006	7	7	1.26 [-0.60 to 3.12]	11.87	H	H	U	U	U	H
Lê et al. 2009 (H)	8	8	2.54 [-0.31 to 5.38]	5.05	U	U	L	U	L	L
Lê et al. 2009 (ODM2)	16	16	4.06 [-0.15 to 8.27]	2.31	U	U	U	U	L	L
Maersk et al. 2012 (NSB)	12	10	1.56 [-0.50 to 3.62]	9.60	U	U	H	U	L	L
Maersk et al. 2012 (Water)	13	10	2.69 [-0.29 to 5.67]	4.61	U	U	H	U	L	L
Ngo Sock et al. 2010	11	11	1.59 [-0.50 to 3.67]	9.40	U	U	L	U	U	L
Sigala et al. 2021 (HFCS)	28	23	2.54 [-0.31 to 5.40]	5.02	H	H	L	U	L	L
Sigala et al. 2021 (Sucrose)	24	23	3.73 [-0.18 to 7.64]	2.68	H	H	L	U	L	L
Silbernagel et al. 2011	10	10	1.75 [-0.46 to 3.95]	8.41	H	H	L	U	U	H
Sobrecases et al. 2010	30	30	2.09 [-0.38 to 4.56]	6.69	H	H	U	U	U	H
Heterogeneity: $\tau^2 = 0.00$, $I^2 = 0.00\%$, $H^2 = 1.00$			1.72 [1.08 to 2.36]							
Test of $\theta_i = \theta_j$: Q(12) = 5.41, p = 0.94										
**Overall**			1.72 [1.08 to 2.36]							
Heterogeneity: $\tau^2 = 0.00$, $I^2 = 0.00\%$, $H^2 = 1.00$										
Test of $\theta_i = \theta_j$: Q(12) = 5.41, p = 0.94										
Test of group differences: $Q_b(0) = -0.00$, p = .										

Risk of bias

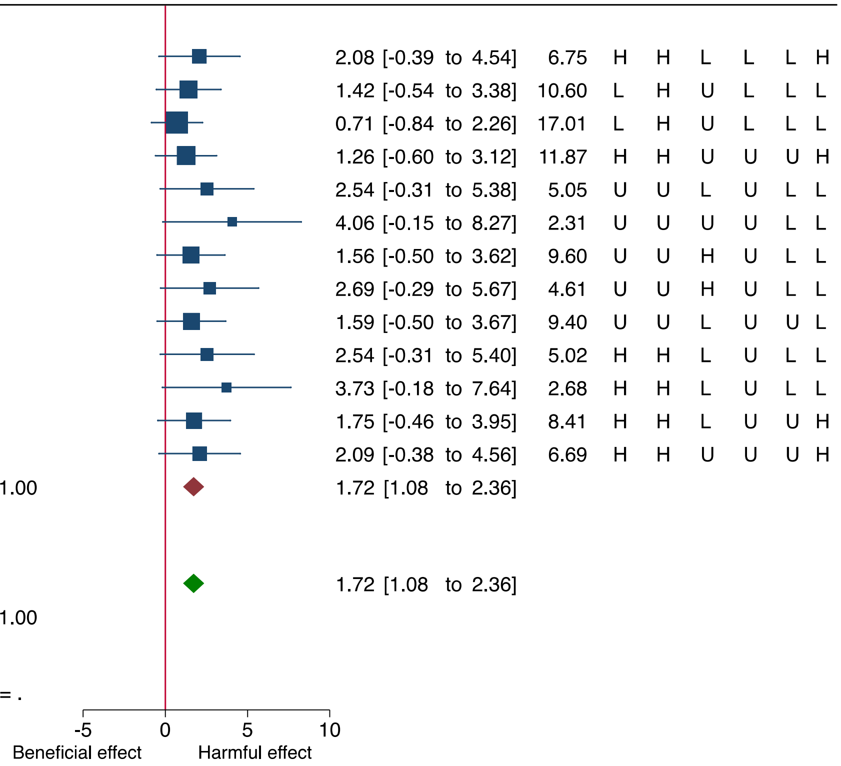

Test of θ = 0: z = 5.272, p = 0.000

Pooled effect estimates for each subgroup and overall effect are represented by the diamonds. Data are expressed as weighted mean differences with 95% confidence intervals using the generic inverse-variance method and random effects DerSimonian-Laird model. Paired analyses were applied to all crossover trials. Inter-study heterogeneity was assessed using the Cochrane Q statistic and quantified using the I^2 statistic, with significance set at p<0.100 and $I^2 \geq 50\%$ considered to be evidence of substantial heterogeneity.

Risk of Bias Legend: (H) High Risk; (L) Low Risk; (U) Unclear. The letters represent the following risk of bias domains: A, random sequence generation (selection bias); B, allocation concealment (selection bias); C, blinding of participants and personnel and outcome assessors (performance bias); D, incomplete outcome data (attrition bias); E, selective reporting (reporting bias); and F, other (carry-over effect) bias. High other risk of bias was given to crossover trials which had no washout between interventions. Trials which did not have this characteristic were rated as Low.

Pooled effect summary calculated with the χ^2 test. Test for group differences calculated with meta-regression, which uses the Wald test.

To convert SMD to %liver fat, multiply the SMD by the baseline pooled standard deviation, 0.71%.

CI=confidence interval; H=healthy; HS-F=high sucrose-frequency; HS-S=high sucrose-size; IHCL=intrahepatocellular lipid; NSB=non-nutritive sweetened beverage; ODM2=offspring of type-2 diabetes patients; SMD=standardized mean difference; SSB=sugar-sweetened beverage.

Supplementary Figure S14: Forest plot of controlled trials of the effect of important food sources of fructose-containing sugars and IHCL (SMD) in subtraction trials

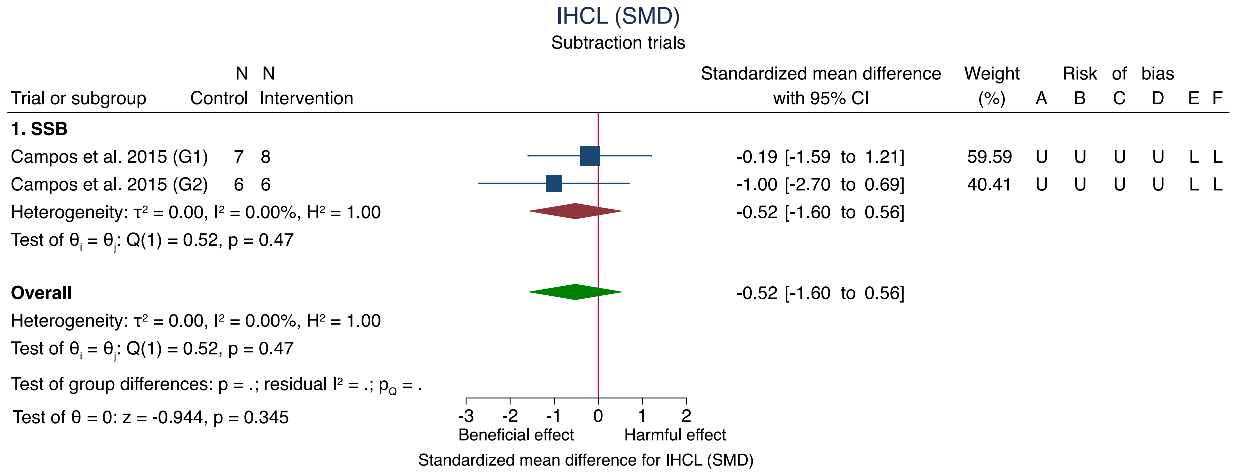

Pooled effect estimates for each subgroup and overall effect are represented by the diamonds. Data are expressed as weighted mean differences with 95% confidence intervals using the generic inverse-variance method and random effects DerSimonian-Laird model. Paired analyses were applied to all crossover trials. Inter-study heterogeneity was assessed using the Cochrane Q statistic and quantified using the I^2 statistic, with significance set at $p<0.100$ and $I^2 \geq 50\%$ considered to be evidence of substantial heterogeneity.

Risk of Bias Legend: (H) High Risk; (L) Low Risk; (U) Unclear. The letters represent the following risk of bias domains: A, random sequence generation (selection bias); B, allocation concealment (selection bias); C, blinding of participants and personnel and outcome assessors (performance bias); D, incomplete outcome data (attrition bias); E, selective reporting (reporting bias); and F, other (carry-over effect) bias. High other risk of bias was given to crossover trials which had no washout between interventions. Trials which did not have this characteristic were rated as Low.

Pooled effect summary calculated with the χ^2 test. test for group differences calculated with meta-regression, which uses the Wald test.

To convert SMD to %liver fat, multiply the SMD by the baseline pooled standard deviation, 0.71%.

CI=confidence interval; G1=group 1; G2=group 2; IHCL=intrahepatocellular lipid; SSB=sugar-sweetened beverage.

Supplementary Figure S15: Forest plot of controlled trials of the effect of important food sources of fructose-containing sugars and ALT (U/L) in substitution trials

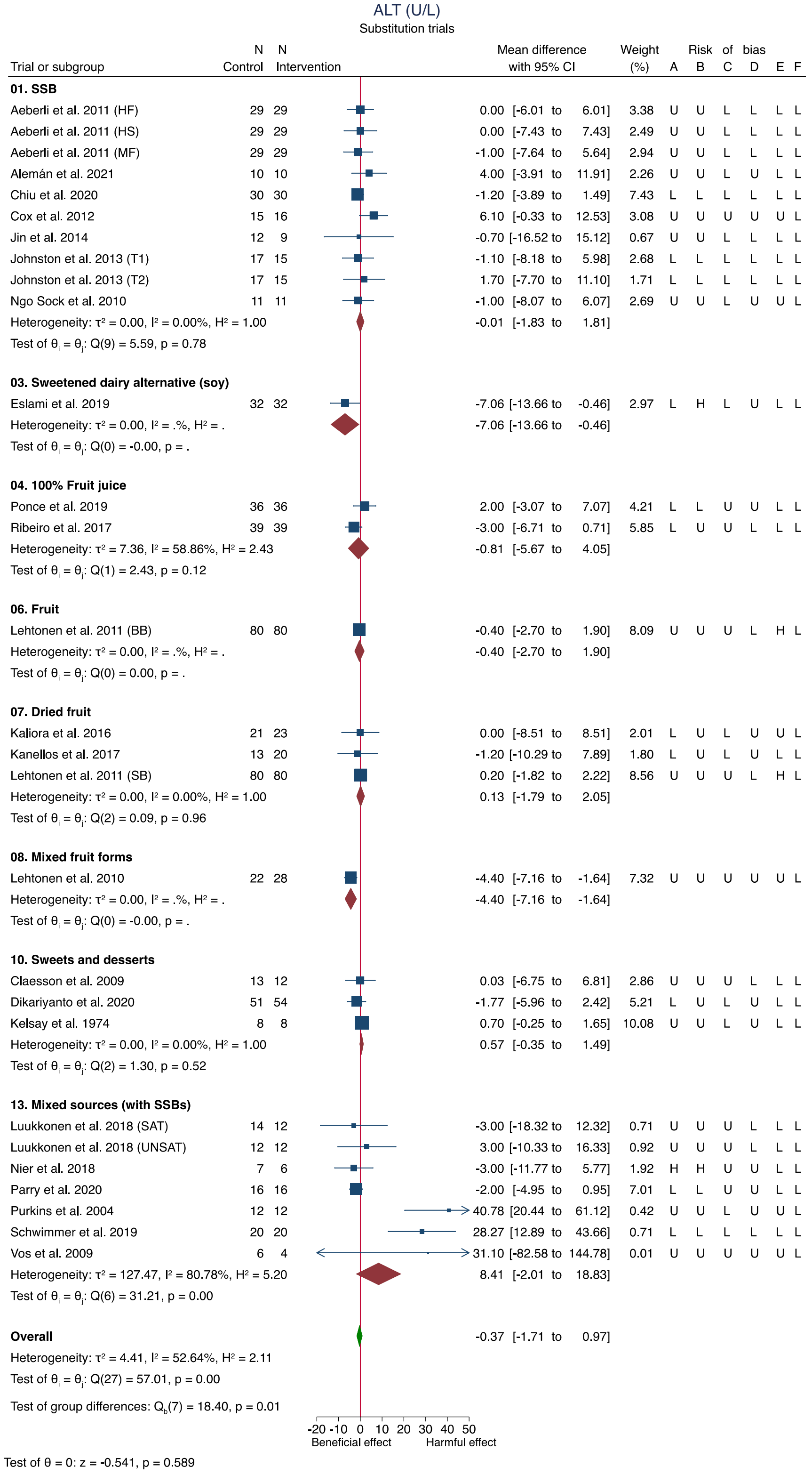

Pooled effect estimates for each subgroup and overall effect are represented by the diamonds. Data are expressed as weighted mean differences with 95% confidence intervals using the generic inverse-variance method and random

effects DerSimonian-Laird model. Paired analyses were applied to all crossover trials. Inter-study heterogeneity was assessed using the Cochrane Q statistic and quantified using the I^2 statistic, with significance set at $p<0.100$ and $I^2 \geq 50\%$ considered to be evidence of substantial heterogeneity.
Risk of Bias Legend: (H) High Risk; (L) Low Risk; (U) Unclear. The letters represent the following risk of bias domains: A, random sequence generation (selection bias); B, allocation concealment (selection bias); C, blinding of participants and personnel and outcome assessors (performance bias); D, incomplete outcome data (attrition bias); E, selective reporting (reporting bias); and F, other (carry-over effect) bias. High other risk of bias was given to crossover trials which had no washout between interventions. Trials which did not have this characteristic were rated as Low.
Pooled effect summary calculated with the χ^2 test. Test for group differences calculated with meta-regression, which uses the Wald test.
To convert U/L to ukat/L, multiply U/L by 0.0167.
ALT=alanine aminotransferase; BB=bilberries; CI=confidence interval; HF=high fructose; HS=high sucrose; MF=medium fructose; SAT=saturated fat; SB=seabuckthorn berry; SSB=sugar-sweetened beverage; T1=test group 1; T2=test group 2; UNSAT=unsaturated fat.

Supplementary Figure S16: Forest plot of controlled trials of the effect of important food sources of fructose-containing sugars and ALT (U/L) in addition trials

ALT (U/L)
Addition trials

Trial or subgroup	N Control	N Intervention	Mean difference with 95% CI	Weight (%)	A	B	C	D	E	F
**01. SSB**										
Aeberli et al. 2011 (HF)	29	29	2.00 [-3.39 to 7.39]	3.92	U	U	L	L	L	L
Aeberli et al. 2011 (HS)	29	29	2.00 [-5.16 to 9.16]	2.58	U	U	L	L	L	L
Aeberli et al. 2011 (MF)	29	29	2.00 [-4.21 to 8.21]	3.21	U	U	L	L	L	L
Debray et al. 2021 (Ctrl)	6	6	-4.96 [-13.12 to 3.20]	2.09	U	U	U	U	L	L
Debray et al. 2021 (hHFI)	6	6	-2.78 [-7.83 to 2.27]	4.26	U	U	U	U	L	L
Johnston et al. 2013	17	15	8.70 [3.32 to 14.08]	3.92	H	H	L	L	L	H
Lê et al. 2009 (H)	8	8	8.00 [0.66 to 15.34]	2.48	U	U	L	U	L	L
Lê et al. 2009 (ODM2)	16	16	12.90 [5.45 to 20.35]	2.43	U	U	L	U	L	L
Ngo Sock et al. 2010	11	11	4.00 [-1.19 to 9.19]	4.12	U	U	L	U	U	L
Sobrecases et al. 2010	8	8	4.66 [-5.41 to 14.73]	1.46	H	H	U	U	U	L
Zafrilla et al. 2021 (Stevia)	45	45	4.00 [0.28 to 7.72]	6.04	U	L	L	L	L	L
Zafrilla et al. 2021 (Sucralose)	45	46	-1.00 [-4.93 to 2.93]	5.71	U	L	L	L	L	L
Heterogeneity: $\tau^2 = 11.50$, $I^2 = 58.18\%$, $H^2 = 2.39$			3.09 [0.49 to 5.68]							
Test of $\theta_i = \theta_j$: Q(11) = 26.30, p = 0.01										
**04. 100% Fruit juice**										
Amagase et al. 2009	30	30	-0.64 [-6.65 to 5.37]	3.36	U	L	L	L	U	L
Banini et al. 2006	15	8	0.70 [-11.44 to 12.84]	1.05	U	U	U	L	U	L
Kojadinovic et al. 2021	12	12	-7.31 [-19.56 to 4.94]	1.03	U	U	U	L	U	L
Ravn-Haren et al. 2013 (Cld vs Ctrl)	23	23	-1.52 [-5.00 to 1.96]	6.44	U	U	L	U	U	H
Ravn-Haren et al. 2013 (Cld vs Pom)	23	23	-0.34 [-3.68 to 3.00]	6.68	U	U	L	U	U	H
Ravn-Haren et al. 2013 (Clr vs Ctrl)	23	23	-1.11 [-4.71 to 2.49]	6.23	U	U	L	U	U	H
Ravn-Haren et al. 2013 (Clr vs Pom)	23	23	0.07 [-3.41 to 3.55]	6.44	U	U	L	U	U	H
Heterogeneity: $\tau^2 = 0.00$, $I^2 = 0.00\%$, $H^2 = 1.00$			-0.80 [-2.43 to 0.84]							
Test of $\theta_i = \theta_j$: Q(6) = 1.65, p = 0.95										
**06. Fruit**										
Ravn-Haren et al. 2013 (Ctrl)	23	23	0.27 [-4.32 to 4.86]	4.79	U	U	L	U	U	H
Ravn-Haren et al. 2013 (Pom)	23	23	1.45 [-3.09 to 5.99]	4.86	U	U	L	U	U	H
Schell et al. 2019	22	22	-1.20 [-7.98 to 5.58]	2.81	U	U	L	L	L	L
Tutino et al. 2021	19	21	0.22 [-6.25 to 6.69]	3.01	L	U	U	L	L	L
Heterogeneity: $\tau^2 = 0.00$, $I^2 = 0.00\%$, $H^2 = 1.00$			0.44 [-2.22 to 3.10]							
Test of $\theta_i = \theta_j$: Q(3) = 0.42, p = 0.94										
**07. Dried fruit**										
Ahmed et al. 2010	35	35	-2.74 [-18.48 to 13.00]	0.65	U	U	U	U	U	L
Irannejad et al. 2020	36	36	-1.18 [-46.99 to 44.63]	0.08	U	L	L	U	L	L
Heterogeneity: $\tau^2 = 0.00$, $I^2 = 0.00\%$, $H^2 = 1.00$			-2.57 [-17.46 to 12.31]							
Test of $\theta_i = \theta_j$: Q(1) = 0.00, p = 0.95										
**10. Sweets and desserts**										
Alavinejad et al. 2015	21	21	-2.15 [-20.93 to 16.63]	0.46	U	L	L	U	L	L
Heterogeneity: $\tau^2 = 0.00$, $I^2 = .\%$, $H^2 = .$			-2.15 [-20.93 to 16.63]							
Test of $\theta_i = \theta_j$: Q(0) = 0.00, p = .										
**11. Honey**										
Bahrami et al. 2009	23	25	0.10 [-3.14 to 3.34]	6.86	U	U	U	U	U	L
Tang et al. 2020 (HD)	23	22	10.53 [-20.55 to 41.61]	0.17	L	U	L	U	U	L
Tang et al. 2020 (LD)	23	26	13.60 [-17.49 to 44.69]	0.17	L	U	L	U	U	L
Tang et al. 2020 (MedD)	23	24	36.89 [-26.50 to 100.28]	0.04	L	U	L	U	U	L
Zakaria et al. 2018	36	36	-5.56 [-12.60 to 1.48]	2.65	L	U	U	L	U	L
Heterogeneity: $\tau^2 = 5.05$, $I^2 = 15.28\%$, $H^2 = 1.18$			-0.87 [-5.33 to 3.59]							
Test of $\theta_i = \theta_j$: Q(4) = 4.72, p = 0.32										
**Overall**			0.91 [-0.39 to 2.21]							
Heterogeneity: $\tau^2 = 3.69$, $I^2 = 31.44\%$, $H^2 = 1.46$										
Test of $\theta_i = \theta_j$: Q(30) = 43.76, p = 0.05										
Test of group differences: $Q_b(5) = 6.62$, p = 0.25										

Axis: -20 -10 0 10 20 30 40
Beneficial effect | Harmful effect

Test of $\theta = 0$: z = 1.374, p = 0.169

Pooled effect estimates for each subgroup and overall effect are represented by the diamonds. Data are expressed as weighted mean differences with 95% confidence intervals using the generic inverse-variance method and random

effects DerSimonian-Laird model. Paired analyses were applied to all crossover trials. Inter-study heterogeneity was assessed using the Cochrane Q statistic and quantified using the I^2 statistic, with significance set at p<0.100 and $I^2 \geq 50\%$ considered to be evidence of substantial heterogeneity.
Risk of Bias Legend: (H) High Risk; (L) Low Risk; (U) Unclear. The letters represent the following risk of bias domains: A, random sequence generation (selection bias); B, allocation concealment (selection bias); C, blinding of participants and personnel and outcome assessors (performance bias); D, incomplete outcome data (attrition bias); E, selective reporting (reporting bias); and F, other (carry-over effect) bias. High other risk of bias was given to crossover trials which had no washout between interventions. Trials which did not have this characteristic were rated as Low.
Pooled effect summary calculated with the χ^2 test. Test for group differences calculated with meta-regression, which uses the Wald test.
To convert U/L to ukat/L, multiply U/L by 0.0167.
ALT=alanine aminotransferase; CI=confidence interval; Cld=cloudy apple juice; Clr=clear apple juice; Ctrl=control group; H=healthy; HD=high dose; HF=high fructose; hHFI=heterozygote high fructose intolerance; HS=high sucrose; LD=low dose; MD=mean difference; MedD=medium dose; MF=medium fructose; ODM2=offspring of type-2 diabetes patients; Pom=apple pomace; SSB=sugar-sweetened beverage.

Supplementary Figure S17: Forest plot of controlled trials of the effect of important food sources of fructose-containing sugars and ALT (U/L) in subtraction trials

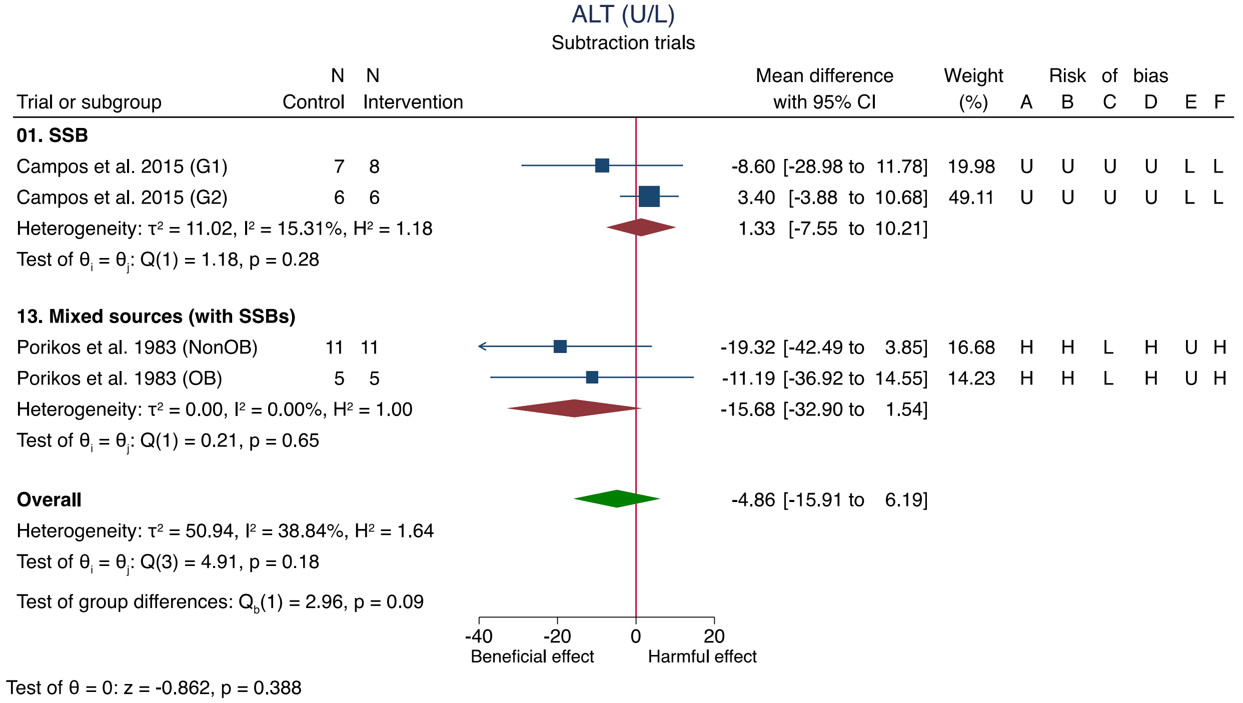

Pooled effect estimates for each subgroup and overall effect are represented by the diamonds. Data are expressed as weighted mean differences with 95% confidence intervals using the generic inverse-variance method and random effects DerSimonian-Laird model. Paired analyses were applied to all crossover trials. Inter-study heterogeneity was assessed using the Cochrane Q statistic and quantified using the I^2 statistic, with significance set at p<0.100 and $I^2 \geq 50\%$ considered to be evidence of substantial heterogeneity.

Risk of Bias Legend: (H) High Risk; (L) Low Risk; (U) Unclear. The letters represent the following risk of bias domains: A, random sequence generation (selection bias); B, allocation concealment (selection bias); C, blinding of participants and personnel and outcome assessors (performance bias); D, incomplete outcome data (attrition bias); E, selective reporting (reporting bias); and F, other (carry-over effect) bias. High other risk of bias was given to crossover trials which had no washout between interventions. Trials which did not have this characteristic were rated as Low.

Pooled effect summary calculated with the χ^2 test. Test for group differences calculated with meta-regression, which uses the Wald test.

To convert U/L to ukat/L, multiply U/L by 0.0167.

ALT=alanine aminotransferase; CI=confidence interval; G1=group 1; G2=group 2; NonOB=non-obese; OB=obese.

Supplementary Figure S18: Forest plot of controlled trials of the effect of important food sources of fructose-containing sugars and ALT (U/L) in *ad libitum* trials

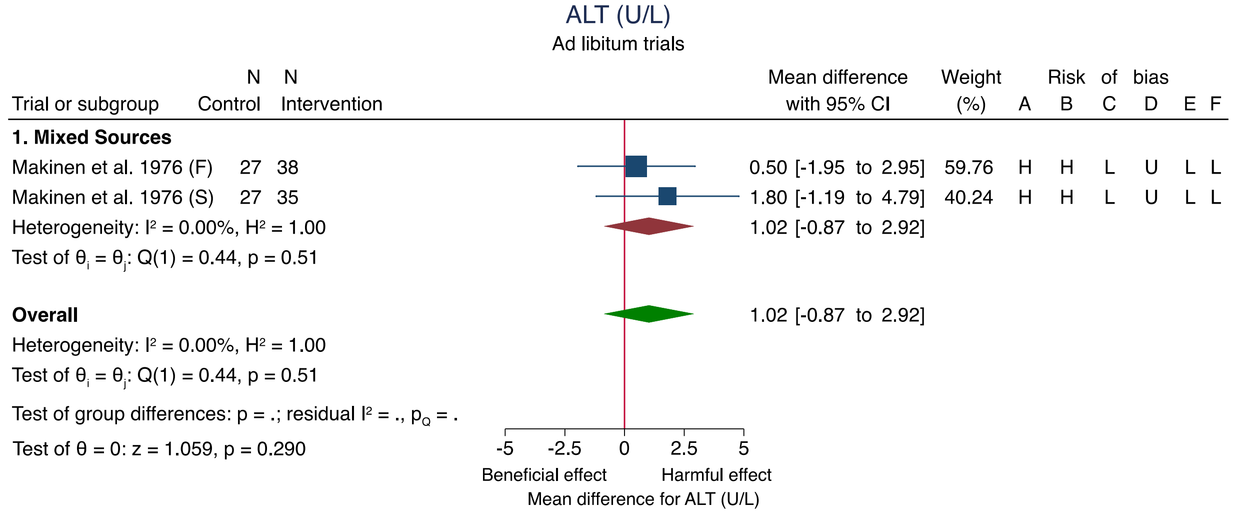

Pooled effect estimates for each subgroup and overall effect are represented by the diamonds. Data are expressed as weighted mean differences with 95% confidence intervals using the generic inverse-variance method and random effects DerSimonian-Laird model. Paired analyses were applied to all crossover trials. Inter-study heterogeneity was assessed using the Cochrane Q statistic and quantified using the I^2 statistic, with significance set at $p<0.100$ and $I^2 \geq 50\%$ considered to be evidence of substantial heterogeneity.

Risk of Bias Legend: (H) High Risk; (L) Low Risk; (U) Unclear. The letters represent the following risk of bias domains: A, random sequence generation (selection bias); B, allocation concealment (selection bias); C, blinding of participants and personnel and outcome assessors (performance bias); D, incomplete outcome data (attrition bias); E, selective reporting (reporting bias); and F, other (carry-over effect) bias. High other risk of bias was given to crossover trials which had no washout between interventions. Trials which did not have this characteristic were rated as Low.

Pooled effect summary calculated with the χ^2 test. Test for group differences calculated with meta-regression, which uses the Wald test.

To convert U/L to ukat/L, multiply U/L by 0.0167.

ALT=alanine aminotransferase; CI=confidence interval; F=fructose; S=sucrose.

Supplementary Figure S19: Forest plot of controlled trials of the effect of important food sources of fructose-containing sugars and AST (U/L) in substitution trials

AST (U/L)

Substitution trials

Trial or subgroup	N Control	N Intervention	Mean difference with 95% CI	Weight (%)	A	B	C	D	E	F
**01. SSB**										
Aeberli et al. 2011 (HF)	29	29	-1.00 [-5.55 to 3.55]	4.68	U	U	L	L	L	L
Aeberli et al. 2011 (HS)	29	29	1.00 [-4.00 to 6.00]	4.15	U	U	L	L	L	L
Aeberli et al. 2011 (MF)	29	29	-1.00 [-5.13 to 3.13]	5.23	U	U	L	L	L	L
Alemán et al. 2021	10	10	2.00 [-6.83 to 10.83]	1.74	U	U	L	U	L	L
Chiu et al. 2020	30	30	-1.50 [-3.17 to 0.17]	9.86	L	L	L	L	L	L
Cox et al. 2012	15	16	3.30 [-1.53 to 8.13]	4.35	U	U	U	U	U	L
Jin et al. 2014	12	9	1.90 [-3.94 to 7.74]	3.36	U	U	L	L	L	L
Johnston et al. 2013 (T1)	17	15	-2.50 [-6.71 to 1.71]	5.13	L	L	L	L	L	L
Johnston et al. 2013 (T2)	17	15	-0.90 [-6.18 to 4.38]	3.87	L	L	L	L	L	L
Ngo Sock et al. 2010	11	11	0.00 [-3.39 to 3.39]	6.40	U	U	L	U	U	L
Heterogeneity: τ^2 = 0.00, I^2 = 0.00%, H^2 = 1.00			-0.72 [-1.86 to 0.42]							
Test of $\theta_i = \theta_j$: Q(9) = 5.99, p = 0.74										
**03. Sweetened dairy alternative (soy)**										
Eslami et al. 2019	32	32	2.25 [-2.33 to 6.83]	4.64	L	H	L	U	L	L
Heterogeneity: τ^2 = 0.00, I^2 = .%, H^2 = .			2.25 [-2.33 to 6.83]							
Test of $\theta_i = \theta_j$: Q(0) = 0.00, p = .										
**04. 100% Fruit juice**										
Ponce et al. 2019	36	36	1.00 [-2.87 to 4.87]	5.63	L	L	U	U	L	L
Ribeiro et al. 2017	39	39	-1.00 [-4.20 to 2.20]	6.75	L	U	U	L	L	L
Heterogeneity: τ^2 = 0.00, I^2 = 0.00%, H^2 = 1.00			-0.19 [-2.65 to 2.28]							
Test of $\theta_i = \theta_j$: Q(1) = 0.61, p = 0.43										
**07. Dried fruit**										
Kaliora et al. 2016	21	23	0.40 [-3.17 to 3.97]	6.11	L	U	L	U	U	L
Kanellos et al. 2017	13	20	0.60 [-6.69 to 7.89]	2.40	L	U	L	U	L	L
Heterogeneity: τ^2 = 0.00, I^2 = 0.00%, H^2 = 1.00			0.44 [-2.76 to 3.64]							
Test of $\theta_i = \theta_j$: Q(1) = 0.00, p = 0.96										
**10. Sweets and desserts**										
Claesson et al. 2009	13	12	1.46 [-2.33 to 5.25]	5.74	U	U	U	L	U	L
Kelsay et al. 1974	8	8	-0.80 [-2.30 to 0.70]	10.20	U	U	L	U	L	L
Heterogeneity: τ^2 = 0.39, I^2 = 15.10%, H^2 = 1.18			-0.37 [-2.11 to 1.37]							
Test of $\theta_i = \theta_j$: Q(1) = 1.18, p = 0.28										
**13. Mixed sources (with SSBs)**										
Luukkonen et al. 2018 (SAT)	14	12	-2.30 [-8.97 to 4.37]	2.76	U	U	U	L	L	L
Luukkonen et al. 2018 (UNSAT)	12	12	1.25 [-5.75 to 8.25]	2.56	U	U	U	L	L	L
Nier et al. 2018	7	6	-9.00 [-19.37 to 1.37]	1.32	H	H	U	U	L	L
Purkins et al. 2004	12	12	23.78 [12.92 to 34.64]	1.21	U	U	L	U	U	L
Schwimmer et al. 2019	20	20	13.04 [4.46 to 21.61]	1.83	L	L	L	L	L	L
Vos et al. 2009	6	4	6.20 [-46.26 to 58.66]	0.06	U	U	U	U	U	L
Heterogeneity: τ^2 = 99.46, I^2 = 81.51%, H^2 = 5.41			5.11 [-4.34 to 14.55]							
Test of $\theta_i = \theta_j$: Q(5) = 27.04, p = 0.00										
**Overall**			0.39 [-0.87 to 1.65]							
Heterogeneity: τ^2 = 3.48, I^2 = 46.64%, H^2 = 1.87										
Test of $\theta_i = \theta_j$: Q(22) = 41.23, p = 0.01										
Test of group differences: $Q_b(5)$ = 3.18, p = 0.67										

(Risk of bias: A–F)

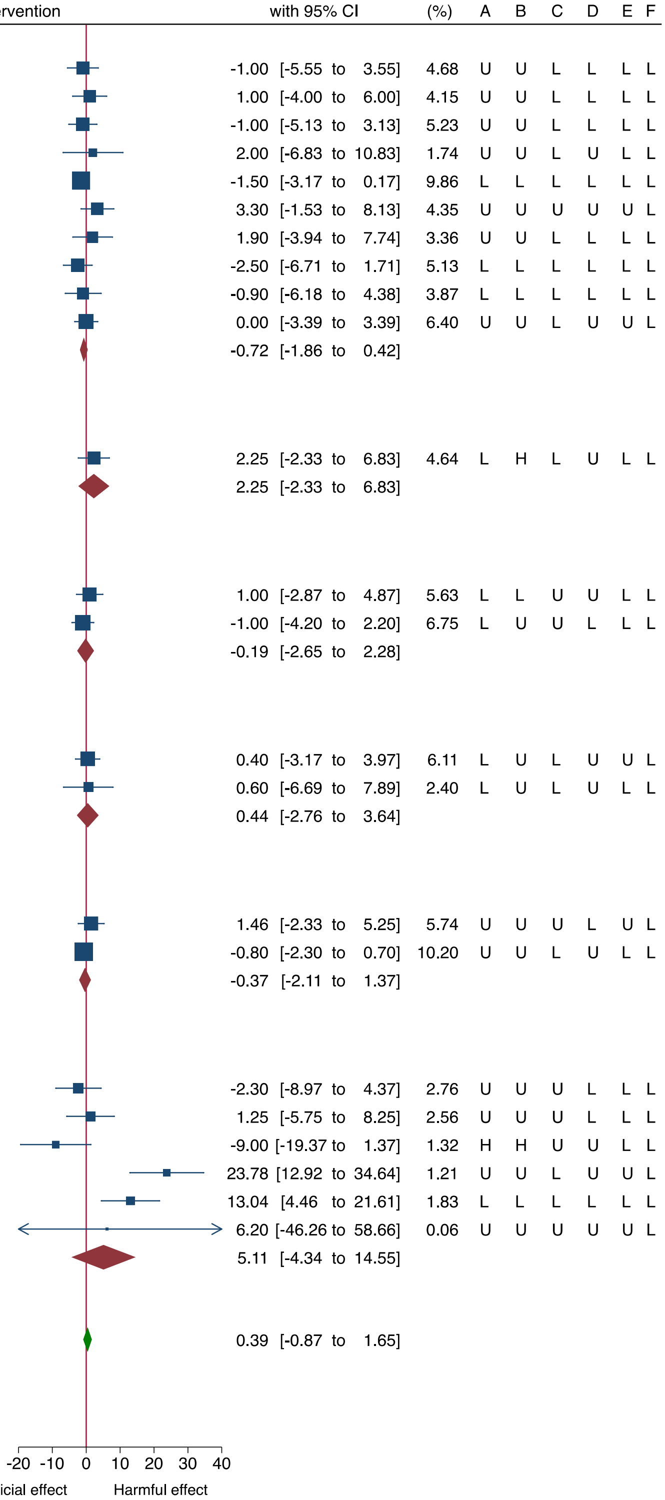

Test of θ = 0: z = 0.604, p = 0.546

Pooled effect estimates for each subgroup and overall effect are represented by the diamonds. Data are expressed as weighted mean differences with 95% confidence intervals using the generic inverse-variance method and random

effects DerSimonian-Laird model. Paired analyses were applied to all crossover trials. Inter-study heterogeneity was assessed using the Cochrane Q statistic and quantified using the I^2 statistic, with significance set at $p<0.100$ and $I^2 \geq 50\%$ considered to be evidence of substantial heterogeneity.
Risk of Bias Legend: (H) High Risk; (L) Low Risk; (U) Unclear. The letters represent the following risk of bias domains: A, random sequence generation (selection bias); B, allocation concealment (selection bias); C, blinding of participants and personnel and outcome assessors (performance bias); D, incomplete outcome data (attrition bias); E, selective reporting (reporting bias); and F, other (carry-over effect) bias. High other risk of bias was given to crossover trials which had no washout between interventions. Trials which did not have this characteristic were rated as Low.
Pooled effect summary calculated with the χ^2 test. Test for group differences calculated with meta-regression, which uses the Wald test.
To convert U/L to ukat/L, multiply U/L by 0.0167.
AST=aspartate aminotransferase; CI=confidence interval; SSB=sugar-sweetened beverage; HF=high fructose; HS=high sucrose; MF=medium fructose; SAT=saturated fat; SSB=sugar-sweetened beverage; T1=test group 1; T2=test group 2; UNSAT=unsaturated fat.

Supplementary Figure S20: Forest plot of controlled trials of the effect of important food sources of fructose-containing sugars and AST (U/L) in addition trials

AST (U/L)

Addition trials

Trial or subgroup	N Control	N Intervention	Mean difference with 95% CI	Weight (%)	Risk of bias A	B	C	D	E	F
**01. SSB**										
Aeberli et al. 2011 (HF)	29	29	-1.00 [-5.76 to 3.76]	2.63	U	U	L	L	L	L
Aeberli et al. 2011 (HS)	29	29	1.00 [-4.16 to 6.16]	2.25	U	U	L	L	L	L
Aeberli et al. 2011 (MF)	29	29	-1.00 [-5.13 to 3.13]	3.44	U	U	L	L	L	L
Debray et al. 2021 (Ctrl)	6	6	-2.13 [-6.60 to 2.34]	2.97	U	U	U	U	L	L
Debray et al. 2021 (hHFI)	6	6	-2.27 [-5.37 to 0.83]	5.85	U	U	U	U	L	L
Johnston et al. 2013	17	15	1.60 [-1.12 to 4.32]	7.39	H	H	L	L	L	H
Ngo Sock et al. 2010	11	11	1.00 [-0.96 to 2.96]	12.85	U	U	L	U	U	L
Zafrilla et al. 2021 (Stevia)	45	45	4.00 [0.85 to 7.15]	5.69	U	L	L	L	L	L
Zafrilla et al. 2021 (Sucralose)	45	46	-1.00 [-4.32 to 2.32]	5.17	U	L	L	L	L	L
Heterogeneity: τ^2 = 1.30, I^2 = 31.62%, H^2 = 1.46			0.29 [-1.07 to 1.64]							
Test of $\theta_i = \theta_j$: Q(8) = 11.70, p = 0.17										
**04. 100% Fruit juice**										
Amagase et al. 2009	30	30	-0.20 [-3.55 to 3.15]	5.08	U	L	L	L	U	L
Banini et al. 2006	15	8	4.20 [-3.55 to 11.95]	1.02	U	U	U	L	U	L
Kojadinovic et al. 2021	12	12	-1.48 [-6.98 to 4.02]	1.99	U	U	U	L	U	L
Heterogeneity: τ^2 = 0.00, I^2 = 0.00%, H^2 = 1.00			0.02 [-2.66 to 2.71]							
Test of $\theta_i = \theta_j$: Q(2) = 1.42, p = 0.49										
**06. Fruit**										
Schell et al. 2019	22	22	-3.30 [-10.87 to 4.27]	1.07	U	U	L	L	L	L
Tutino et al. 2021	19	21	-1.19 [-4.88 to 2.50]	4.25	L	U	U	L	L	L
Heterogeneity: τ^2 = 0.00, I^2 = 0.00%, H^2 = 1.00			-1.60 [-4.91 to 1.72]							
Test of $\theta_i = \theta_j$: Q(1) = 0.24, p = 0.62										
**07. Dried fruit**										
Ahmed et al. 2010	35	35	-7.77 [-21.06 to 5.52]	0.35	U	U	U	H	U	L
Irannejad et al. 2020	36	36	-2.94 [-15.59 to 9.71]	0.39	U	L	L	U	L	L
Heterogeneity: τ^2 = 0.00, I^2 = 0.00%, H^2 = 1.00			-5.24 [-14.40 to 3.93]							
Test of $\theta_i = \theta_j$: Q(1) = 0.27, p = 0.61										
**10. Sweets and desserts**										
Alavinejad et al. 2015	21	21	-6.08 [-14.50 to 2.34]	0.86	U	L	L	U	L	L
Heterogeneity: τ^2 = 0.00, I^2 = .%, H^2 = .			-6.08 [-14.50 to 2.34]							
Test of $\theta_i = \theta_j$: Q(0) = -0.00, p = .										
**11. Honey**										
Bahrami et al. 2009	23	25	-0.20 [-1.04 to 0.64]	36.57	U	U	U	U	U	L
Tang et al. 2020 (HD)	23	22	10.60 [-17.86 to 39.06]	0.08	L	U	L	U	U	L
Tang et al. 2020 (LD)	23	26	15.10 [-12.23 to 42.43]	0.08	L	U	L	U	U	L
Tang et al. 2020 (MD)	23	24	23.18 [-15.15 to 61.51]	0.04	L	U	L	U	U	L
Heterogeneity: τ^2 = 7.18, I^2 = 5.63%, H^2 = 1.06			1.05 [-4.04 to 6.15]							
Test of $\theta_i = \theta_j$: Q(3) = 3.18, p = 0.36										
**Overall**			-0.03 [-0.82 to 0.76]							
Heterogeneity: τ^2 = 0.26, I^2 = 7.84%, H^2 = 1.09										
Test of $\theta_i = \theta_j$: Q(20) = 21.70, p = 0.36										
Test of group differences: $Q_b(5)$ = 4.45, p = 0.49										

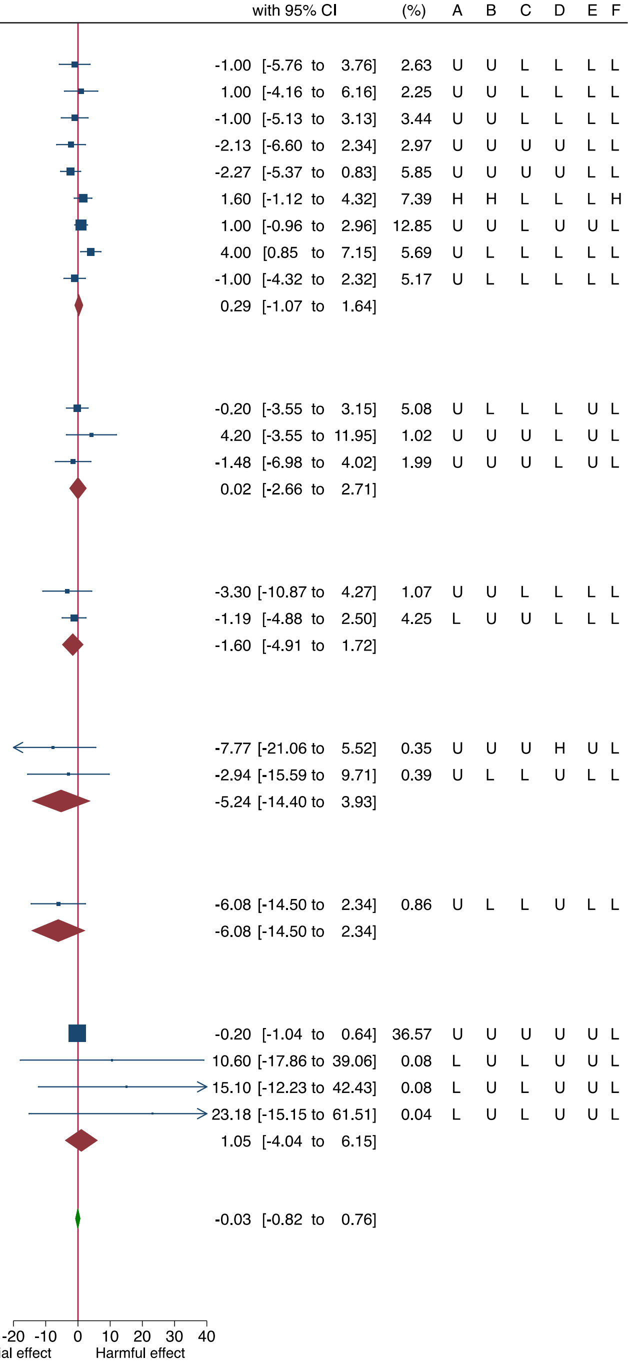

Test of θ = 0: z = -0.069, p = 0.945

Pooled effect estimates for each subgroup and overall effect are represented by the diamonds. Data are expressed as weighted mean differences with 95% confidence intervals using the generic inverse-variance method and random effects DerSimonian-Laird model. Paired analyses were applied to all crossover trials. Inter-study heterogeneity was assessed using the Cochrane Q statistic and quantified using the I^2 statistic, with significance set at p<0.100 and $I^2 \geq 50\%$ considered to be evidence of substantial heterogeneity.

Risk of Bias Legend: (H) High Risk; (L) Low Risk; (U) Unclear. The letters represent the following risk of bias domains: A, random sequence generation (selection bias); B, allocation concealment (selection bias); C, blinding of participants and personnel and outcome assessors (performance bias); D, incomplete outcome data (attrition bias); E, selective reporting (reporting bias); and F, other (carry-over effect) bias. High other risk of bias was given to crossover trials which had no washout between interventions. Trials which did not have this characteristic were rated as Low.
Pooled effect summary calculated with the χ^2 test. Test for group differences calculated with meta-regression, which uses the Wald test.
To convert U/L to ukat/L, multiply U/L by 0.0167.
AST=aspartate aminotransferase; CI=confidence interval; Ctrl=control; HD=high dose; HF=high fructose; hHFI=heterozygote high fructose intolerance; HS=high sucrose; LD=low dose; MD=medium dose; MF=medium fructose; SSB=sugar-sweetened beverage.

Supplementary Figure S21: Forest plot of controlled trials of the effect of important food sources of fructose-containing sugars and AST (U/L) in subtraction trials

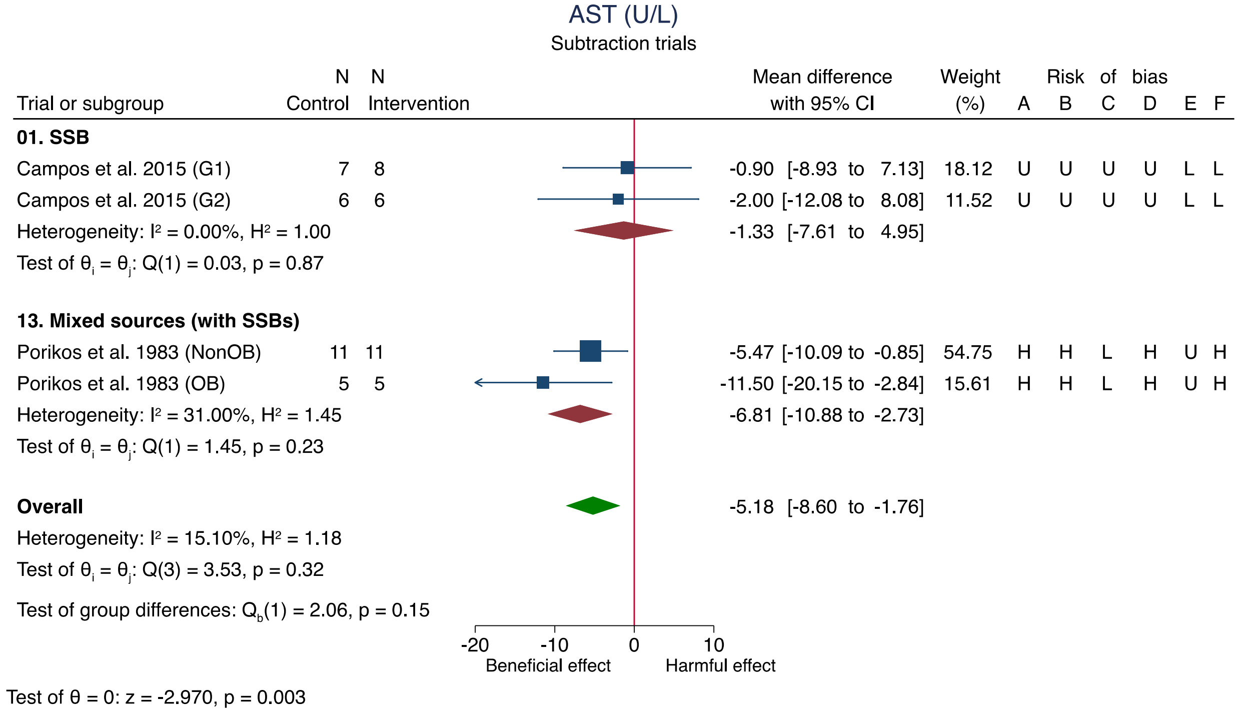

Pooled effect estimates for each subgroup and overall effect are represented by the diamonds. Data are expressed as weighted mean differences with 95% confidence intervals using the generic inverse-variance method and random effects DerSimonian-Laird model. Paired analyses were applied to all crossover trials. Inter-study heterogeneity was assessed using the Cochrane Q statistic and quantified using the I^2 statistic, with significance set at $p<0.100$ and $I^2 \geq 50\%$ considered to be evidence of substantial heterogeneity.

Risk of Bias Legend: (H) High Risk; (L) Low Risk; (U) Unclear. The letters represent the following risk of bias domains: A, random sequence generation (selection bias); B, allocation concealment (selection bias); C, blinding of participants and personnel and outcome assessors (performance bias); D, incomplete outcome data (attrition bias); E, selective reporting (reporting bias); and F, other (carry-over effect) bias. High other risk of bias was given to crossover trials which had no washout between interventions. Trials which did not have this characteristic were rated as Low.

Pooled effect summary calculated with the χ^2 test. Test for group differences calculated with meta-regression, which uses the Wald test.

To convert U/L to ukat/L, multiply U/L by 0.0167.

AST=aspartate aminotransferase; CI=confidence interval; G1=group 1; G2=group 2; NonOB=non-obese; OB=obese; SSB=sugar-sweetened beverage.

Supplementary Figure S22: Forest plot of controlled trials of the effect of important food sources of fructose-containing sugars and AST (U/L) in *ad libitum* trials

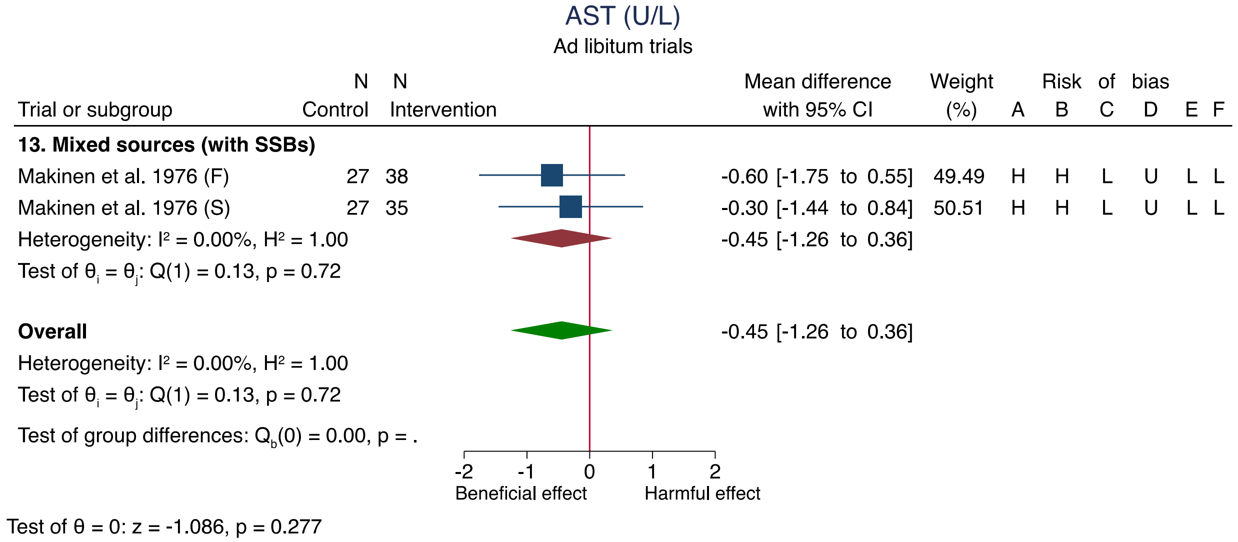

Pooled effect estimates for each subgroup and overall effect are represented by the diamonds. Data are expressed as weighted mean differences with 95% confidence intervals using the generic inverse-variance method and random effects DerSimonian-Laird model. Paired analyses were applied to all crossover trials. Inter-study heterogeneity was assessed using the Cochrane Q statistic and quantified using the I^2 statistic, with significance set at $p<0.100$ and $I^2 \geq 50\%$ considered to be evidence of substantial heterogeneity.

Risk of Bias Legend: (H) High Risk; (L) Low Risk; (U) Unclear. The letters represent the following risk of bias domains: A, random sequence generation (selection bias); B, allocation concealment (selection bias); C, blinding of participants and personnel and outcome assessors (performance bias); D, incomplete outcome data (attrition bias); E, selective reporting (reporting bias); and F, other (carry-over effect) bias. High other risk of bias was given to crossover trials which had no washout between interventions. Trials which did not have this characteristic were rated as Low.

Pooled effect summary calculated with the χ^2 test. Test for group differences calculated with meta-regression, which uses the Wald test.

To convert U/L to ukat/L, multiply U/L by 0.0167.

AST=aspartate aminotransferase; CI=confidence interval; F=fructose; S=sucrose; SSB=sugar-sweetened beverage.

Supplementary Figure S23: Sensitivity analysis of the systematic removal of each trial for the effect of important food sources of fructose-containing sugars and IHCL (SMD) in substitution trials

Influence analysis

IHCL (SMD) in substitution trials

Trial removed	Mean difference with 95% CI	P_{MD}	I^2 (%)	P_Q
**Overall**	0.36 [-0.07 to 0.79]	0.098	7	0.377
Agebratt et al. 2016	0.38 [-0.08 to 0.84]	0.111	13	0.309
Dikariyanto et al. 2020	0.31 [-0.13 to 0.75]	0.165	4	0.406
Jin et al. 2014	0.42 [-0.04 to 0.87]	0.066	8	0.360
Johnston et al. 2013 (T1)	0.38 [-0.09 to 0.85]	0.108	13	0.309
Johnston et al. 2013 (T2)	0.43 [-0.02 to 0.88]	0.063	8	0.369
Luukkonen et al. 2018 (SAT)	0.43 [-0.01 to 0.88]	0.058	6	0.389
Luukkonen et al. 2018 (UNSAT)	0.39 [-0.08 to 0.85]	0.105	13	0.309
Maersk et al. 2012 (M)	0.31 [-0.13 to 0.75]	0.164	5	0.391
Ngo Sock et al. 2010	0.41 [-0.05 to 0.87]	0.083	11	0.325
Parry et al. 2020	0.43 [0.00 to 0.86]	0.046	0	0.487
Schwarz et al. 2015	0.32 [-0.12 to 0.76]	0.158	8	0.360
Schwimmer et al. 2019	0.31 [-0.11 to 0.73]	0.144	0	0.510
Silbernagel et al. 2011	0.41 [-0.05 to 0.87]	0.082	11	0.327
Simons et al. 2020	0.31 [-0.13 to 0.75]	0.163	6	0.384
Umpleby et al. 2017 (H)	0.30 [-0.12 to 0.73]	0.164	3	0.418
Umpleby et al. 2017 (NAFLD)	0.30 [-0.12 to 0.73]	0.165	4	0.411

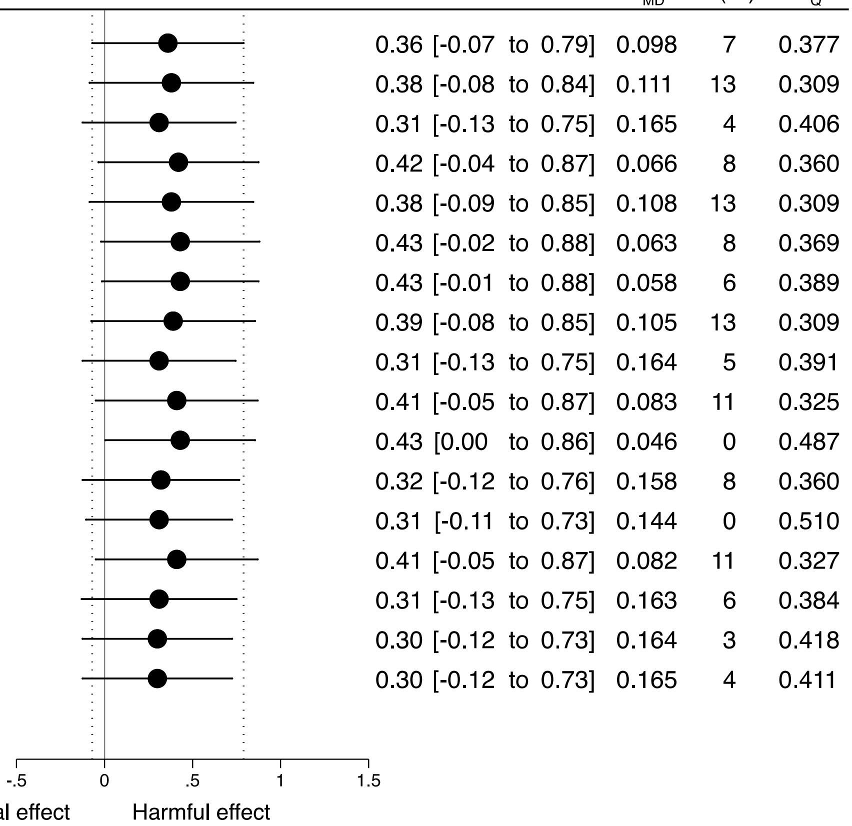

Influence analysis: Removal of each trial, one at a time and recalculation of the overall effect and heterogeneity

CI=confidence interval; IHCL=intrahepatocellular lipid; H=healthy; M=milk; MD=mean difference; NAFLD=non-alcoholic fatty liver disease; SAT=saturated fat; SMD=standardized mean difference; T1=test group 1; T2=test group 2; UNSAT=unsaturated fat.

Supplementary Figure S24: Sensitivity analysis of the systematic removal of each trial for the effect of important food sources of fructose-containing sugars and IHCL (SMD) in addition trials

Influence analysis

IHCL (SMD) in addition trials

Trial removed	Mean difference with 95% CI	P_{MD}	I^2 (%)	P_Q
**Overall**	1.72 [1.08 to 2.36]	< 0.001	0	0.943
Johnston et al. 2013	1.69 [1.03 to 2.36]	< 0.001	0	0.915
Koopman et al. 2014 (HS-F)	1.76 [1.08 to 2.44]	< 0.001	0	0.916
Koopman et al. 2014 (HS-S)	1.93 [1.23 to 2.63]	< 0.001	0	0.984
Lê et al. 2006	1.78 [1.10 to 2.46]	< 0.001	0	0.924
Lê et al. 2009 (H)	1.68 [1.02 to 2.33]	< 0.001	0	0.928
Lê et al. 2009 (ODM2)	1.66 [1.01 to 2.30]	< 0.001	0	0.964
Maersk et al. 2012 (NSB)	1.74 [1.06 to 2.42]	< 0.001	0	0.911
Maersk et al. 2012 (Water)	1.67 [1.01 to 2.32]	< 0.001	0	0.932
Ngo Sock et al. 2010	1.73 [1.05 to 2.41]	< 0.001	0	0.911
Sigala et al. 2021 (HFCS)	1.68 [1.02 to 2.33]	< 0.001	0	0.928
Sigala et al. 2021 (Sucrose)	1.66 [1.01 to 2.30]	< 0.001	0	0.958
Silbernagel et al. 2011	1.72 [1.05 to 2.39]	< 0.001	0	0.910
Sobrecases et al. 2010	1.69 [1.03 to 2.36]	< 0.001	0	0.915

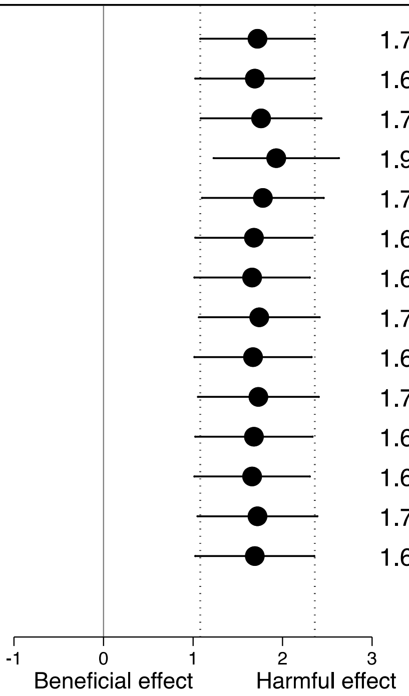

Influence analysis: Removal of each trial, one at a time and recalculation of the overall effect and heterogeneity

CI=confidence interval; H=healthy; HS-F=high sucrose-frequency; HS-S=high sucrose-size; IHCL=intrahepatocellular lipid; MD=mean difference; NSB=non-nutritive sweetened beverage; ODM2=offspring of type-2 diabetes patients; SMD=standardized mean difference.

Supplementary Figure S25: Sensitivity analysis of the systematic removal of each trial in the primary analysis of the effect of important food sources of fructose-containing sugars and IHCL (SMD) in subtraction trials

Influence analysis

IHCL (SMD) in subtraction trials

Trial removed	Mean difference with 95% CI	P_{MD}	I^2 (%)	P_Q
**Overall**	-0.52 [-1.60 to 0.56]	0.345	0	0.470
Campos et al. 2015 (G1)	-1.00 [-2.70 to 0.70]	0.247	.	.
Campos et al. 2015 (G2)	-0.19 [-1.59 to 1.21]	0.788	.	.

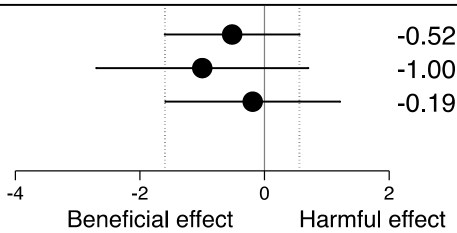

Influence analysis: Removal of each trial, one at a time and recalculation of the overall effect and heterogeneity

CI=confidence interval; G1=group 1; G2=group 2; IHCL=intrahepatocellular lipid; MD=mean difference; SMD=standardized mean difference.

Supplementary Figure S26: Sensitivity analysis of the systematic removal of each trial for the effect of important food sources of fructose-containing sugars and ALT (U/L) in substitution trials

Influence analysis

ALT (U/L) in substitution trials

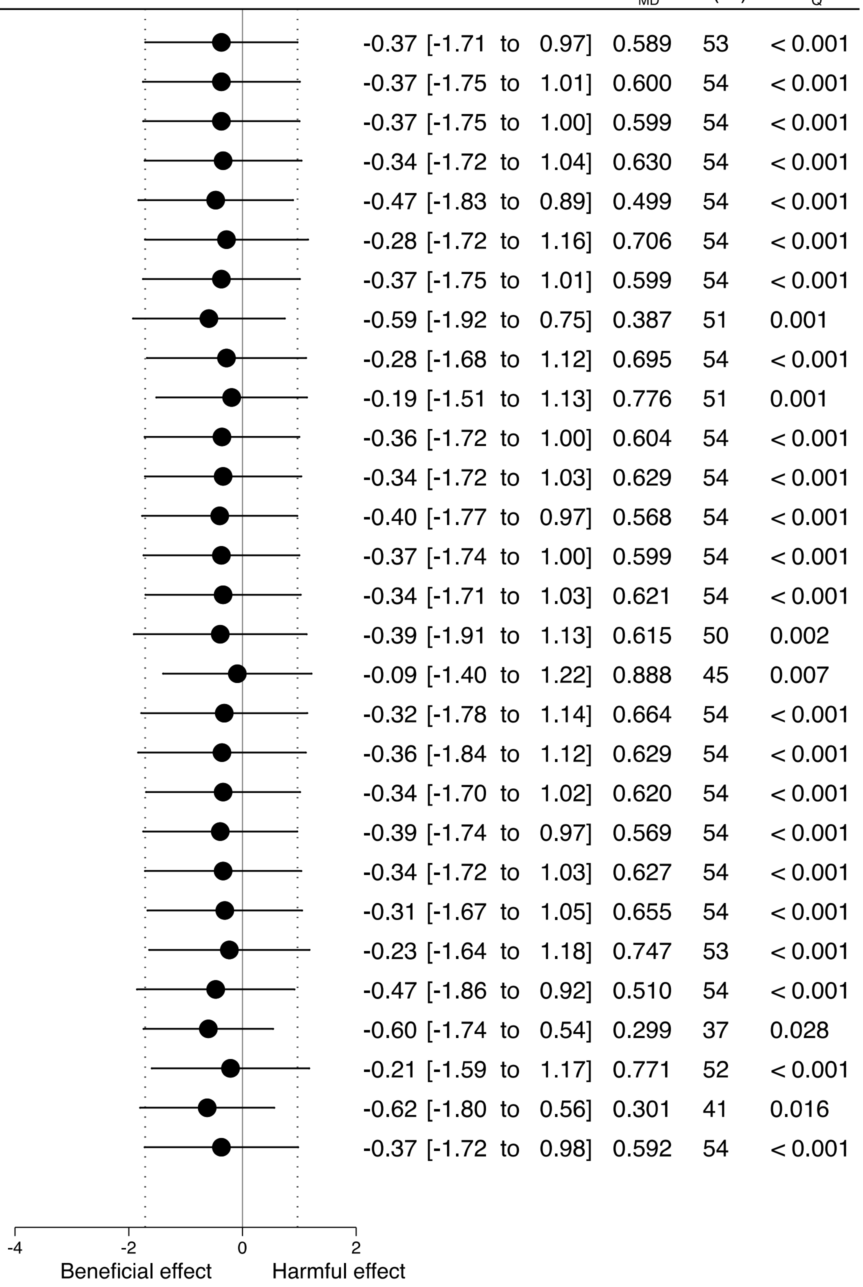

Trial removed	Mean difference with 95% CI	P_{MD}	I^2 (%)	P_Q
**Overall**	-0.37 [-1.71 to 0.97]	0.589	53	< 0.001
Aeberli et al. 2011 (HF)	-0.37 [-1.75 to 1.01]	0.600	54	< 0.001
Aeberli et al. 2011 (HS)	-0.37 [-1.75 to 1.00]	0.599	54	< 0.001
Aeberli et al. 2011 (MF)	-0.34 [-1.72 to 1.04]	0.630	54	< 0.001
Alemán et al. 2021	-0.47 [-1.83 to 0.89]	0.499	54	< 0.001
Chiu et al. 2020	-0.28 [-1.72 to 1.16]	0.706	54	< 0.001
Claesson et al. 2009	-0.37 [-1.75 to 1.01]	0.599	54	< 0.001
Cox et al. 2012	-0.59 [-1.92 to 0.75]	0.387	51	0.001
Dikariyanto et al. 2020	-0.28 [-1.68 to 1.12]	0.695	54	< 0.001
Eslami et al. 2019	-0.19 [-1.51 to 1.13]	0.776	51	0.001
Jin et al. 2014	-0.36 [-1.72 to 1.00]	0.604	54	< 0.001
Johnston et al. 2013 (T1)	-0.34 [-1.72 to 1.03]	0.629	54	< 0.001
Johnston et al. 2013 (T2)	-0.40 [-1.77 to 0.97]	0.568	54	< 0.001
Kaliora et al. 2016	-0.37 [-1.74 to 1.00]	0.599	54	< 0.001
Kanellos et al. 2017	-0.34 [-1.71 to 1.03]	0.621	54	< 0.001
Kelsay et al. 1974	-0.39 [-1.91 to 1.13]	0.615	50	0.002
Lehtonen et al. 2010	-0.09 [-1.40 to 1.22]	0.888	45	0.007
Lehtonen et al. 2011 (BB)	-0.32 [-1.78 to 1.14]	0.664	54	< 0.001
Lehtonen et al. 2011 (SB)	-0.36 [-1.84 to 1.12]	0.629	54	< 0.001
Luukkonen et al. 2018 (SAT)	-0.34 [-1.70 to 1.02]	0.620	54	< 0.001
Luukkonen et al. 2018 (UNSAT)	-0.39 [-1.74 to 0.97]	0.569	54	< 0.001
Ngo Sock et al. 2010	-0.34 [-1.72 to 1.03]	0.627	54	< 0.001
Nier et al. 2018	-0.31 [-1.67 to 1.05]	0.655	54	< 0.001
Parry et al. 2020	-0.23 [-1.64 to 1.18]	0.747	53	< 0.001
Ponce et al. 2019	-0.47 [-1.86 to 0.92]	0.510	54	< 0.001
Purkins et al. 2004	-0.60 [-1.74 to 0.54]	0.299	37	0.028
Ribeiro et al. 2017	-0.21 [-1.59 to 1.17]	0.771	52	< 0.001
Schwimmer et al. 2019	-0.62 [-1.80 to 0.56]	0.301	41	0.016
Vos et al. 2009	-0.37 [-1.72 to 0.98]	0.592	54	< 0.001

Influence analysis: Removal of each trial, one at a time and recalculation of the overall effect and heterogeneity

ALT=alanine aminotransferase; BB=bilberries; CI=confidence interval; HF=high fructose; HS=high sucrose; MD=mean difference; MF=medium fructose; SAT=saturated fat; SB=seabuckthorn berries; T1=test group 1; T2=test group 2; UNSAT=unsaturated fat.

Supplementary Figure S27: Sensitivity analysis of the systematic removal of each trial for the effect of important food sources of fructose-containing sugars and ALT (U/L) in addition trials

Influence analysis

ALT (U/L) in addition trials

Trial removed	Mean difference with 95% CI	P_{MD}	I^2 (%)	P_Q
**Overall**	0.91 [-0.39 to 2.21]	0.169	31	0.050
Aeberli et al. 2011 (HF)	0.88 [-0.47 to 2.23]	0.203	33	0.041
Aeberli et al. 2011 (HS)	0.89 [-0.45 to 2.23]	0.192	34	0.040
Aeberli et al. 2011 (MF)	0.88 [-0.47 to 2.23]	0.198	33	0.040
Ahmed et al. 2010	0.94 [-0.38 to 2.26]	0.162	33	0.040
Alavinejad et al. 2015	0.93 [-0.39 to 2.25]	0.166	34	0.039
Amagase et al. 2009	0.98 [-0.36 to 2.32]	0.156	33	0.040
Bahrami et al. 2009	0.99 [-0.40 to 2.38]	0.164	33	0.040
Banini et al. 2006	0.92 [-0.41 to 2.25]	0.174	34	0.039
Debray et al. 2021 (Ctrl)	1.03 [-0.27 to 2.33]	0.120	31	0.058
Debray et al. 2021 (hHFI)	1.07 [-0.25 to 2.39]	0.112	31	0.058
Irannejad et al. 2020	0.92 [-0.40 to 2.24]	0.171	34	0.039
Johnston et al. 2013	0.54 [-0.63 to 1.71]	0.368	17	0.203
Kojadinovic et al. 2021	0.99 [-0.30 to 2.28]	0.134	31	0.055
Lê et al. 2009 (H)	0.72 [-0.54 to 1.99]	0.267	27	0.085
Lê et al. 2009 (ODM2)	0.56 [-0.55 to 1.67]	0.321	13	0.265
Ngo Sock et al. 2010	0.78 [-0.55 to 2.11]	0.250	31	0.054
Ravn-Haren et al. 2013 (Cld vs Ctrl)	1.08 [-0.27 to 2.43]	0.117	31	0.056
Ravn-Haren et al. 2013 (Cld vs Pom)	1.01 [-0.38 to 2.40]	0.151	33	0.043
Ravn-Haren et al. 2013 (Clr vs Ctrl)	1.05 [-0.32 to 2.41]	0.130	32	0.049
Ravn-Haren et al. 2013 (Clr vs Pom)	0.98 [-0.40 to 2.36]	0.163	33	0.040
Ravn-Haren et al. 2013 (Ctrl)	0.96 [-0.40 to 2.32]	0.170	34	0.039
Ravn-Haren et al. 2013 (Pom)	0.90 [-0.47 to 2.26]	0.198	34	0.040
Schell et al. 2019	0.98 [-0.36 to 2.32]	0.151	33	0.041
Sobrecases et al. 2010	0.86 [-0.46 to 2.18]	0.202	33	0.044
Tang et al. 2020 (HD)	0.90 [-0.41 to 2.21]	0.179	33	0.042
Tang et al. 2020 (LD)	0.89 [-0.42 to 2.20]	0.181	33	0.045
Tang et al. 2020 (MedD)	0.89 [-0.41 to 2.19]	0.176	32	0.051
Tutino et al. 2021	0.94 [-0.40 to 2.28]	0.169	34	0.039
Zafrilla et al. 2021 (Stevia)	0.70 [-0.62 to 2.02]	0.297	29	0.075
Zafrilla et al. 2021 (Sucralose)	1.04 [-0.33 to 2.41]	0.136	32	0.046
Zakaria et al. 2018	1.07 [-0.21 to 2.35]	0.102	29	0.074

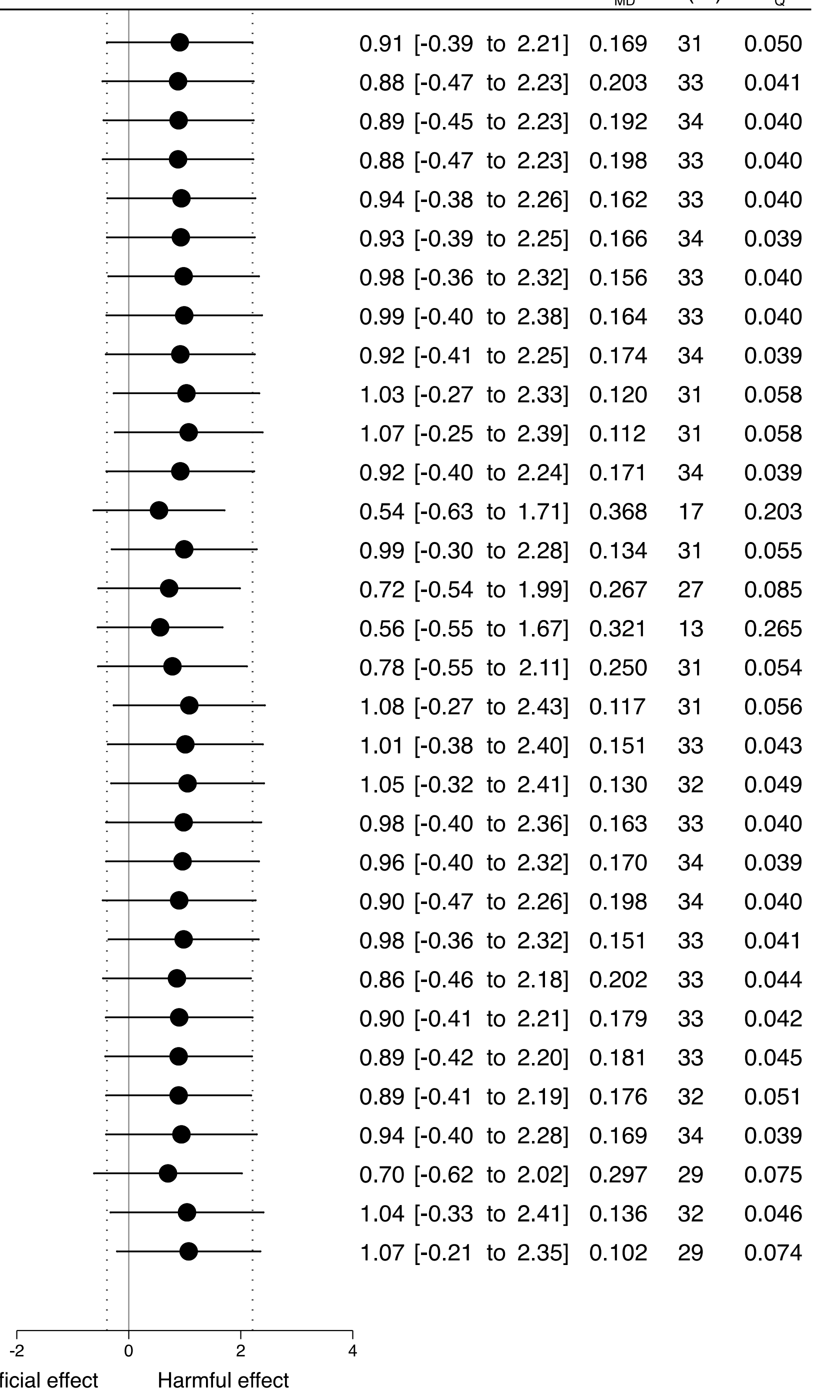

Influence analysis: Removal of each trial, one at a time and recalculation of the overall effect and heterogeneity

ALT=alanine aminotransferase; CI=confidence interval; Cld=cloudy apple juice; Clr=clear apple juice; Ctrl=control group; H=healthy; HD=high dose; HF=high fructose; hHFI=heterozygote high fructose intolerance; HS=high

sucrose; LD=low dose; MD=mean difference; MedD=medium dose; MF=medium fructose; ODM2=offspring of type-2 diabetes patients; Pom=apple pomace; SSB=sugar-sweetened beverage.

Supplementary Figure S28: Sensitivity analysis of the systematic removal of each trial for the effect of important food sources of fructose-containing sugars and ALT (U/L) in subtraction trials

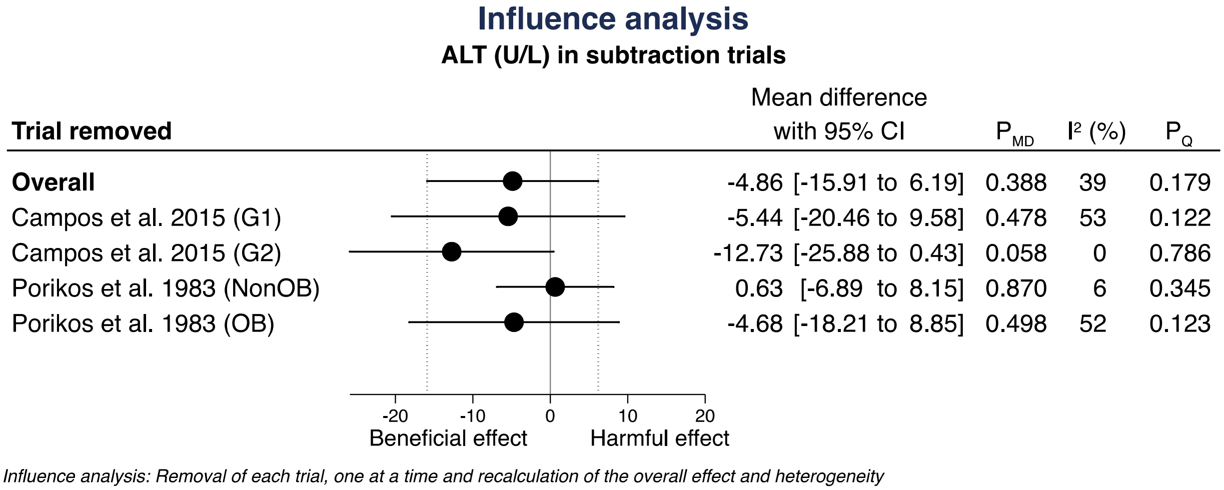

ALT=alanine aminotransferase; CI=confidence interval; G1=group 1; G2=group 2; MD=mean difference; NonOB=non-obese; OB=obese.

Supplementary Figure S29: Sensitivity analysis of the systematic removal of each trial for the effect of important food sources of fructose-containing sugars and ALT (U/L) in *ad libitum* trials

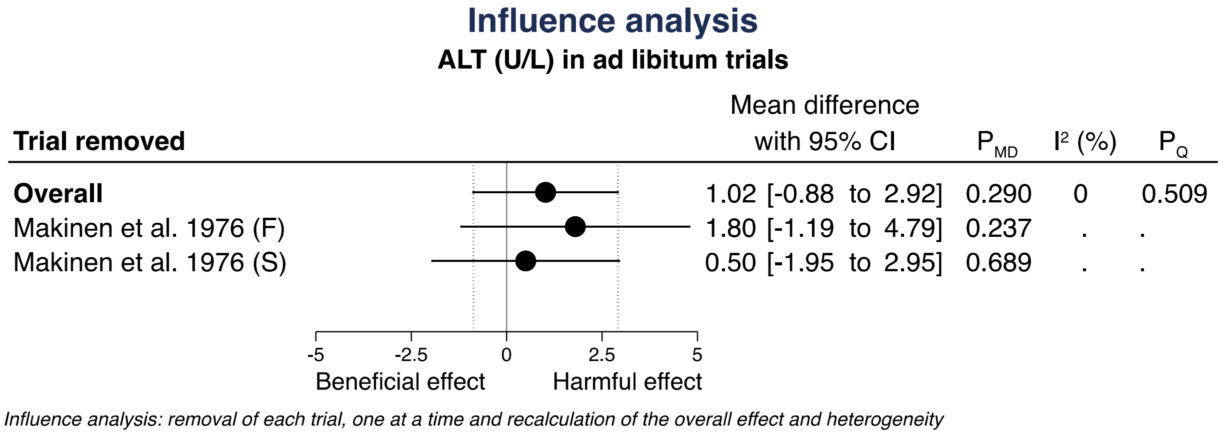

ALT=alanine aminotransferase; CI=confidence interval; F=fructose; MD=mean difference; S=sucrose.

Supplementary Figure S30: Sensitivity analysis of the systematic removal of each trial for the effect of important food sources of fructose-containing sugars and AST (U/L) in substitution trials

Influence analysis

AST (U/L) in substitution trials

Trial removed	Mean difference with 95% CI	P_{MD}	I^2 (%)	P_Q
**Overall**	0.39 [-0.87 to 1.65]	0.546	47	0.008
Aeberli et al. 2011 (HF)	0.48 [-0.84 to 1.80]	0.475	49	0.005
Aeberli et al. 2011 (HS)	0.38 [-0.93 to 1.69]	0.568	49	0.006
Aeberli et al. 2011 (MF)	0.49 [-0.84 to 1.82]	0.467	49	0.005
Alemán et al. 2021	0.37 [-0.92 to 1.66]	0.570	49	0.006
Chiu et al. 2020	0.63 [-0.75 to 2.01]	0.370	45	0.011
Claesson et al. 2009	0.34 [-0.98 to 1.66]	0.612	48	0.007
Cox et al. 2012	0.25 [-1.03 to 1.53]	0.703	46	0.010
Eslami et al. 2019	0.31 [-1.00 to 1.62]	0.645	48	0.007
Jin et al. 2014	0.35 [-0.95 to 1.65]	0.598	48	0.006
Johnston et al. 2013 (T1)	0.56 [-0.75 to 1.87]	0.405	48	0.007
Johnston et al. 2013 (T2)	0.46 [-0.85 to 1.78]	0.489	49	0.005
Kaliora et al. 2016	0.42 [-0.92 to 1.76]	0.539	49	0.005
Kanellos et al. 2017	0.40 [-0.90 to 1.70]	0.545	49	0.005
Kelsay et al. 1974	0.60 [-0.83 to 2.03]	0.411	48	0.006
Luukkonen et al. 2018 (SAT)	0.48 [-0.82 to 1.78]	0.468	49	0.006
Luukkonen et al. 2018 (UNSAT)	0.38 [-0.92 to 1.68]	0.564	49	0.006
Ngo Sock et al. 2010	0.45 [-0.89 to 1.79]	0.512	49	0.005
Nier et al. 2018	0.49 [-0.75 to 1.74]	0.443	45	0.011
Ponce et al. 2019	0.38 [-0.95 to 1.71]	0.578	49	0.006
Purkins et al. 2004	-0.28 [-1.12 to 0.56]	0.523	6	0.381
Ribeiro et al. 2017	0.52 [-0.83 to 1.86]	0.447	49	0.006
Schwimmer et al. 2019	0.05 [-1.07 to 1.18]	0.935	34	0.059
Vos et al. 2009	0.40 [-0.88 to 1.68]	0.541	49	0.005

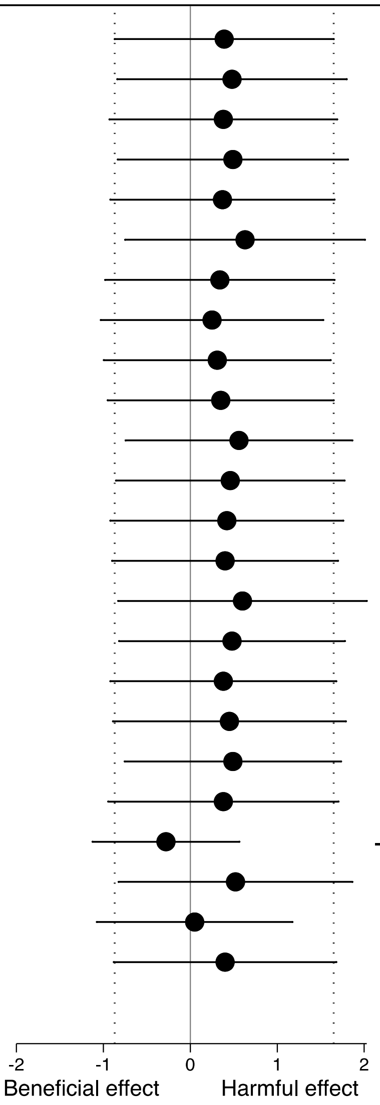

Influence analysis: Removal of each trial, one at a time and recalculation of the overall effect and heterogeneity

AST=aspartate aminotransferase; CI=confidence interval; HF=high fructose; HS=high sucrose; MD=mean difference; MF=medium fructose; SAT=saturated fat; T1=test group 1; T2=test group 2; UNSAT=unsaturated fat.

Supplementary Figure S31: Sensitivity analysis of the systematic removal of each trial for the effect of important food sources of fructose-containing sugars and AST (U/L) in addition trials

Influence analysis

AST (U/L) in addition trials

Trial removed	Mean difference with 95% CI	P_{MD}	I^2 (%)	P_Q
**Overall**	-0.03 [-0.82 to 0.76]	0.945	8	0.357
Aeberli et al. 2011 (HF)	0.00 [-0.85 to 0.85]	0.998	12	0.307
Aeberli et al. 2011 (HS)	-0.06 [-0.91 to 0.79]	0.897	12	0.308
Aeberli et al. 2011 (MF)	0.01 [-0.85 to 0.87]	0.985	12	0.310
Ahmed et al. 2010	0.00 [-0.77 to 0.77]	0.994	7	0.371
Alavinejad et al. 2015	0.01 [-0.71 to 0.73]	0.982	4	0.411
Amagase et al. 2009	-0.02 [-0.90 to 0.86]	0.962	12	0.300
Bahrami et al. 2009	0.02 [-1.04 to 1.08]	0.976	11	0.313
Banini et al. 2006	-0.07 [-0.85 to 0.71]	0.860	7	0.363
Debray et al. 2021 (Ctrl)	0.04 [-0.77 to 0.85]	0.926	9	0.345
Debray et al. 2021 (hHFI)	0.08 [-0.65 to 0.81]	0.823	3	0.415
Irannejad et al. 2020	-0.02 [-0.86 to 0.82]	0.969	12	0.310
Johnston et al. 2013	-0.15 [-0.94 to 0.64]	0.704	6	0.383
Kojadinovic et al. 2021	0.00 [-0.84 to 0.84]	0.994	11	0.313
Ngo Sock et al. 2010	-0.18 [-1.03 to 0.67]	0.679	7	0.367
Schell et al. 2019	0.01 [-0.80 to 0.82]	0.983	9	0.337
Tang et al. 2020 (HD)	-0.04 [-0.85 to 0.77]	0.929	10	0.328
Tang et al. 2020 (LD)	-0.04 [-0.82 to 0.74]	0.919	7	0.364
Tang et al. 2020 (Med D)	-0.04 [-0.80 to 0.72]	0.921	6	0.377
Tutino et al. 2021	0.03 [-0.82 to 0.88]	0.951	11	0.319
Zafrilla et al. 2021 (Stevia)	-0.22 [-0.86 to 0.42]	0.495	0	0.718
Zafrilla et al. 2021 (Sucralose)	0.03 [-0.83 to 0.89]	0.949	11	0.316

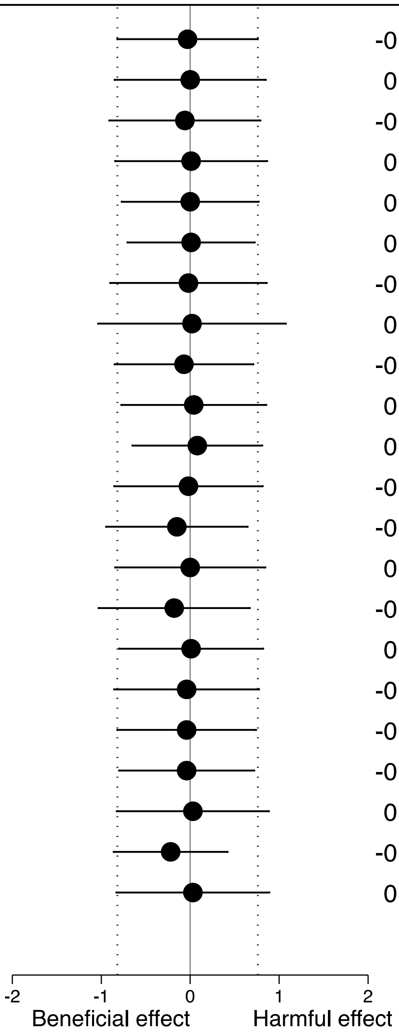

Influence analysis: Removal of each trial, one at a time and recalculation of the overall effect and heterogeneity

AST=aspartate aminotransferase; CI=confidence interval; Ctrl=control; HD=high dose; HF=high fructose; hHFI=heterozygote high fructose intolerance; HS=high sucrose; LD=low dose; MD=mean difference; Med D=medium dose; MF=medium fructose.

Supplementary Figure S32: Sensitivity analysis of the systematic removal of each trial in the primary analysis of the effect of important food sources of fructose-containing sugars and AST (U/L) in subtraction trials

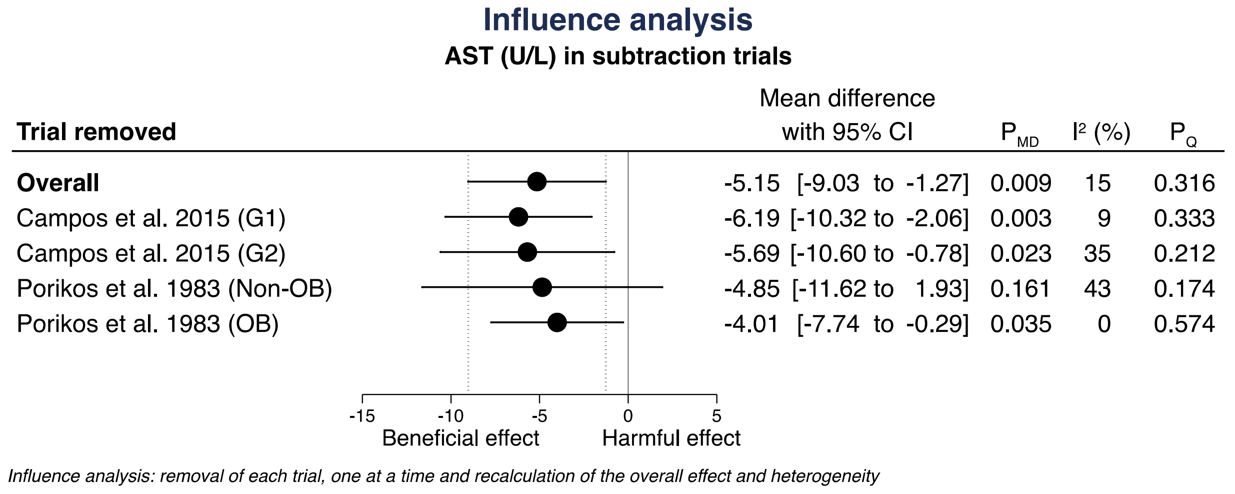

AST=aspartate aminotransferase; CI=confidence interval; G1=group 1; G2=group 2; MD=mean difference; NonOB=non-obese; OB=obese.

Supplementary Figure S33: Sensitivity analysis of the systematic removal of each trial in the primary analysis of the effect of important food sources of fructose-containing sugars and AST (U/L) in *ad libitum* trials

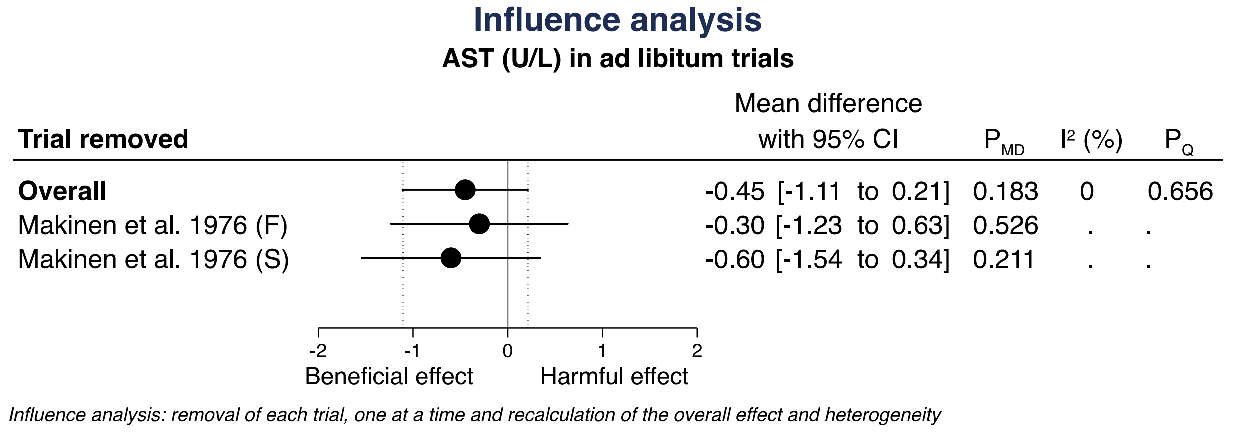

AST=aspartate aminotransferase; CI, confidence interval; F=fructose; MD=mean difference; S=sucrose; SSB=sugar-sweetened beverage.

Supplementary Figure S34: Sensitivity analysis of the systematic removal of each trial for the effect of SSBs on ALT (U/L) in addition trials

Influence analysis

ALT (U/L) in addition SSB trials

Trial removed	Mean difference with 95% CI	P_{MD}	I^2 (%)	P_Q
**Overall**	3.09 [0.50 to 5.68]	0.020	58	0.006
Aeberli et al. 2011 (HF)	3.22 [0.36 to 6.08]	0.027	62	0.003
Aeberli et al. 2011 (HS)	3.19 [0.40 to 5.99]	0.025	62	0.003
Aeberli et al. 2011 (MF)	3.20 [0.37 to 6.03]	0.026	62	0.003
Debray et al. 2021 (Ctrl)	3.58 [1.00 to 6.15]	0.006	56	0.012
Debray et al. 2021 (hHFI)	3.69 [1.10 to 6.28]	0.005	53	0.020
Johnston et al. 2013	2.48 [-0.09 to 5.05]	0.058	53	0.020
Lê et al. 2009 (H)	2.72 [0.04 to 5.40]	0.046	59	0.007
Lê et al. 2009 (ODM2)	2.36 [0.00 to 4.71]	0.050	47	0.042
Ngo Sock et al. 2010	3.01 [0.14 to 5.88]	0.039	62	0.004
Sobrecases et al. 2010	3.02 [0.29 to 5.75]	0.030	62	0.004
Zafrilla et al. 2021 (Stevia)	3.00 [0.04 to 5.96]	0.047	61	0.004
Zafrilla et al. 2021 (Sucralose)	3.60 [0.87 to 6.32]	0.009	55	0.015

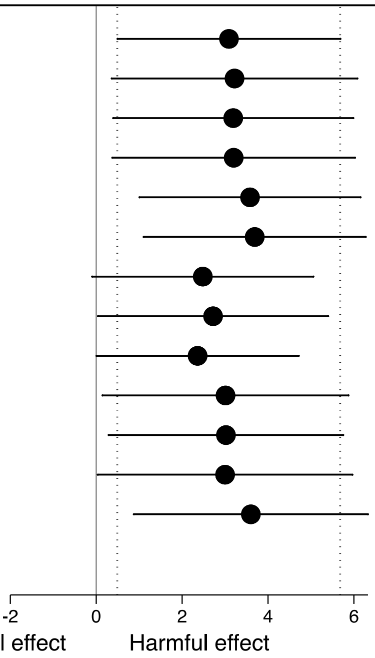

Influence analysis: Removal of each trial, one at a time and recalculation of the overall effect and heterogeneity

ALT=alanine aminotransferase; CI=confidence interval; Ctrl=control group; H=healthy; hHFI=heterozygote high fructose intolerance; HS=high sucrose; MD=mean difference; MF=medium fructose; ODM2=offspring of type-2 diabetes patients; SSB=sugar-sweetened beverage.

Supplementary Figure S35: Sensitivity analysis of the systematic removal of each trial for the effect of 100% fruit juice on ALT (U/L) in addition trials

Influence analysis

ALT (U/L) in addition 100% fruit juice trials

Trial removed	Mean difference with 95% CI	P_{MD}	I^2 (%)	P_Q
**Overall**	-0.80 [-2.44 to 0.84]	0.340	0	0.949
Amagase et al. 2009	-0.81 [-2.51 to 0.89]	0.351	0	0.895
Banini et al. 2006	-0.83 [-2.48 to 0.83]	0.327	0	0.902
Kojadinovic et al. 2021	-0.68 [-2.33 to 0.97]	0.420	0	0.990
Ravn-Haren et al. 2013 (Cld vs Ctrl)	-0.59 [-2.44 to 1.27]	0.532	0	0.920
Ravn-Haren et al. 2013 (Cld vs Pom)	-0.94 [-2.82 to 0.94]	0.325	0	0.906
Ravn-Haren et al. 2013 (Clr vs Ctrl)	-0.72 [-2.56 to 1.11]	0.445	0	0.899
Ravn-Haren et al. 2013 (Clr vs Pom)	-1.04 [-2.90 to 0.82]	0.270	0	0.930

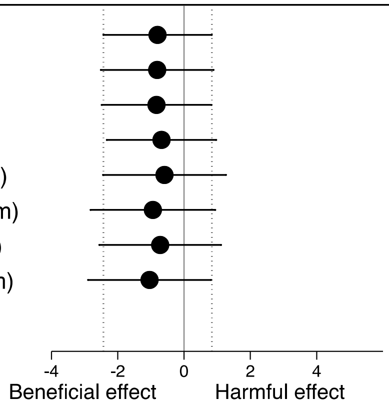

Influence analysis: Removal of each trial, one at a time and recalculation of the overall effect and heterogeneity

ALT=alanine aminotransferase; CI=confidence interval; Cld=cloudy apple juice; Clr=clear apple juice; Ctrl=control group; MD=mean difference; Pom=apple pomace.

Supplementary Figure S36: Sensitivity analysis of the systematic removal of each trial for the effect of fruit on ALT (U/L) in addition trials

Influence analysis

ALT (U/L) in addition fruit trials

Trial removed	Mean difference with 95% CI	P_{MD}	I^2 (%)	P_Q
**Overall**	0.44 [-2.22 to 3.10]	0.745	0	0.935
Ravn-Haren et al. 2013 (Ctrl)	0.53 [-2.73 to 3.79]	0.752	0	0.812
Ravn-Haren et al. 2013 (Pom)	-0.09 [-3.37 to 3.19]	0.959	0	0.935
Schell et al. 2019	0.74 [-2.15 to 3.63]	0.617	0	0.924
Tutino et al. 2021	0.48 [-2.44 to 3.40]	0.744	0	0.811

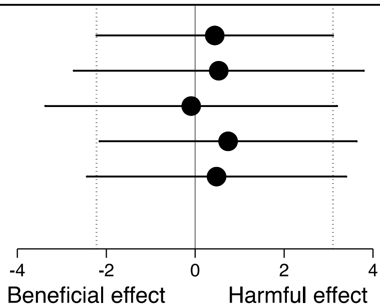

Influence analysis: Removal of each trial, one at a time and recalculation of the overall effect and heterogeneity

ALT=alanine aminotransferase; CI=confidence interval; Ctrl=control group; MD=mean difference; Pom=apple pomace.

Supplementary Figure S37: Sensitivity analysis of the systematic removal of each trial for the effect of dried fruit on ALT (U/L) in addition trials

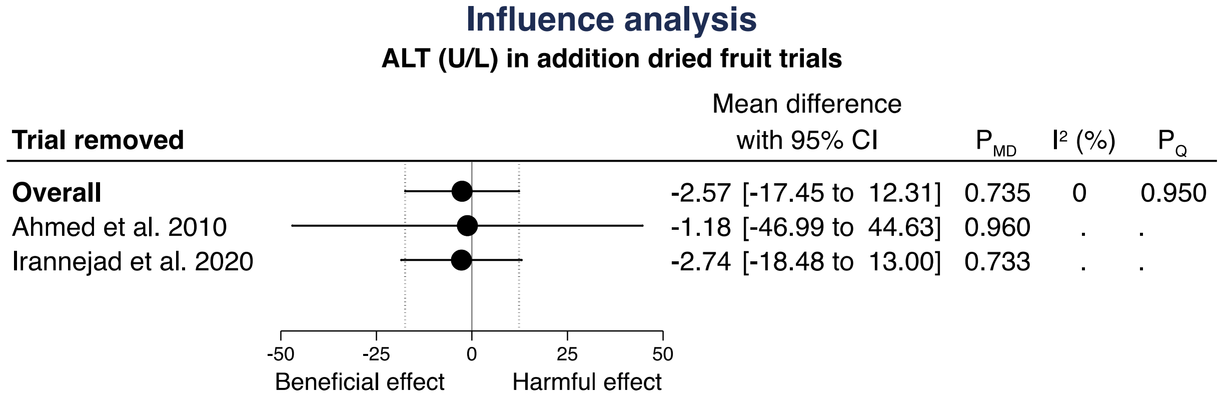

Influence analysis: Removal of each trial, one at a time and recalculation of the overall effect and heterogeneity

ALT=alanine aminotransferase; CI=confidence interval; MD=mean difference.

Supplementary Figure S38: Sensitivity analysis of the systematic removal of each trial for the effect of honey on ALT (U/L) in addition trials

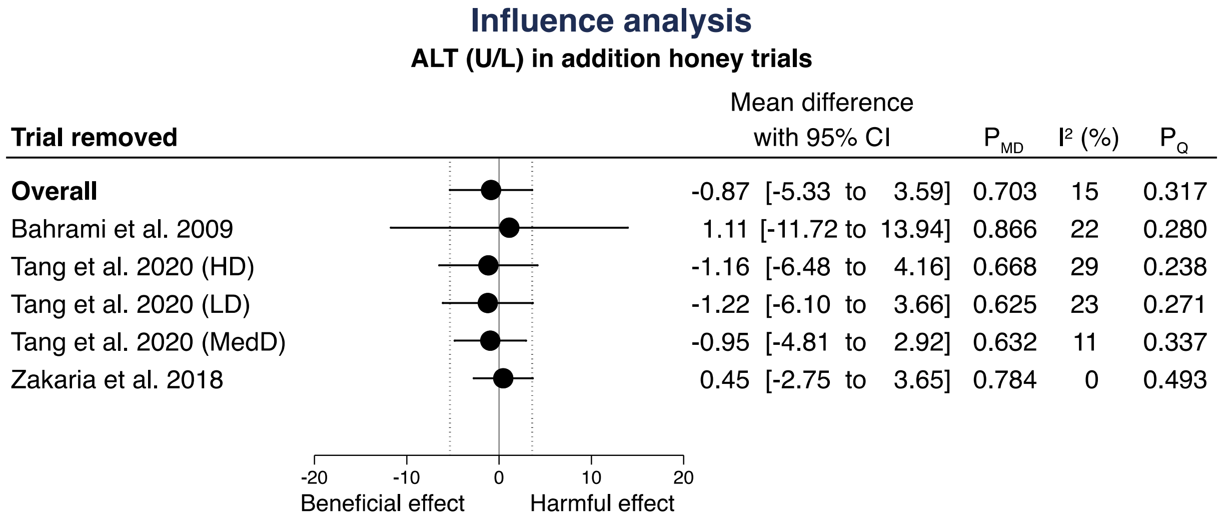

Influence analysis: Removal of each trial, one at a time and recalculation of the overall effect and heterogeneity

ALT=alanine aminotransferase; CI=confidence interval; HD=high dose; MD=mean difference; MedD=medium dose.

Supplementary Figure S39: Sensitivity analysis of the systematic removal of each trial for the effect of SSBs on ALT (U/L) in subtraction trials

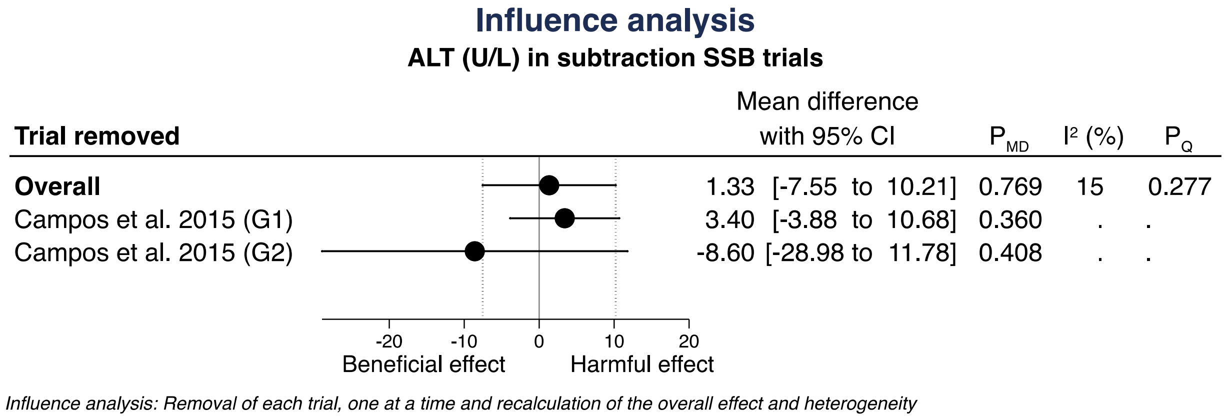

ALT=alanine aminotransferase; CI=confidence interval; G1=group 1; G2=group 2; MD=mean difference; SSB=sugar-sweetened beverage.

Supplementary Figure S40: Sensitivity analysis of the systematic removal of each trial for the effect of mixed sources (with SSBs) on ALT (U/L) in subtraction trials

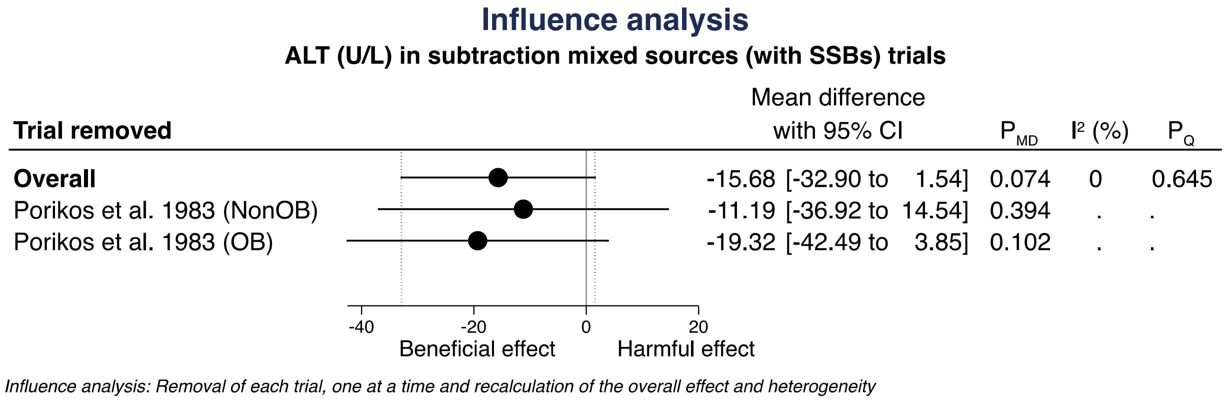

ALT=alanine aminotransferase; CI=confidence interval; G1=group 1; G2=group 2; MD=mean difference; NonOB=non-obese; OB=obese; SSB=sugar-sweetened beverage.

Supplementary Figure S41: Sensitivity analysis of the systematic removal of each trial for the effect of SSBs on AST (U/L) in addition trials

Influence analysis

AST (U/L) in addition SSB trials

Trial removed	Mean difference with 95% CI	P_{MD}	I^2 (%)	P_Q
**Overall**	0.29 [-1.07 to 1.65]	0.677	32	0.165
Aeberli et al. 2011 (HF)	0.36 [-1.11 to 1.83]	0.629	38	0.125
Aeberli et al. 2011 (HS)	0.21 [-1.27 to 1.69]	0.777	40	0.113
Aeberli et al. 2011 (MF)	0.39 [-1.09 to 1.87]	0.608	38	0.130
Debray et al. 2021 (Ctrl)	0.48 [-0.93 to 1.88]	0.501	32	0.169
Debray et al. 2021 (hHFI)	0.72 [-0.57 to 2.01]	0.275	17	0.299
Johnston et al. 2013	0.04 [-1.50 to 1.58]	0.962	36	0.145
Ngo Sock et al. 2010	0.07 [-1.58 to 1.72]	0.934	38	0.128
Zafrilla et al. 2021 (Stevia)	-0.03 [-1.16 to 1.10]	0.961	0	0.524
Zafrilla et al. 2021 (Sucralose)	0.44 [-1.05 to 1.93]	0.567	36	0.143

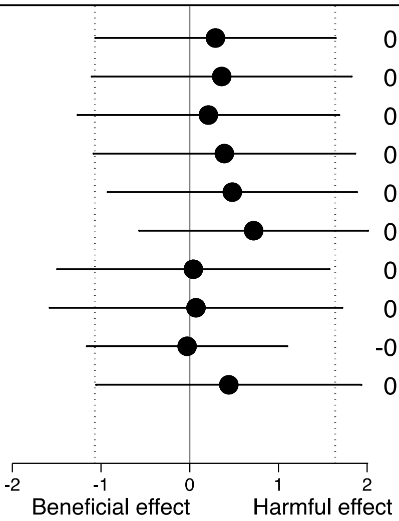

Influence analysis: Removal of each trial, one at a time and recalculation of the overall effect and heterogeneity

AST=aspartate aminotransferase; CI=confidence interval; Ctrl=control; HD=high dose; HF=high fructose; hHFI=heterozygote high fructose intolerance; HS=high sucrose; MD=mean difference; SSB=sugar-sweetened beverage.

Supplementary Figure S42: Sensitivity analysis of the systematic removal of each trial for the effect of 100% fruit juice on AST (U/L) in addition trials

Influence analysis

AST (U/L) in addition 100% fruit juice trials

Trial removed	Mean difference with 95% CI	P_{MD}	I^2 (%)	P_Q
**Overall**	0.02 [-2.67 to 2.71]	0.986	0	0.491
Amagase et al. 2009	0.68 [-4.72 to 6.08]	0.806	27	0.241
Banini et al. 2006	-0.55 [-3.42 to 2.31]	0.708	0	0.697
Kojadinovic et al. 2021	0.56 [-2.69 to 3.81]	0.737	4	0.307

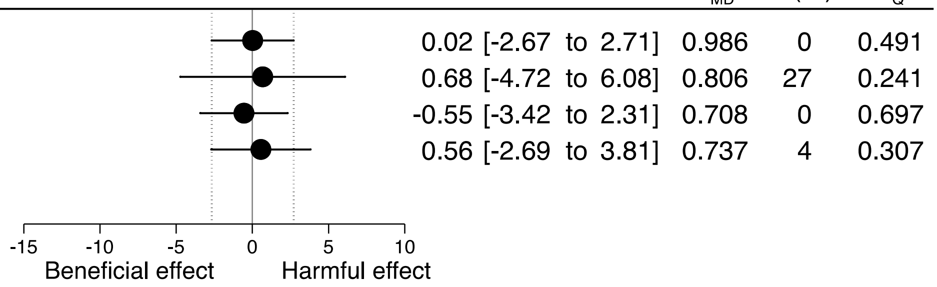

Influence analysis: Removal of each trial, one at a time and recalculation of the overall effect and heterogeneity

AST=aspartate aminotransferase; CI=confidence interval; MD=mean difference.

Supplementary Figure S43: Sensitivity analysis of the systematic removal of each trial for the effect of fruit on AST (U/L) in addition trials

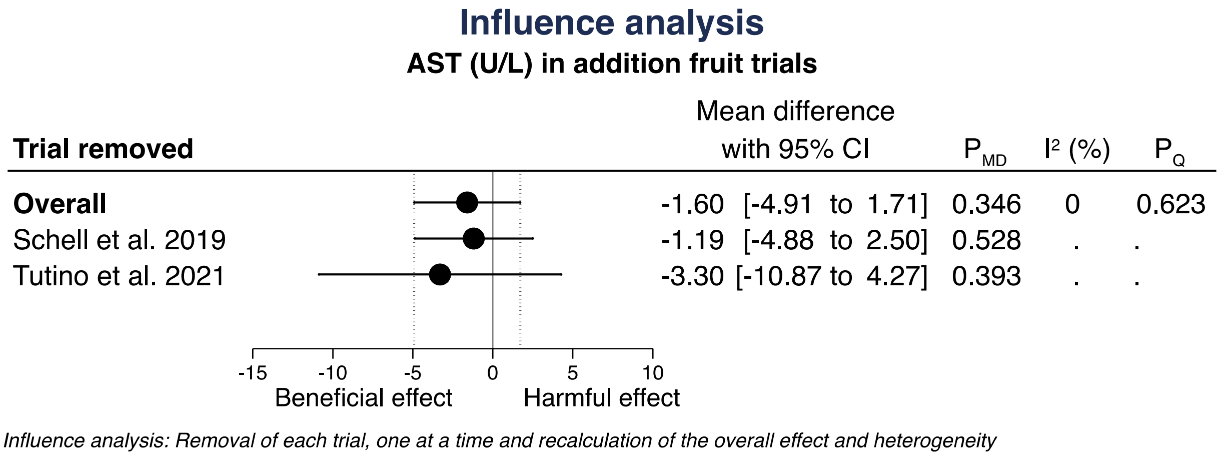

AST=aspartate aminotransferase; CI=confidence interval; MD=mean difference.

Supplementary Figure S44: Sensitivity analysis of the systematic removal of each trial for the effect of dried fruit on AST (U/L) in addition trials

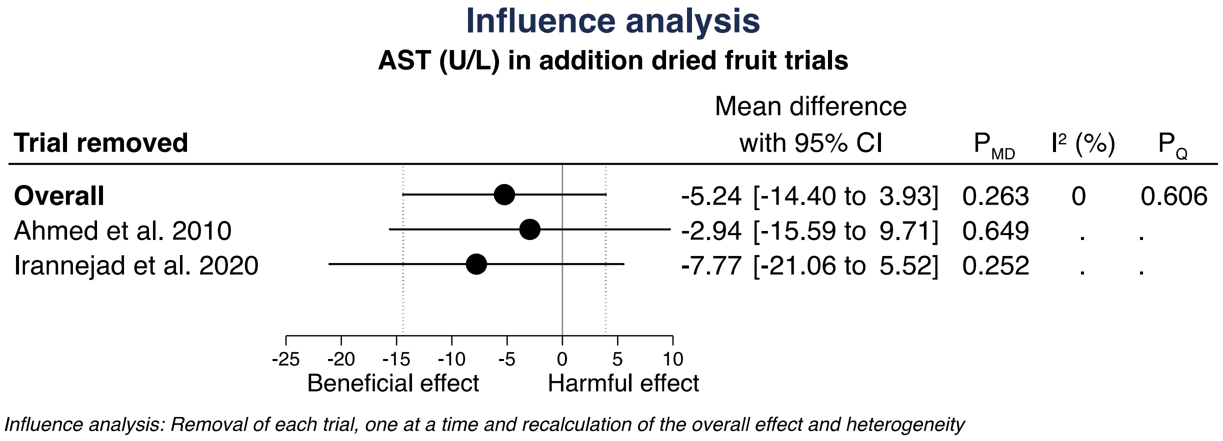

AST=aspartate aminotransferase; CI=confidence interval; MD=mean difference.

Supplementary Figure S45: Sensitivity analysis of the systematic removal of each trial for the effect of honey on AST (U/L) in addition trials

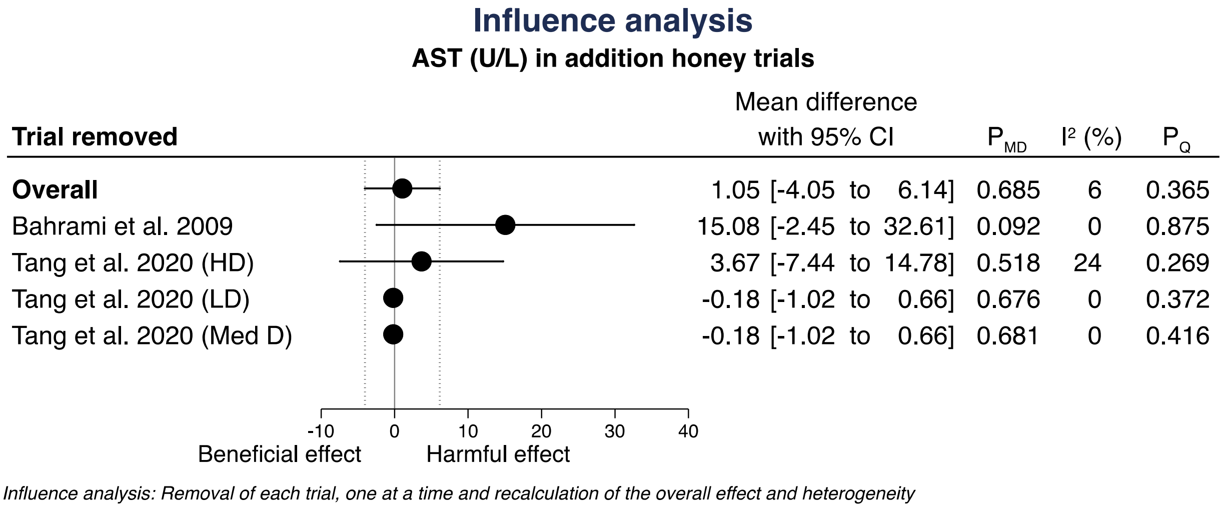

AST=aspartate aminotransferase; CI=confidence interval; HD=high dose; LD=low dose; MD=mean difference; Med D=medium dose.

Supplementary Figure S46: Sensitivity analysis of the systematic removal of each trial for the effect of SSB on AST (U/L) in subtraction trials

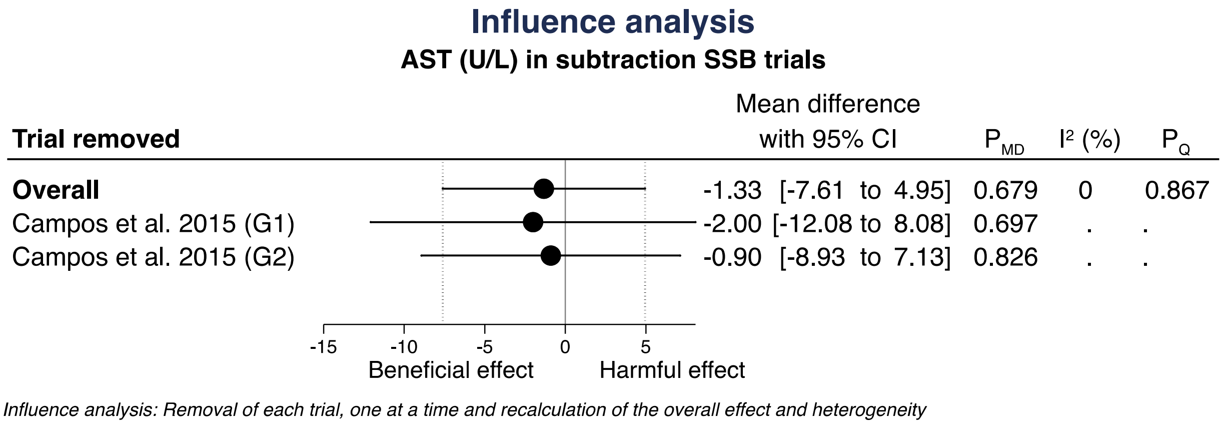

AST=aspartate aminotransferase; CI=confidence interval; G1=group 1; G2=group 2; MD=mean difference; SSB=sugar-sweetened beverage.

Supplementary Figure S47: Sensitivity analysis of the systematic removal of each trial for the effect of mixed sources (with SSBs) on AST (U/L) in subtraction trials

Influence analysis

AST (U/L) in subtraction mixed sources (with SSBs) trials

Trial removed	Mean difference with 95% CI	P_{MD}	I^2 (%)	P_Q
**Overall**	-7.33 [-12.78 to -1.88]	0.008	31	0.229
Porikos et al. 1983 (NonOB)	-11.50 [-20.15 to -2.85]	0.009	.	.
Porikos et al. 1983 (OB)	-5.47 [-10.09 to -0.85]	0.020	.	.

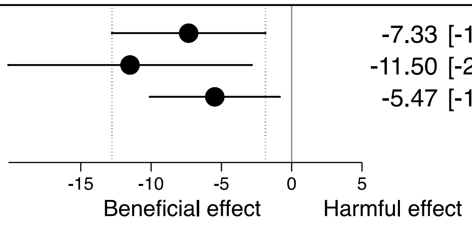

Influence analysis: Removal of each trial, one at a time and recalculation of the overall effect and heterogeneity

AST=aspartate aminotransferase; CI=confidence interval; MD=mean difference; NonOB=non-obese; SSB=sugar-sweetened beverage; OB=obese.

Supplementary Figure S48 (part 1 of 3): Subgroup analyses for the effect of important food sources of fructose-containing sugars and IHCL (SMD) in substitution trials

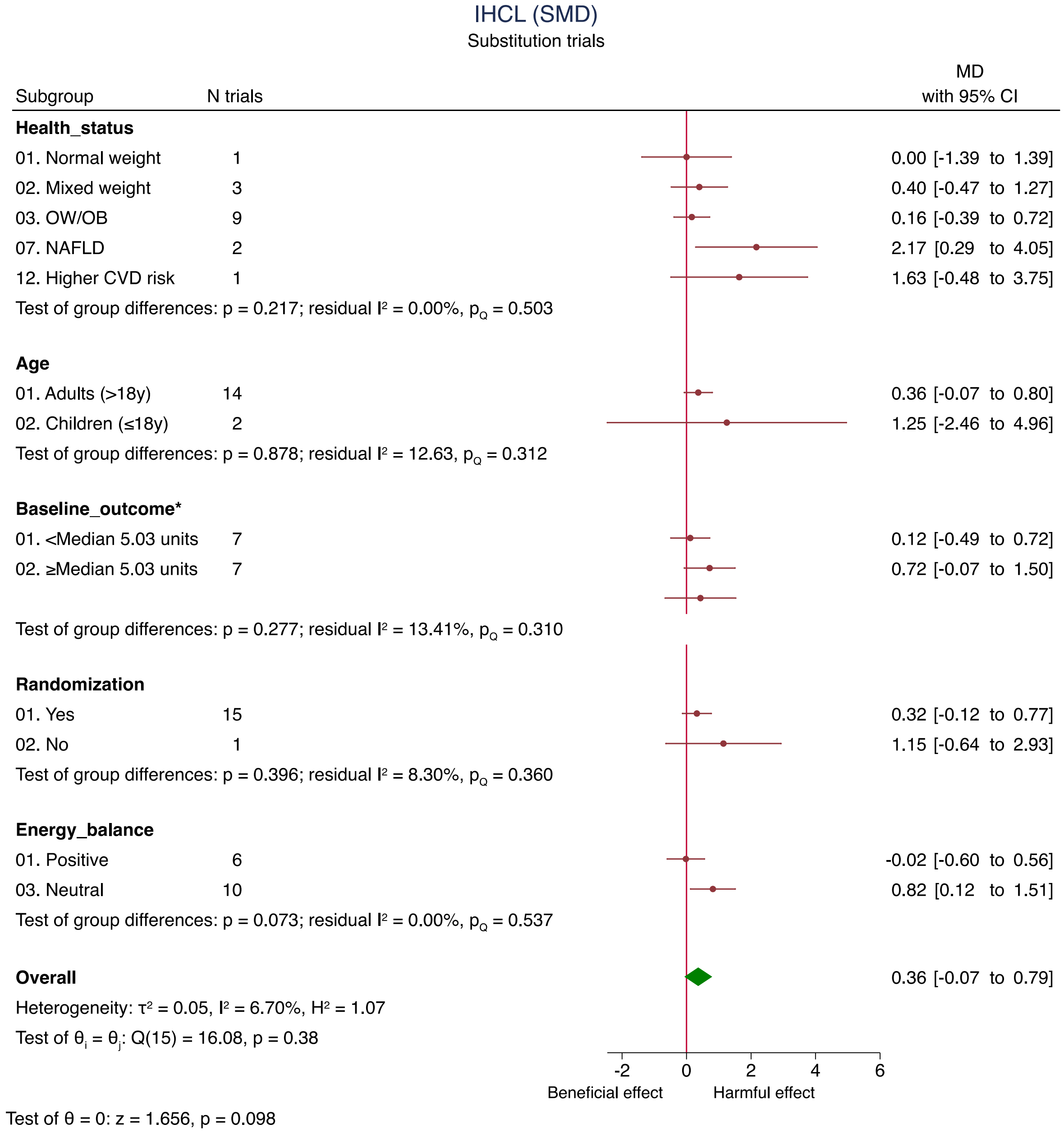

*N=2 trials missing data for baseline IHCL.

The green diamond represents the pooled estimate for the overall primary analysis of food sources of fructose-containing sugars and IHCL. Within subgroup mean differences are the pooled effect estimates represented by a red circle. 95% confidence intervals are represented by the line through the circle. Data are expressed as mean differences with 95% confidence intervals using the generic inverse-variance method and random effects DerSimonian-Laird model. Inter-study heterogeneity was assessed using the Cochrane Q statistic and quantified using the I^2 statistic, with significance set at $p<0.100$ and $I^2 \geq 50\%$ considered to be evidence of substantial heterogeneity. $p<0.050$ indicates that the effect size differed between levels of the subgroup.

CI=confidence interval; CVD=cardiovascular disease; IHCL=intrahepatocellular lipid; MD=mean difference; N=number; NAFLD=non-alcoholic fatty liver disease; OW/OB=overweight or obese; SMD=standardized mean difference; y=years.

Supplementary Figure S48 (part 2 of 3): Subgroup analyses for the effect of important food sources of fructose-containing sugars and IHCL (SMD) in substitution trials

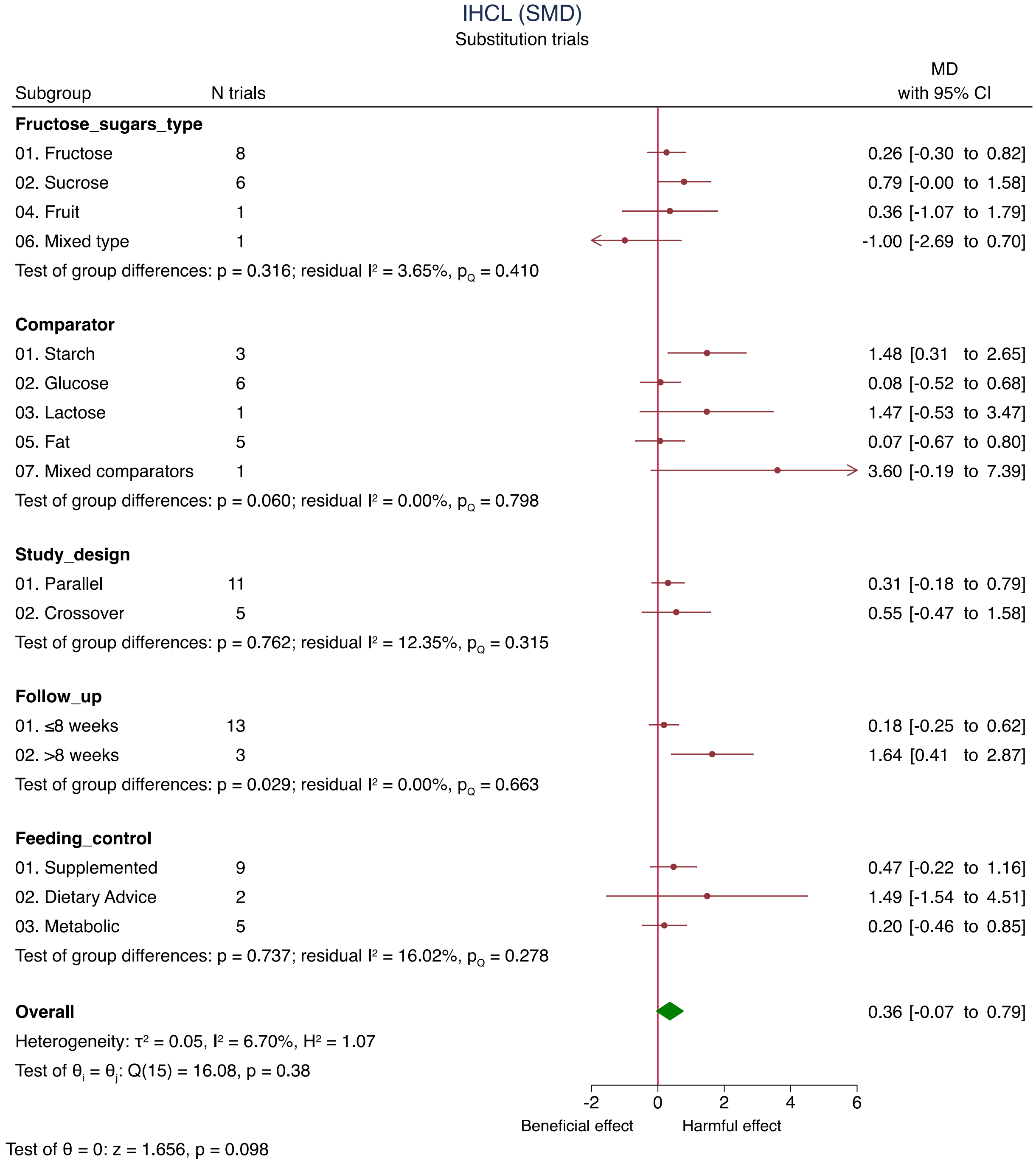

The green diamond represents the pooled estimate for the overall primary analysis of food sources of fructose-containing sugars and IHCL. Within subgroup mean differences are the pooled effect estimates represented by a red circle. 95% confidence intervals are represented by the line through the circle. Data are expressed as mean differences with 95% confidence intervals using the generic inverse-variance method and random effects DerSimonian-Laird model. Inter-study heterogeneity was assessed using the Cochrane Q statistic and quantified using the I^2 statistic, with significance set at p<0.100 and $I^2 \geq 50\%$ considered to be evidence of substantial heterogeneity. p<0.050 indicates that the effect size differed between levels of the subgroup. CI=confidence interval; IHCL=intrahepatocellular lipid; MD=mean difference; N=number; SMD=standardized mean difference.

Supplementary Figure S48 (part 3 of 3): Subgroup analyses for the effect of important food sources of fructose-containing sugars and IHCL (SMD) in substitution trials

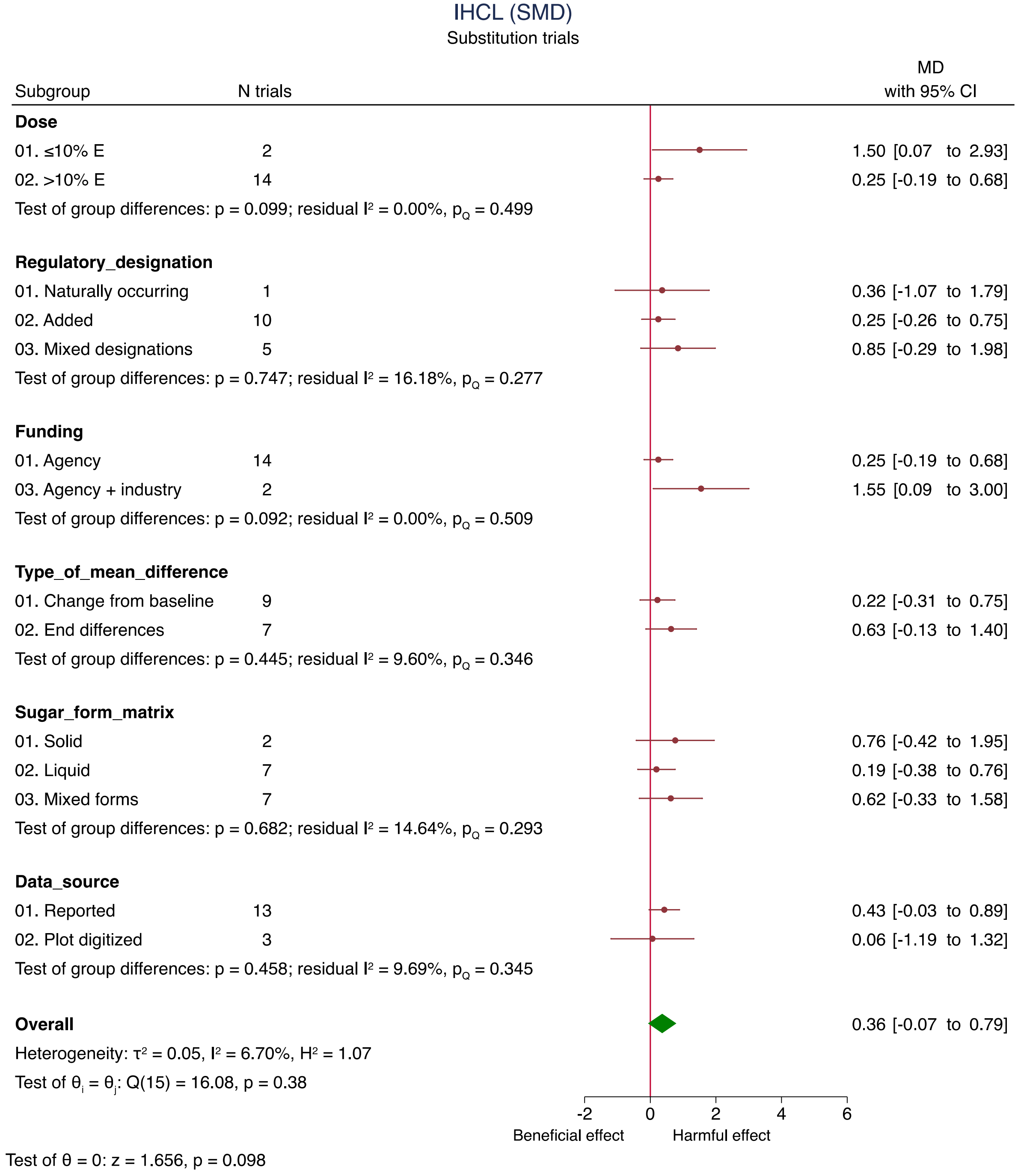

The green diamond represents the pooled estimate for the overall primary analysis of food sources of fructose-containing sugars and IHCL. Within subgroup mean differences are the pooled effect estimates represented by a red circle. 95% confidence intervals are represented by the line through the circle. Data are expressed as mean differences with 95% confidence intervals using the generic inverse-variance method and random effects DerSimonian-Laird model. Inter-study heterogeneity was assessed using the Cochrane Q statistic and quantified using the I^2 statistic, with significance set at $p<0.100$ and $I^2 \geq 50\%$ considered to be evidence of substantial heterogeneity. $p<0.050$ indicates that the effect size differed between levels of the subgroup.
CI=confidence interval; E=energy; IHCL=intrahepatocellular lipid; MD=mean difference; N=number; SMD=standardized mean difference.

Supplementary Figure S49: Risk of bias (using The Cochrane Collaboration Tool) subgroup analysis for the effect of important food sources of fructose-containing sugars and IHCL (SMD) in substitution trials

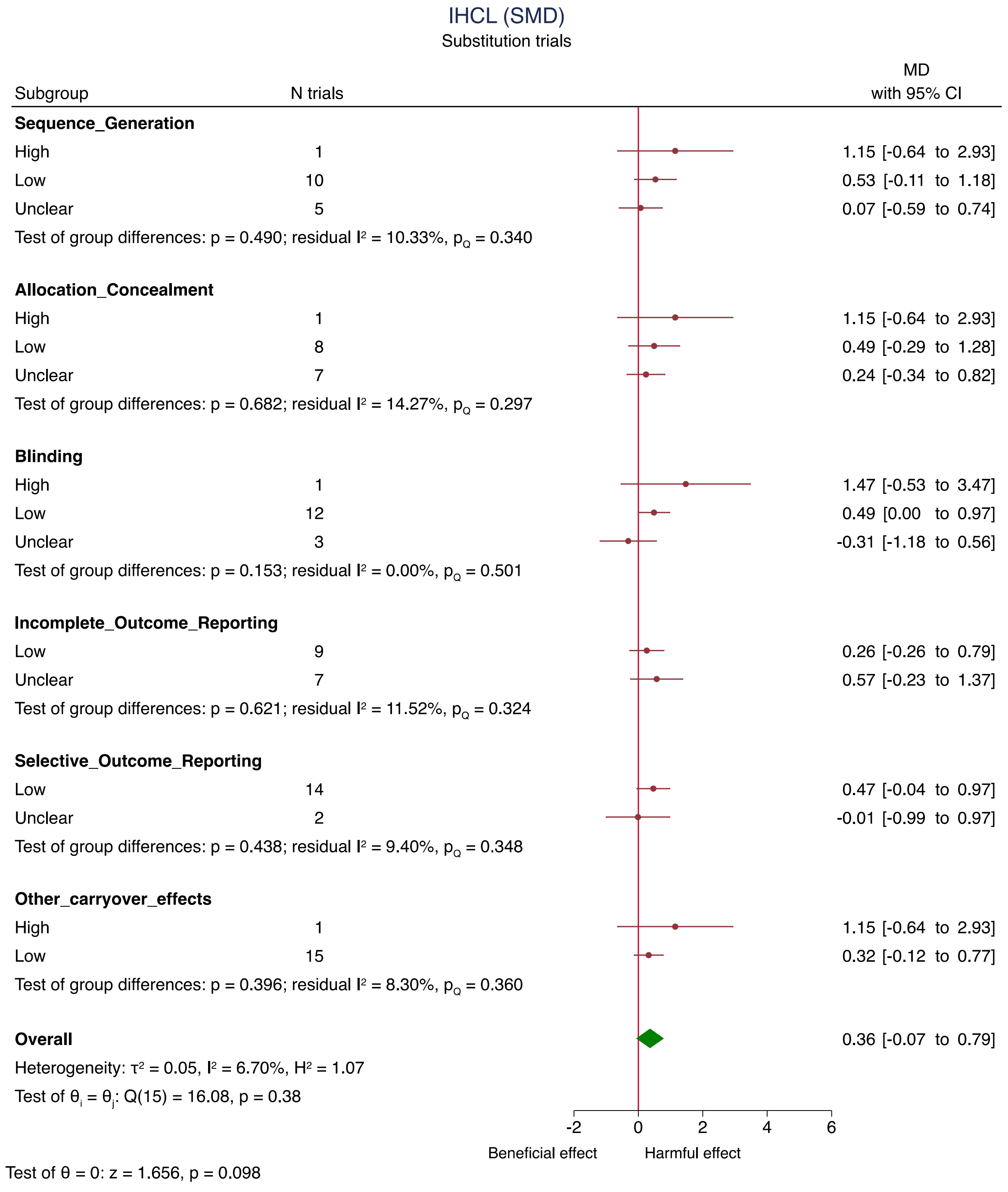

The green diamond represents the pooled estimate for the overall primary analysis of food sources of fructose-containing sugars and IHCL. Within subgroup mean differences are the pooled effect estimates represented by a red circle. 95% confidence intervals are represented by the line through the circle. Data are expressed as mean differences with 95% confidence intervals using the generic inverse-variance method and random effects DerSimonian-Laird model. Inter-study heterogeneity was assessed using the I^2 statistic, with significance set at $p<0.100$ and $I^2 \geq 50\%$ considered to be evidence of substantial heterogeneity. $p<0.050$ indicates that the effect size differed between levels of the subgroup.

CI=confidence interval; IHCL=intrahepatocellular lipid; MD=mean difference; N=number; SMD=standardized mean difference.

Supplementary Figure S50 (part 1 of 3): Subgroup analyses for the effect of important food sources of fructose-containing sugars and IHCL (SMD) in addition trials

IHCL (SMD)
Addition trials

Subgroup	N trials	MD with 95% CI
**Health_status**		
01. Normal weight	8	1.51 [0.75 to 2.27]
02. Mixed weight	2	2.96 [0.65 to 5.26]
03. OW/OB	3	1.98 [0.58 to 3.37]
Test of group differences: $p = 0.467$; residual $I^2 = 0.00\%$, $p_Q = 0.953$		
**Age**		
01. Adults (≥18y)	13	1.72 [1.08 to 2.36]
Test of group differences: p = .; residual I^2 = ., p_Q = .		
**Baseline_outcome***		
01. <Median 5.36 units	5	2.24 [1.04 to 3.43]
02. ≥Median 5.36 units	6	1.34 [0.54 to 2.14]
Test of group differences: $p = 0.220$; residual $I^2 = 0.00\%$, $p_Q = 0.986$		
**Randomization**		
01. Yes	7	1.56 [0.73 to 2.40]
02. No	6	1.94 [0.95 to 2.93]
Test of group differences: $p = 0.569$; residual $I^2 = 0.00\%$, $p_Q = 0.927$		
**Energy_balance**		
01. Positive	13	1.72 [1.08 to 2.36]
Test of group differences: p = .; residual I^2 = ., p_Q = .		
**Overall**		1.72 [1.08 to 2.36]
Heterogeneity: $\tau^2 = 0.00$, $I^2 = 0.00\%$, $H^2 = 1.00$		
Test of $\theta_i = \theta_j$: $Q(12) = 5.41$, $p = 0.94$		

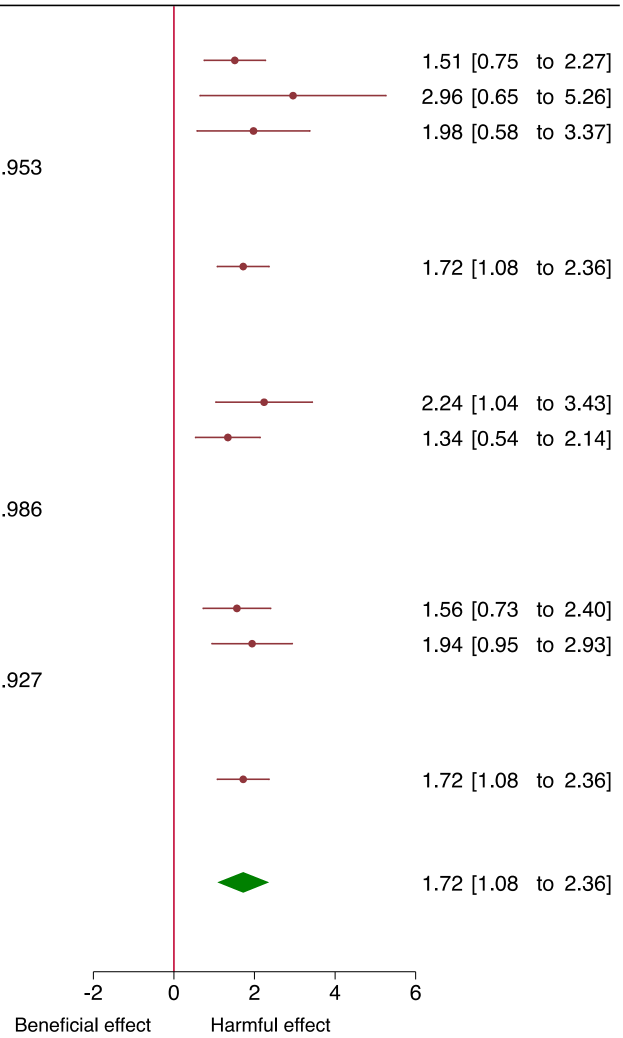

Test of $\theta = 0$: $z = 5.272$, $p = 0.000$

The green diamond represents the pooled estimate for the overall primary analysis of food sources of fructose-containing sugars and IHCL. Within subgroup mean differences are the pooled effect estimates represented by a red circle. 95% confidence intervals are represented by the line through the circle. Data are expressed as mean differences with 95% confidence intervals using the generic inverse-variance method and random effects DerSimonian-Laird model. Inter-study heterogeneity was assessed using the Cochrane Q statistic and quantified using the I^2 statistic, with significance set at $p<0.100$ and $I^2 \geq 50\%$ considered to be evidence of substantial heterogeneity. $p<0.050$ indicates that the effect size differed between levels of the subgroup.

*N=2 trials missing data for baseline IHCL.

CI=confidence interval; IHCL=intrahepatocellular lipid; MD=mean difference; N=number; OW/OB=overweight or obese; SMD=standardized mean difference; y=years.

Supplementary Figure S50 (part 2 of 3): A priori subgroup analyses for the effect of important food sources of fructose-containing sugars and IHCL (SMD) in addition trials

IHCL (SMD)

Addition trials

Subgroup	N trials	MD with 95% CI
**Fructose_sugars_type**		
01. Fructose	7	1.88 [0.98 to 2.78]
02. Sucrose	5	1.45 [0.49 to 2.41]
03. HFCS	1	2.54 [-0.31 to 5.40]
Test of group differences: p = 0.689; residual I^2 = 0.00%, p_Q = 0.913		
**Comparator**		
01. NNS	3	2.18 [0.64 to 3.72]
02. Water	1	2.69 [-0.29 to 5.67]
03. Diet alone	8	1.51 [0.75 to 2.27]
06. Mixed comparators	1	2.08 [-0.39 to 4.54]
Test of group differences: p = 0.771; residual I^2 = 0.00%, p_Q = 0.892		
**Study_design**		
01. Parallel	7	1.62 [0.77 to 2.47]
02. Crossover	6	1.85 [0.88 to 2.81]
Test of group differences: p = 0.733; residual I^2 = 0.00%, p_Q = 0.916		
**Follow_up**		
01. ≤8 weeks	11	1.69 [1.00 to 2.38]
02. >8 weeks	2	1.93 [0.23 to 3.62]
Test of group differences: p = 0.509; residual I^2 = 0.00%, p_Q = 0.945		
**Feeding_control**		
01. Supplemented	7	1.43 [0.66 to 2.20]
03. Metabolic	3	2.21 [0.65 to 3.78]
04. Metabolic + supplemented	3	2.55 [0.86 to 4.23]
Test of group differences: p = 0.394; residual I^2 = 0.00%, p_Q = 0.966		
**Overall**		1.72 [1.08 to 2.36]
Heterogeneity: τ^2 = 0.00, I^2 = 0.00%, H^2 = 1.00		
Test of $\theta_i = \theta_j$: Q(12) = 5.41, p = 0.94		

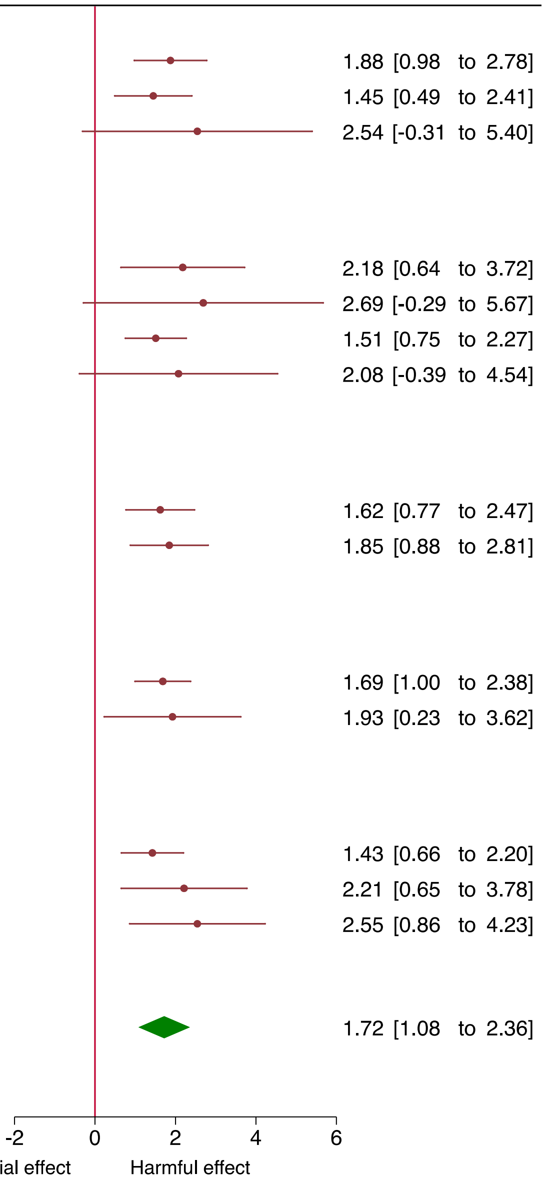

Test of θ = 0: z = 5.272, p = 0.000

The green diamond represents the pooled estimate for the overall primary analysis of food sources of fructose-containing sugars and IHCL. Within subgroup mean differences are the pooled effect estimates represented by a red circle. 95% confidence intervals are represented by the line through the circle. Data are expressed as mean differences with 95% confidence intervals using the generic inverse-variance method and random effects DerSimonian-Laird model. Inter-study heterogeneity was assessed using the Cochrane Q statistic and quantified using the I^2 statistic, with significance set at p<0.100 and $I^2 \geq 50\%$ considered to be evidence of substantial heterogeneity. p<0.050 indicates that the effect size differed between levels of the subgroup. CI=confidence interval; IHCL=intrahepatocellular lipid; MD=mean difference; NNS=non-nutritive sweetener; N=number; SMD=standardized mean difference.

Supplementary Figure S50 (part 3 of 3): A priori subgroup analyses for the effect of important food sources of fructose-containing sugars and IHCL (SMD) in addition trials

IHCL (SMD)

Addition trials

Subgroup	N trials	MD with 95% CI
**Dose**		
02. >10%E	13	1.72 [1.08 to 2.36]
Test of group differences: p = .; residual I² = ., p_Q = .		
**Regulatory_designation**		
02. Added	13	1.72 [1.08 to 2.36]
Test of group differences: p = .; residual I² = ., p_Q = .		
**Funding**		
01. Agency	9	1.56 [0.84 to 2.28]
03. Agency + industry	4	2.30 [0.92 to 3.67]
Test of group differences: p = 0.354; residual I² = 0.00%, p_Q = 0.951		
**Type_of_mean_difference**		
01. Change from baseline	12	1.69 [1.03 to 2.36]
02. End differences	1	2.09 [-0.38 to 4.56]
Test of group differences: p = 0.763; residual I² = 0.00%, p_Q = 0.915		
**Sugar_form_matrix**		
02. Liquid	13	1.72 [1.08 to 2.36]
Test of group differences: p = .; residual I² = ., p_Q = .		
**Data_source**		
01. Reported	9	1.66 [0.89 to 2.43]
02. Plot digitized	4	1.86 [0.70 to 3.02]
Test of group differences: p = 0.781; residual I² = 0.00%, p_Q = 0.914		
**Overall**		1.72 [1.08 to 2.36]
Heterogeneity: τ^2 = 0.00, I² = 0.00%, H² = 1.00		
Test of $\theta_i = \theta_j$: Q(12) = 5.41, p = 0.94		

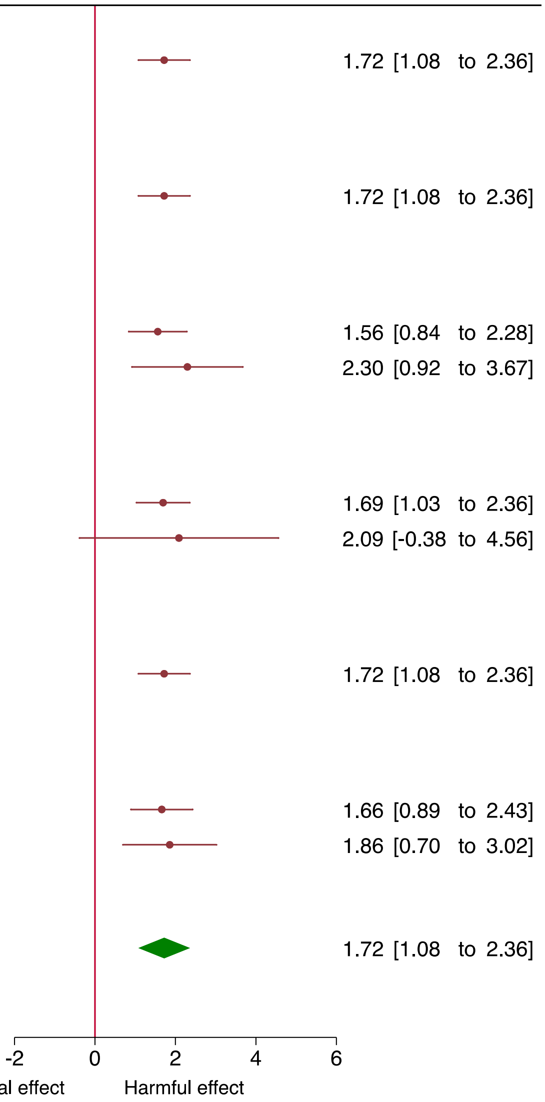

Test of θ = 0: z = 1.656, p = 0.098

The green diamond represents the pooled estimate for the overall primary analysis of food sources of fructose-containing sugars and IHCL. Within subgroup mean differences are the pooled effect estimates represented by a red circle. 95% confidence intervals are represented by the line through the circle. Data are expressed as mean differences with 95% confidence intervals using the generic inverse-variance method and random effects DerSimonian-Laird model. Inter-study heterogeneity was assessed using the Cochrane Q statistic and quantified using the I^2 statistic, with significance set at p<0.100 and I^2≥50% considered to be evidence of substantial heterogeneity. p<0.050 indicates that the effect size differed between levels of the subgroup. CI=confidence interval; %E=percent total energy intake; IHCL=intrahepatocellular lipid; MD=mean difference; N=number; SMD=standardized mean difference.

Supplementary Figure S51: Risk of bias (using The Cochrane Collaboration Tool) subgroup analysis for the effect of important food sources of fructose-containing sugars and IHCL (SMD) in addition trials

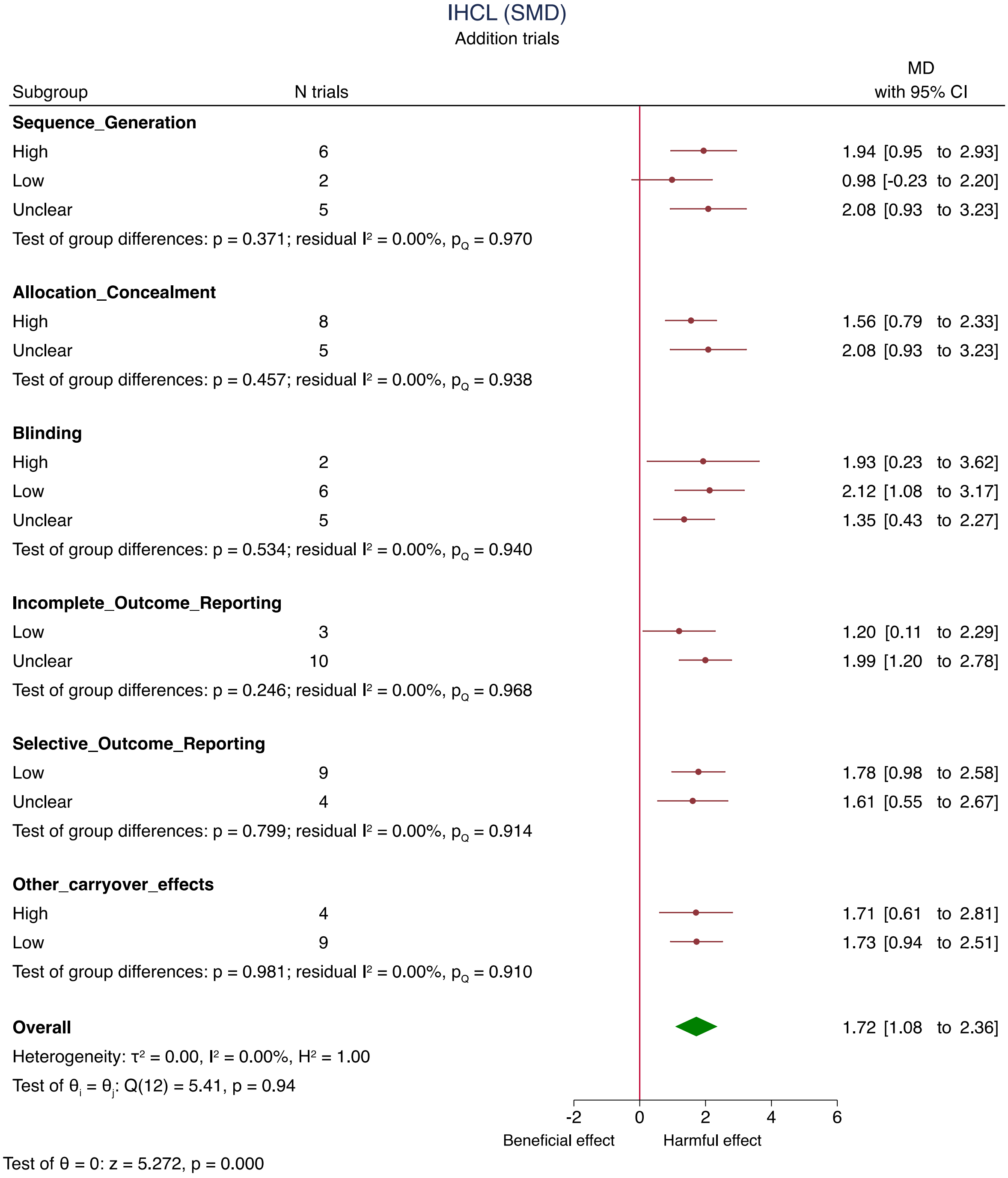

The green diamond represents the pooled estimate for the overall primary analysis of food sources of fructose-containing sugars and fasting serum uric acid levels. Within subgroup mean differences are the pooled effect estimates represented by a red circle. 95% confidence intervals are represented by the line through the circle. Data are expressed as mean differences with 95% confidence intervals using the generic inverse-variance method and random effects DerSimonian-Laird model. Inter-study heterogeneity was assessed using the Cochrane Q statistic and quantified using the I^2 statistic, with significance set at $p<0.100$ and $I^2 \geq 50\%$ considered to be evidence of substantial heterogeneity. $p<0.050$ indicates that the effect size differed between levels of the subgroup.
CI=confidence interval; IHCL=intrahepatocellular lipid; MD=mean difference; N=number; SMD=standardized mean difference.

Supplementary Figure S52 (part 1 of 3): Subgroup analyses for the effect of important food sources of fructose-containing sugars and ALT (U/L) in substitution trials

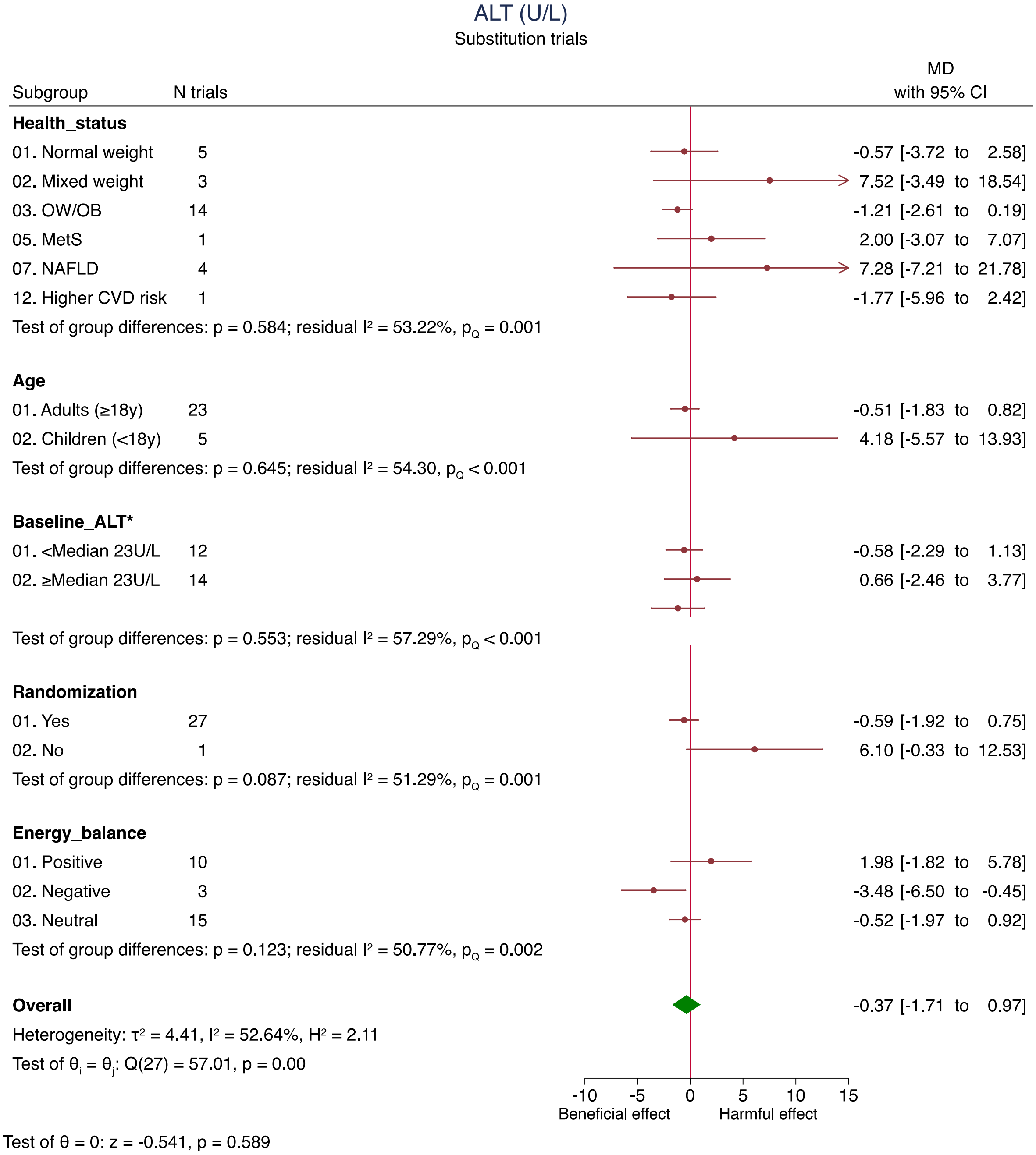

The green diamond represents the pooled estimate for the overall primary analysis of food sources of fructose-containing sugars and ALT. Within subgroup mean differences are the pooled effect estimates represented by a red circle. 95% confidence intervals are represented by the line through the circle. Data are expressed as mean differences with 95% confidence intervals using the generic inverse-variance method and random effects DerSimonian-Laird model. Inter-study heterogeneity was assessed using the Cochrane Q statistic and quantified using the I^2 statistic, with significance set at $p<0.100$ and $I^2 \geq 50\%$ considered to be evidence of substantial heterogeneity. $p<0.050$ indicates that the effect size differed between levels of the subgroup.
*N=2 trials missing data for baseline ALT.
ALT=alanine aminotransferase; AST=aspartate aminotransferase; CI=confidence interval; CVD=cardiovascular disease; MD=mean difference; MetS=metabolic syndrome; N=number; NAFLD=non-alcoholic fatty liver disease; OW/OB=overweight or obese; y=years.

Supplementary Figure S52 (part 2 of 3): Subgroup analyses for the effect of important food sources of fructose-containing sugars and ALT (U/L) in substitution trials

ALT (U/L)

Substitution trials

Subgroup	N trials	MD with 95% CI
**Fructose_sugars_type**		
01. Fructose	9	1.14 [-1.48 to 3.76]
02. Sucrose	9	-0.22 [-3.76 to 3.32]
03. HFCS	2	12.52 [-16.29 to 41.34]
04. Fruit	7	-1.20 [-2.94 to 0.53]
06. Mixed type	1	-2.00 [-4.95 to 0.95]
Test of group differences: p = 0.686; residual I^2 = 54.39%, $p_Q < 0.001$		
**Comparator**		
02. Glucose	10	0.74 [-0.15 to 1.62]
03. Lactose	1	-1.20 [-3.89 to 1.49]
05. Fat	7	1.23 [-3.21 to 5.67]
07. Mixed comparators	10	-1.41 [-4.02 to 1.20]
Test of group differences: p = 0.571; residual I^2 = 50.80%, p_Q = 0.002		
**Study_design**		
01. Parallel	17	-0.54 [-2.95 to 1.86]
02. Crossover	11	-0.03 [-1.52 to 1.46]
Test of group differences: p = 0.482; residual I^2 = 49.76%, p_Q = 0.002		
**Follow_up**		
01. ≤8 weeks	21	-0.15 [-1.62 to 1.32]
02. >8 weeks	7	-0.89 [-4.04 to 2.26]
Test of group differences: p = 0.359; residual I^2 = 48.87%, p_Q = 0.003		
**Feeding_control**		
01. Supplemented	17	-1.25 [-2.18 to -0.31]
02. Dietary advice	3	12.99 [-15.50 to 41.48]
03. Metabolic	7	2.39 [-2.37 to 7.14]
04. Metabolic + supplemented	1	6.10 [-0.33 to 12.53]
Test of group differences: p = 0.055; residual I^2 = 43.79%, p_Q = 0.011		
**Overall**		-0.37 [-1.71 to 0.97]
Heterogeneity: τ^2 = 4.41, I^2 = 52.64%, H^2 = 2.11		
Test of $\theta_i = \theta_j$: Q(27) = 57.01, p = 0.00		

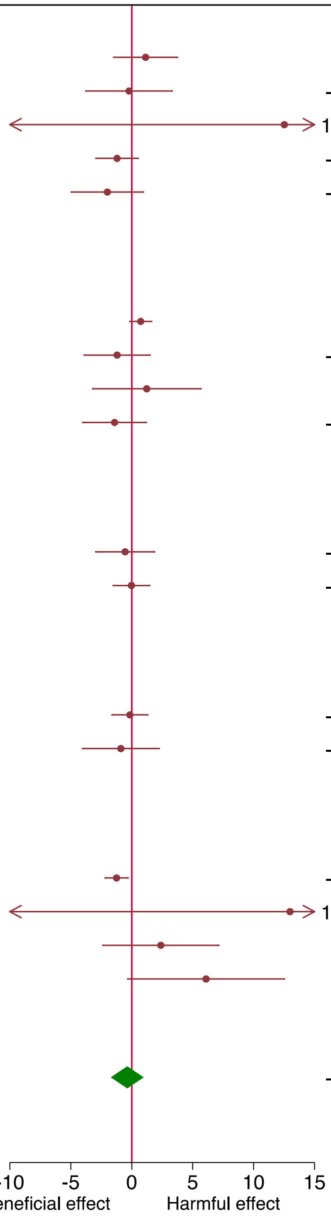

Test of θ = 0: z = -0.541, p = 0.589

The green diamond represents the pooled estimate for the overall primary analysis of food sources of fructose-containing sugars and ALT. Within subgroup mean differences are the pooled effect estimates represented by a red circle. 95% confidence intervals are represented by the line through the circle. Data are expressed as mean differences with 95% confidence intervals using the generic inverse-variance method and random effects DerSimonian-Laird model. Inter-study heterogeneity was assessed using the Cochrane Q statistic and quantified using the I^2 statistic, with significance set at p<0.100 and $I^2 \geq 50\%$ considered to be evidence of substantial heterogeneity. p<0.050 indicates that the effect size differed between levels of the subgroup.
ALT=alanine aminotransferase; CI=confidence interval; HFCS=high fructose corn syrup; MD=mean difference; N=number; y=years.

Supplementary Figure S52 (part 3 of 3): A priori subgroup analyses for the effect of important food sources of fructose-containing sugars and ALT (U/L) in substitution trials

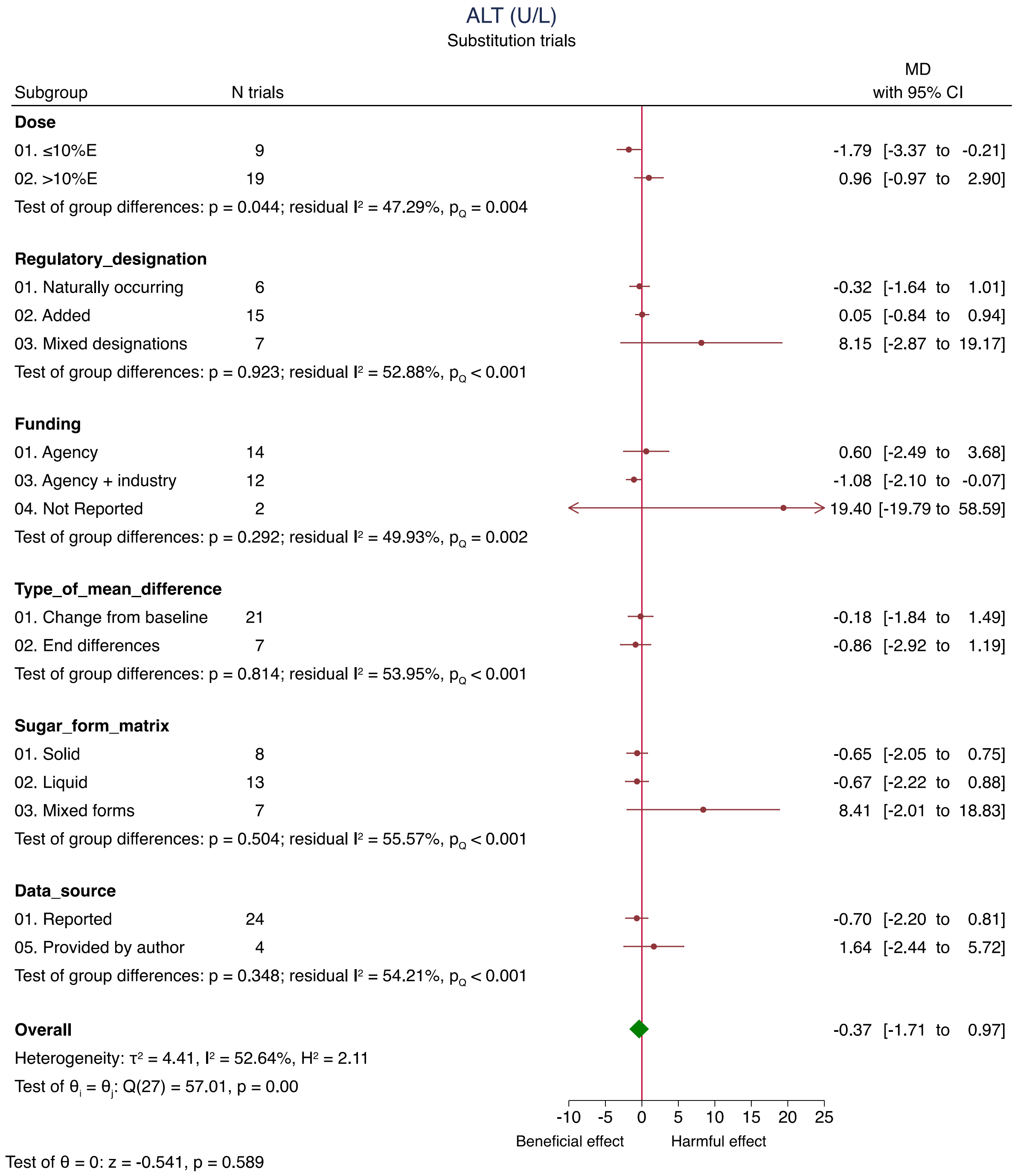

The green diamond represents the pooled estimate for the overall primary analysis of food sources of fructose-containing sugars and ALT. Within subgroup mean differences are the pooled effect estimates represented by a red circle. 95% confidence intervals are represented by the line through the circle. Data are expressed as mean differences with 95% confidence intervals using the generic inverse-variance method and random effects DerSimonian-Laird model. Inter-study heterogeneity was assessed using the Cochrane Q statistic and quantified using the I^2 statistic, with significance set at $p<0.100$ and $I^2 \geq 50\%$ considered to be evidence of substantial heterogeneity. $p<0.050$ indicates that the effect size differed between levels of the subgroup.
ALT=alanine aminotransferase; CI=confidence interval; %E= percentage of total energy intake; MD=mean difference; N=number.

Supplementary Figure S53: Risk of bias (using The Cochrane Collaboration Tool) subgroup analysis for the effect of important food sources of fructose-containing sugars and ALT (U/L) in substitution trials

ALT (U/L)

Substitution trials

Subgroup	N trials	MD with 95% CI
**Sequence_Generation**		
High	1	-3.00 [-11.77 to 5.77]
Low	11	-0.96 [-3.25 to 1.32]
Unclear	16	0.21 [-1.53 to 1.95]
Test of group differences: p = 0.567; residual I^2 = 52.47%, p_Q = 0.001		
**Allocation_Concealment**		
High	2	-5.59 [-10.86 to -0.32]
Low	6	1.02 [-2.77 to 4.81]
Unclear	20	-0.27 [-1.75 to 1.20]
Test of group differences: p = 0.232; residual I^2 = 52.22%, p_Q = 0.001		
**Blinding**		
Low	16	0.33 [-1.93 to 2.59]
Unclear	12	-0.96 [-2.47 to 0.54]
Test of group differences: p = 0.497; residual I^2 = 50.97%, p_Q = 0.001		
**Incomplete_Outcome_Reporting**		
Low	14	-0.37 [-1.86 to 1.12]
Unclear	14	-0.29 [-2.61 to 2.02]
Test of group differences: p = 0.841; residual I^2 = 54.11%, p_Q < 0.001		
**Selective_Outcome_Reporting**		
High	2	-0.06 [-1.58 to 1.46]
Low	20	-0.57 [-1.97 to 0.84]
Unclear	6	3.99 [-3.75 to 11.74]
Test of group differences: p = 0.897; residual I^2 = 54.27%, p_Q < 0.001		
**Other_carryover_effects**		
Low	28	-0.37 [-1.71 to 0.97]
Test of group differences: p = 0.981; residual I^2 = 0.00%, p_Q = 0.910		
**Overall**		-0.37 [-1.71 to 0.97]
Heterogeneity: τ^2 = 4.41, I^2 = 52.64%, H^2 = 2.11		
Test of $\theta_i = \theta_j$: Q(27) = 57.01, p = 0.00		

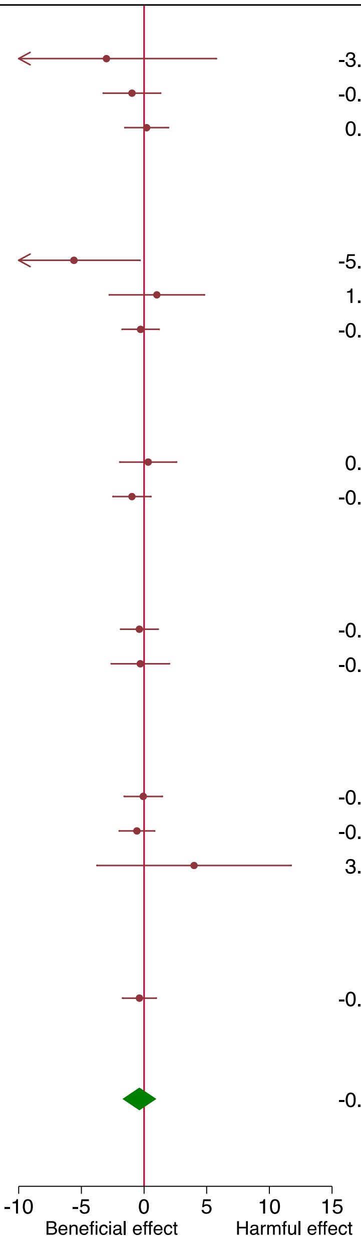

Test of θ = 0: z = -0.541, p = 0.589

The green diamond represents the pooled estimate for the overall primary analysis of food sources of fructose-containing sugars and ALT. Within subgroup mean differences are the pooled effect estimates represented by a red circle. 95% confidence intervals are represented by the line through the circle. Data are expressed as mean differences with 95% confidence intervals using the generic inverse-variance method and random effects DerSimonian-Laird model. Inter-study heterogeneity was assessed using the Cochrane Q statistic and quantified using the I^2 statistic, with significance set at p<0.100 and I^2≥50% considered to be evidence of substantial heterogeneity. p<0.050 indicates that the effect size differed between levels of the subgroup. ALT=alanine aminotransferase; CI=confidence interval; MD=mean difference; N=number.

Supplementary Figure S54 (part 1 of 3): A priori subgroup analyses for the effect of important food sources of fructose-containing sugars and ALT (U/L) in addition trials

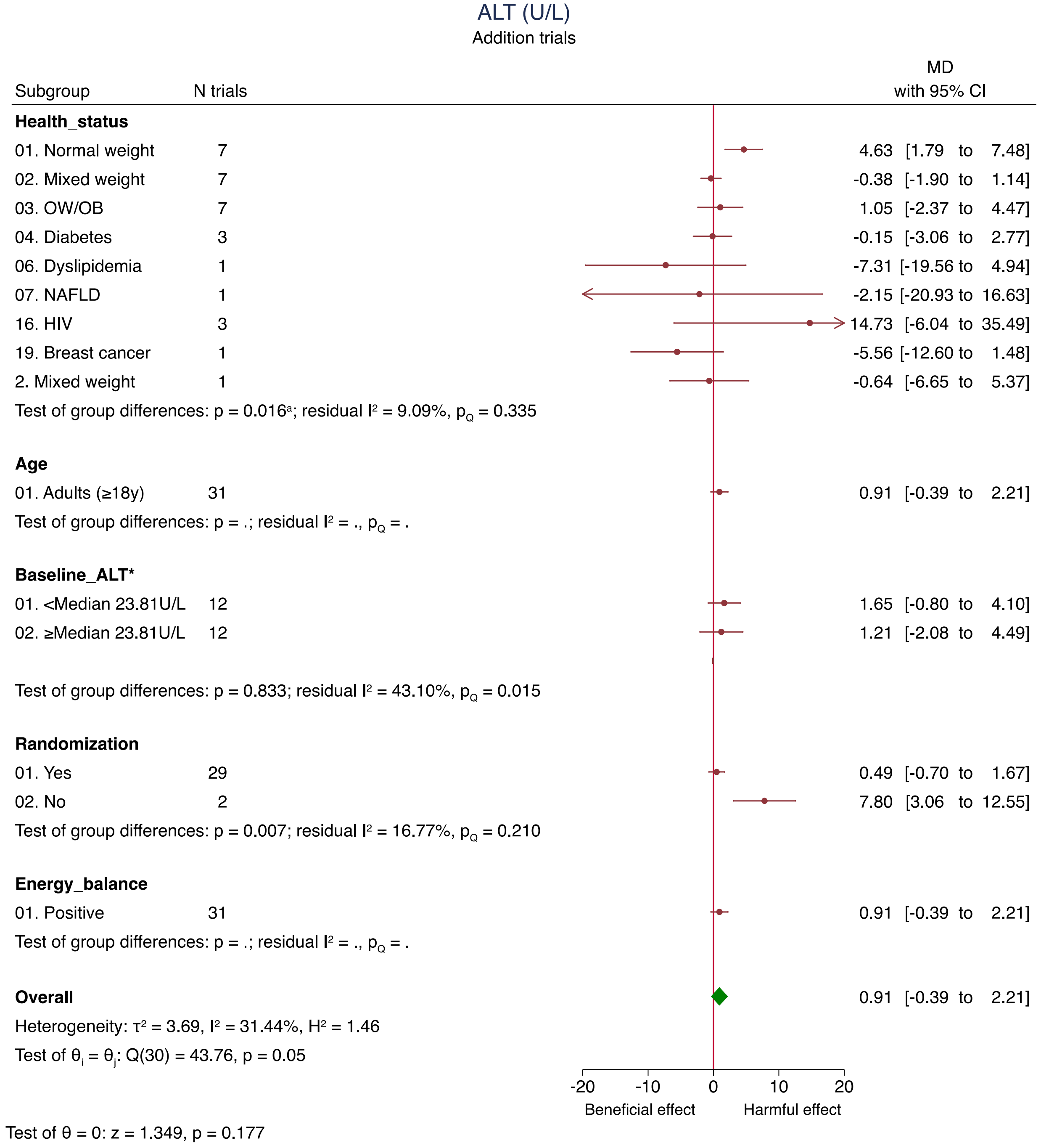

The green diamond represents the pooled estimate for the overall primary analysis of food sources of fructose-containing sugars and ALT. Within subgroup mean differences are the pooled effect estimates represented by a red circle. 95% confidence intervals are represented by the line through the circle. Data are expressed as mean differences with 95% confidence intervals using the generic inverse-variance method and random effects DerSimonian-Laird model. Inter-study heterogeneity was assessed using the Cochrane Q statistic and quantified using the I^2 statistic, with significance set at $p<0.100$ and $I^2 \geq 50\%$ considered to be evidence of substantial heterogeneity. $p<0.050$ indicates that the effect size differed between levels of the subgroup.
*N=7 trials missing data for baseline ALT.

[a] Pairwise between-subgroup mean differences (95% CIs) for Health status were as follows: (**2 vs 1**) -4.92U/L (-7.96, -1.88U/L); (3 vs 1) -3.21U/L (-6.54, 0.12U/L); (**4 vs 1**) -4.72U/L (-8.86, -0.585U/L); (6 vs 1) -11.8U/L (-24.5, 0.79U/L); (7 vs 1) -6.69U/L (-25.7, 12.3U/L); (16 vs 1) 10.2U/L (-10.8, 31.2U/L); (**19 vs 1**) -10.1U/L (-17.8, -2.4U/L); (3 vs 2) 1.71U/L (-0.958, 4.38U/L); (4 vs 2) 0.195U/L (-3.43, 3.82U/L); (6 vs 2) -6.93U/L (-19.4, 5.55U/L); (7 vs 2) -1.77U/L (-20.7, 17.2U/L); (16 vs 2) 15.1U/L (-5.74, 36U/L); (19 vs 2) -5.18U/L (-12.6, 2.25U/L); (4 vs 3) -1.51U/L (-5.39, 2.36U/L); (6 vs 3) -8.64U/L (-21.2, 3.91U/L); (7 vs 3) -3.48U/L (-22.5, 15.5U/L); (16 vs 3) 13.4U/L (-7.5, 34.3U/L); (19 vs 3) -6.89U/L (-14.4, 0.668U/L); (6 vs 4) -7.12U/L (-19.9, 5.67U/L); (7 vs 4) -1.96U/L (-21.1, 17.2U/L); (16 vs 4) 14.9U/L (-6.13, 36U/L); (19 vs 4) -5.37U/L (-13.3, 2.57U/L); (7 vs 6) 5.16U/L (-17.4, 27.7U/L); (16 vs 6) 22U/L (-2.15, 46.2U/L); (19 vs 6) 1.75U/L (-12.6, 16.1U/L); (16 vs 7) 16.9U/L (-11.2, 45U/L); (19 vs 7) -3.41U/L (-23.6, 16.8U/L); (19 vs 16) -20.3U/L (-42.3, 1.73U/L).
ALT=alanine aminotransferase; CI=confidence interval; HIV=human immunodeficiency virus; MD=mean difference; N=number; NAFLD=non-alcoholic fatty liver disease; OW/OB=overweight or obese; y=years.

Supplementary Figure S54 (part 2 of 3): A priori subgroup analyses for the effect of important food sources of fructose-containing sugars and ALT (U/L) in addition trials

ALT (U/L)

Addition trials

Subgroup	N trials	MD with 95% CI
**Fructose_sugars_type**		
01. Fructose	9	3.74 [0.22 to 7.26]
02. Sucrose	4	1.58 [-1.25 to 4.41]
04. Fruit	13	-0.48 [-1.86 to 0.91]
05. Honey	5	-0.87 [-5.33 to 3.59]
Test of group differences: $p = 0.032^a$; residual $I^2 = 17.59\%$, $p_Q = 0.205$		
**Comparator**		
01. NNS	2	1.54 [-3.36 to 6.44]
02. Water	2	-5.59 [-15.25 to 4.08]
03. Diet alone	25	0.49 [-0.78 to 1.76]
06. Mixed comparators	2	7.16 [-0.27 to 14.58]
Test of group differences: $p = 0.059$; residual $I^2 = 20.29\%$, $p_Q = 0.170$		
**Study_design**		
01. Parallel	15	0.91 [-1.42 to 3.23]
02. Crossover	16	0.90 [-0.72 to 2.53]
Test of group differences: $p = 0.997$; residual $I^2 = 33.38\%$, $p_Q = 0.041$		
**Follow_up**		
01. ≤8 weeks	23	0.99 [-0.45 to 2.43]
02. >8 weeks	8	0.47 [-3.16 to 4.11]
Test of group differences: $p = 0.069$; residual $I^2 = 25.78\%$, $p_Q = 0.104$		
**Feeding_control**		
01. Supplemented	27	-0.08 [-1.12 to 0.96]
03. Metabolic	3	7.75 [2.54 to 12.96]
04. Metabolic + supplemented	1	8.70 [3.32 to 14.08]
Test of group differences: $p < 0.001^b$; residual $I^2 = 0.00\%$, $p_Q = 0.817$		
**Overall**		0.91 [-0.39 to 2.21]
Heterogeneity: $\tau^2 = 3.69$, $I^2 = 31.44\%$, $H^2 = 1.46$		
Test of $\theta_i = \theta_j$: $Q(30) = 43.76$, $p = 0.05$		

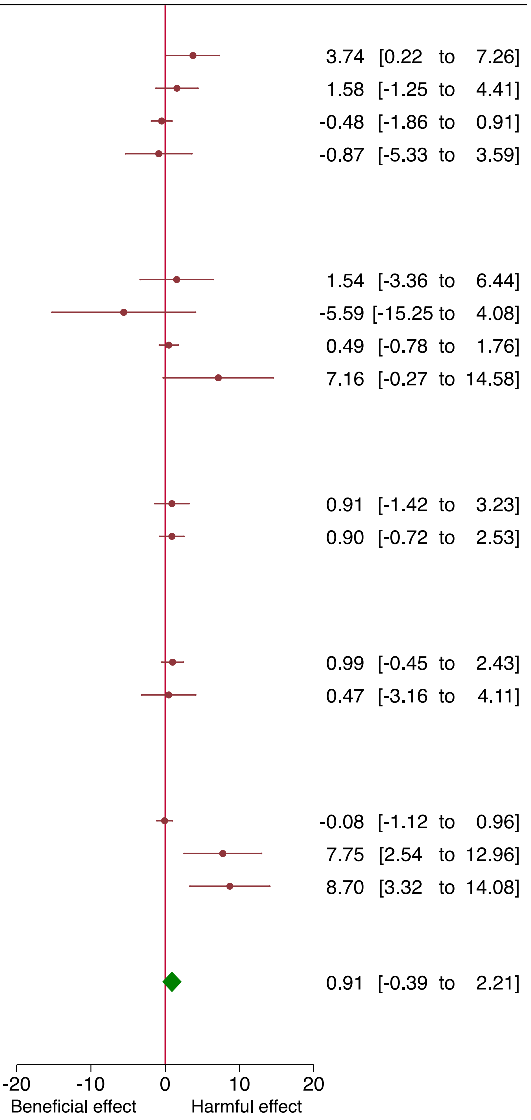

Test of $\theta = 0$: $z = 1.349$, $p = 0.177$

The green diamond represents the pooled estimate for the overall primary analysis of food sources of fructose-containing sugars and ALT. Within subgroup mean differences are the pooled effect estimates represented by a red circle. 95% confidence intervals are represented by the line through the circle. Data are expressed as mean differences with 95% confidence intervals using the generic inverse-variance method and random effects DerSimonian-Laird model. Inter-study heterogeneity was assessed using the Cochrane Q statistic and quantified using the I^2 statistic, with significance set at p<0.100 and $I^2 \geq 50\%$ considered to be evidence of substantial heterogeneity. p<0.050 indicates that the effect size differed between levels of the subgroup.

[a] Pairwise between-subgroup mean differences (95% CIs) for fructose sugars type were as follows: (2 vs 1) -2U/L (-5.77, 1.77U/L); (**4 vs 1**) -4.05U/L (-6.88, -1.21U/L); (**5 vs 1**) -4.33U/L (-8.61, -0.042U/L); (4 vs 2) -2.05U/L (-5.48, 1.39U/L); (5 vs 2) -2.33U/L (-7.03, 2.38U/L); (5 vs 4) -0.28U/L (-4.27, 3.71U/L).

[b]Pairwise between-subgroup mean differences (95%CIs) for feeding control were as follows: (**2 vs 1**) 7.26U/L (3.44, 11.1U/L); (**4 vs 1**) 8.78U/L (3.3, 14.3U/L); (4 vs 3) 1.52U/L (-5, 8.04U/L).

Supplementary Figure S54 (part 3 of 3): A priori subgroup analyses for the effect of important food sources of fructose-containing sugars and ALT (U/L) in addition trials

ALT (U/L)

Addition trials

Subgroup	N trials	MD with 95% CI
**Dose**		
01. ≤10%E	16	0.50 [-1.18 to 2.19]
02. >10%E	15	1.52 [-0.43 to 3.48]
Test of group differences: p = 0.427; residual I² = 33.55%, p_Q = 0.040		
**Regulatory_designation**		
01. Naturally occurring	13	-0.48 [-1.86 to 0.91]
02. Added	18	2.33 [0.06 to 4.60]
Test of group differences: p = 0.034; residual I² = 23.41%, p_Q = 0.125		
**Funding**		
01. Agency	22	0.34 [-1.01 to 1.68]
02. Industry	1	-0.64 [-6.65 to 5.37]
03. Agency + industry	8	3.56 [0.22 to 6.90]
Test of group differences: p = 0.143; residual I² = 27.64%, p_Q = 0.086		
**Type_of_mean_difference**		
01. Change from baseline	23	1.15 [-0.47 to 2.76]
02. End differences	8	0.45 [-1.81 to 2.70]
Test of group differences: p = 0.637; residual I² = 33.60%, p_Q = 0.039		
**Sugar_form_matrix**		
01. Solid	7	0.30 [-2.29 to 2.89]
02. Liquid	24	1.15 [-0.47 to 2.76]
Test of group differences: p = 0.611; residual I² = 33.53%, p_Q = 0.040		
**Data_source**		
01. Reported	30	0.92 [-0.41 to 2.25]
05. Provided by author	1	0.70 [-11.44 to 12.84]
Test of group differences: p = 0.973; residual I² = 33.72%, p_Q = 0.039		
**Overall**		0.91 [-0.39 to 2.21]
Heterogeneity: τ² = 3.69, I² = 31.44%, H² = 1.46		
Test of $\theta_i = \theta_j$: Q(30) = 43.76, p = 0.05		

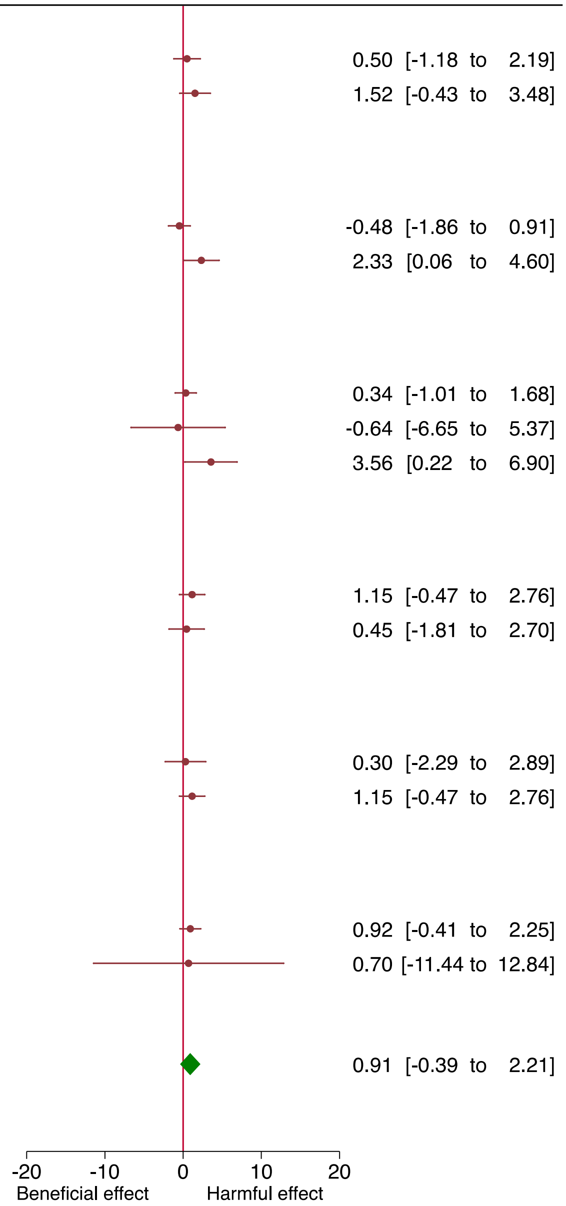

Test of θ = 0: z = 1.349, p = 0.177

The green diamond represents the pooled estimate for the overall primary analysis of food sources of fructose-containing sugars and ALT. Within subgroup mean differences are the pooled effect estimates represented by a red circle. 95% confidence intervals are represented by the line through the circle. Data are expressed as mean differences with 95% confidence intervals using the generic inverse-variance method and random effects DerSimonian-Laird model. Inter-study heterogeneity was assessed using the Cochrane Q statistic and quantified using the I^2 statistic, with significance set at p<0.100 and I^2≥50% considered to be evidence of substantial heterogeneity. p<0.050 indicates that the effect size differed between levels of the subgroup.
ALT=alanine aminotransferase; CI=confidence interval; %E=percentage of total energy intake; MD=mean difference; NAFLD=non-alcoholic fatty liver disease; N=number.

Supplementary Figure S55: Risk of bias (using The Cochrane Collaboration Tool) subgroup analysis for the effect of important food sources of fructose-containing sugars and ALT (U/L) in addition trials

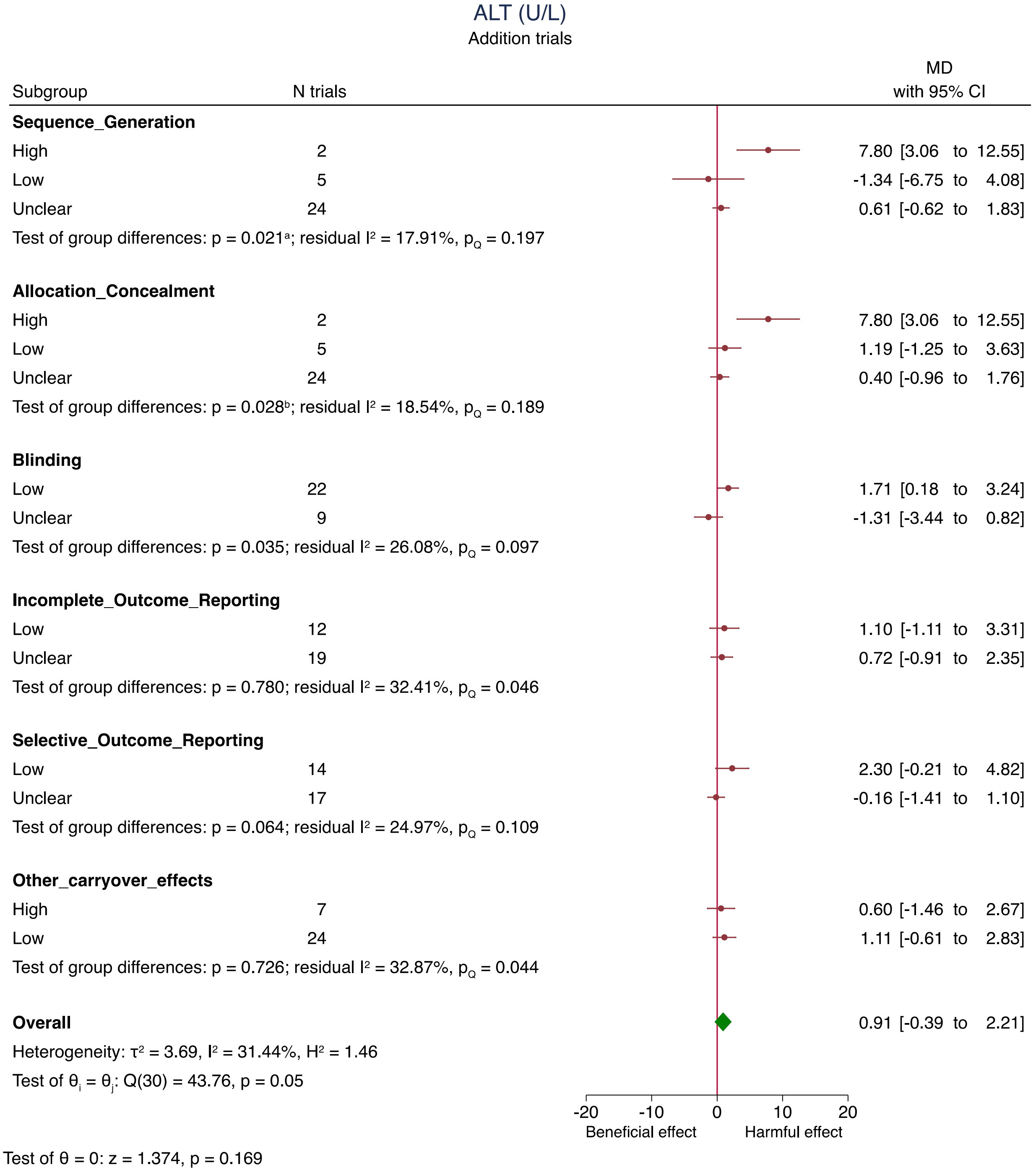

The green diamond represents the pooled estimate for the overall primary analysis of food sources of fructose-containing sugars and ALT. Within subgroup mean differences are the pooled effect estimates represented by a red circle. 95% confidence intervals are represented by the line through the circle. Data are expressed as mean differences with 95% confidence intervals using the generic inverse-variance method and random effects DerSimonian-Laird model. Inter-study heterogeneity was assessed using the Cochrane Q statistic and quantified using the I^2 statistic, with significance set at $p<0.100$ and $I^2 \geq 50\%$ considered to be evidence of substantial heterogeneity. $p<0.050$ indicates that the effect size differed between levels of the subgroup.

[a] Pairwise between-subgroup mean differences (95% CIs) for sequence generation were as follows: (**Low vs High**) -9.18U/L (-16.3, -2.01U/L); (**Unclear vs High**) -7.1U/L (-12.4, -1.78U/L); (Unclear vs Low) 2.09U/L (-3.02, 7.19U/L).

[b] Pairwise between-subgroup mean differences (95% CIs) for allocation concealment were as follows: (**Low vs High**) -6.63U/L (-12.6, -0.665U/L); (**Unclear vs Low**) -7.32U/L (-12.7, -1.94U/L); (Unclear vs Low) -0.682U/L (-3.88, 2.51U/L).

ALT=alanine aminotransferase; CI=confidence interval; N/A=undeterminable; N=number.

Supplementary Figure S56 (part 1 of 3): A priori subgroup analyses for the effect of SSB on ALT (U/L) in addition trials

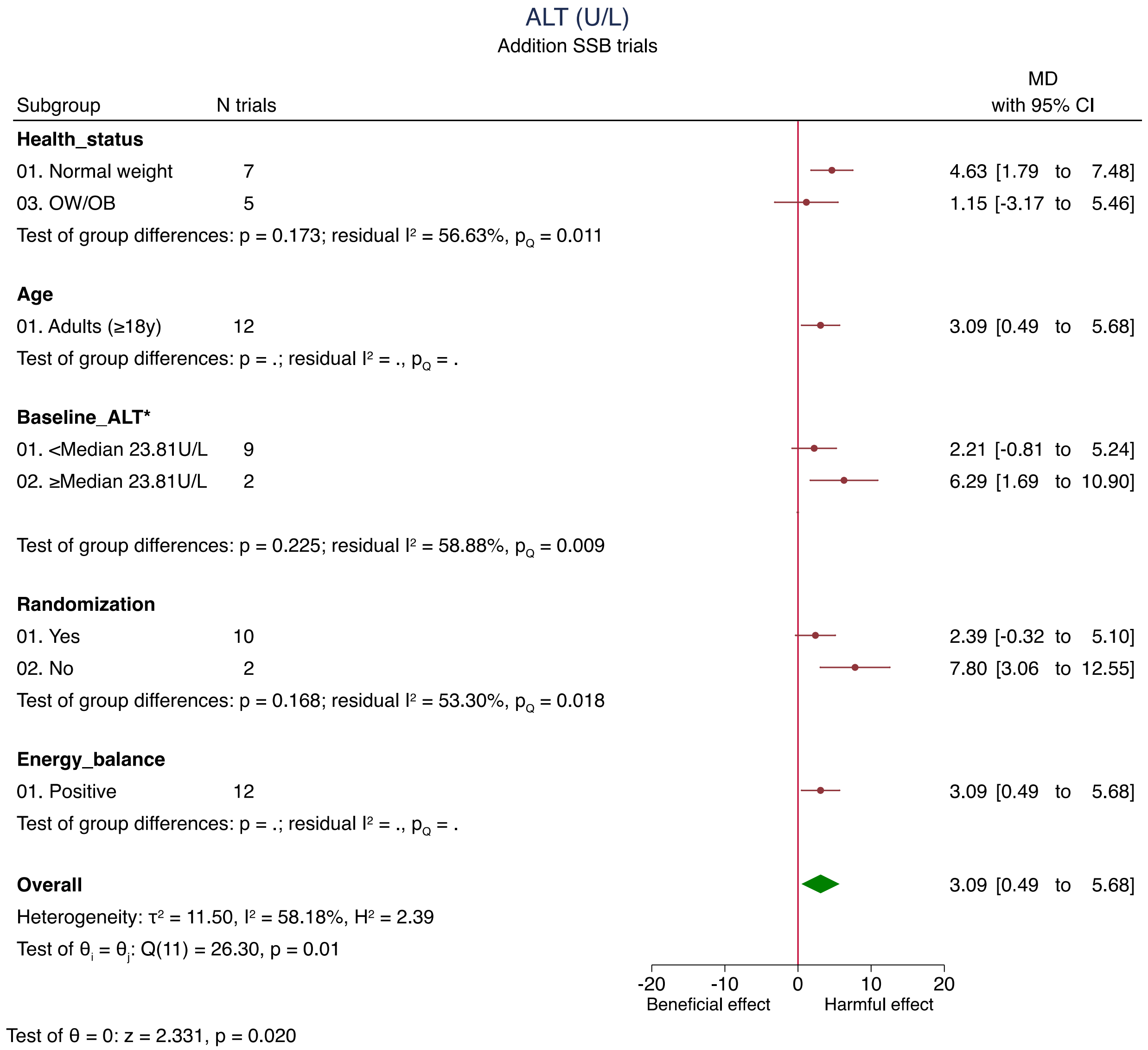

The green diamond represents the pooled estimate for the overall primary analysis of food sources of fructose-containing sugars and ALT. Within subgroup mean differences are the pooled effect estimates represented by a red circle. 95% confidence intervals are represented by the line through the circle. Data are expressed as mean differences with 95% confidence intervals using the generic inverse-variance method and random effects DerSimonian-Laird model. Inter-study heterogeneity was assessed using the Cochrane Q statistic and quantified using the I^2 statistic, with significance set at $p<0.100$ and $I^2 \geq 50\%$ considered to be evidence of substantial heterogeneity. $p<0.050$ indicates that the effect size differed between levels of the subgroup.
*N=1 trial missing data for baseline ALT.
ALT=alanine aminotransferase; CI=confidence interval; MD=mean difference; N=number; NAFLD=non-alcoholic fatty liver disease; OW/OB=overweight or obese; y=years.

Supplementary Figure S56 (part 2 of 3): A priori subgroup analyses for the effect of SSB on ALT (U/L) in addition trials

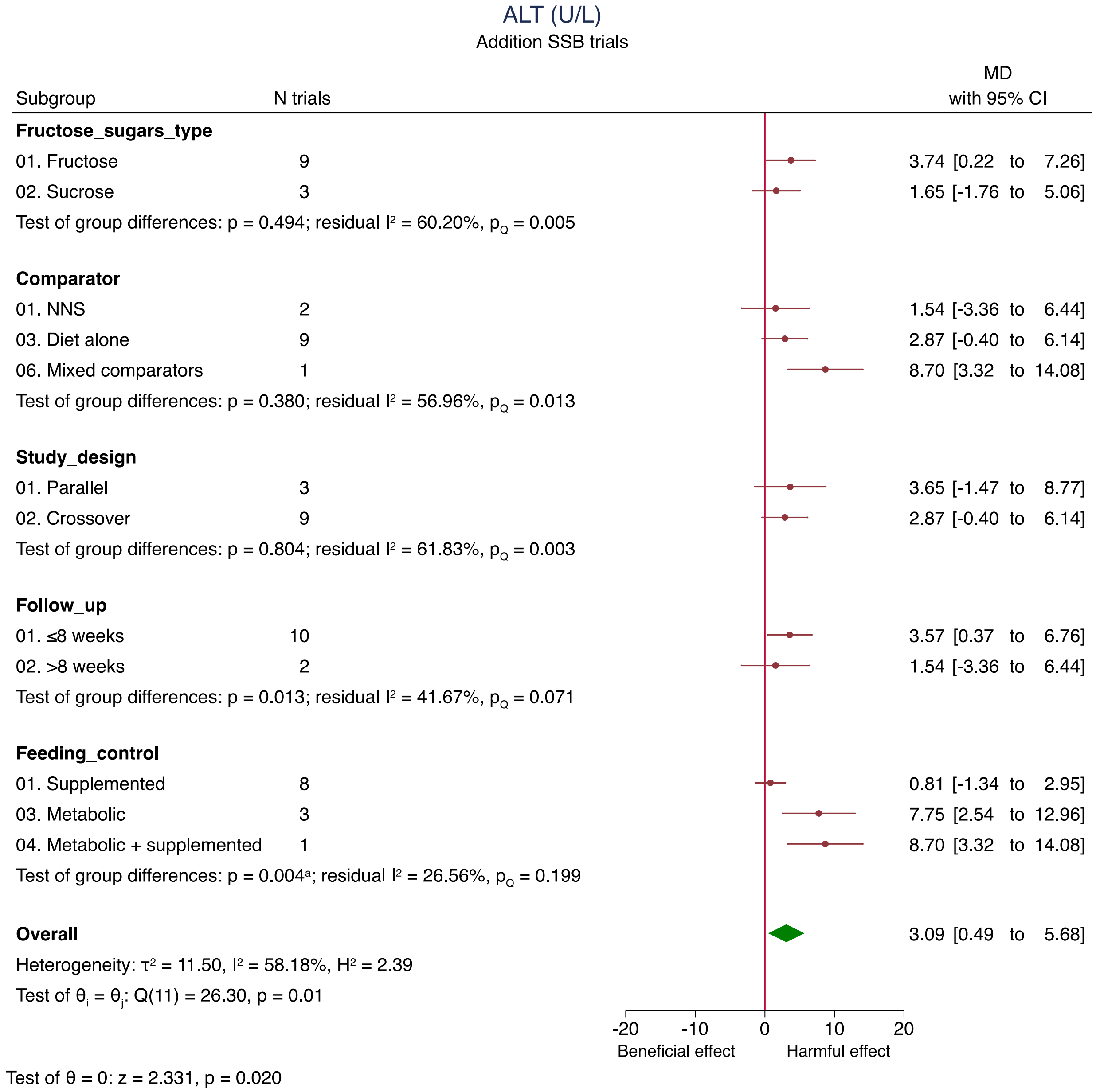

The green diamond represents the pooled estimate for the overall primary analysis of food sources of fructose-containing sugars and ALT. Within subgroup mean differences are the pooled effect estimates represented by a red circle. 95% confidence intervals are represented by the line through the circle. Data are expressed as mean differences with 95% confidence intervals using the generic inverse-variance method and random effects DerSimonian-Laird model. Inter-study heterogeneity was assessed using the Cochrane Q statistic and quantified using the I^2 statistic, with significance set at p<0.100 and $I^2 \geq 50\%$ considered to be evidence of substantial heterogeneity. p<0.050 indicates that the effect size differed between levels of the subgroup.

[a] Pairwise between-subgroup mean differences (95% CIs) for feeding control were as follows: (**3 vs 1**) 6.67U/L (1.85, 11.5U/L); (**4 vs 1**) 7.92U/L (1.14, 14.7U/L); (4 vs 3) 1.25U/L (-6.4, 8.89U/L).

Supplementary Figure S56 (part 3 of 3): A priori subgroup analyses for the effect of SSB on ALT (U/L) in addition trials

ALT (U/L)

Addition SSB trials

Subgroup	N trials	MD with 95% CI
**Dose**		
01. ≤10%E	3	1.66 [-1.62 to 4.95]
02. >10%E	9	3.76 [0.19 to 7.33]
Test of group differences: p = 0.486; residual I^2 = 60.11%, p_Q = 0.005		
**Regulatory_designation**		
02. Added	12	3.09 [0.49 to 5.68]
Test of group differences: p = .; residual I^2 = ., p_Q = .		
**Funding**		
01. Agency	7	1.90 [-1.46 to 5.25]
03. Agency + industry	5	5.00 [0.89 to 9.11]
Test of group differences: p = 0.254; residual I^2 = 58.43%, p_Q = 0.007		
**Type_of_mean_difference**		
01. Change from baseline	6	5.70 [1.57 to 9.82]
02. End differences	6	0.71 [-1.90 to 3.32]
Test of group differences: p = 0.055; residual I^2 = 53.33%, p_Q = 0.018		
**Sugar_form_matrix**		
02. Liquid	12	3.09 [0.49 to 5.68]
Test of group differences: p = .; residual I^2 = ., p_Q = .		
**Data_source**		
01. Reported	12	3.09 [0.49 to 5.68]
Test of group differences: p = .; residual I^2 = ., p_Q = .		
**Overall**		3.09 [0.49 to 5.68]
Heterogeneity: τ^2 = 11.50, I^2 = 58.18%, H^2 = 2.39		
Test of $\theta_i = \theta_j$: Q(11) = 26.30, p = 0.01		

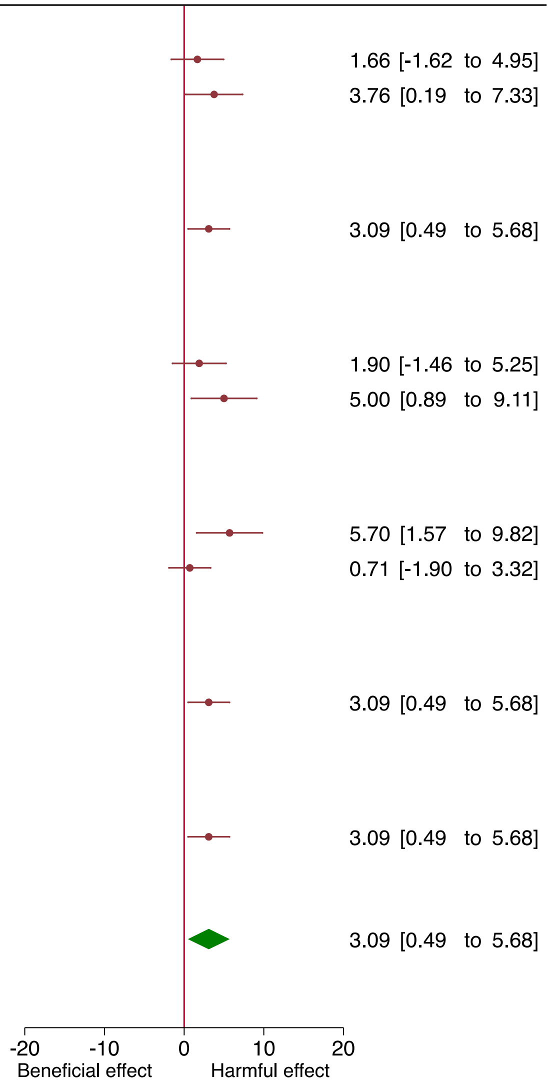

Test of θ = 0: z = 2.331, p = 0.020

The green diamond represents the pooled estimate for the overall primary analysis of food sources of fructose-containing sugars and ALT. Within subgroup mean differences are the pooled effect estimates represented by a red circle. 95% confidence intervals are represented by the line through the circle. Data are expressed as mean differences with 95% confidence intervals using the generic inverse-variance method and random effects DerSimonian-Laird model. Inter-study heterogeneity was assessed using the Cochrane Q statistic and quantified using the I^2 statistic, with significance set at p<0.100 and $I^2 \geq 50\%$ considered to be evidence of substantial heterogeneity. p<0.050 indicates that the effect size differed between levels of the subgroup. ALT=alanine aminotransferase; CI=confidence interval; %E=percentage of total energy intake; MD=mean difference; N=number; NAFLD=non-alcoholic fatty liver disease.

Supplementary Figure S57: Risk of bias (using The Cochrane Collaboration Tool) subgroup analysis for the effect of SSB on ALT (U/L) in addition trials

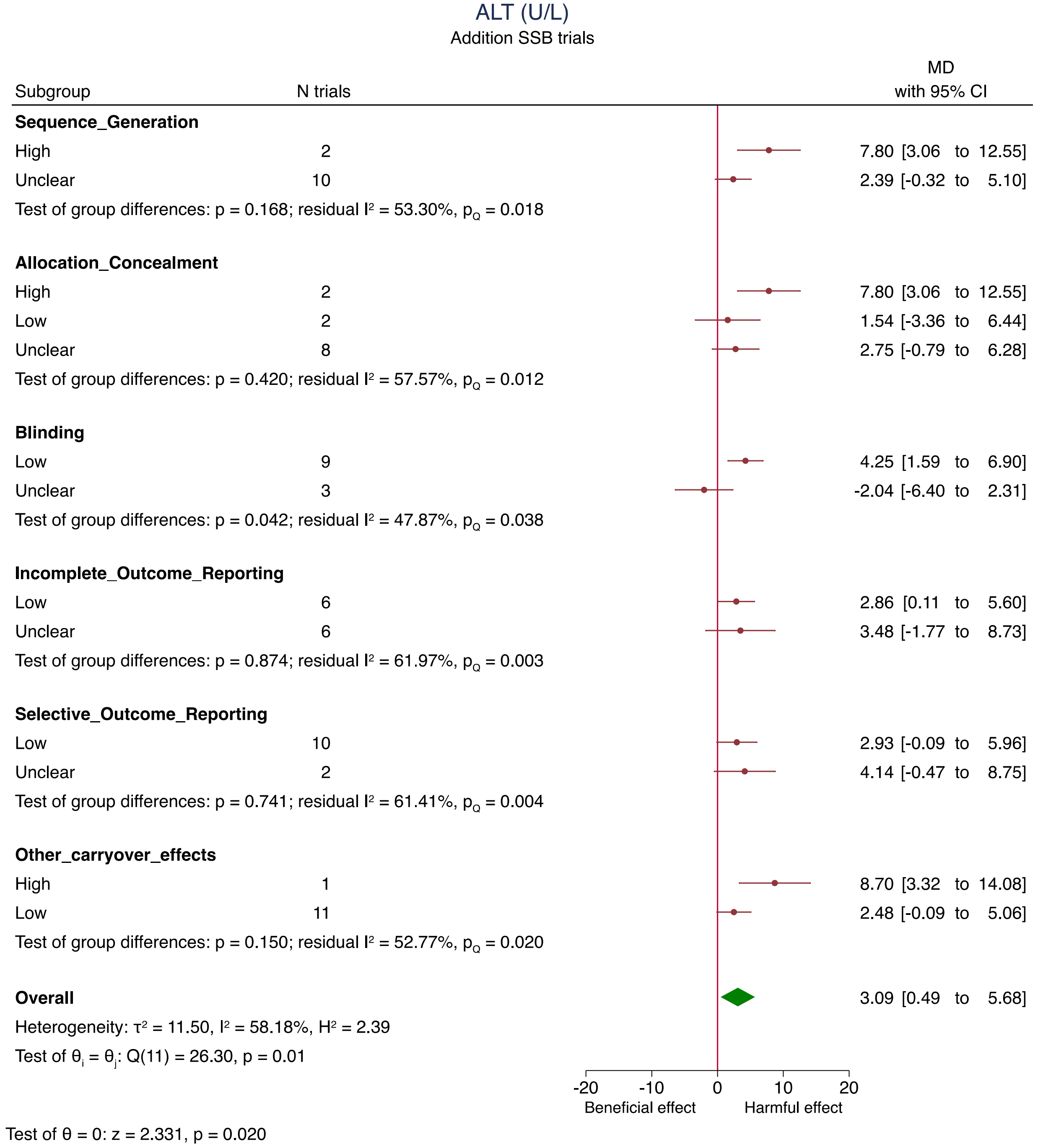

The green diamond represents the pooled estimate for the overall primary analysis of food sources of fructose-containing sugars and ALT. Within subgroup mean differences are the pooled effect estimates represented by a red circle. 95% confidence intervals are represented by the line through the circle. Data are expressed as mean differences with 95% confidence intervals using the generic inverse-variance method and random effects DerSimonian-Laird model. Inter-study heterogeneity was assessed using the Cochrane Q statistic and quantified using the I^2 statistic, with significance set at $p<0.100$ and $I^2 \geq 50\%$ considered to be evidence of substantial heterogeneity. $p<0.050$ indicates that the effect size differed between levels of the subgroup.
ALT=alanine aminotransferase; CI=confidence interval; N/A=undeterminable; N=number.

Supplementary Figure S58 (part 1 of 3): A priori subgroup analyses for the effect of important food sources of fructose-containing sugars and AST (U/L) in substitution trials

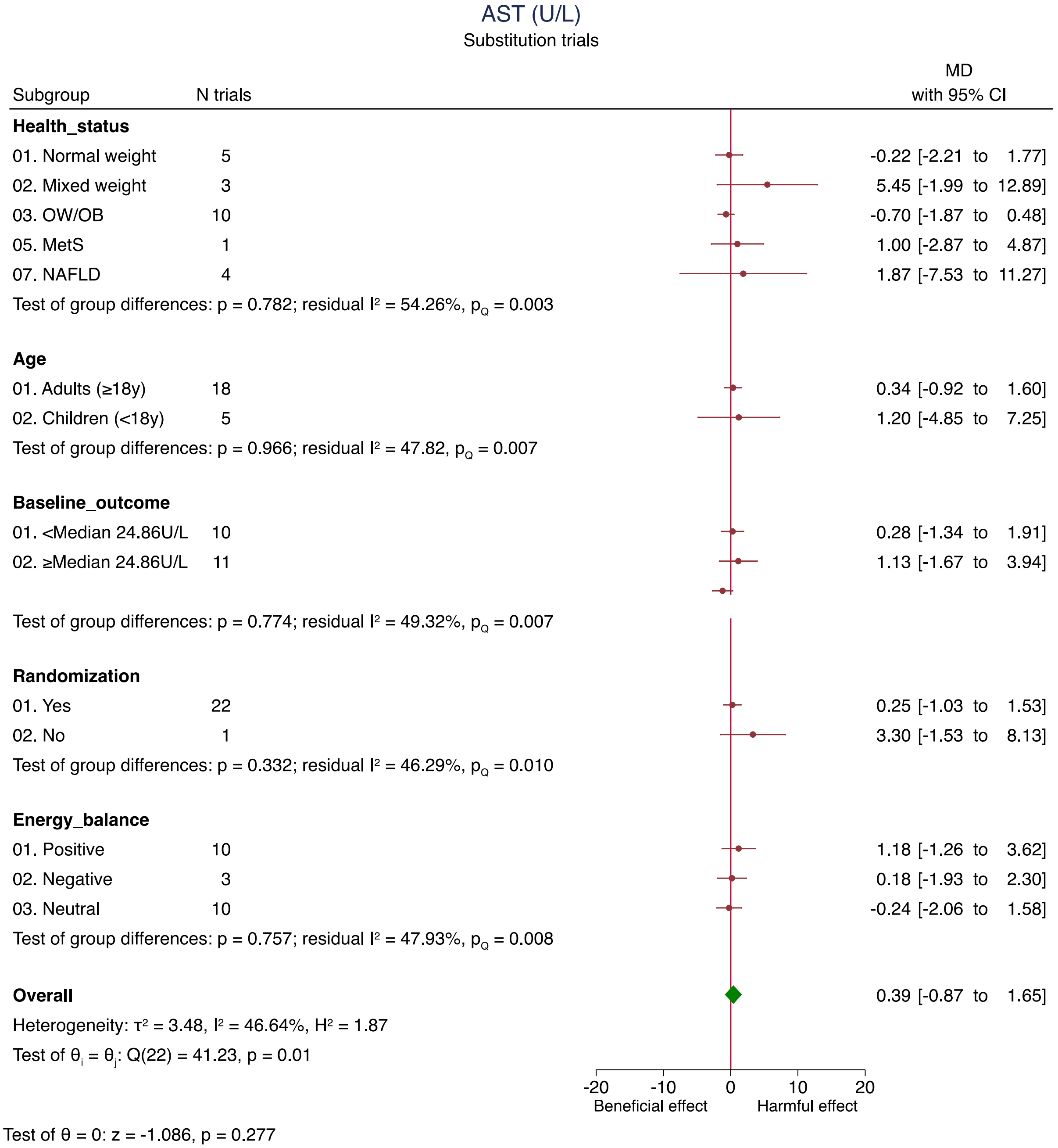

The green diamond represents the pooled estimate for the overall primary analysis of food sources of fructose-containing sugars and AST. Within subgroup mean differences are the pooled effect estimates represented by a red circle. 95% confidence intervals are represented by the line through the circle. Data are expressed as mean differences with 95% confidence intervals using the generic inverse-variance method and random effects DerSimonian-Laird model. Inter-study heterogeneity was assessed using the Cochrane Q statistic and quantified using the I^2 statistic, with significance set at $p<0.100$ and $I^2 \geq 50\%$ considered to be evidence of substantial heterogeneity. $p<0.050$ indicates that the effect size differed between levels of the subgroup.
*N=2 trials missing data for baseline AST.
ALT=alanine aminotransferase; AST=aspartate aminotransferase; CI=confidence interval; MD=mean difference; MetS=metabolic syndrome; N=number; NAFLD=non-alcoholic fatty liver disease; OW/OB=overweight or obese BMI; y=years.

Supplementary Figure S58 (part 2 of 3): A priori subgroup analyses for the effect of important food sources of fructose-containing sugars and AST (U/L) in substitution trials

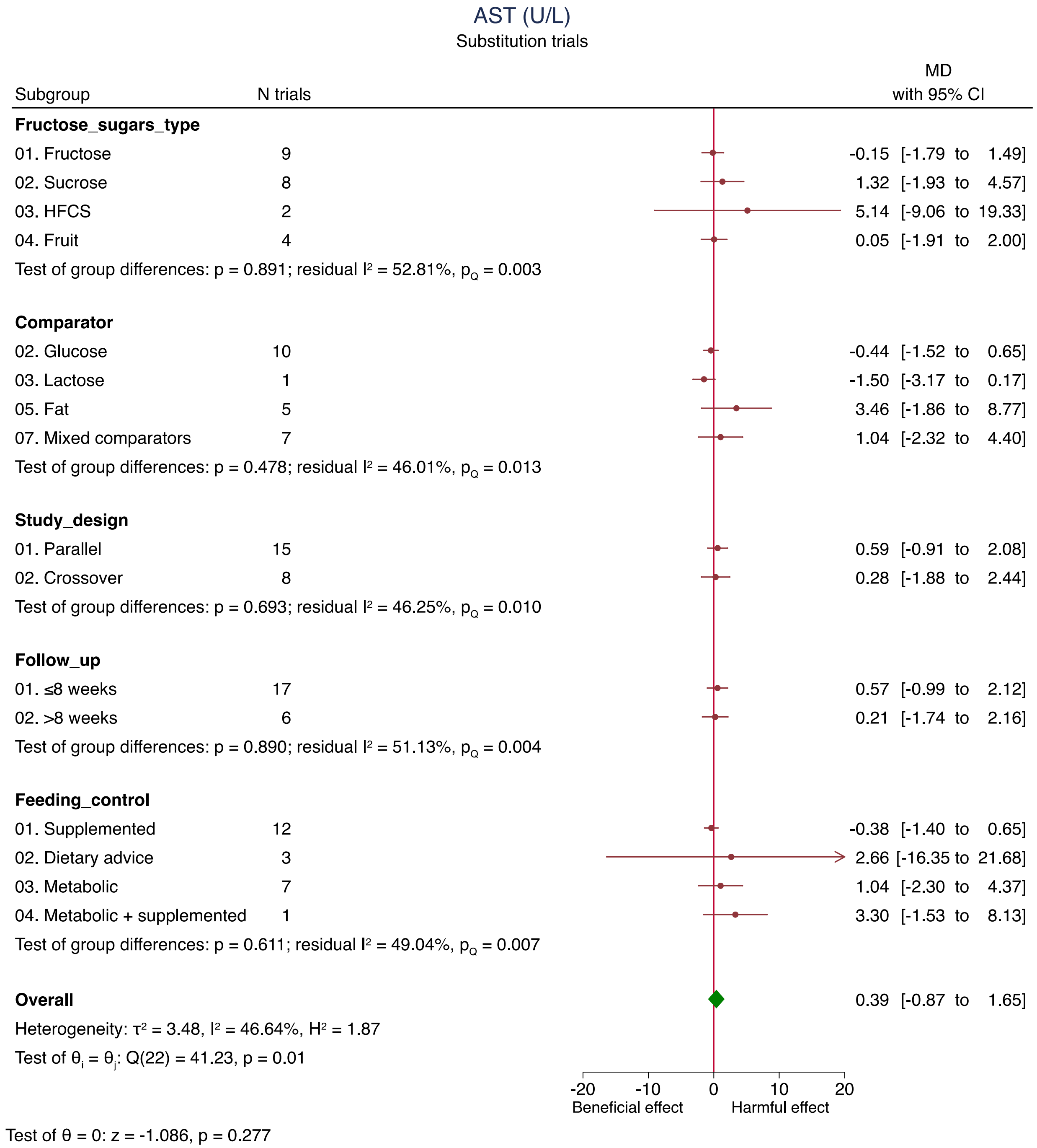

The green diamond represents the pooled estimate for the overall primary analysis of food sources of fructose-containing sugars and AST. Within subgroup mean differences are the pooled effect estimates represented by a red circle. 95% confidence intervals are represented by the line through the circle. Data are expressed as mean differences with 95% confidence intervals using the generic inverse-variance method and random effects DerSimonian-Laird model. Inter-study heterogeneity was assessed using the Cochrane Q statistic and quantified using the I^2 statistic, with significance set at p<0.100 and $I^2 \geq 50\%$ considered to be evidence of substantial heterogeneity. p<0.050 indicates that the effect size differed between levels of the subgroup.
AST=aspartate aminotransferase; CI=confidence interval; HFCS=high-fructose corn syrup; MD=mean difference; N=number.

Supplementary Figure S58 (part 3 of 3): A priori subgroup analyses for the effect of important food sources of fructose-containing sugars and AST (U/L) in substitution trials

AST (U/L)

Substitution trials

Subgroup	N trials	MD with 95% CI
**Dose**		
01. ≤10%E	5	-0.05 [-1.93 to 1.83]
02. >10%E	18	0.62 [-0.97 to 2.22]
Test of group differences: p = 0.769; residual I^2 = 49.01%, p_Q = 0.005		
**Regulatory_designation**		
01. Naturally occurring	4	0.05 [-1.91 to 2.00]
02. Added	13	-0.53 [-1.39 to 0.34]
03. Mixed designations	6	5.11 [-4.34 to 14.55]
Test of group differences: p = 0.194; residual I^2 = 45.10%, p_Q = 0.014		
**Funding**		
01. Agency	12	0.66 [-1.41 to 2.74]
03. Agency + industry	9	-0.59 [-1.70 to 0.52]
04. Not Reported	2	10.88 [-13.18 to 34.94]
Test of group differences: p = 0.530; residual I^2 = 49.83%, p_Q = 0.005		
**Type_of_mean_difference**		
01. Change from baseline	18	1.03 [-0.72 to 2.79]
02. End differences	5	-1.02 [-2.32 to 0.28]
Test of group differences: p = 0.283; residual I^2 = 46.35%, p_Q = 0.009		
**Sugar_form_matrix**		
01. Solid	4	-0.34 [-1.63 to 0.94]
02. Liquid	13	-0.49 [-1.50 to 0.52]
03. Mixed forms	6	5.11 [-4.34 to 14.55]
Test of group differences: p = 0.202; residual I^2 = 45.47%, p_Q = 0.013		
**Data_source**		
01. Reported	18	-0.57 [-1.38 to 0.24]
02. Plot digitized	1	23.78 [12.92 to 34.64]
05. Provided by author	4	2.71 [-2.42 to 7.85]
Test of group differences: p < 0.001[a]; residual I^2 = 0.00%, p_Q = 0.498		
**Overall**		0.39 [-0.87 to 1.65]
Heterogeneity: τ^2 = 3.48, I^2 = 46.64%, H^2 = 1.87		
Test of $\theta_i = \theta_j$: Q(22) = 41.23, p = 0.01		

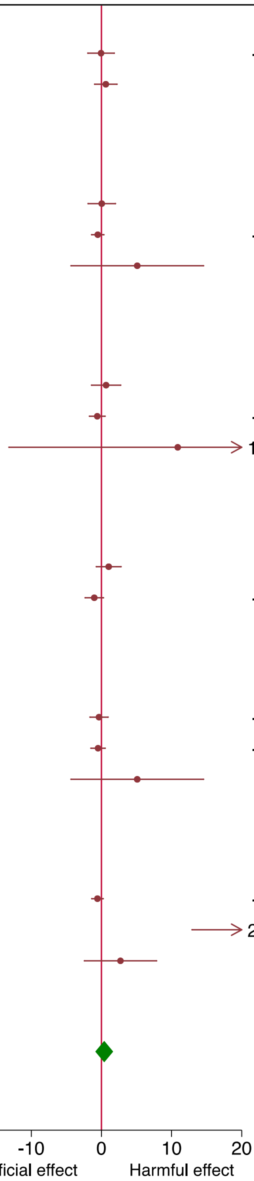

Test of θ = 0: z = -1.086, p = 0.277

The green diamond represents the pooled estimate for the overall primary analysis of food sources of fructose-containing sugars and AST. Within subgroup mean differences are the pooled effect estimates represented by a red circle. 95% confidence intervals are represented by the line through the circle. Data are expressed as mean differences with 95% confidence intervals using the generic inverse-variance method and random effects DerSimonian-Laird model. Inter-study heterogeneity was assessed using the Cochrane Q statistic and quantified using the I^2 statistic, with significance set at p<0.100 and I^2≥50% considered to be evidence of substantial heterogeneity. p<0.050 indicates that the effect size differed between levels of the subgroup.

[a] Pairwise between-subgroup mean differences (95% CIs) for data source were as follows: (**2 vs 1**) 24.4U/L (13.5, 35.2U/L); (5 vs 1) 2.57U/L (-0.359, 5.5U/L); (**5 vs 2**) -21.8U/L (-33, -10.6U/L).

AST=aspartate aminotransferase; CI=confidence interval; %E=percentage of total energy; MD=mean difference; N=number.

Supplementary Figure S59: Risk of bias (using The Cochrane Collaboration Tool) subgroup analysis for the effect of important food sources of fructose-containing sugars and AST (U/L) in substitution trials

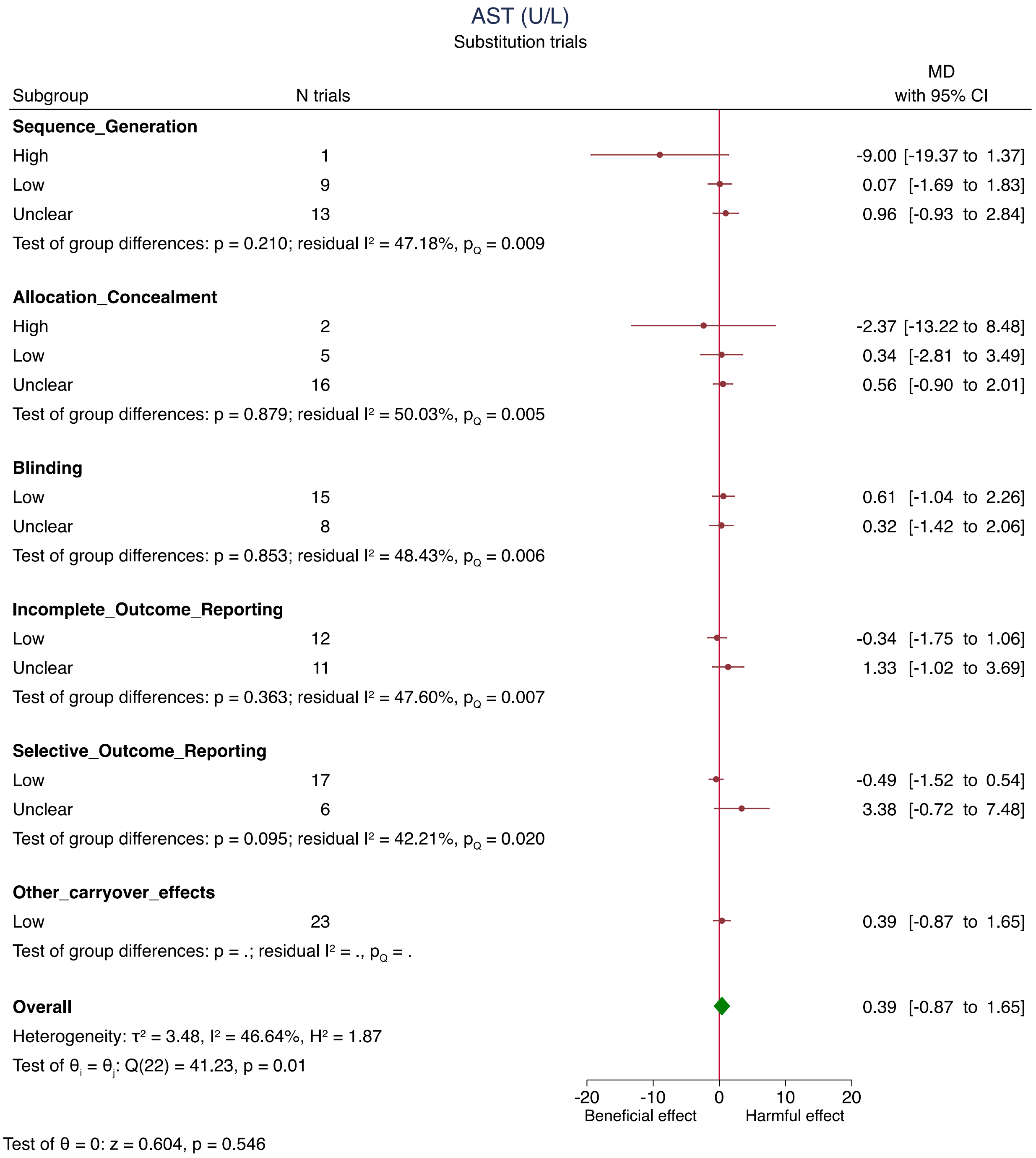

The green diamond represents the pooled estimate for the overall primary analysis of food sources of fructose-containing sugars and AST. Within subgroup mean differences are the pooled effect estimates represented by a red circle. 95% confidence intervals are represented by the line through the circle. Data are expressed as mean differences with 95% confidence intervals using the generic inverse-variance method and random effects DerSimonian-Laird model. Inter-study heterogeneity was assessed using the Cochrane Q statistic and quantified using the I^2 statistic, with significance set at $p<0.100$ and $I^2 \geq 50\%$ considered to be evidence of substantial heterogeneity. $p<0.050$ indicates that the effect size differed between levels of the subgroup. AST=aspartate aminotransferase; CI=confidence interval; MD=mean difference; N=number.

Supplementary Figure S60 (part 1 of 3): A priori subgroup analyses for the effect of important food sources of fructose-containing sugars and AST (U/L) in addition trials

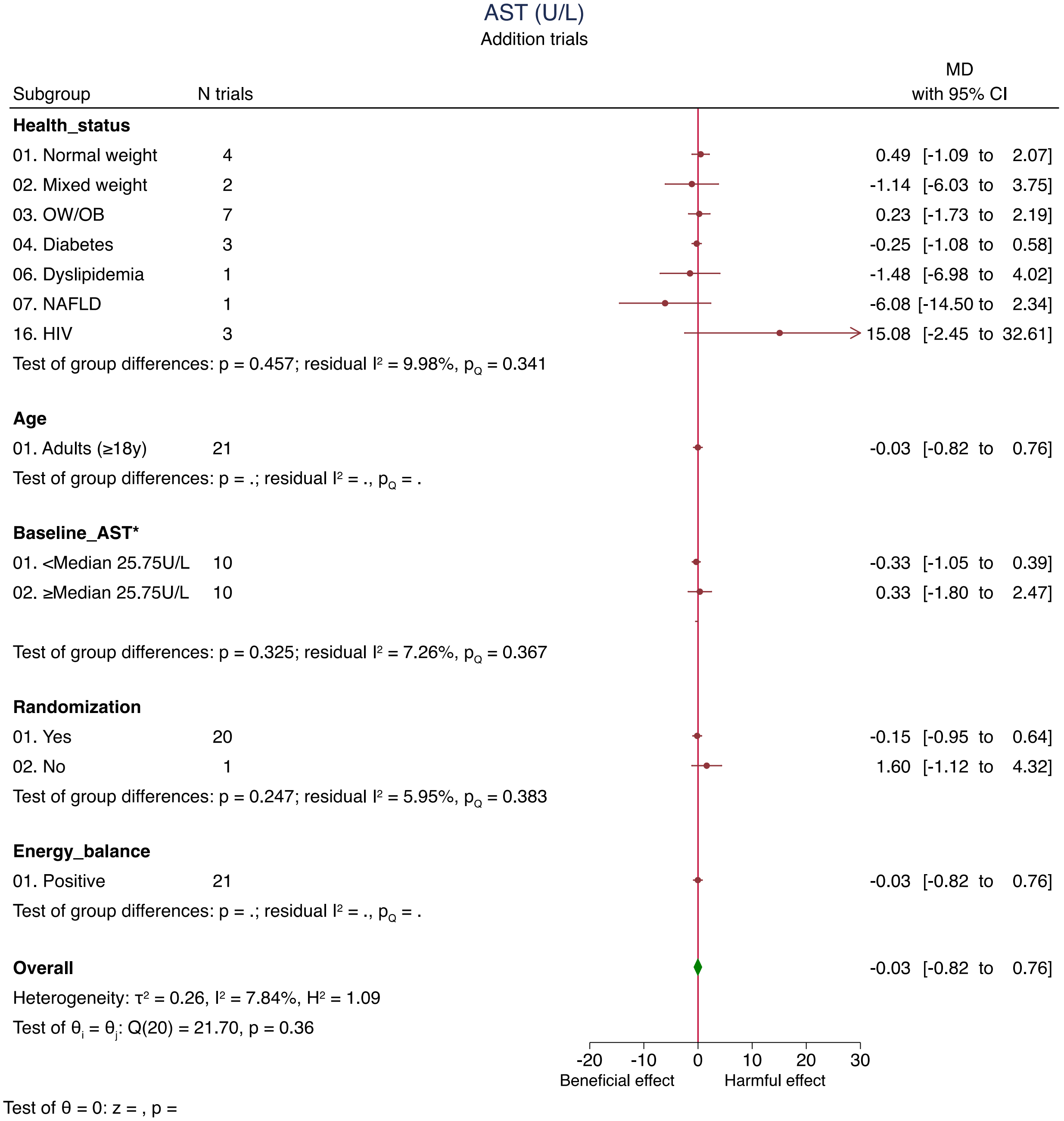

The green diamond represents the pooled estimate for the overall primary analysis of food sources of fructose-containing sugars and AST. Within subgroup mean differences are the pooled effect estimates represented by a red circle. 95% confidence intervals are represented by the line through the circle. Data are expressed as mean differences with 95% confidence intervals using the generic inverse-variance method and random effects DerSimonian-Laird model. Inter-study heterogeneity was assessed using the Cochrane Q statistic and quantified using the I^2 statistic, with significance set at p<0.100 and I^2≥50% considered to be evidence of substantial heterogeneity. p<0.050 indicates that the effect size differed between levels of the subgroup.
*N=1 trial missing data for baseline AST.
AST=aspartate aminotransferase; CI=confidence interval; HIV=human immunodeficiency virus; MD=mean difference; N=number; NAFLD=non-alcoholic fatty liver disease; OW/OB=overweight or obese; y=years; y=years.

Supplementary Figure S60 (part 2 of 3): A priori subgroup analyses for the effect of important food sources of fructose-containing sugars and AST (U/L) in addition trials

AST (U/L)

Addition trials

Subgroup	N trials	MD with 95% CI
**Fructose_sugars_type**		
01. Fructose	6	-0.05 [-1.40 to 1.31]
02. Sucrose	4	0.45 [-3.10 to 4.00]
04. Fruit	7	-0.84 [-2.88 to 1.19]
05. Honey	4	1.05 [-4.04 to 6.15]
Test of group differences: p = 0.731; residual I^2 = 14.22%, p_Q = 0.284		
**Comparator**		
01. NNS	3	0.98 [-2.12 to 4.07]
02. Water	2	-2.40 [-7.48 to 2.68]
03. Diet alone	14	-0.23 [-0.92 to 0.47]
06. Mixed comparators	2	-1.16 [-8.39 to 6.06]
Test of group differences: p = 0.478; residual I^2 = 9.77%, p_Q = 0.338		
**Study_design**		
01. Parallel	14	0.26 [-0.98 to 1.50]
02. Crossover	7	-0.36 [-1.68 to 0.96]
Test of group differences: p = 0.435; residual I^2 = 11.39%, p_Q = 0.313		
**Follow_up**		
01. ≤8 weeks	14	-0.17 [-0.82 to 0.48]
02. >8 weeks	7	0.68 [-3.40 to 4.75]
Test of group differences: p = 0.129; residual I^2 = 0.00%, p_Q = 0.482		
**Feeding_control**		
01. Supplemented	19	-0.33 [-1.15 to 0.50]
03. Metabolic	1	1.00 [-0.96 to 2.96]
04. Metabolic + supplemented	1	1.60 [-1.12 to 4.32]
Test of group differences: p = 0.261; residual I^2 = 3.87%, p_Q = 0.409		
**Overall**		-0.03 [-0.82 to 0.76]
Heterogeneity: τ^2 = 0.26, I^2 = 7.84%, H^2 = 1.09		
Test of $\theta_i = \theta_j$: Q(20) = 21.70, p = 0.36		

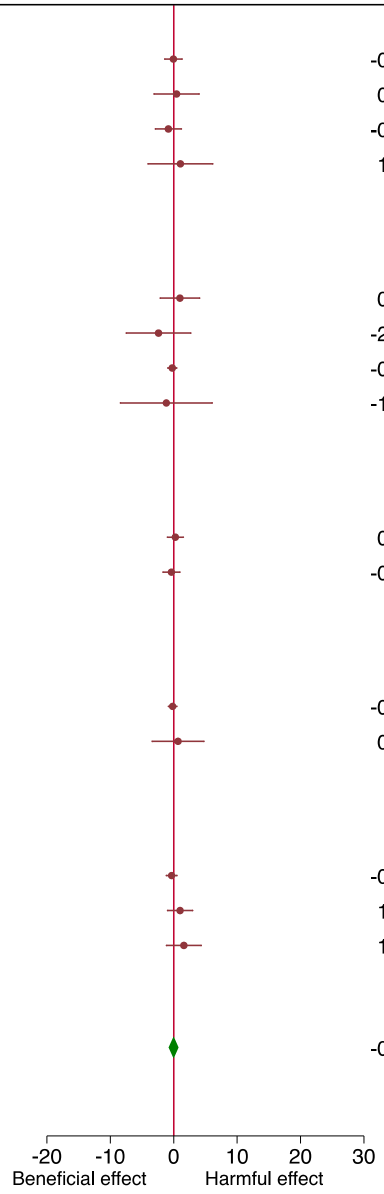

Test of θ = 0: z = , p =

The green diamond represents the pooled estimate for the overall primary analysis of food sources of fructose-containing sugars and AST. Within subgroup mean differences are the pooled effect estimates represented by a red circle. 95% confidence intervals are represented by the line through the circle. Data are expressed as mean differences with 95% confidence intervals using the generic inverse-variance method and random effects DerSimonian-Laird model. Inter-study heterogeneity was assessed using the Cochrane Q statistic and quantified using the I^2 statistic, with significance set at p<0.100 and $I^2 \geq 50\%$ considered to be evidence of substantial heterogeneity. p<0.050 indicates that the effect size differed between levels of the subgroup. AST=aspartate aminotransferase; CI=confidence interval; MD=mean difference; NNS=non-nutritive sweetener; N=number.

Supplementary Figure S60 (part 3 of 3): A priori subgroup analyses for the effect of important food sources of fructose-containing sugars and AST (U/L) in addition trials

AST (U/L)

Addition trials

Subgroup	N trials	MD with 95% CI
**Dose**		
01. ≤10%E	13	-0.01 [-1.94 to 1.92]
02. >10%E	8	-0.11 [-0.80 to 0.57]
Test of group differences: p = 0.772; residual I² = 11.78%, p_Q = 0.308		
**Regulatory_designation**		
01. Naturally occurring	7	-0.84 [-2.88 to 1.19]
02. Added	14	0.13 [-0.93 to 1.20]
Test of group differences: p = 0.403; residual I² = 9.80%, p_Q = 0.333		
**Funding**		
01. Agency	14	0.09 [-0.98 to 1.17]
02. Industry	1	-0.20 [-3.55 to 3.15]
03. Agency + industry	6	-0.73 [-3.04 to 1.57]
Test of group differences: p = 0.817; residual I² = 15.60%, p_Q = 0.263		
**Type_of_mean_difference**		
01. Change from baseline	12	0.23 [-1.00 to 1.46]
02. End differences	8	-0.43 [-1.75 to 0.89]
05. Provided by author	1	4.20 [-3.55 to 11.95]
Test of group differences: p = 0.400; residual I² = 10.84%, p_Q = 0.322		
**Sugar_form_matrix**		
01. Solid	5	-2.51 [-5.43 to 0.42]
02. Liquid	16	0.17 [-0.68 to 1.03]
Test of group differences: p = 0.093; residual I² = 0.00%, p_Q = 0.465		
**Data_source**		
01. Reported	21	-0.03 [-0.82 to 0.76]
Test of group differences: p = .; residual I² = ., p_Q = .		
**Overall**		-0.03 [-0.82 to 0.76]
Heterogeneity: τ² = 0.26, I² = 7.84%, H² = 1.09		
Test of $θ_i = θ_j$: Q(20) = 21.70, p = 0.36		

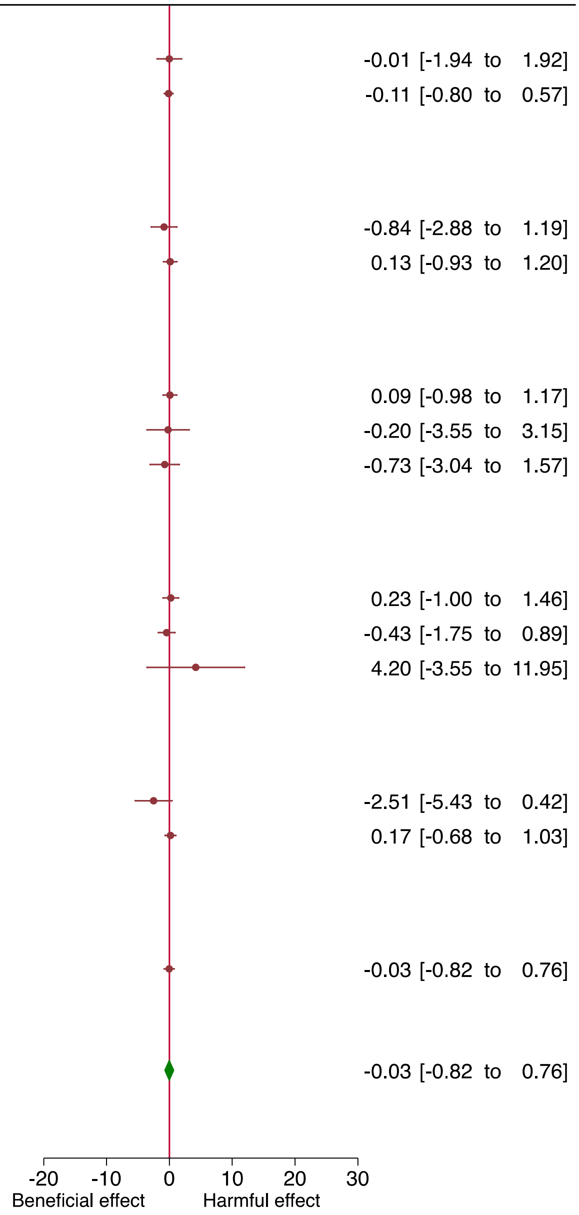

Test of θ = 0: z = , p =

The green diamond represents the pooled estimate for the overall primary analysis of food sources of fructose-containing sugars and AST. Within subgroup mean differences are the pooled effect estimates represented by a red circle. 95% confidence intervals are represented by the line through the circle. Data are expressed as mean differences with 95% confidence intervals using the generic inverse-variance method and random effects DerSimonian-Laird model. Inter-study heterogeneity was assessed using the Cochrane Q statistic and quantified using the I^2 statistic, with significance set at p<0.100 and I^2≥50% considered to be evidence of substantial heterogeneity. p<0.050 indicates that the effect size differed between levels of the subgroup. AST=aspartate aminotransferase; CI=confidence interval; %E=percentage of total energy intake; MD=mean difference; N=number.

Supplementary Figure S61: Risk of bias (using The Cochrane Collaboration Tool) subgroup analysis for the effect of important food sources of fructose-containing sugars and AST (U/L) in addition trials

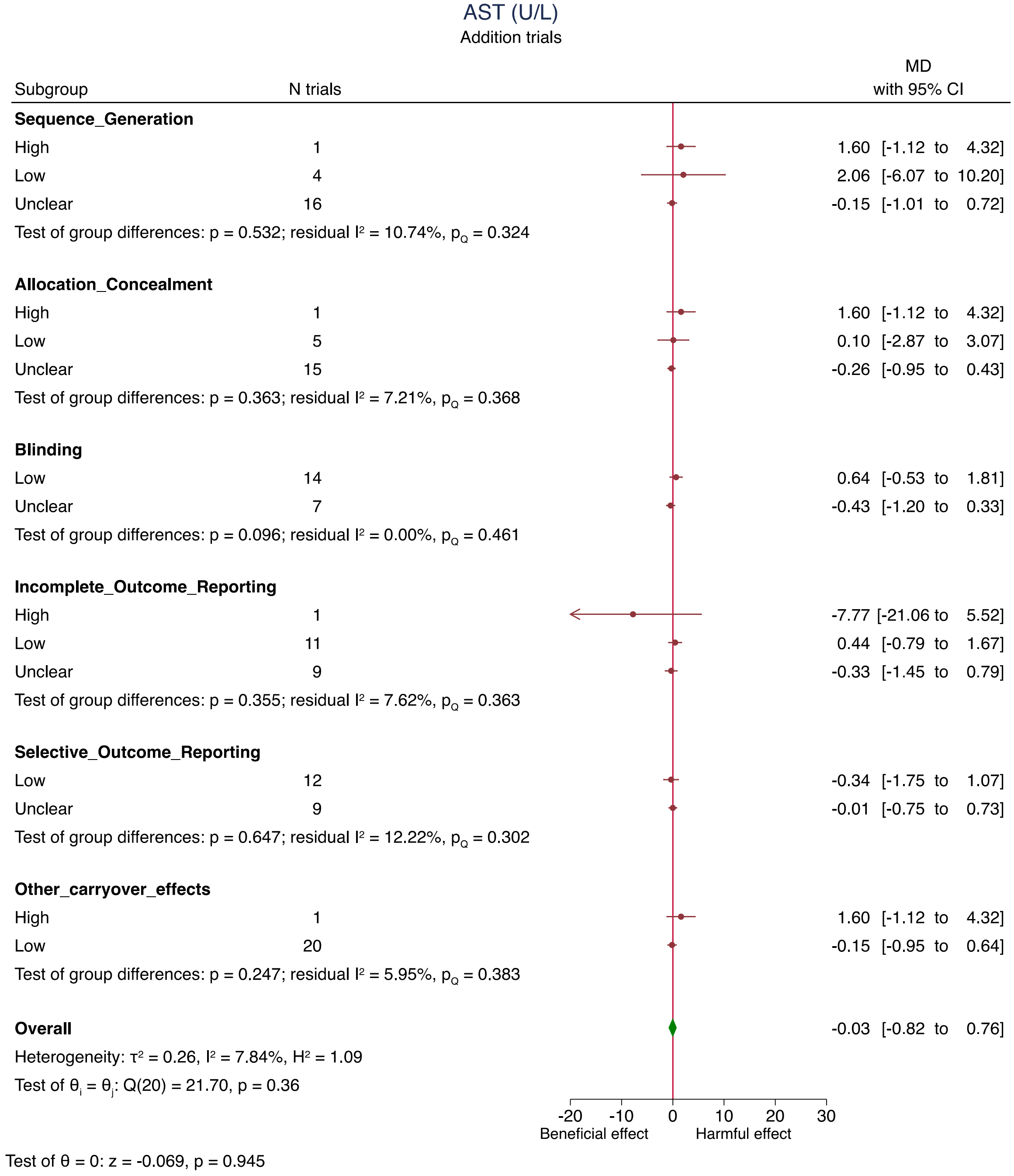

The green diamond represents the pooled estimate for the overall primary analysis of food sources of fructose-containing sugars and AST. Within subgroup mean differences are the pooled effect estimates represented by a red circle. 95% confidence intervals are represented by the line through the circle. Data are expressed as mean differences with 95% confidence intervals using the generic inverse-variance method and random effects DerSimonian-Laird model. Inter-study heterogeneity was assessed using the Cochrane Q statistic and quantified using the I^2 statistic, with significance set at p<0.100 and $I^2 \geq 50\%$ considered to be evidence of substantial heterogeneity. p<0.050 indicates that the effect size differed between levels of the subgroup. AST=aspartate aminotransferase; CI=confidence interval; MD=mean difference; N=number.

Supplementary Figure S62: Continuous meta-regression analysis for the effect of important food sources of fructose-containing sugars and IHCL (SMD) in substitution trials

Continuous meta-regression

IHCL (SMD) in substitution trials

Subgroup	Range	Trials	Beta [95% CI]	P_{SMD}	Residual I^2 (%)	P_Q
Baseline IHCL (SMD)[a]	2.6—16.5	14	-0.02 [-0.16 to 0.12]	0.793	17	0.271
Follow-up (weeks)	1.0—24.0	16	0.10 [0.01 to 0.19]	0.027	0	0.712
Food source of fructose containing-sugars dose (%E)	3.0—35.0	16	-0.03 [-0.07 to 0.01]	0.140	0	0.496
Mean age (years)	13.0—59.0	16	0.02 [-0.01 to 0.05]	0.281	3	0.421

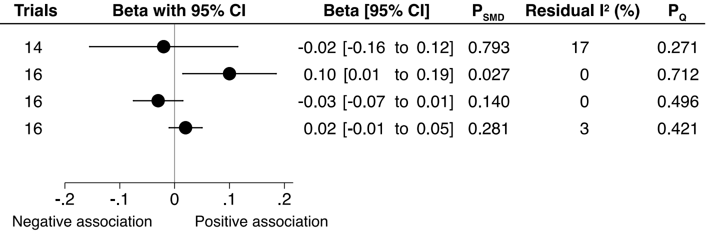

Data is presented as between group mean difference (95% confidence intervals) for a 1-unit change in the predictor variable. ß–coefficients were estimated using continuous meta-regression analysis. A positive ß-coefficient implies an increase in IHCL with the food source of fructose-containing sugars intervention as the subgroup variable increases, and a negative ß-coefficient implies a decrease in uric acid. Residual I^2 reports inter-study heterogeneity not explained by the subgroup and was estimated using the Cochrane Q statistic.

[a] N=2 trials missing data for baseline IHCL.

CI=confidence interval; %E=percentage of total energy intake; IHCL=intrahepatocellular lipid; SMD=standardized mean difference.

Supplementary Figure S63: Continuous meta-regression analysis for the effect of important food sources of fructose-containing sugars and IHCL (SMD) in addition trials

Continuous meta-regression

IHCL (SMD) in addition trials

Subgroup	Range	Trials	Beta [95% CI]	P_{MD}	Residual I^2 (%)	P_Q
Baseline IHCL (SMD)[a]	1.6— 9.7	11	-0.13 [-0.39 to 0.13]	0.349	0	0.967
Follow-up (weeks)	1.0—24.0	13	-0.01 [-0.10 to 0.08]	0.875	0	0.911
Food source of fructose containing sugars dose (%E)	18.0—35.0	13	0.03 [-0.08 to 0.14]	0.612	0	0.924
Mean age (years)	21.7—39.0	13	0.03 [-0.08 to 0.13]	0.526	0	0.931

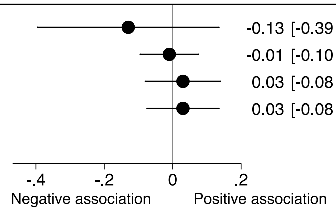

Data is presented as between group mean difference (95% confidence intervals) for a 1-unit change in the predictor variable. ß–coefficients were estimated using continuous meta-regression analysis. A positive ß-coefficient implies an increase in IHCL with the food source of fructose-containing sugars intervention as the subgroup variable increases, and a negative ß-coefficient implies a decrease in uric acid. Residual I^2 reports inter-study heterogeneity not explained by the subgroup and was estimated using the Cochrane Q statistic.

[a]N=2 trials missing data for baseline IHCL.

CI=confidence interval; %E=percentage of total energy intake; IHCL=intrahepatocellular lipid; SMD=standardized mean difference.

Supplementary Figure S64: Continuous meta-regression analysis for the effect of important food sources of fructose-containing sugars and ALT (U/L) in substitution trials

Continuous meta-regression

ALT (U/L) in substitution trials

Subgroup	Range	Trials	Beta [95% CI]	P_{MD}	Residual I^2 (%)	P_Q
Baseline ALT (U/L)[a]	4.8—116.6	26	-0.07 [-0.26 to 0.12]	0.502	54	0.001
Follow-up (weeks)	1.0—52.0	28	-0.10 [-0.26 to 0.06]	0.204	48	0.003
Food source of fructose containing sugars dose (%E)	1.0—42.0	28	0.10 [-0.00 to 0.21]	0.076	47	0.004
Mean age (years)	7.7—57.6	28	-0.02 [-0.12 to 0.08]	0.667	52	0.001

Beta with 95% CI

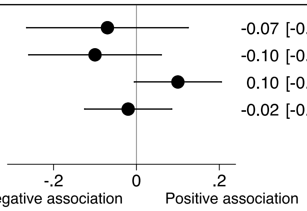

Data is presented as between group mean difference (95% confidence intervals) for a 1-unit change in the predictor variable. ß–coefficients were estimated using continuous meta-regression analysis. A positive ß-coefficient implies an increase in ALT (U/L) with the food source of fructose-containing sugars intervention as the subgroup variable increases, and a negative ß-coefficient implies a decrease in uric acid. Residual I^2 reports inter-study heterogeneity not explained by the subgroup and was estimated using the Cochrane Q statistic.

[a] N=2 trials missing data for baseline ALT.

ALT=alanine aminotransferase; CI=confidence interval; %E=percentage of total energy intake.

Supplementary Figure S65: Continuous meta-regression analysis for the effect of important food sources of fructose-containing sugars and ALT (U/L) in addition trials

Continuous meta-regression

ALT (U/L) in addition trials

Subgroup	Range	Trials	Beta [95% CI]	P_{MD}	Residual I^2 (%)	P_Q
Baseline ALT (U/L)[a]	16.4—46.9	24	-0.12 [-0.43 to 0.19]	0.422	42	0.017
Follow-up (weeks)	1.0—24.0	31	-0.23 [-0.67 to 0.21]	0.305	32	0.050
Food source of fructose containing sugars dose (%E)	1.2—35.0	31	0.15 [0.03 to 0.27]	0.011	23	0.133
Mean age (years)	23.9—58.9	31	-0.17 [-0.28 to -0.06]	0.004	18	0.192

Beta with 95% CI

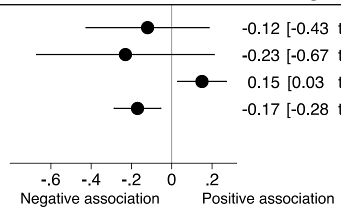

Data is presented as between group mean difference (95% confidence intervals) for a 1-unit change in the predictor variable. ß–coefficients were estimated using continuous meta-regression analysis. A positive ß-coefficient implies an increase in ALT (U/L) with the food source of fructose-containing sugars intervention as the subgroup variable increases, and a negative ß-coefficient implies a decrease in uric acid. Residual I^2 reports inter-study heterogeneity not explained by the subgroup and was estimated using the Cochrane Q statistic.

*N=7 trials missing data for baseline ALT.

ALT=alanine aminotransferase; CI=confidence interval; %E=percentage of total energy intake.

Supplementary Figure S66: Continuous meta-regression analysis for the effect of SSB on ALT (U/L) in addition trials

Continuous meta-regression
ALT (U/L) in addition SSB trials

Subgroup	Range	Trials	Beta with 95% CI	Beta [95% CI]	P_{MD}	Residual I^2 (%)	P_Q
Baseline ALT (U/L)[a]	16.4—28.9	11		0.01 [-0.76 to 0.78]	0.983	64	0.003
Follow-up (weeks)	1.0— 8.6	12		-0.31 [-1.24 to 0.62]	0.512	60	0.005
Food source of fructose containing sugars dose (%E)	5.0—35.0	12		0.19 [-0.01 to 0.39]	0.082	52	0.021
Mean age (years)	23.9—43.0	12		-0.30 [-0.61 to 0.01]	0.055	51	0.026

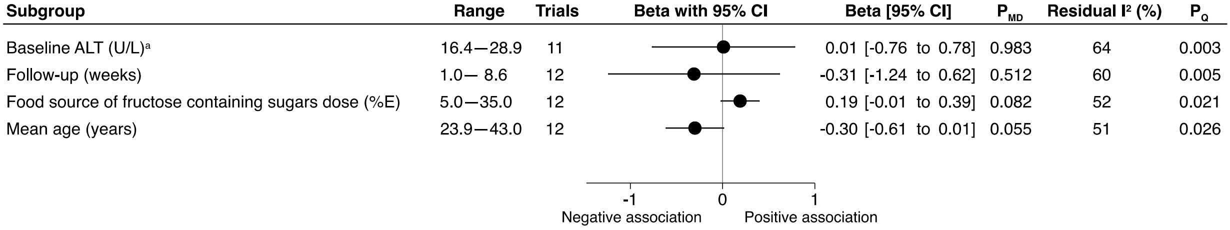

Data is presented as between group mean difference (95% confidence intervals) for a 1-unit change in the predictor variable. ß–coefficients were estimated using continuous meta-regression analysis. A positive ß-coefficient implies an increase in ALT (U/L) with the food source of fructose-containing sugars intervention as the subgroup variable increases, and a negative ß-coefficient implies a decrease in uric acid. Residual I^2 reports inter-study heterogeneity not explained by the subgroup and was estimated using the Cochrane Q statistic.
*N=1 trial missing data for baseline ALT.
ALT=alanine aminotransferase; CI=confidence interval; %E=percentage of total energy intake.

Supplementary Figure S67: Continuous meta-regression analysis for the effect of important food sources of fructose-containing sugars and AST (U/L) in substitution trials

Continuous meta-regression

AST (U/L) in substitution trials

Subgroup	Range	Trials	Beta [95% CI]	P_{MD}	Residual I^2 (%)	P_Q
Baseline AST (U/L)[a]	5.0—68.8	21	-0.10 [-0.34 to 0.13]	0.387	50	0.006
Follow-up (weeks)	1.0—52.0	23	-0.08 [-0.24 to 0.09]	0.360	49	0.005
Food source of fructose containing sugars dose (%E)	1.0—42.0	23	0.01 [-0.10 to 0.12]	0.806	49	0.005
Mean age (years)	7.7—57.6	23	0.03 [-0.06 to 0.12]	0.510	45	0.011

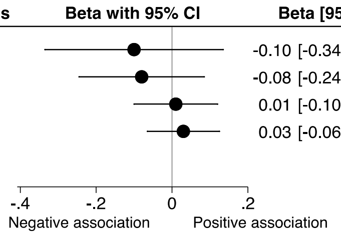

Data is presented as between group mean difference (95% confidence intervals) for a 1-unit change in the predictor variable. ß–coefficients were estimated using continuous meta-regression analysis. A positive ß-coefficient implies an increase in AST (U/L) with the food source of fructose-containing sugars intervention as the subgroup variable increases, and a negative ß-coefficient implies a decrease in uric acid. Residual I^2 reports inter-study heterogeneity not explained by the subgroup and was estimated using the Cochrane Q statistic.

[a] N=2 trials missing data for baseline AST.

AST=aspartate aminotransferase; CI=confidence interval; %E=percentage of total energy intake.

Supplementary Figure S68: Continuous meta-regression analysis for the effect of important food sources of fructose-containing sugars and AST (U/L) in addition trials

Continuous meta-regression

AST (U/L) in addition trials

Subgroup	Range	Trials	Beta [95% CI]	P_{MD}	Residual I^2 (%)	P_Q
Baseline AST (U/L)[a]	18.1—36.8	20	0.08 [-0.25 to 0.41]	0.638	10	0.328
Follow-up (weeks)	1.0—24.0	21	0.08 [-0.19 to 0.34]	0.545	12	0.302
Food source of fructose containing sugars dose (%E)	1.2—35.0	21	0.01 [-0.06 to 0.08]	0.785	12	0.300
Mean age (years)	24.6—58.9	21	-0.02 [-0.09 to 0.05]	0.494	9	0.342

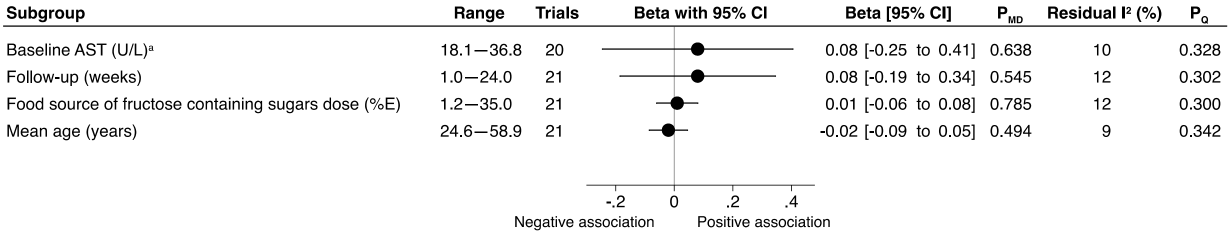

Data is presented as between group mean difference (95% confidence intervals) for a 1-unit change in the predictor variable. ß–coefficients were estimated using continuous meta-regression analysis. A positive ß-coefficient implies an increase in AST (U/L) with the food source of fructose-containing sugars intervention as the subgroup variable increases, and a negative ß-coefficient implies a decrease in uric acid. Residual I^2 reports inter-study heterogeneity not explained by the subgroup and was estimated using the Cochrane Q statistic.

[a] N=1 trial missing data for baseline AST.

AST=aspartate aminotransferase; CI=confidence interval; %E=percentage of total energy intake.

Supplementary Figure S69: Linear and non-linear meta-regression analyses for the effect of important food sources of fructose-containing sugars dose and IHCL (SMD) in substitution trials

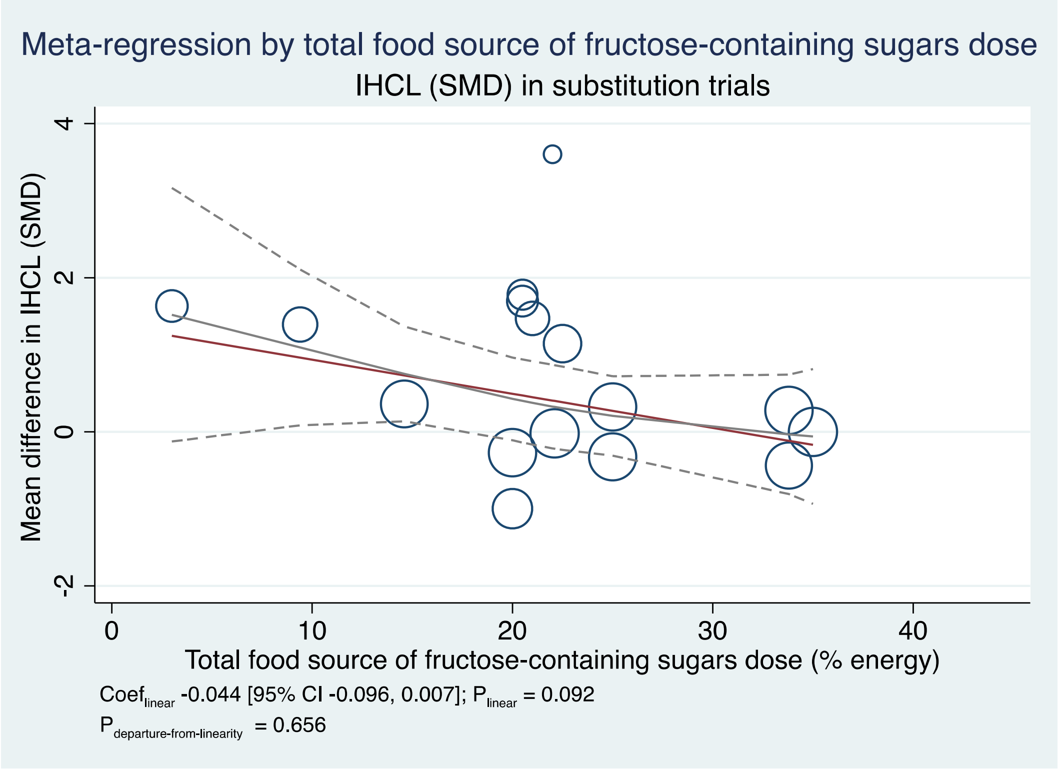

Individual trials are represented by the circles, with their weight in the overall analysis represented by the size of the circles. The straight line represents the estimate dose response for amount of fructose-containing sugars consumed (% of total energy intake) and the dashed lines represent the upper and lower 95% confidence intervals. Coef=coefficient; CI=confidence interval; IHCL=intrahepatocellular lipid; SMD=standardized mean difference.

Supplementary Figure S70: Linear and non-linear meta-regression analyses for the effect of important food sources of fructose-containing sugars dose and IHCL (SMD) in addition trials

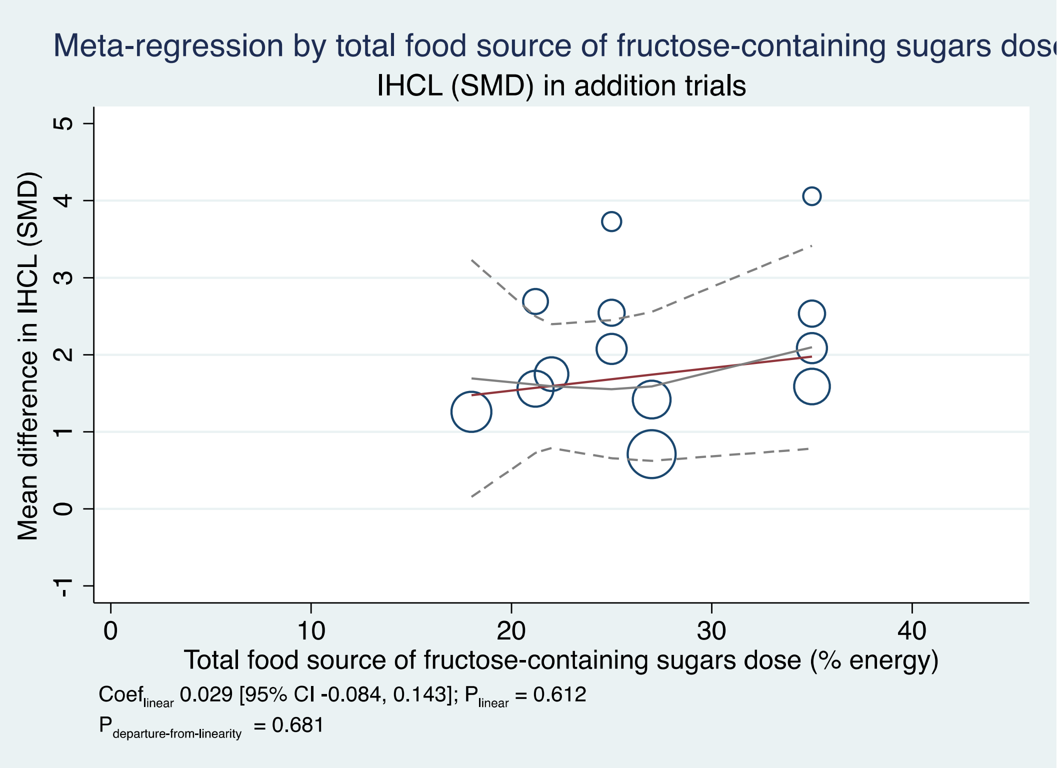

Individual trials are represented by the circles, with their weight in the overall analysis represented by the size of the circles. The straight line represents the estimate dose response for amount of fructose-containing sugars consumed (% of total energy intake) and the dashed lines represent the upper and lower 95% confidence intervals. Coef=coefficient; CI=confidence interval; IHCL=intrahepatocellular lipid; SMD=standardized mean difference.

Supplementary Figure S71: Linear and non-linear meta-regression analyses for the effect of important food sources of fructose-containing sugars dose and ALT (U/L) in substitution trials

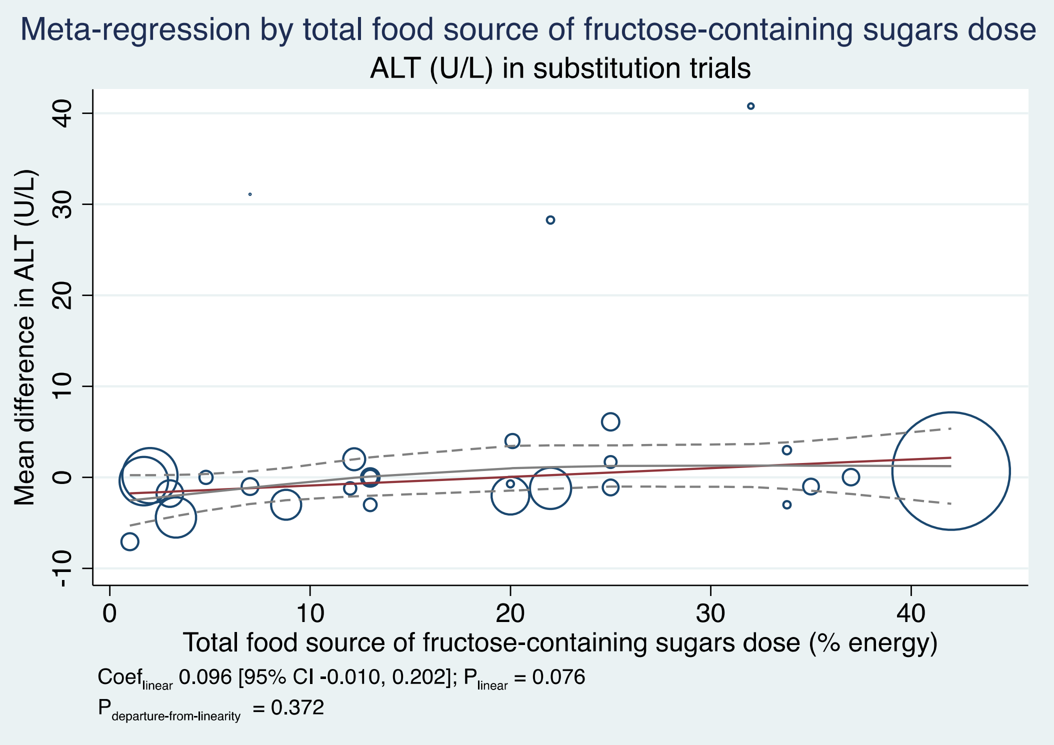

Individual trials are represented by the circles, with their weight in the overall analysis represented by the size of the circles. The straight line represents the estimate dose response for amount of fructose-containing sugars consumed (% of total energy intake) and the dashed lines represent the upper and lower 95% confidence intervals. ALT=alanine aminotransferase; coef=coefficient; CI=confidence interval.

Supplementary Figure S72: Linear and non-linear meta-regression analyses for the effect of important food sources of fructose-containing sugars dose and ALT (U/L) in addition trials

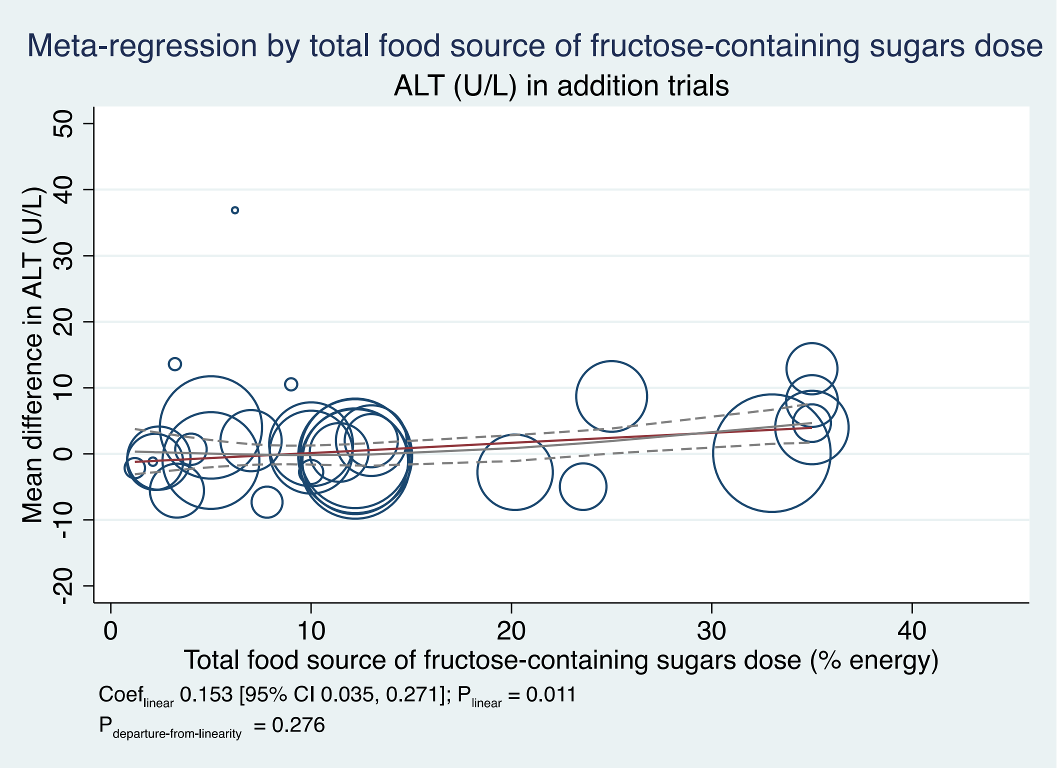

Individual trials are represented by the circles, with their weight in the overall analysis represented by the size of the circles. The straight line represents the estimate dose response for amount of fructose-containing sugars consumed (% of total energy intake) and the dashed lines represent the upper and lower 95% confidence intervals.
To convert coefficient to represent per serving of total sugars in a meal, multiply by 6 since according to national survey data, the average intake of total sugars is 18%E, which divided by 3 (for serving per meal) is 6%E(6). Thus, the equation can be expressed as 0.153U/L (95% CI: 0.035 to 0.271) * 6 = 0.918U/L (95% CI: 0.21, 1.626) per serving (6%E) of sugars in a meal
ALT=alanine aminotransferase; coef=coefficient; CI=confidence interval; %E=percentage of total energy intake.

Supplementary Figure S73: Linear and non-linear meta-regression analyses for the effect of SSBs on ALT (U/L) in addition trials

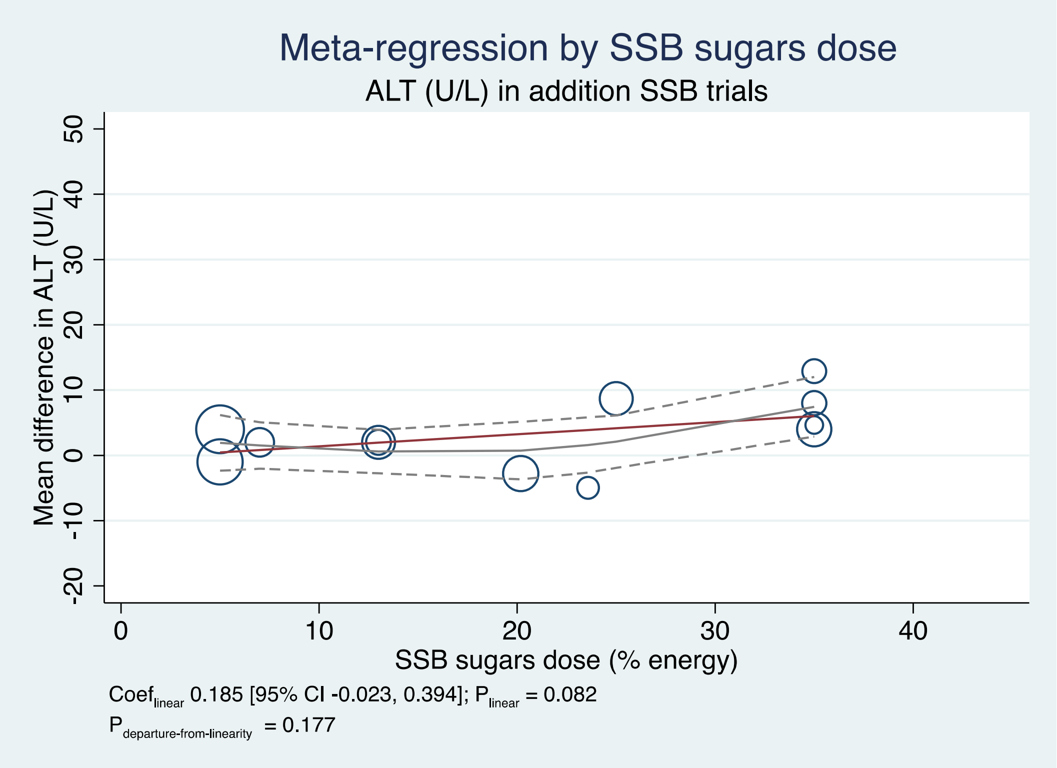

Individual trials are represented by the circles, with their weight in the overall analysis represented by the size of the circles. The straight line represents the estimate dose response for amount of fructose-containing sugars consumed (% of total energy intake) and the dashed lines represent the upper and lower 95% confidence intervals.
Linear and non-linear dose response not possible for fruit; dried fruit; added nutritive (caloric) sweetener; sweets and desserts; and mixed sources as there were fewer than six trial comparisons or only one unique dose was available.
To convert coefficient to represent per serving of SSB, multiply by 8 since 1 can (355mL) of cola is ~40g sugar or about 8%E of a 2000kcal diet. Thus, the equation can be expressed as 0.185U/L (95% CI: -0.023 to 0.394) * 8 = 1. 48U/L (95% CI: -0.184, 3.152) per serving (355ml, 8%E) of SSB.
ALT=alanine aminotransferase; coef=coefficient; CI=confidence interval; %E=percentage of total energy intake; SSB=sugar-sweetened beverage.

Supplementary Figure S74: Linear and non-linear meta-regression analyses for the effect of 100% fruit juice on ALT (U/L) in addition trials

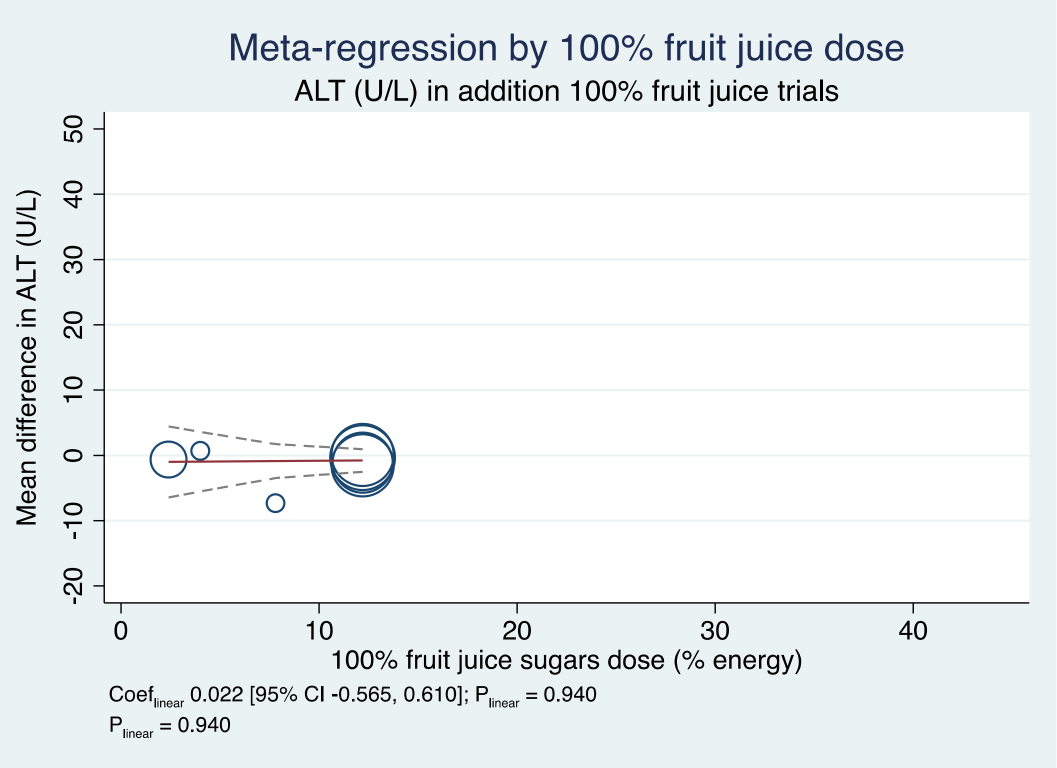

Individual trials are represented by the circles, with their weight in the overall analysis represented by the size of the circles. The straight line represents the estimate dose response for amount of fructose-containing sugars consumed (% of total energy intake) and the dashed lines represent the upper and lower 95% confidence intervals.
Linear and non-linear dose response not possible for fruit; dried fruit; added nutritive (caloric) sweetener; sweets and desserts; and mixed sources as there were fewer than six trial comparisons or only one unique dose was available.
To convert coefficient to represent per serving of SSB, multiply by 8 since 1 can (355mL) of cola is ~40g sugar or about 8%E of a 2000kcal diet. Thus, the equation can be expressed as 0.185U/L (95% CI: -0.023 to 0.394) * 8 = 1. 48U/L (95% CI: -0.184, 3.152) per serving (355ml, 8%E) of SSB.
ALT=alanine aminotransferase; coef=coefficient; CI=confidence interval; %E=percentage of total energy intake.

Supplementary Figure S75: Linear and non-linear meta-regression analyses for the effect of important food sources of fructose-containing sugars dose and ALT (U/L) in subtraction trials

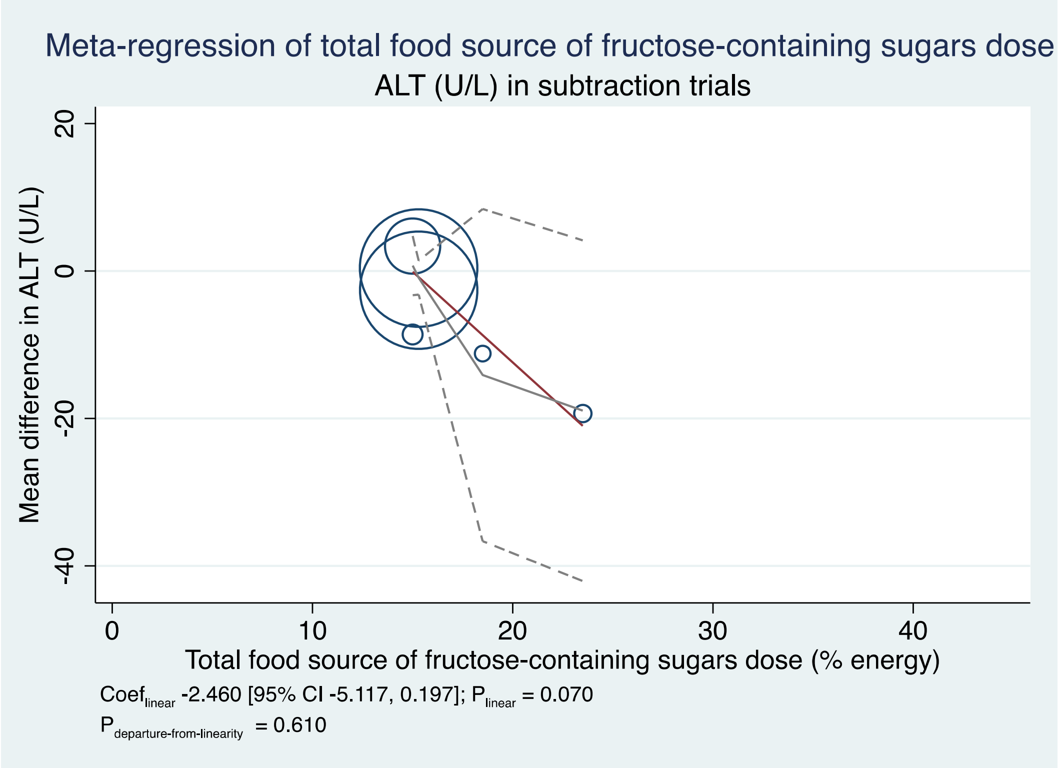

Individual trials are represented by the circles, with their weight in the overall analysis represented by the size of the circles. The straight line represents the estimate dose response for amount of fructose-containing sugars consumed (% of total energy intake) and the dashed lines represent the upper and lower 95% confidence intervals. ALT=alanine aminotransferase; coef=coefficient; CI=confidence interval; %E=percentage of total energy intake.

Supplementary Figure S76: Linear and non-linear meta-regression analyses for the effect of important food sources of fructose-containing sugars dose and AST (U/L) in substitution trials

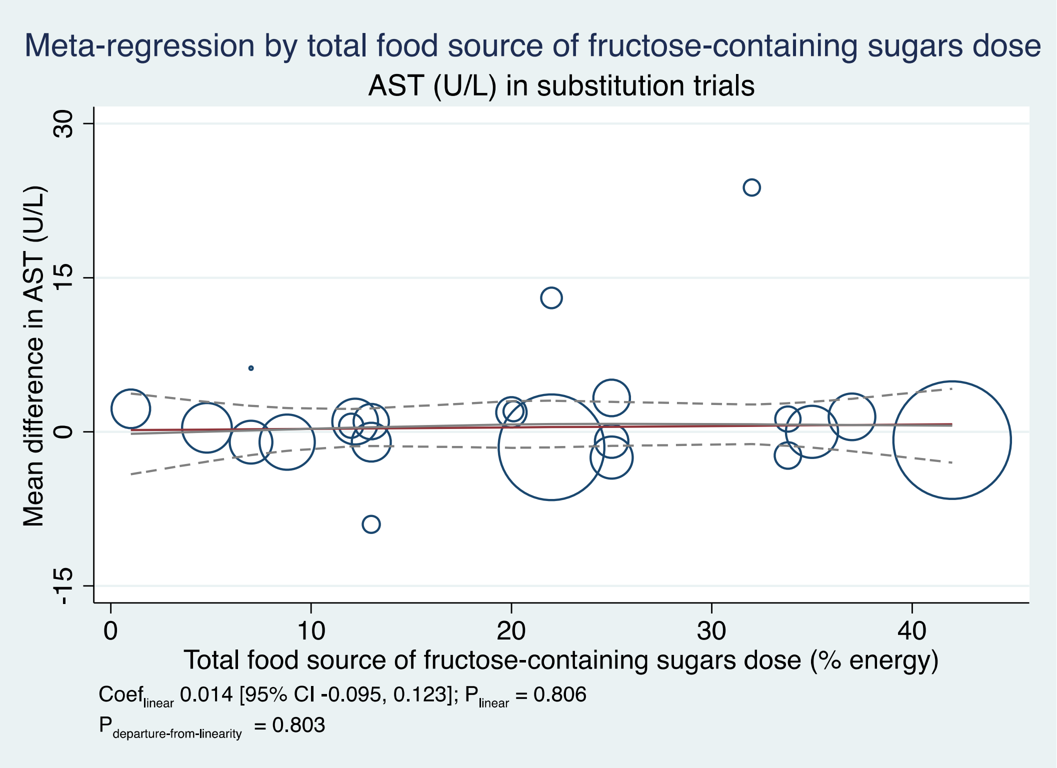

Individual trials are represented by the circles, with their weight in the overall analysis represented by the size of the circles. The straight line represents the estimate dose response for amount of fructose-containing sugars consumed (% of total energy intake) and the dashed lines represent the upper and lower 95% confidence intervals.
AST=aspartate aminotransferase; coef=coefficient; CI=confidence interval; %E=percentage of total energy intake.

Supplementary Figure S77: Linear and non-linear meta-regression analyses for the effect of important food sources of fructose-containing sugars dose and AST (U/L) in addition trials

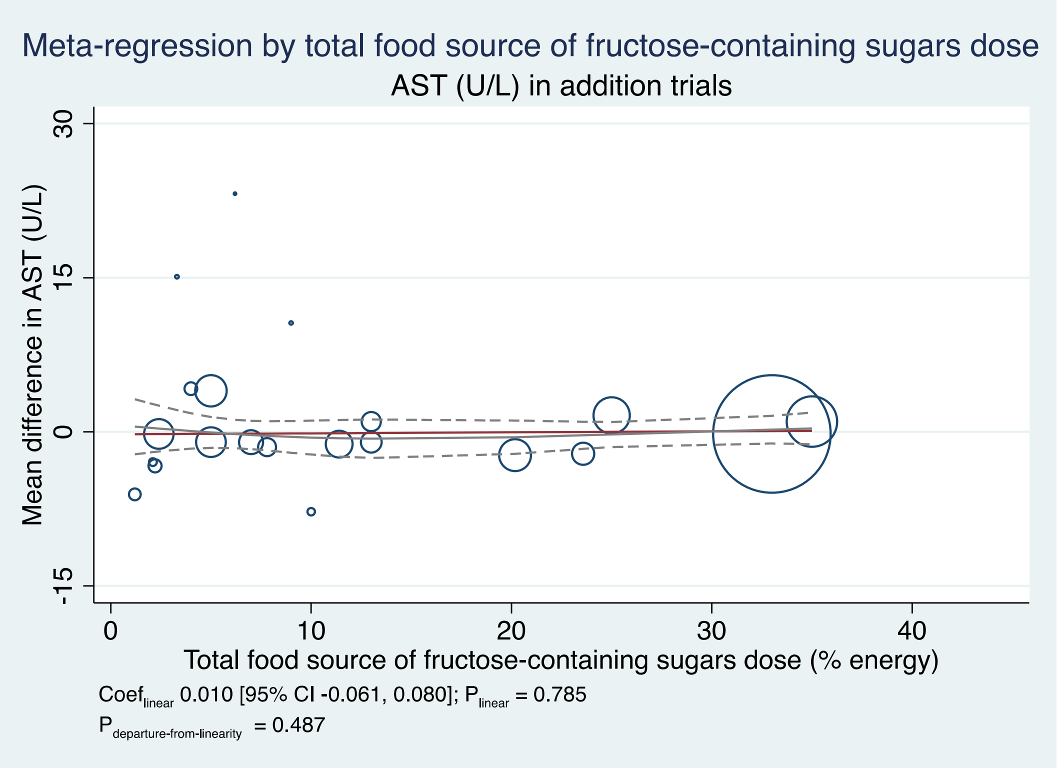

Individual trials are represented by the circles, with their weight in the overall analysis represented by the size of the circles. The straight line represents the estimate dose response for amount of fructose-containing sugars consumed (% of total energy intake) and the dashed lines represent the upper and lower 95% confidence intervals. AST=aspartate aminotransferase; coef=coefficient; CI=confidence interval; %E=percentage of total energy intake.

Supplementary Figure S78: Linear and non-linear meta-regression analyses for the effect of SSBs on AST (U/L) in addition trials

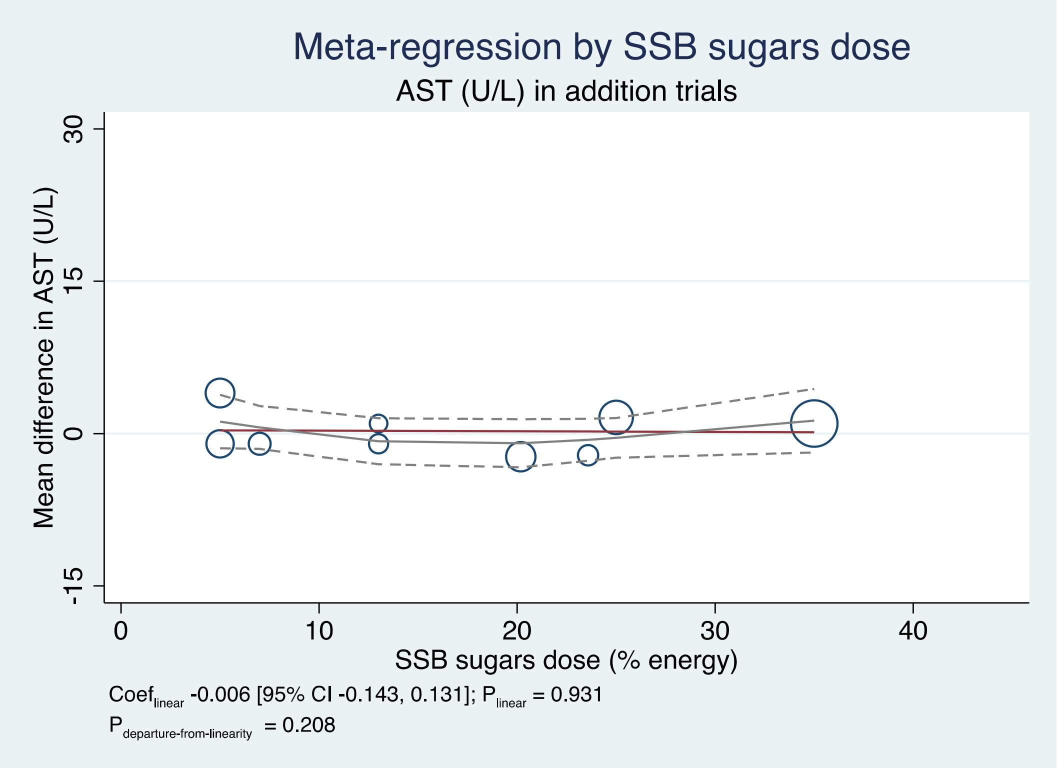

Individual trials are represented by the circles, with their weight in the overall analysis represented by the size of the circles. The straight line represents the estimate dose response for amount of fructose-containing sugars consumed (% of total energy intake) and the dashed lines represent the upper and lower 95% confidence intervals. AST=aspartate aminotransferase; coef=coefficient; CI=confidence interval; %E=percentage of total energy intake; SSB=sugar-sweetened beverage.

Supplementary Figure S79: Linear and non-linear meta-regression analyses for the effect of important food sources of fructose-containing sugars dose and AST (U/L) in subtraction trials

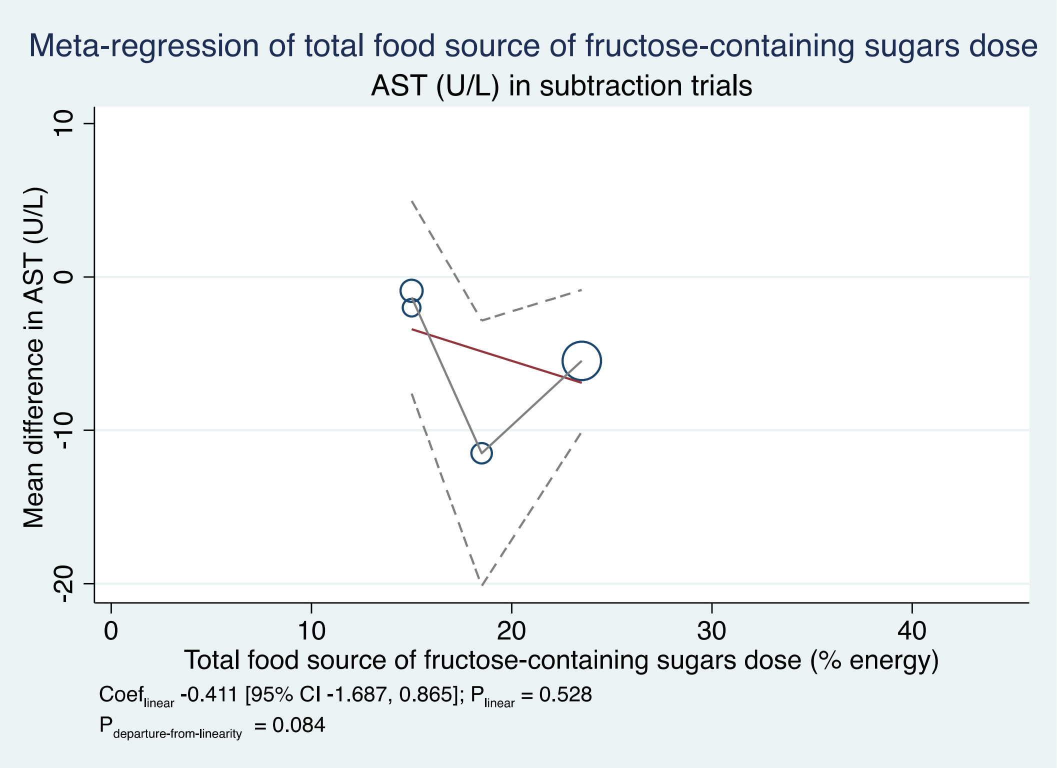

Individual trials are represented by the circles, with their weight in the overall analysis represented by the size of the circles. The straight line represents the estimate dose response for amount of fructose-containing sugars consumed (% of total energy intake) and the dashed lines represent the upper and lower 95% confidence intervals. AST=aspartate aminotransferase; coef=coefficient; CI=confidence interval.

Supplementary Figure S80: Non-linear dose-response analysis using public thresholds of 5%, 10%, and 25% of energy for the effect of important food sources of fructose-containing sugars and IHCL (SMD) in substitution trials

A

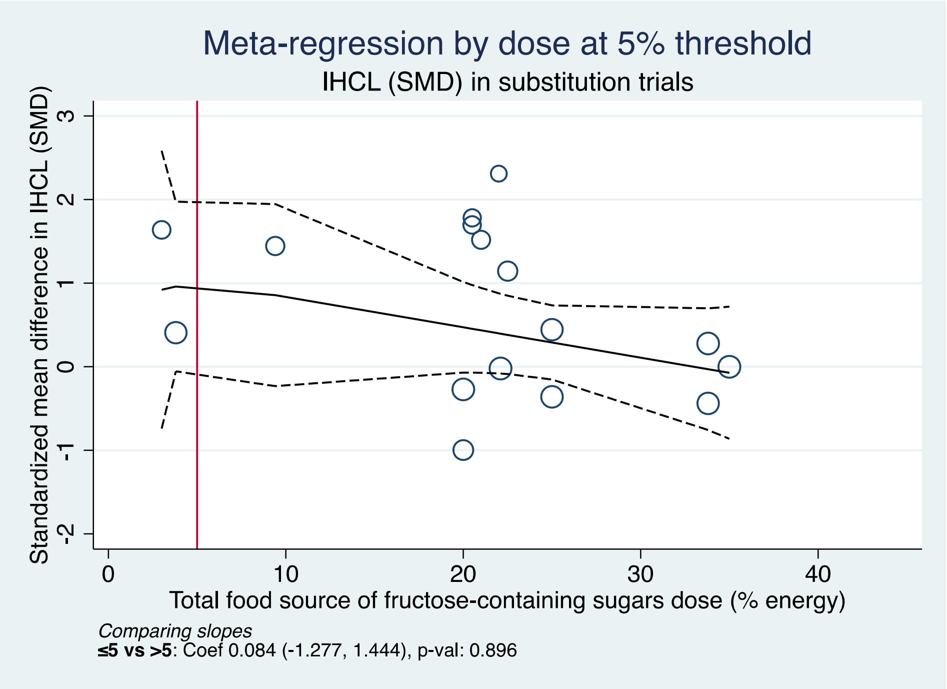

B

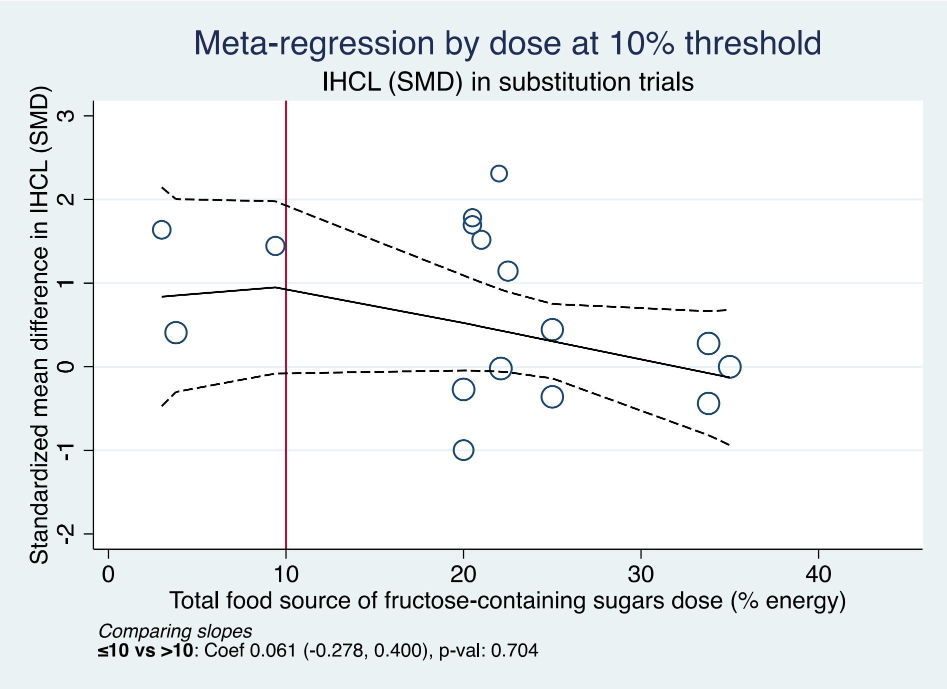

C

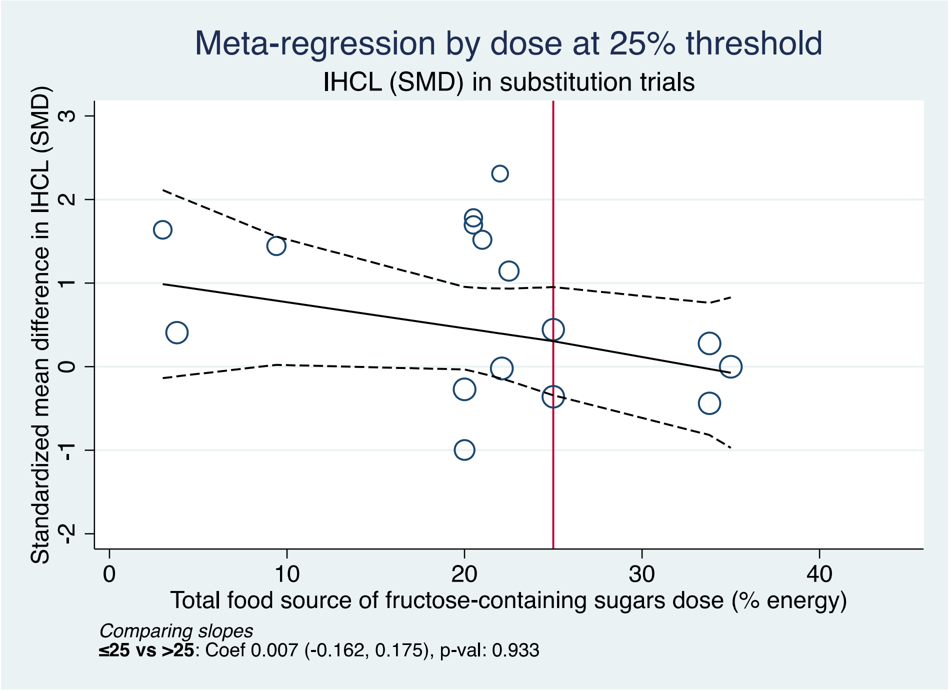

Individual trials are represented by the circles, with their weight in the overall analysis represented by the size of the circles. The straight line represents the estimate dose response for amount of fructose-containing sugars consumed (% of total energy intake) and the dashed lines represent the upper and lower 95% confidence intervals.
Panel A: 5% threshold; B: 10% threshold; C: 25% threshold.
Coef=coefficient; IHCL=intrahepatocellular lipid; SMD=standardized mean difference.

Supplementary Figure S81: Non-linear dose-response analysis using public threshold of 25% energy for the effect of important food sources of fructose-containing sugars and IHCL (SMD) in addition trials

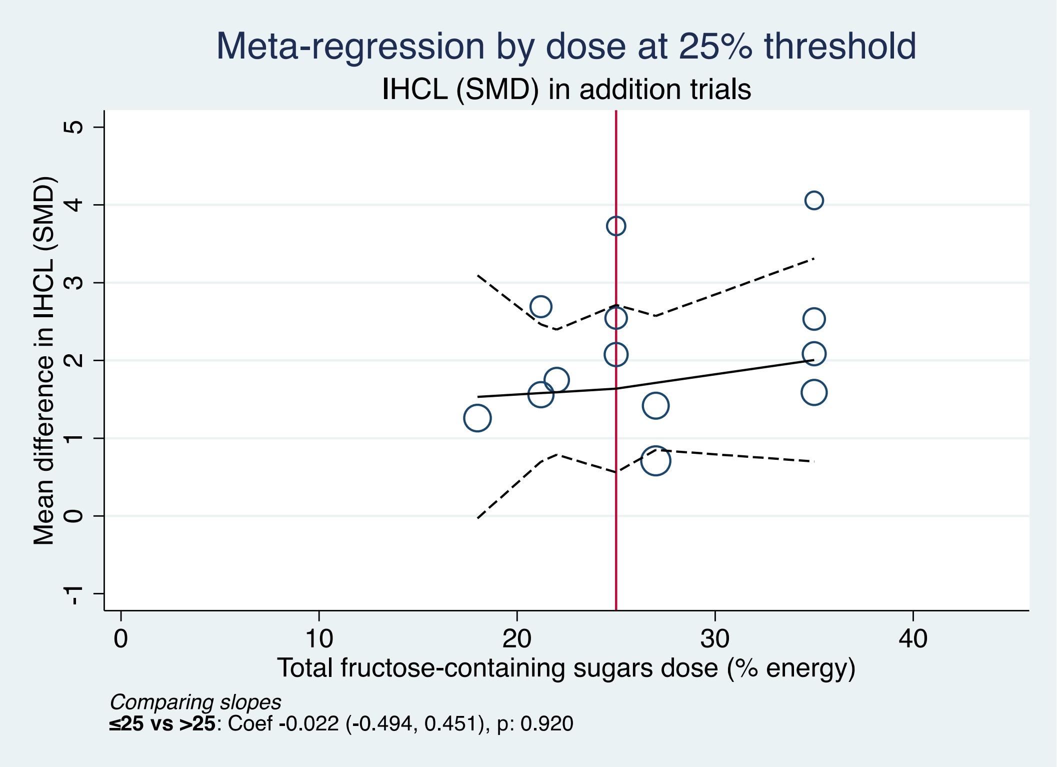

Individual trials are represented by the circles, with their weight in the overall analysis represented by the size of the circles. The straight line represents the estimate dose response for amount of fructose-containing sugars consumed (% of total energy intake) and the dashed lines represent the upper and lower 95% confidence intervals.
Non-linear dose-response analysis using public thresholds of 5% and 10% of energy was not conducted as there were no trials included which had a dose less than 10% of total energy.
Coef=coefficient; IHCL=intrahepatocellular lipid; SMD=standardized mean difference.

Supplementary Figure S82: Non-linear dose-response analysis using public thresholds of 5%, 10%, and 25% of energy for the effect of important food sources of fructose-containing sugars and ALT (U/L) in substitution trials

A

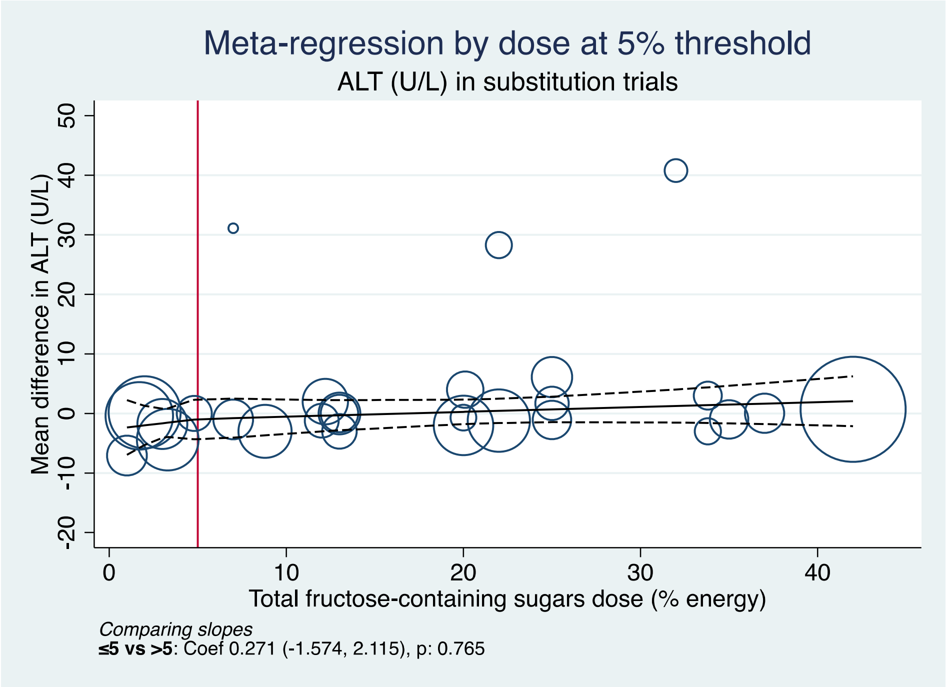

B

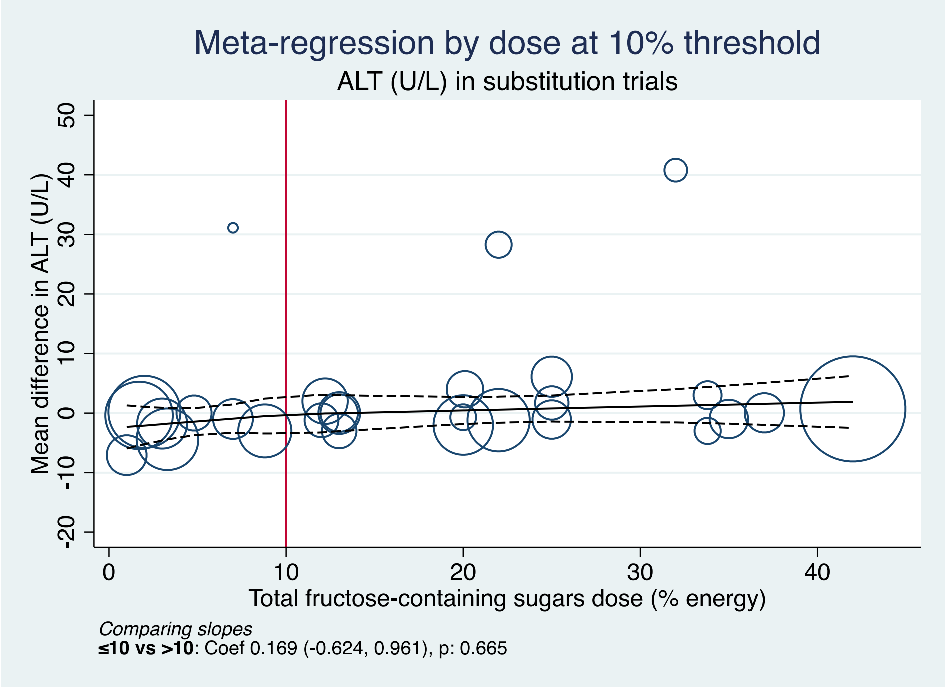

C

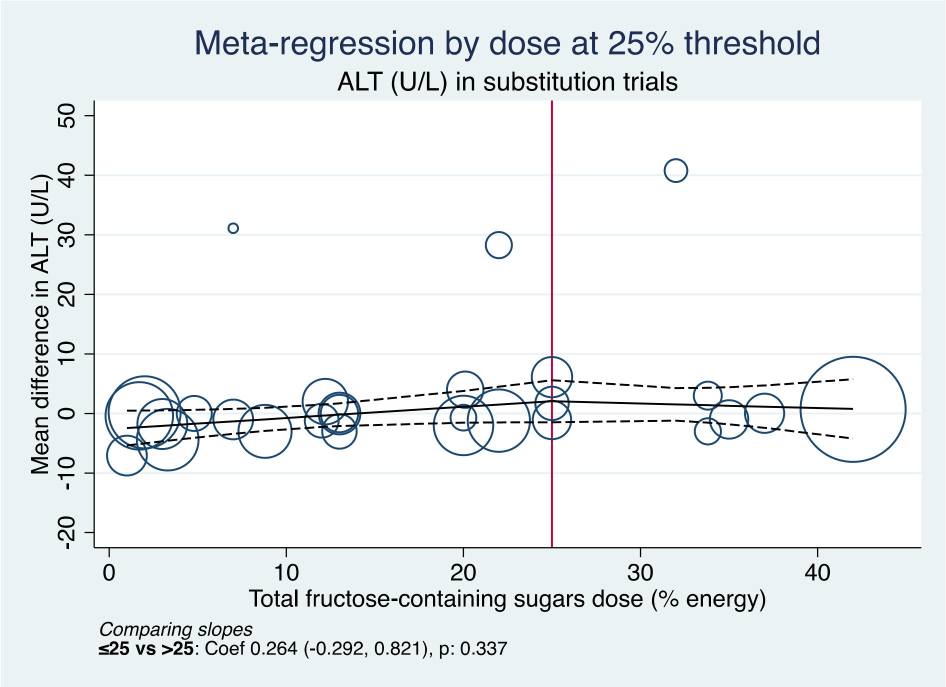

Individual trials are represented by the circles, with their weight in the overall analysis represented by the size of the circles. The horizontal straight line represents the estimate dose response for amount of fructose-containing sugars consumed (% of total energy intake), and the dashed lines represent the upper and lower 95% confidence intervals. The vertical straight lines represent the threshold knots.
Panel A: 5% threshold; B: 10% threshold; C: 25% threshold.
ALT=alanine aminotransferase; coef=coefficient; SSB=sugar-sweetened beverage.

Supplementary Figure S83: Non-linear dose-response analysis using public thresholds of 5%, 10%, and 25% of energy for the effect of important food sources of fructose-containing sugars and ALT (U/L) in addition trials

A

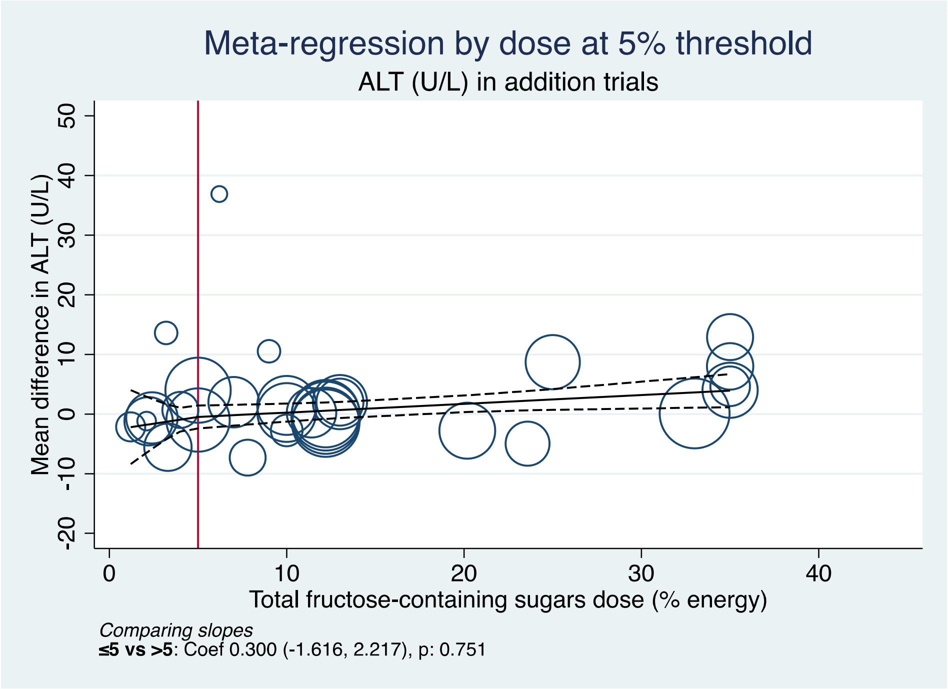

B

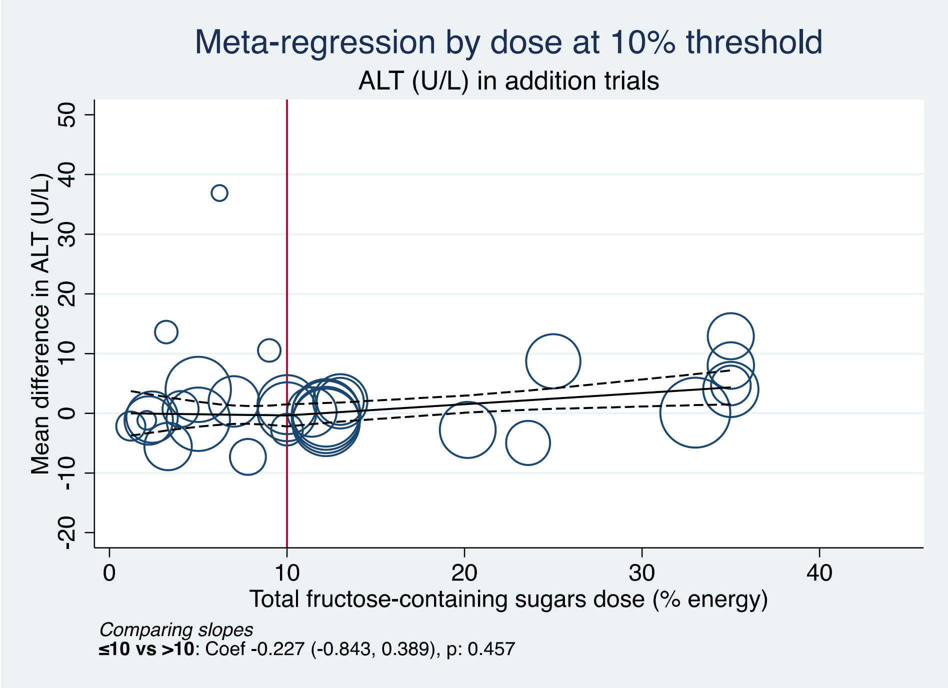

C

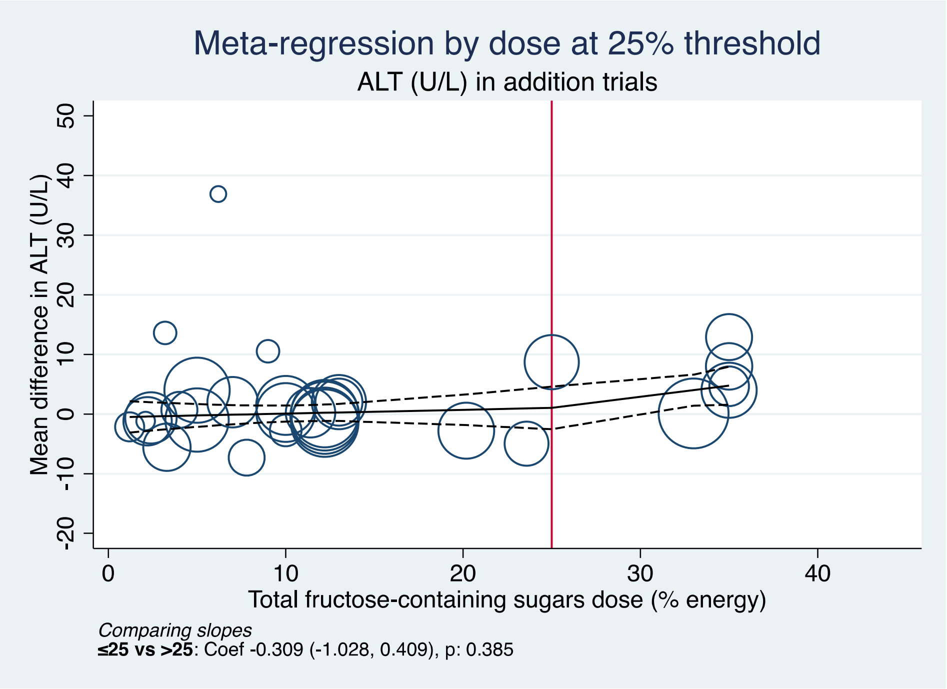

Individual trials are represented by the circles, with their weight in the overall analysis represented by the size of the circles. The straight line represents the estimate dose response for amount of fructose-containing sugars consumed (% of total energy intake) and the dashed lines represent the upper and lower 95% confidence intervals.
Panel A: 5% threshold; B: 10% threshold; C: 25% threshold.
ALT=alanine aminotransferase; coef=coefficient.

Supplementary Figure S84: Non-linear dose-response analysis using public thresholds of 10% and 25% of energy for the effect of SSBs on ALT (U/L) in addition trials

A

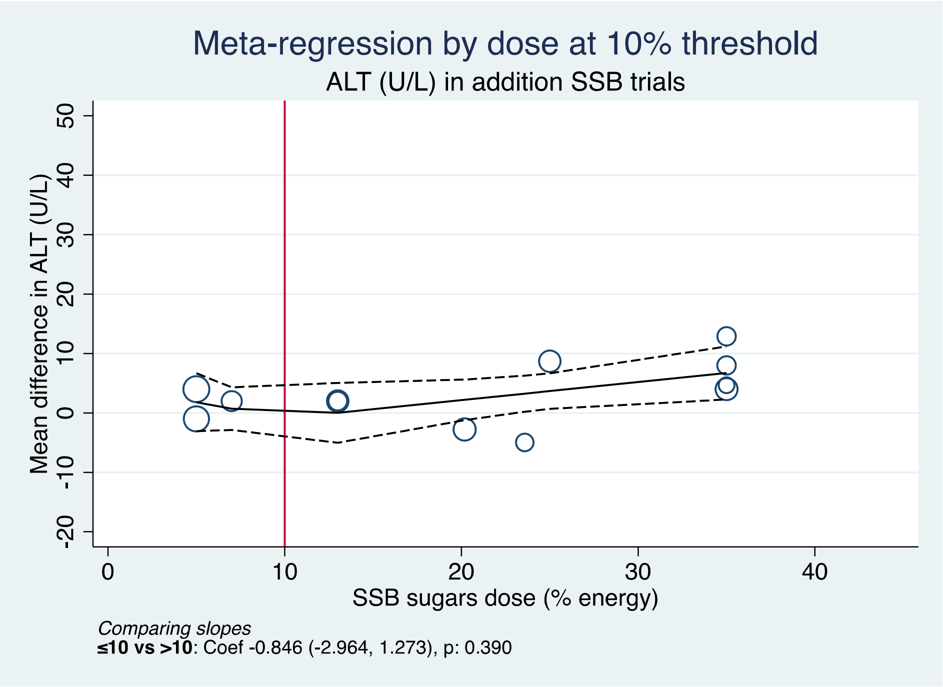

B

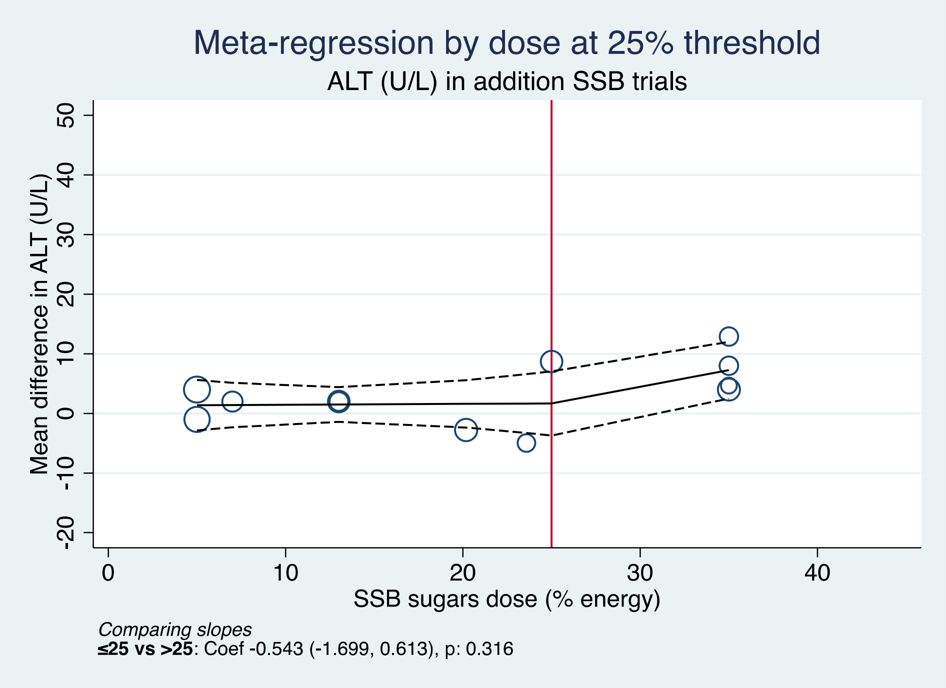

Individual trials are represented by the circles, with their weight in the overall analysis represented by the size of the circles. The straight line represents the estimate dose response for amount of fructose-containing sugars consumed (% of total energy intake) and the dashed lines represent the upper and lower 95% confidence intervals.
Panel A: 10% threshold; B: 25% threshold.
ALT=alanine aminotransferase; coef=coefficient; SSBs=sugar-sweetened beverage.

Supplementary Figure S85: Non-linear dose-response analysis using public thresholds of 5% and 10% of energy for the effect of 100% fruit juice on ALT (U/L) in addition trials

A

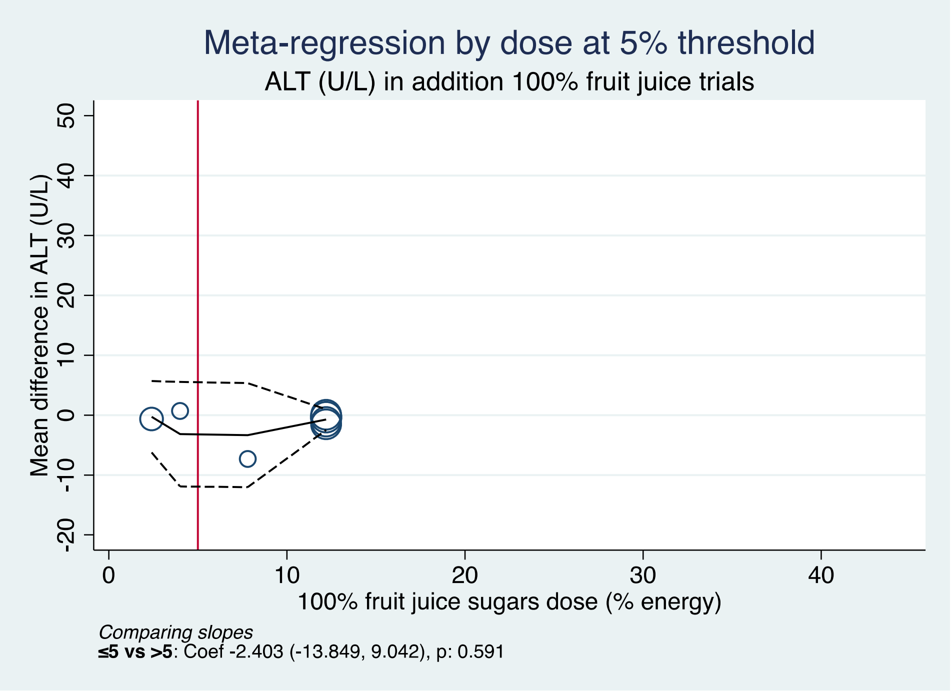

B

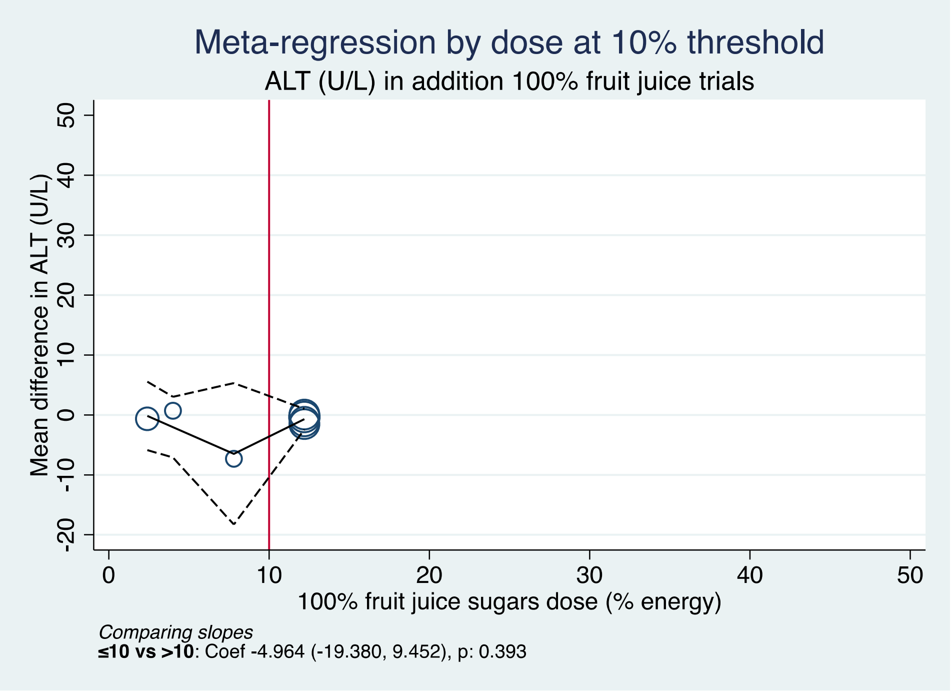

Individual trials are represented by the circles, with their weight in the overall analysis represented by the size of the circles. The straight line represents the estimate dose response for amount of fructose-containing sugars consumed (% of total energy intake) and the dashed lines represent the upper and lower 95% confidence intervals.

Panel A: 5% threshold; B: 10% threshold.

ALT=alanine aminotransferase; coef=coefficient.

Supplementary Figure S86: Non-linear dose-response analysis using public thresholds of 5%, 10%, and 25% of energy for the effect of important food sources of fructose-containing sugars and AST (U/L) in substitution trials

A

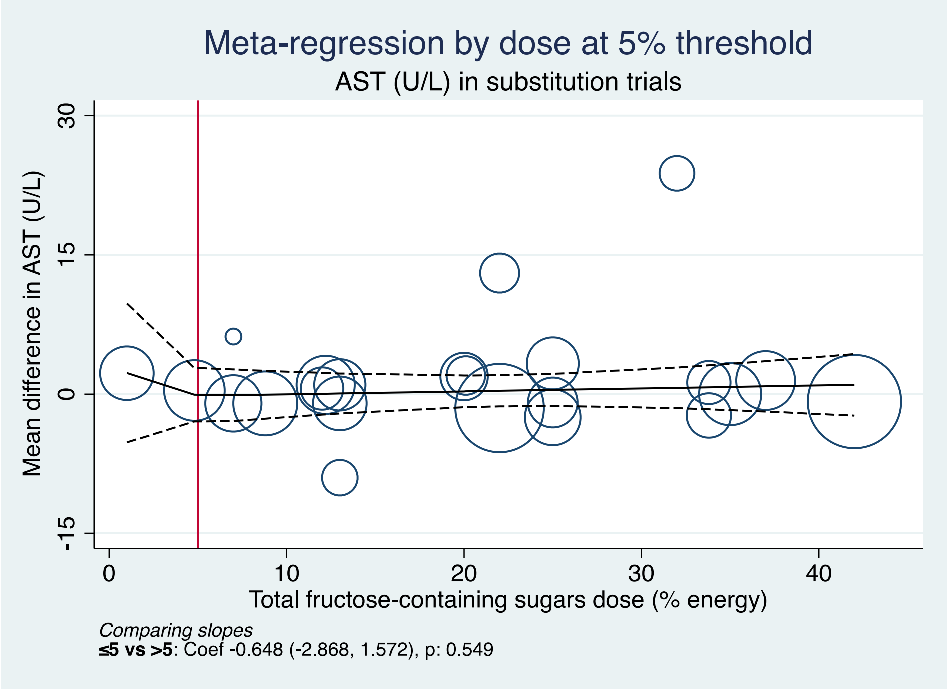

B

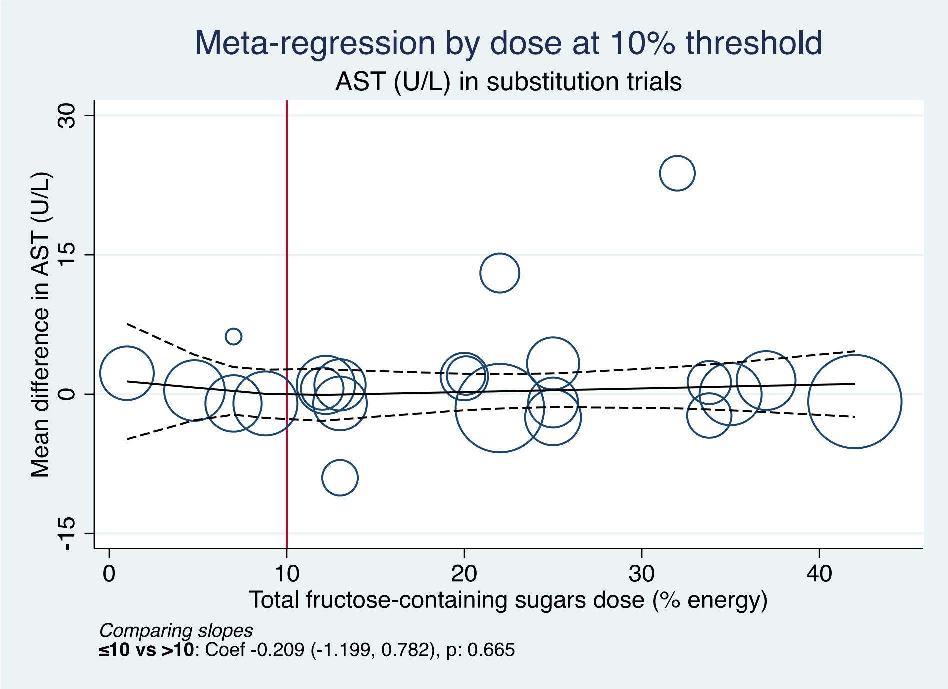

C

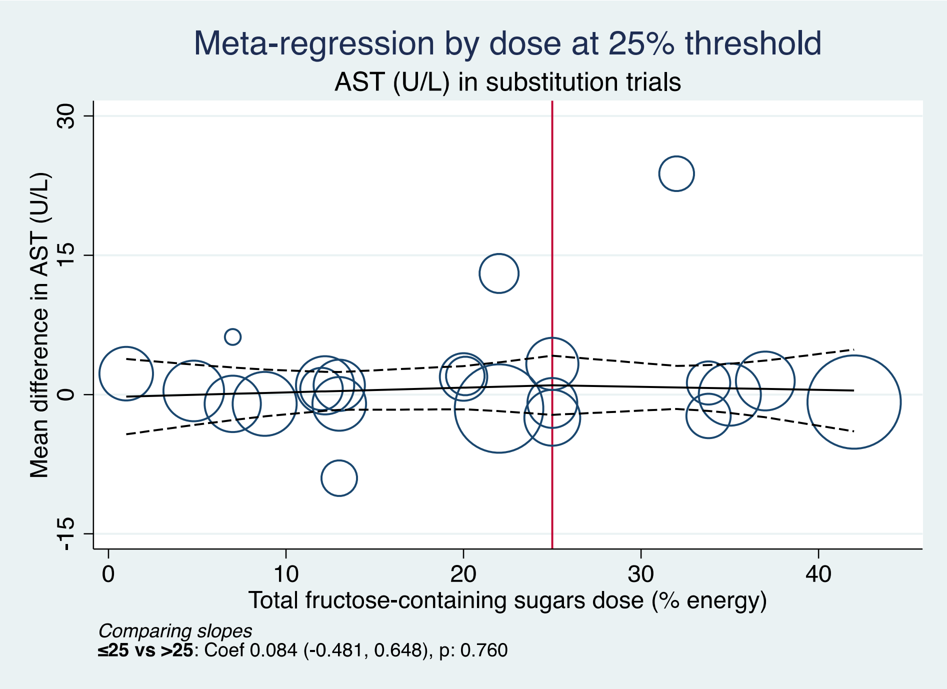

Individual trials are represented by the circles, with their weight in the overall analysis represented by the size of the circles. The horizontal straight line represents the estimate dose response for amount of fructose-containing sugars consumed (% of total energy intake), and the dashed lines represent the upper and lower 95% Confidence Intervals. The vertical straight lines represent the threshold knots.

Panel A: 5% threshold; B: 10% threshold; C: 25% threshold.

AST=aspartate aminotransferase; coef=coefficient.

Supplementary Figure S87: Non-linear dose-response analysis using public thresholds of 5%, 10%, and 25% of energy for the effect of important food sources of fructose-containing sugars and AST (U/L) in addition trials

A

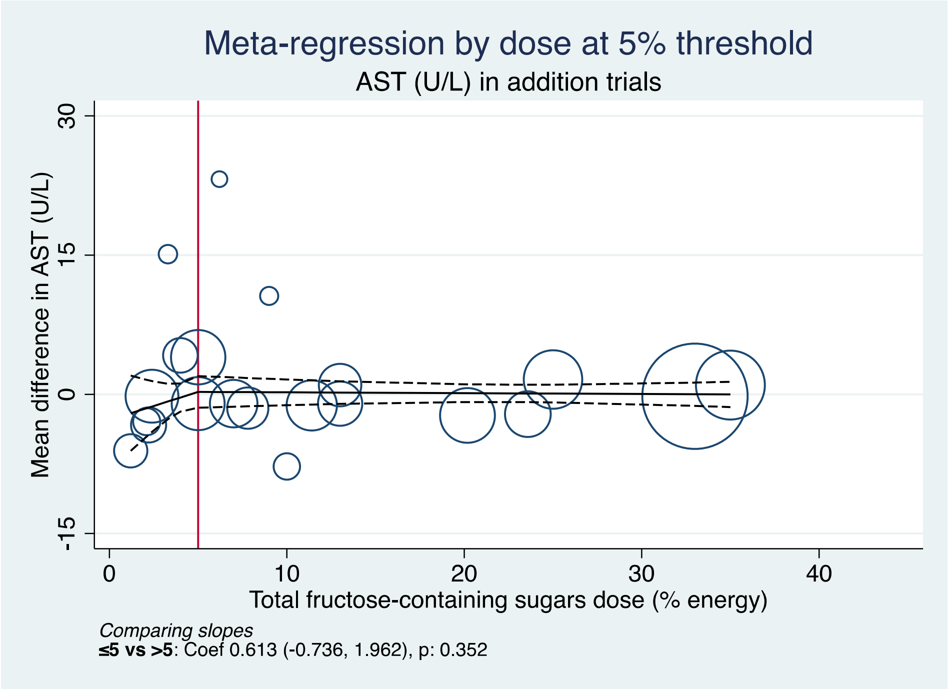

B

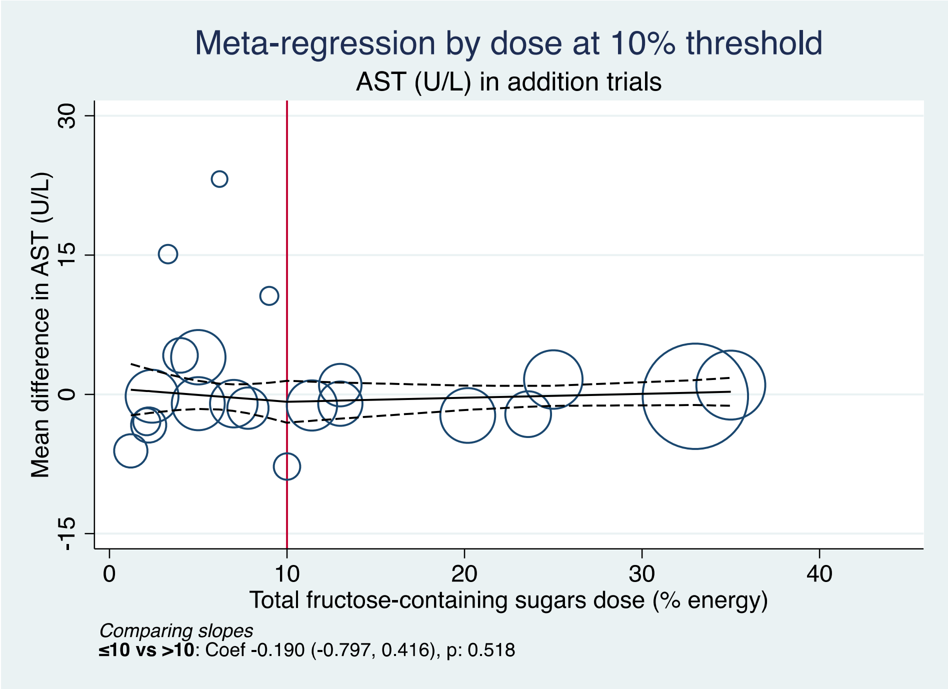

C

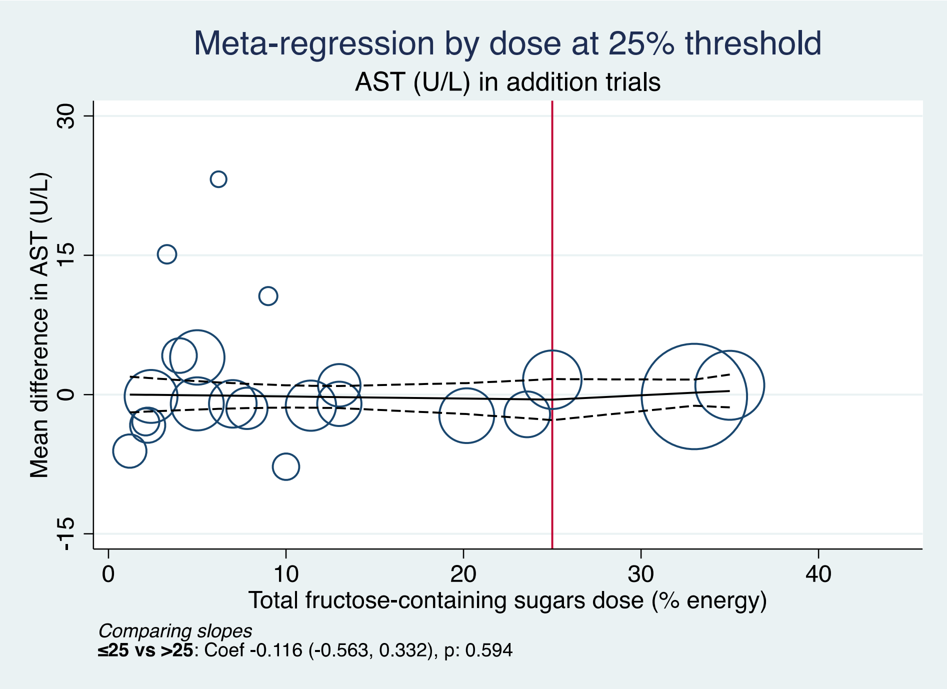

Individual trials are represented by the circles, with their weight in the overall analysis represented by the size of the circles. The horizontal straight line represents the estimate dose response for amount of fructose-containing sugars consumed (% of total energy intake), and the dashed lines represent the upper and lower 95% Confidence Intervals. The vertical straight lines represent the threshold knots.

Panel A: 5% threshold; B: 10% threshold; C: 25% threshold.

AST=aspartate aminotransferase; coef=coefficient.

Supplementary Figure S88: Non-linear dose-response analysis using public thresholds of 5%, 10%, and 25% of energy for the effect of SSBs on AST (U/L) in addition trials

A

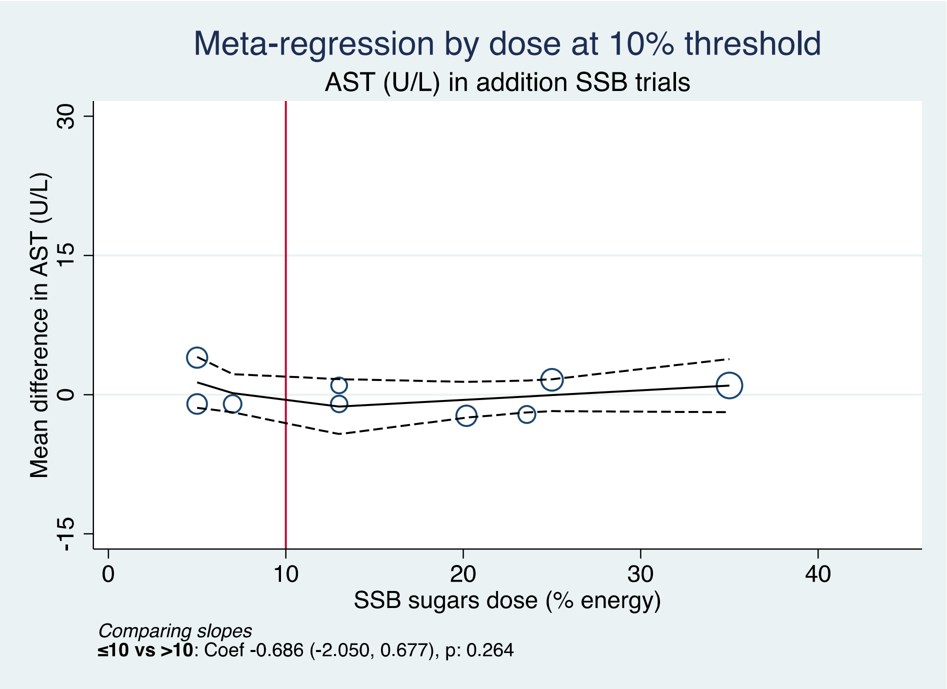

B

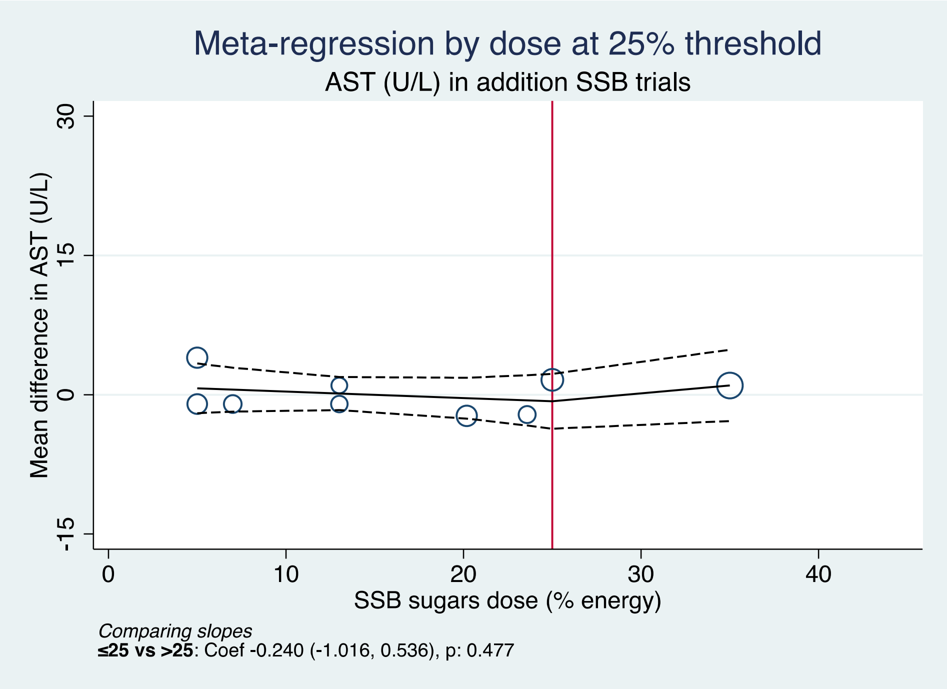

Individual trials are represented by the circles, with their weight in the overall analysis represented by the size of the circles. The horizontal straight line represents the estimate dose response for amount of fructose-containing sugars consumed (% of total energy intake), and the dashed lines represent the upper and lower 95% Confidence Intervals. The vertical straight lines represent the threshold knots.

Panel A: 10% threshold; B: 25% threshold.

AST=aspartate aminotransferase; coef=coefficient; SSB=sugar-sweetened beverage.

Supplementary Figure S89: Publication bias funnel plots for the effect of important food sources of fructose-containing sugars and IHCL (SMD) in substitution trials

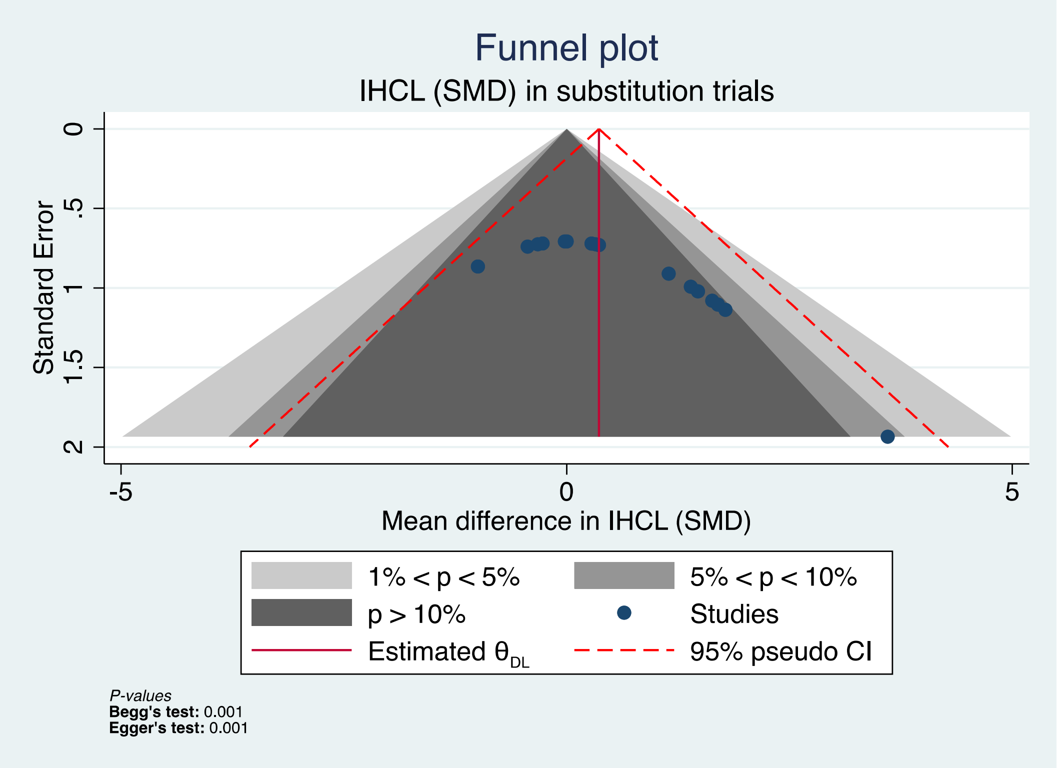

Contour-enhanced funnel plot is a scatterplot of each trial weighted mean difference on the x-axis with the standard error representing precision on the y-axis. The vertical solid red line represents the pooled effect estimate and the dashed red lines represent the pseudo-95% confidence limits. The blue dots represent individual trials. The contour regions define the regions for the test of significance of individual trial effect size for a given p-value range >0.100 (dark grey), 0.500 to <0.100 (medium grey), 0.010 to <0.500 (light grey), <0.0100 (white)]. The contour-enhanced funnel plots may suggest funnel-plot asymmetry is due to publication bias when less precise (smaller) trials are missing in the non-significant regions. Quantitative assessment of publication bias was also performed using Egger's and Begg's tests set at a significance level of p<0.100.
CI=confidence interval; IHCL=intrahepatocellular lipid; DL=DerSimmonian Laird; SMD=standardized mean difference.

Supplementary Figure S90: Publication bias funnel plots for the effect of important food sources of fructose-containing sugars and IHCL (SMD) in addition trials*

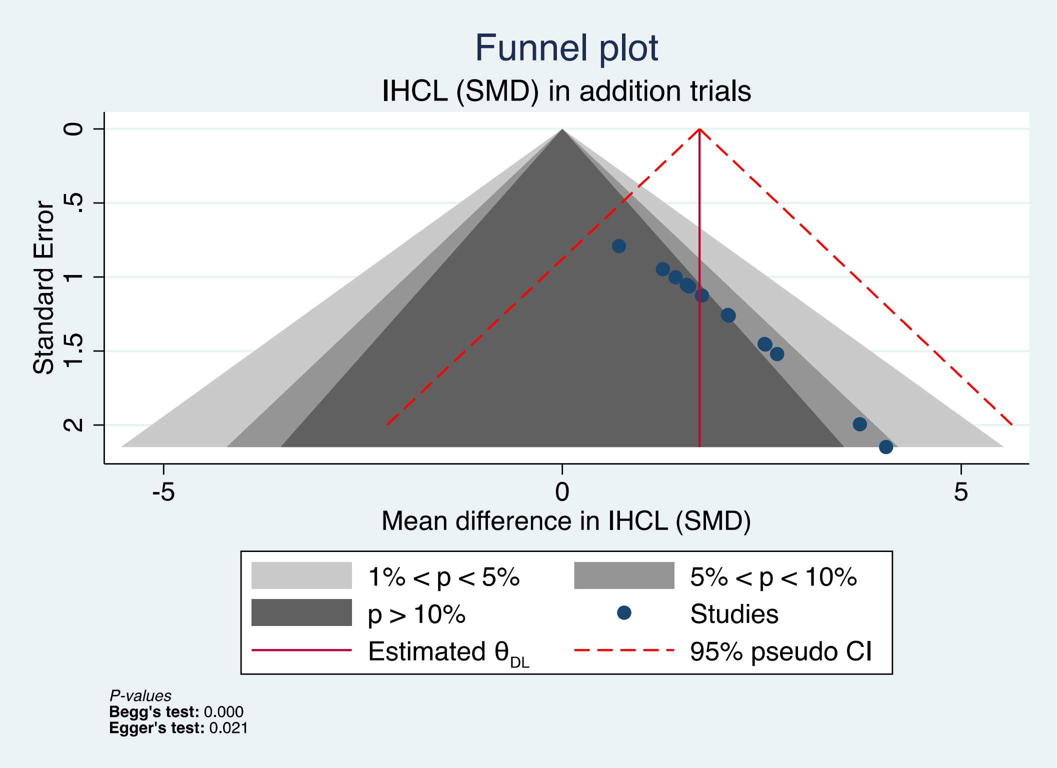

Contour-enhanced funnel plot is a scatterplot of each trial weighted mean difference on the x-axis with the standard error representing precision on the y-axis. The vertical solid red line represents the pooled effect estimate and the dashed red lines represent the pseudo-95% confidence limits. The blue dots represent individual trials. The contour regions define the regions for the test of significance of individual trial effect size for a given p-value range >0.100 (dark grey), 0.500 to <0.100 (medium grey), 0.010 to <0.500 (light grey), <0.0100 (white)]. The contour-enhanced funnel plots may suggest funnel-plot asymmetry is due to publication bias when less precise (smaller) trials are missing in the non-significant regions. Quantitative assessment of publication bias was also performed using Egger's and Begg's tests set at a significance level of p<0.100.

*All included addition trials were sugar-sweetened beverages.

CI=confidence interval; IHCL=intrahepatocellular lipid; SMD=standardized mean difference. CI=confidence interval; DL=DerSimmonian Laird; IHCL=intrahepatocellular lipid; SMD=standardized mean difference.

Supplementary Figure S91: Publication bias funnel plots for the effect of important food sources of fructose-containing sugars and ALT (U/L) in substitution trials

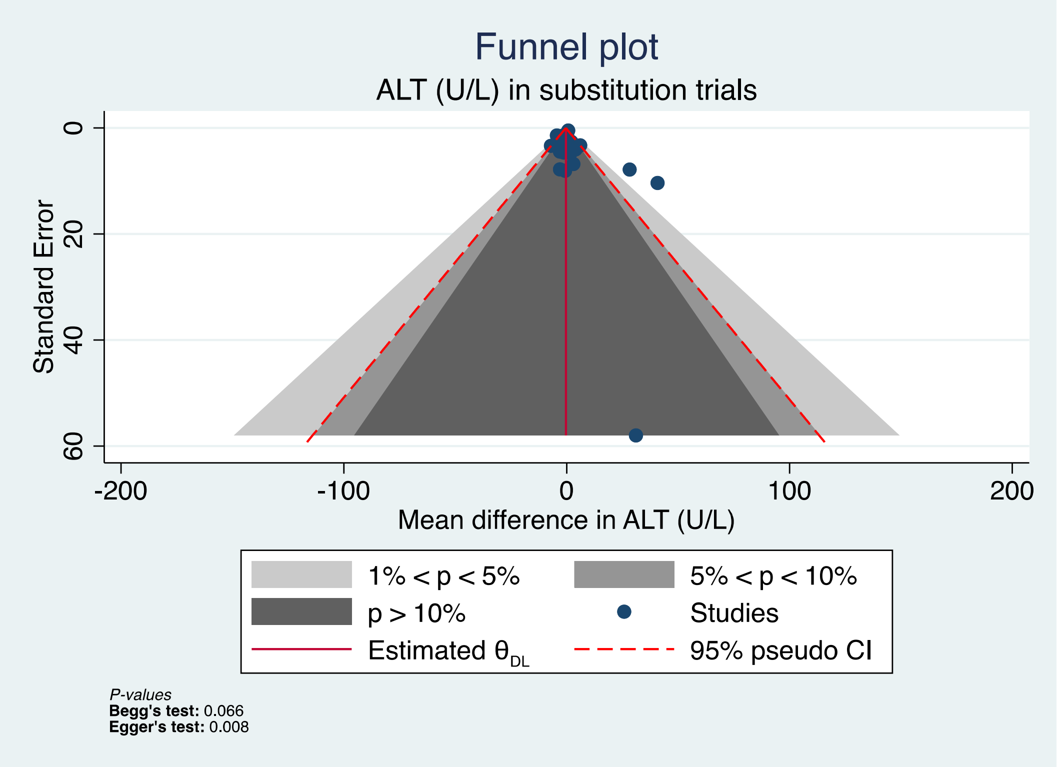

Contour-enhanced funnel plot is a scatterplot of each trial weighted mean difference on the x-axis with the standard error representing precision on the y-axis. The vertical solid red line represents the pooled effect estimate and the dashed red lines represent the pseudo-95% confidence limits. The blue dots represent individual trials. The contour regions define the regions for the test of significance of individual trial effect size for a given p-value range >0.100 (dark grey), 0.500 to <0.100 (medium grey), 0.010 to <0.500 (light grey), <0.0100 (white)]. The contour-enhanced funnel plots may suggest funnel-plot asymmetry is due to publication bias when less precise (smaller) trials are missing in the non-significant regions. Quantitative assessment of publication bias was also performed using Egger's and Begg's tests set at a significance level of p<0.100.
ALT=alanine aminotransferase; CI=confidence interval; DL=DerSimmonian Laird.

Supplementary Figure S92: Publication bias funnel plots for the effect of important food sources of fructose-containing sugars and ALT (U/L) in addition trials

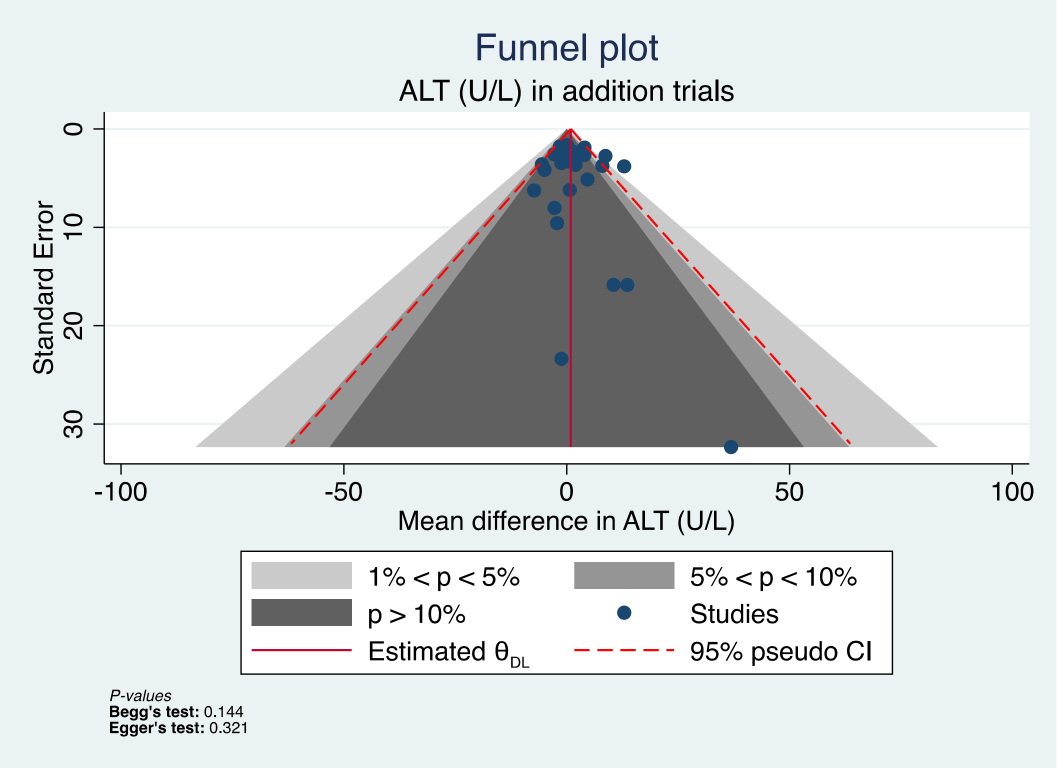

Contour-enhanced funnel plot is a scatterplot of each trial weighted mean difference on the x-axis with the standard error representing precision on the y-axis. The vertical solid red line represents the pooled effect estimate and the dashed red lines represent the pseudo-95% confidence limits. The blue dots represent individual trials. The contour regions define the regions for the test of significance of individual trial effect size for a given p-value range >0.100 (dark grey), 0.500 to <0.100 (medium grey), 0.010 to <0.500 (light grey), <0.0100 (white)]. The contour-enhanced funnel plots may suggest funnel-plot asymmetry is due to publication bias when less precise (smaller) trials are missing in the non-significant regions. Quantitative assessment of publication bias was also performed using Egger's and Begg's tests set at a significance level of p<0.100.
ALT=alanine aminotransferase; CI=confidence interval; DL=DerSimmonian Laird.

Supplementary Figure S93: Publication bias funnel plots for the effect of SSBs on ALT (U/L) in addition trials

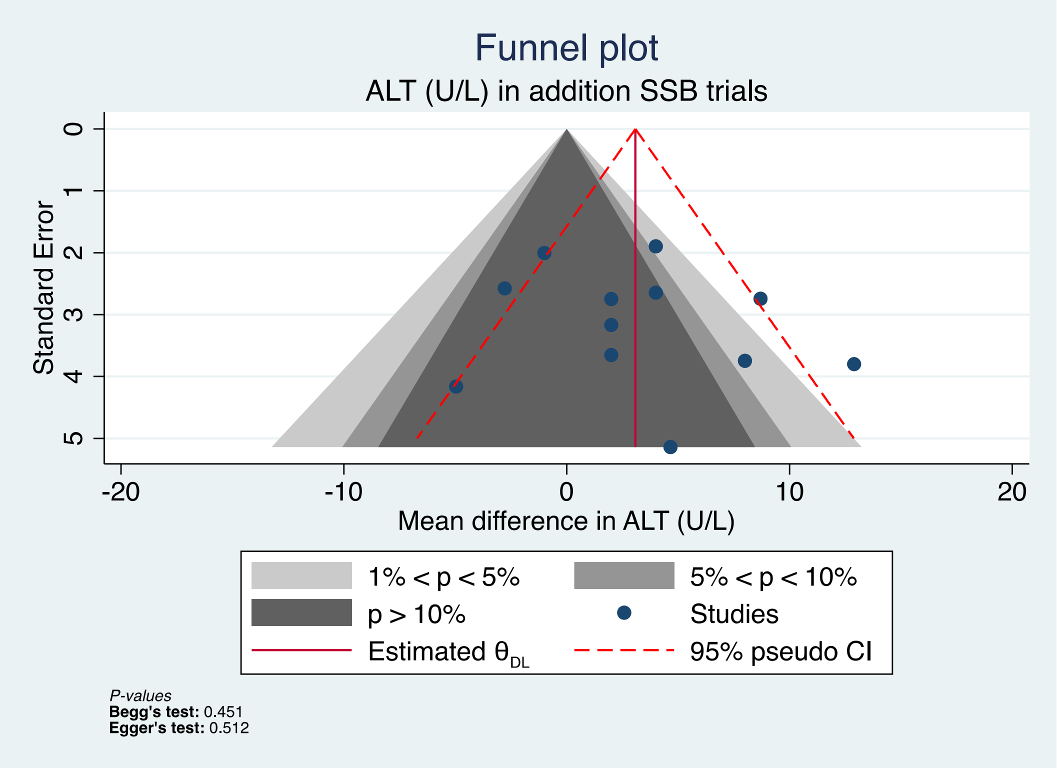

Contour-enhanced funnel plot is a scatterplot of each trial weighted mean difference on the x-axis with the standard error representing precision on the y-axis. The vertical solid red line represents the pooled effect estimate and the dashed red lines represent the pseudo-95% confidence limits. The blue dots represent individual trials. The contour regions define the regions for the test of significance of individual trial effect size for a given p-value range >0.100 (dark grey), 0.500 to <0.100 (medium grey), 0.010 to <0.500 (light grey), <0.0100 (white)]. The contour-enhanced funnel plots may suggest funnel-plot asymmetry is due to publication bias when less precise (smaller) trials are missing in the non-significant regions. Quantitative assessment of publication bias was also performed using Egger's and Begg's tests set at a significance level of p<0.100.
ALT=alanine aminotransferase; CI=confidence interval; DL=DerSimmonian Laird; SSB=sugar-sweetened beverage.

Supplementary Figure S94: Publication bias funnel plots for the effect of important food sources of fructose-containing sugars and AST (U/L) in substitution trials

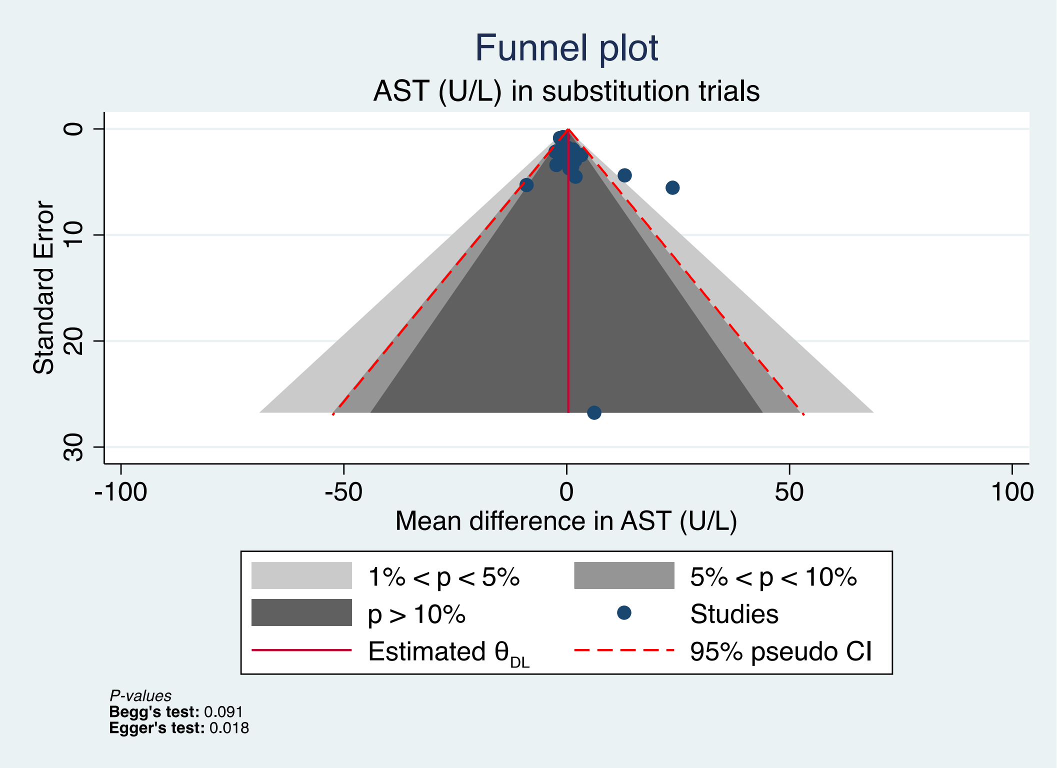

Contour-enhanced funnel plot is a scatterplot of each trial weighted mean difference on the x-axis with the standard error representing precision on the y-axis. The vertical solid red line represents the pooled effect estimate and the dashed red lines represent the pseudo-95% confidence limits. The blue dots represent individual trials. The contour regions define the regions for the test of significance of individual trial effect size for a given p-value range >0.100 (dark grey), 0.500 to <0.100 (medium grey), 0.010 to <0.500 (light grey), <0.0100 (white)]. The contour-enhanced funnel plots may suggest funnel-plot asymmetry is due to publication bias when less precise (smaller) trials are missing in the non-significant regions. Quantitative assessment of publication bias was also performed using Egger's and Begg's tests set at a significance level of p<0.100.
AST=aspartate aminotransferase; CI=confidence interval; DL=DerSimmonian Laird.

Supplementary Figure S95: Publication bias funnel plots for the effect of important food sources of fructose-containing sugars and AST (U/L) in addition trials

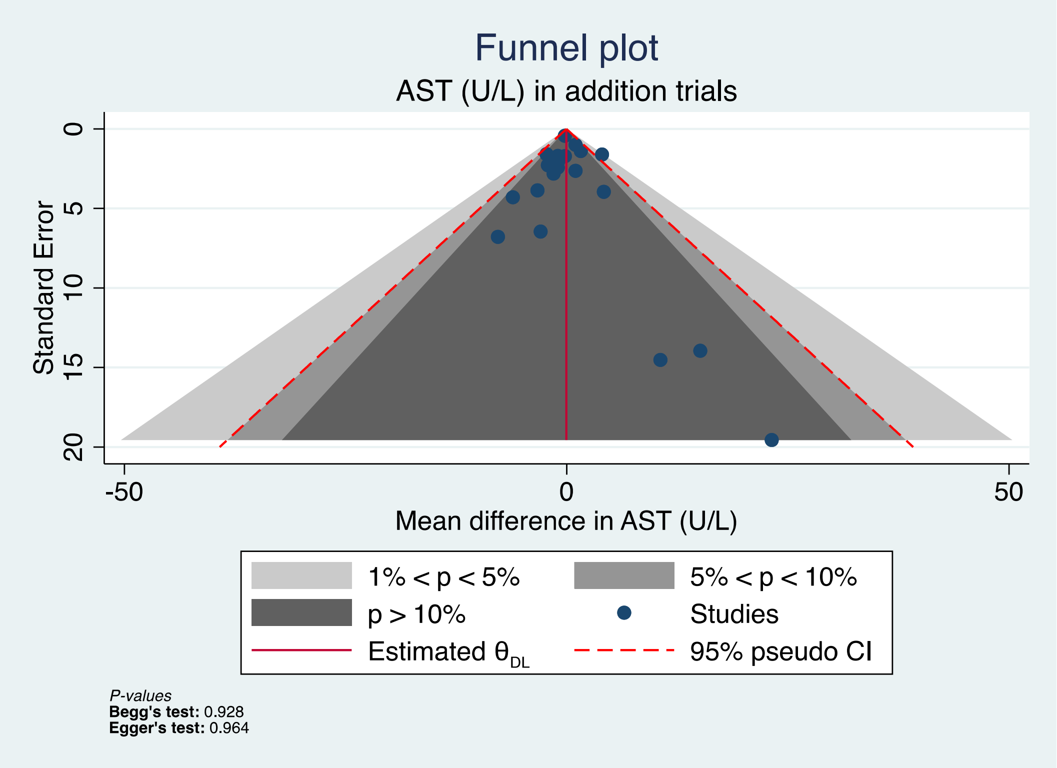

The dots represent individual trial comparisons. The vertical solid red line represents the pooled effect estimate as the weighted mean difference (MD) and the dashed red lines represent the pseudo-95% confidence limits. The contour regions define the regions of statistically significant and nonsignificant levels with dark grey representing p>0.1, medium grey p >0.05 to ≤0.1, light grey p≤0.01 to p<0.05 and no shading p<0.01. Publication bias is suspect if there are studies, especially smaller studies, that are missing in the nonsignificant regions. The p-values were derived from quantitative assessment of publication bias by Egger's and Begg's tests set at a significance level of p<0.05.
AST=aspartate aminotransferase; CI=confidence interval; DL=DerSimmonian Laird.

Supplementary Figure S96: Trim and Fill funnel plot for the effect of important food sources of fructose-containing sugars IHCL (SMD) in substitution trials

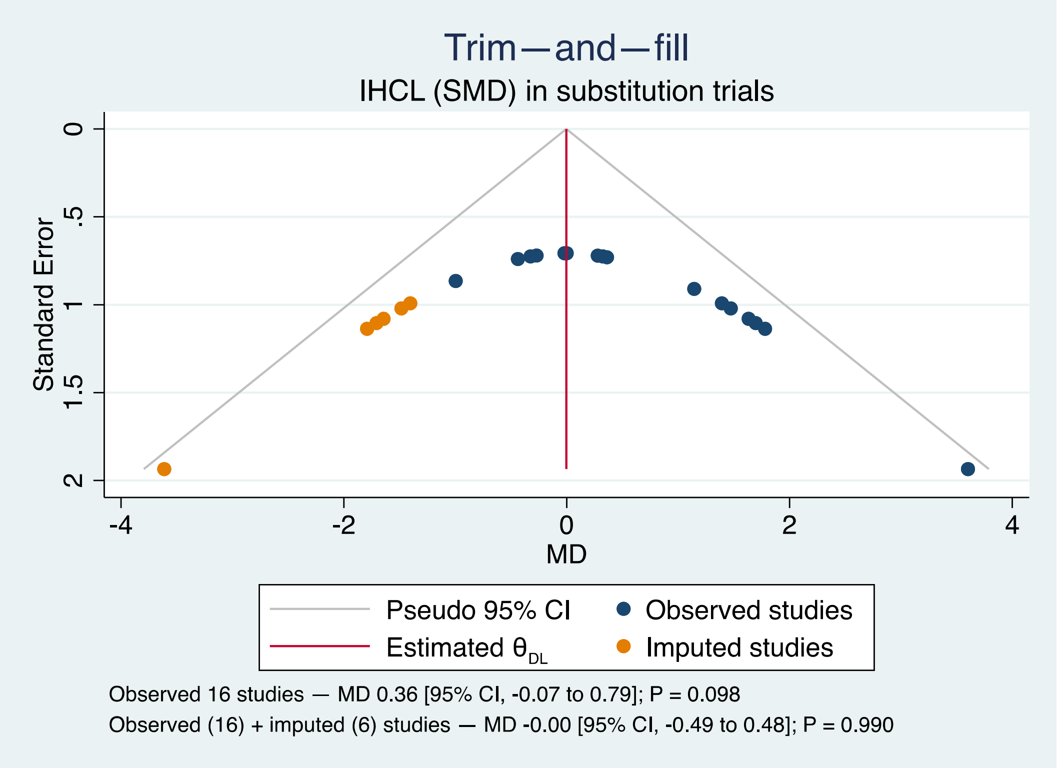

The vertical line represents the pooled effect estimate expressed as standardized mean difference. The diagonal lines represent the pseudo-95% confidence limits, the blue circles represent the effect estimate for each included study, and orange circles represent the effect estimate for each imputed "missed" study. Imputed random standardized mean difference is provided; when the imputed result differs from the primary result in either significance or magnitude (>1 MID = 0.26 SMD units for IHCL), this is considered evidence of small-study effects. CI=confidence interval; DL=DerSimmonian Laird; IHCL=intrahepatocellular lipid; SMD=standardized mean difference.

Supplementary Figure S97: Trim and Fill funnel plot for the effect of important food sources of fructose-containing sugars IHCL (SMD) in addition trials

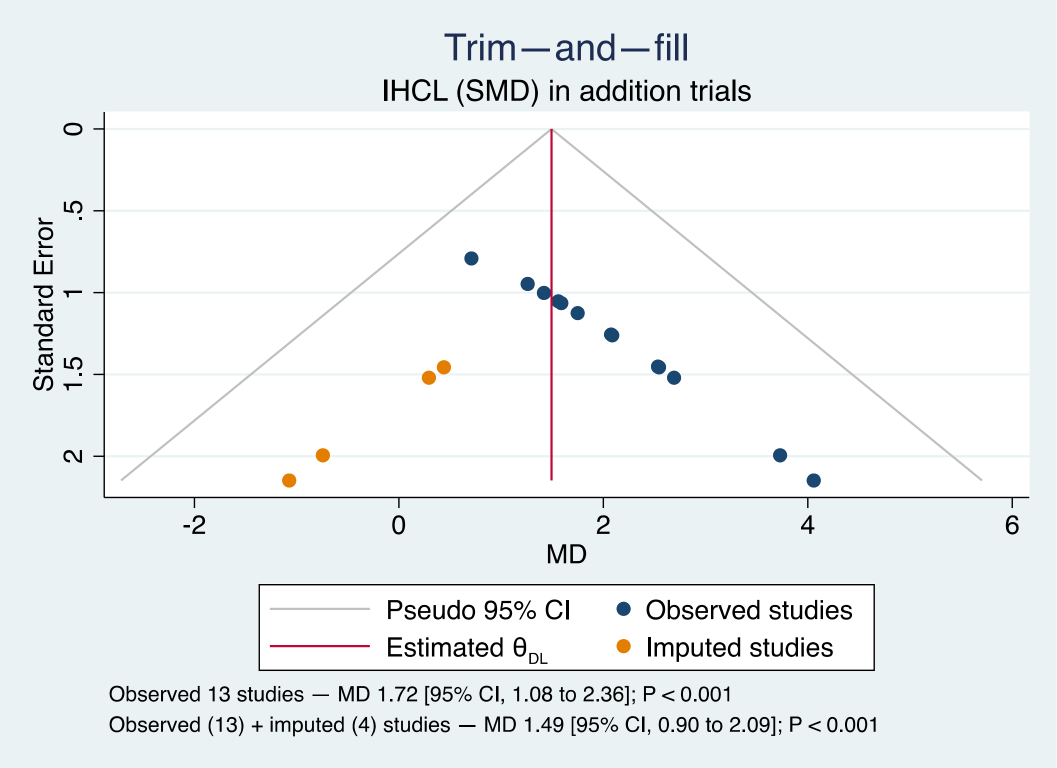

The vertical line represents the pooled effect estimate expressed as standardized mean difference. The diagonal lines represent the pseudo-95% confidence limits, the blue circles represent the effect estimate for each included study, and orange circles represent the effect estimate for each imputed "missed" study. Imputed random standardized mean difference is provided; when the imputed result differs from the primary result in either significance or magnitude (>1 MID = 0.26 SMD units for IHCL), this is considered evidence of small-study effects. CI=confidence interval; DL=DerSimmonian Laird; IHCL=intrahepatocellular lipid; SMD=standardized mean difference.

Supplementary Figure S98: Trim and Fill funnel plot for the effect of important food sources of fructose-containing sugars ALT (U/L) in substitution trials

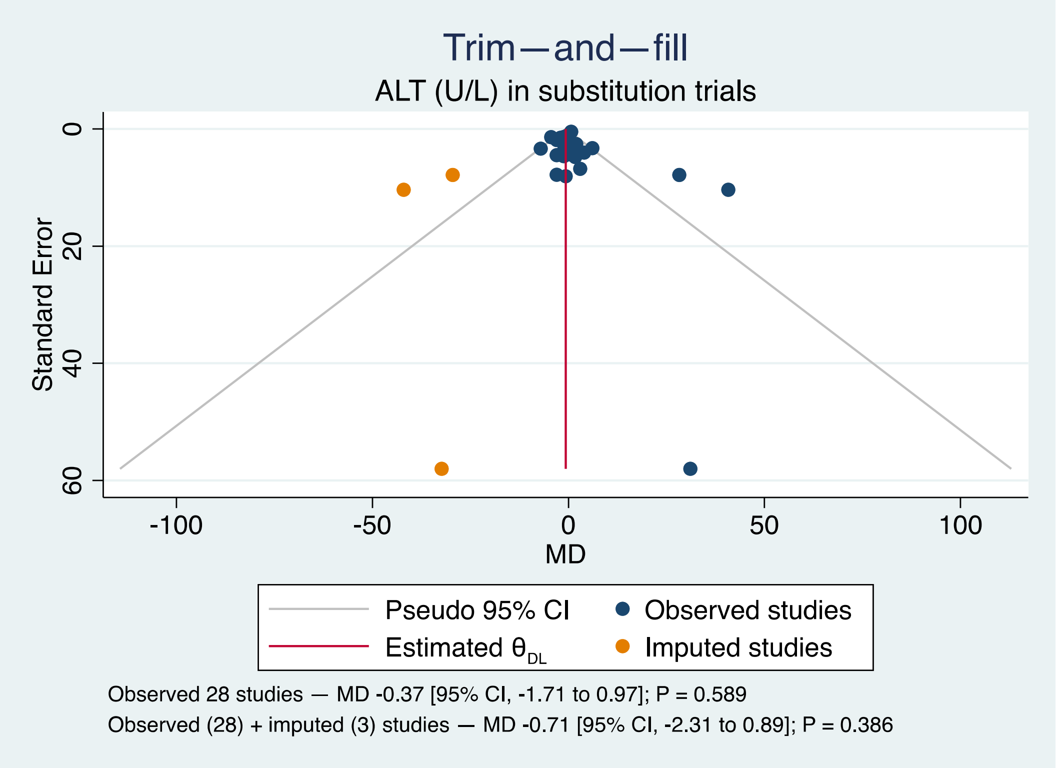

The vertical line represents the pooled effect estimate expressed as mean difference. The diagonal lines represent the pseudo-95% confidence limits, the blue circles represent the effect estimate for each included study, and orange circles represent the effect estimate for each imputed "missed" study. Imputed random mean difference is provided; when the imputed result differs from the primary result in either significance or magnitude (>1 MID = 2.85U/L for ALT(2)), this is considered evidence of small-study effects.

ALT=alanine aminotransferase; CI=confidence interval; DL=DerSimmonian Laird; MD=mean difference.

Supplementary Figure S99: Trim and Fill funnel plot for the effect of important food sources of fructose-containing sugars AST (U/L) in substitution trials

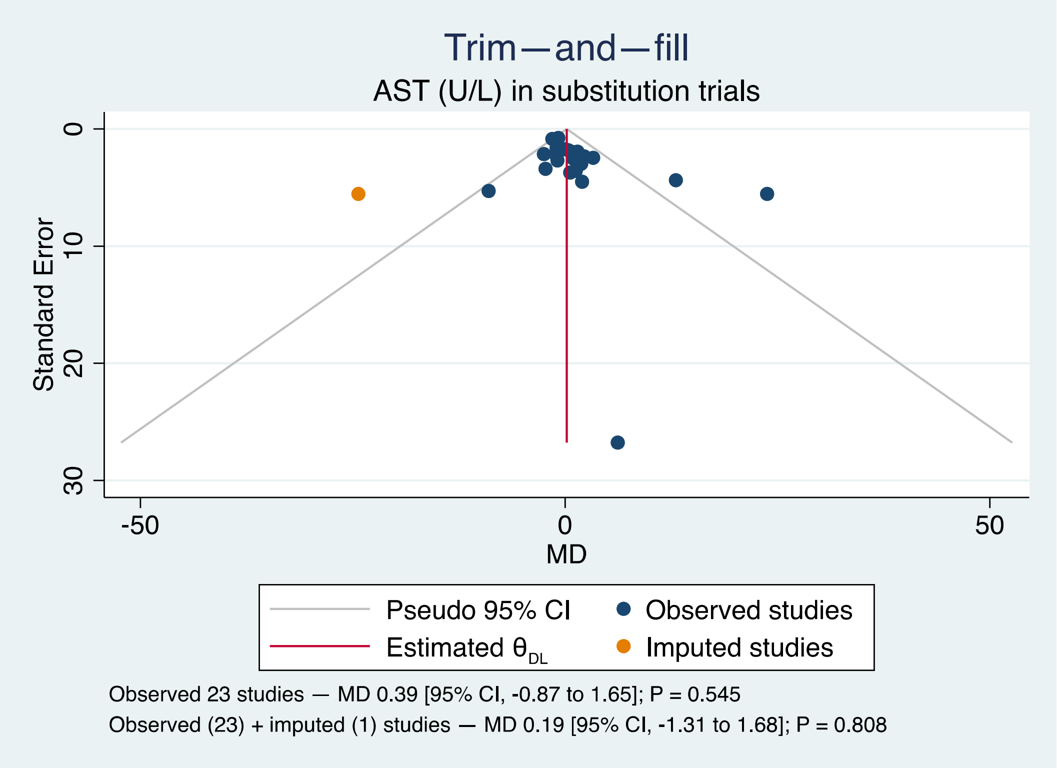

The vertical line represents the pooled effect estimate expressed as mean difference. The diagonal lines represent the pseudo-95% confidence limits, the blue circles represent the effect estimate for each included study, and orange circles represent the effect estimate for each imputed "missed" study. Imputed random mean difference is provided; when the imputed result differs from the primary result in either significance or magnitude (>1 MID = 2.55U/L for AST(2)), this is considered evidence of small-study effects.

AST=aspartate aminotransferase; CI=confidence interval; MD=mean difference.

Supplementary References

1. Page MJ, Moher D, Bossuyt PM, Boutron I, Hoffmann TC, Mulrow CD, et al. PRISMA 2020 explanation and elaboration: updated guidance and exemplars for reporting systematic reviews. Bmj. 2021;372:n160.
2. Tietz NW, Burtis, Carl A., Ashwood, Edward R. Tietz Textbook of Clinical Chemistry. 2 ed. Philadelphia: Saunders; 1994.
3. Santesso N, Glenton C, Dahm P, Garner P, Akl EA, Alper B, et al. GRADE guidelines 26: informative statements to communicate the findings of systematic reviews of interventions. J Clin Epidemiol. 2020;119:126-35.
4. Schünemann H BJ, Guyatt GOA. GRADE Handbook [Internet]. 2013 [cited 2018 Nov 10]. Available from: https://handbook-5-1.cochrane.org.
5. Balshem H, Helfand M, Schünemann HJ, Oxman AD, Kunz R, Brozek J, et al. GRADE guidelines: 3. Rating the quality of evidence. J Clin Epidemiol. 2011;64(4):401-6.
6. Wang YF, Chiavaroli L, Roke K, DiAngelo C, Marsden S, Sievenpiper J. Canadian Adults with Moderate Intakes of Total Sugars have Greater Intakes of Fibre and Key Micronutrients: Results from the Canadian Community Health Survey 2015 Public Use Microdata File. Nutrients. 2020;12(4).
